# Genome size and identification of abundant repetitive sequences in *Vallisneria spinulosa*

**DOI:** 10.7717/peerj.3982

**Published:** 2017-10-31

**Authors:** RuiJuan Feng, Xin Wang, Min Tao, Guanchao Du, Qishuo Wang

**Affiliations:** 1 Institute of Hydrobiology, Chinese Academy of Sciences, Wuhan, Hubei, China; 2 Jiangsu Tianshen Co., Ltd, Huai’an, Jiangsu, China; 3 Hongze Lake Fisheries Administration Committee Office of Jiangsu Province, Huai’an, Jiangsu, China; 4 School of Environmental Science and Engineering, Hubei Polytechnic University, Huangshi, Hubei, China; 5 Management Office of Yanlong Lake, Yancheng, Jiangsu, China

**Keywords:** Genome size, *C* value, Repetitive sequences, *Vallisneria spinulosa*, RepeatExplorer

## Abstract

*Vallisneria spinulosa* is a freshwater aquatic plant of ecological and economic importance. However, there is limited cytogenetic and genomics information on *Vallisneria*. In this study, we measured the nuclear DNA content of *Vallisneria spinulosa* by flow cytometry, performed a *de novo* assembly, and annotated repetitive sequences by using a combination of next-generation sequencing (NGS) and bioinformatics tools. The genome size of *Vallisneria spinulosa* is approximately 3,595 Mbp, in which nearly 60% of the genome consists of repetitive sequences. The majority of the repetitive sequences are LTR-retrotransposons comprising 43% of the genome. Although the amount of sequencing data used in this study was not sufficient for a whole-genome assembly, it could generate an overview of representative elements in the genome. These results will lay a new foundation for further studies on various species that belong to the *Vallisneria* genus.

## Introduction

*Vallisneria*, commonly called eelgrass, is a genus of freshwater aquatic plant. This genus consists of over 12 species worldwide and is widely distributed in tropical and subtropical regions of Asia, Africa, Europe, and North America (Les et al., 2008). *Vallisneria spinulosa* is of interest because of its importance in biodiversity and is of major human concern. The species has great impact on fisheries, wildlife, water resources, etc. (Baron et al., 2002). It usually occurs sympatrically in the middle to lower reaches of the Yangtze River in China (Wang et al., 2010) and is thought to be endemic to China (Xie, Deng & Wang, 2007). This species can provide food for waterfowl, nursery habitats for fish, and a substrate for invertebrates and may have a strong influence on water quality (Wang et al., 2010). Because of its ecological and economic importance, the interest of study *V. spinulosa* has raised greatly. Population genetic analysis revealed that *V. spinulosa* maintained high levels of genetic variation within populations and low subdivision among populations in ten lakes separated by approximately 900 km in the middle-lower reaches of the Yangtze River (Chen, Xu & Huang, 2007). Microsatellite primers were developed for studies of population genetic structure in the *Vallisneria* genus (Wang et al. , 2011). In other experiments, adaptive mechanisms in *V. spinulosa* operated via growth strategies and physiological responses in evading or adapting to Pb stress in a heterogeneously contaminated patch so that they could be chosen as suitable species in ecological restorations of heterogeneously contaminated habitats (Yan et al., 2006). However, the genetic and genomic knowledge of this genome is still limited. As an example, genome size of *Vallisneria spinulosa* is still unknown, whereas only the number of chromosomes was reported (Liang, 1991).

Recent advances in next-generation sequencing (NGS) technology and improvements in assembly strategies have resulted in the construction of complete genome sequences for more than 100 model and non-model plant species (Michael & VanBuren, 2015). Although the importance of aquatic plants has been noted because of their ability to yield a large amount of biomass without competing for agricultural land, relatively few aquatic plants have been subjected to genome sequencing projects. Aquatic plants which produce considerable amounts of biomass without competing with the agricultural land have drawn increasing attention. However, at present, relatively few aquatic plants except duckweeds (Wang et al., 2014; Van Hoeck et al., 2015) have been incorporated in sequencing projects exploring genome dynamics and the species’ roles in evolution and speciation. Undoubtedly, the eventual *Vallisneria spinulosa* genome sequence will serve as a reference genome for other species in the genus of *Vallisneria*. However, a complete genome sequencing project is still challenging with limited sequencing data. Apart from coding sequences that generally make up just a small fraction of the genome, repetitive sequences can account for more than 90% of a genome. *De novo* assembly and annotation of these repetitive sequences can be achieved effectively at reasonable costs by combining low-pass NGS (reviewed by Kelly & Leitch, 2011) and a series of software programs and pipelines (Novák, Neumann & Macas, 2010; Zytnicki, Akhunov & Quesneville, 2014).

Here we report the genome size, and compositions and fractions of various repetitive sequences in *Vallisneria spinulosa* genome. Our results lay a foundation for further researches on *Vallisneria spinulosa*. It will be also useful for genome studies of other species of the *Vallisneria* genus.

## Material and Methods

### Material

*Vallisneria spinulosa* was collected from E119°09.186′, N33°09.855′, Baima Lake, Jiangsu Province, China. Seedlings were grown in the green house until enough leaves were collected.

### *C*-value measurement by flow cytometry

For nuclear DNA content determinations, flow cytometric analysis was performed as described in Li & Arumugnathan (2000). The seeds of *Pisum sativum* (Cultivar Ctirad) were kindly provided by Prof. Ing. Doležel. Olomouc, Czech Republic. The *C*-value of *Pisum sativum* was used as a standard. Briefly, young *Vallisneria spinulosa* and *Pisum sativum* leaf tissues (approximately 30 mg of each sample) were hand-scraped on ice with a sharp razor blade in 1.5 ml of Tris-MgCl_2_ buffer (Pfosser et al., 1995). The nuclear suspension was filtered through a 30-µm mesh size nylon cloth into a labeled tube. Following filtration, the supernatant was centrifuged at 3,000 rpm at 4 °C for 1.5 min, and nuclei were resuspended in 450 µl of Tris-MgCl_2_ buffer. In this step, 50 µl of RNase A (50 µg/ml) was added to prevent the staining of double-stranded RNA. After resuspension, the suspension was stained with 5 µl of propidium iodide (PI) and was incubated in the dark at 37 °C for 15 min.

Nuclei were analyzed using FACSVerse™ flow cytometer (BD Biosciences, San Jose, CA, USA) with an excitation wavelength of 488 nm. Four independent samples were measured three times each. The nuclear DNA contents of each sample were calculated using the flowing formula:

\begin{eqnarray*}\text{Sample 2C DNA content}=\text{[(sample G1 peak mean) / (internal standards peak mean)]}\nonumber\\\displaystyle \quad \quad \quad \ast \text{internal standards DNA content}. \end{eqnarray*}

### NGS

Herbarium vouchers of *Vallisneria spinulosa* were prepared and deposited in the cytogenetic lab of Huai’an Research Centre, Institute of Hydrobiology, Chinese Academy of Sciences, China. Genomic DNA was extracted using a DNeasy plant mini kit from Qiagen, Valencia, CA, USA. Sequencing library was prepared using NEBNext^®^ Ultra™DNALibrary Prep Kit Illumina (New England, Biolabs, Ipswich, MA, USA). Paired-end sequencing (2X150 bp, 350–400 bp insert size) of total genomic DNA was performed by Novogene (Tianjin, China) on the Illumina HiSeq 2500 platform on a single lane. Clean sequencing data were supplied in FASTQ format without adapters. The raw data has been deposited in NCBI SRA database (accession number: SRR6038670).

### Data analysis

**Figure 1:**
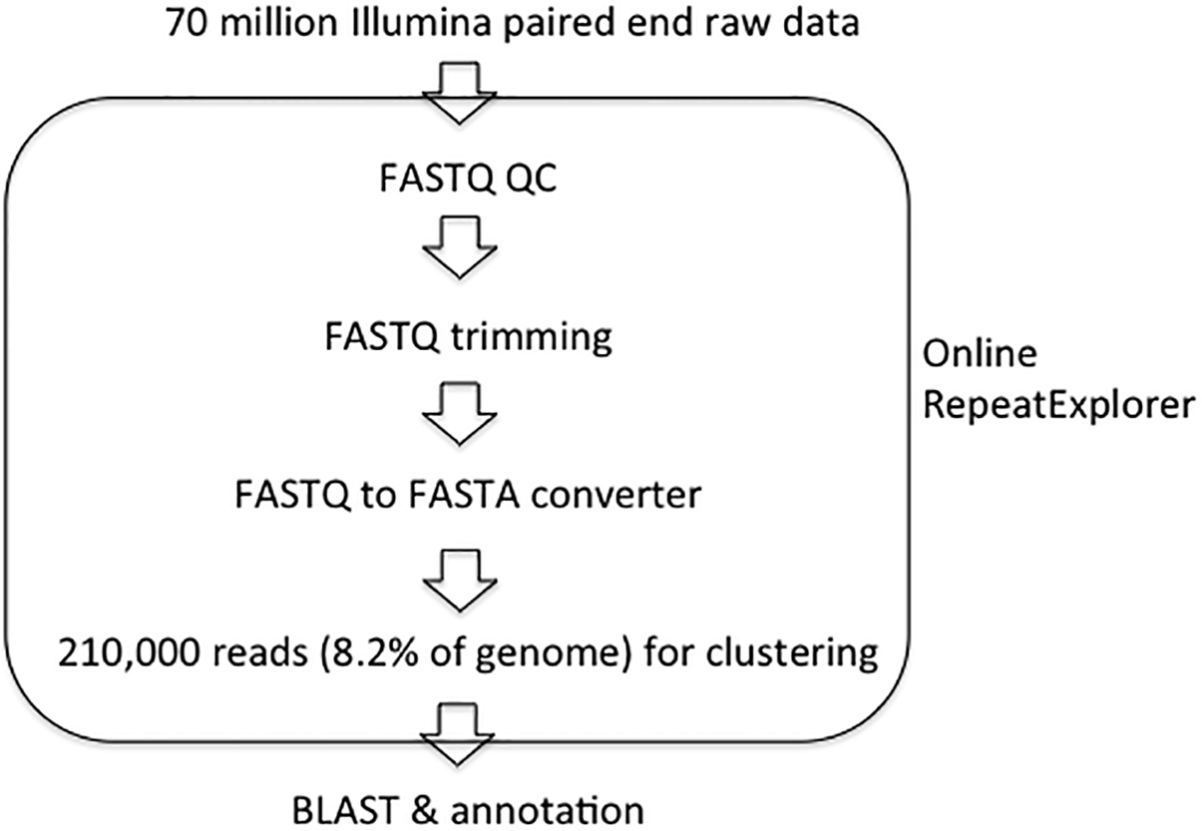
Workflow for repeat analysis in this study. Raw data from next-generation sequencing were uploaded to the Galaxy-based RepeatExplorer platform. The FASTQ QC: READ QC tool was then used to verify the quality of the reads before removing unnecessary sequences (i.e., adapter sequences) from the ends of each read using the FASTQ Trimmer tool. The QC analysis was then repeated, and the FASTQ to FASTA converter tool was used to convert each read into FASTA format. Using these DNA sequence reads as input, sequences undergo clustering, during which an “all-to-all” sequence comparison is performed, and similar sequences are grouped together into clusters.

The RepeatExplorer pipeline (Novak et al., 2013) (http://repeatexplorer.org/) was used to cluster next-generation sequencing reads into groups of similar reads and to assemble contigs from these reads. As shown in Fig. 1, a subset of Illumina paired-end reads from *Vallisneria spinulosa* were preprocessed, randomly selected and clustered into repeat families using RepeatExplorer (Novák, Neumann & Macas, 2010; Novak et al., 2013) with default setting. The minimum overlap length for clustering is 55 bp, and the minimal overlap for assembly is 40 bp. Repeat clusters with genome proportions of no less than 0.01% were detail annotated. Repeat clusters with known protein domains can be classified by RepeatExplorer pipeline directly. Other clusters were subjected to analysis with similarity searches against GenBank databases (Nt and Nr) using Blastn and Blastx (Altschul et al., 1990) with *E*-value at 1e^−5^ manually. The consensus DNA sequences of chromovirus were classified using reverse transcriptase (RT) domain (Macas, Neumann & Navrátilová, 2007). Protein sequences of RT cores were downloaded from Gypsy Database (GyDB) (Llorens et al., 2011) and used as custom database for BLAST. The RT cores of *Vallisneria spinulosa* were achieved by BLASTx using consensus sequences of clusters as query (*E*-value at 1e^−5^). Alignment of RTs was carried out with CLustalX (Thompson et al., 1997) and the phylogenetic trees were calculated in Geneious (version 5.5.6) using neighbor-joining method.

## Results and Discussion

### *C*-value measurement in *Vallisneria spinulosa*

**Figure 2:**
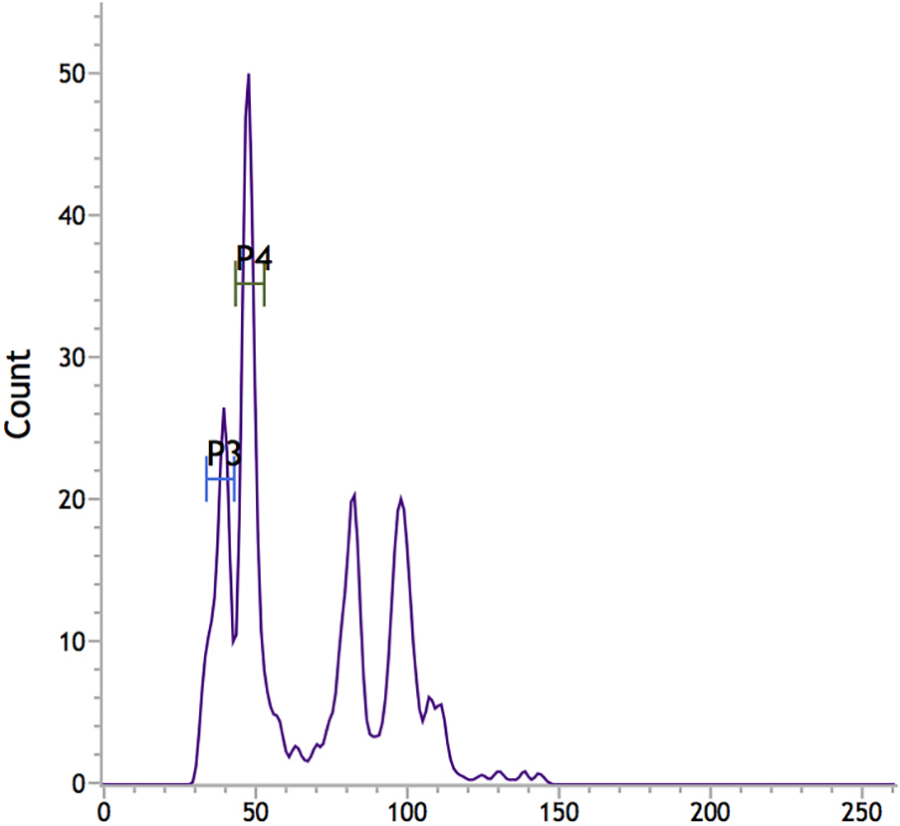
Fluorescence histograms of the genome size assessments in *Vallisneria spinulosa* by flow cytometry using propidium iodide. P3, *Vallisneria spinulosa*; P4, *Pisum sativum*.

*Vallisneria spinulosa* Yan (Hydrocharitaceae) is a submerged macrophyte, which is also an endemic and dominant species in the Yangtze River Basin in China. There has been no DNA content of the members of genus *Vallisneria* recorded until the present study (last accessed: 2017.08.30, http://data.kew.org/cvalues/). In this study, the nuclear DNA content of *Vallisneria spinulosa* was measured by flow cytometry. Fluorescence histograms representing genome size and the internal standards used are shown in Fig. 2. The haploid genome size value (1C) in *Vallisneria spinulosa* is 3.68 pg, which equals 3,595 Mbp (1 pg = 978 Mbp (Dolezel et al., 2003)). The genome size is in the range of intermediate genome sizes (3.51–13.99 pg) (Soltis et al., 2003). Compared to the other aquatic plant species with known genome size (Wang, Kerstetter & Michael, 2011), the genome size of *Vallisneria spinulosa* is at least twice larger than the duckweeds (Wang, Kerstetter & Michael, 2011). However, more genome size data is needed to compare the genome evolution and intraspecific variation in the *Vallisneria* genus.

### Graph-based sequence clustering and genome repeat composition analysis of *Vallisneria spinulosa* genome

The genome sizes of wetland plants are usually large (Hidalgo et al., 2015), making it difficult to analyze the genome using traditional molecular methods. Thus, we employed the latest next generation sequencing technology and a series of bioinformatics tools to reveal the genome structure of *Vallisneria spinulosa*. The recent advent of NGS methods has, for the first time, analyzed in details for any genome a possibility with reasonable costs. Although *Vallisneria spinulosa* is a non-model plant of great interest, there is no whole-genome sequencing project for *Vallisneria* proposed.

Table 1 shows the major cytogenetic and genomic parameters of *Vallisneria spinulosa*. Our Illumina sequencing returned 35,196,639 150-bp paired-end reads (70,393,278 reads in total) with more than 10 Gbp of raw DNA sequences. These sequence results constitute an essential genome resource for further study of this species in the future.

One of the aims of this study was to annotate the repetitive sequences of *Vallisneria spinulosa* to investigate the structure of its genome. RepeatExplorer (Novák, Neumann & Macas, 2010), a graph-based clustering approach to identify repetitive sequences, was employed to analyze the repetitive sequences in *Vallisneria spinulosa* genome.

**Figure 3:**
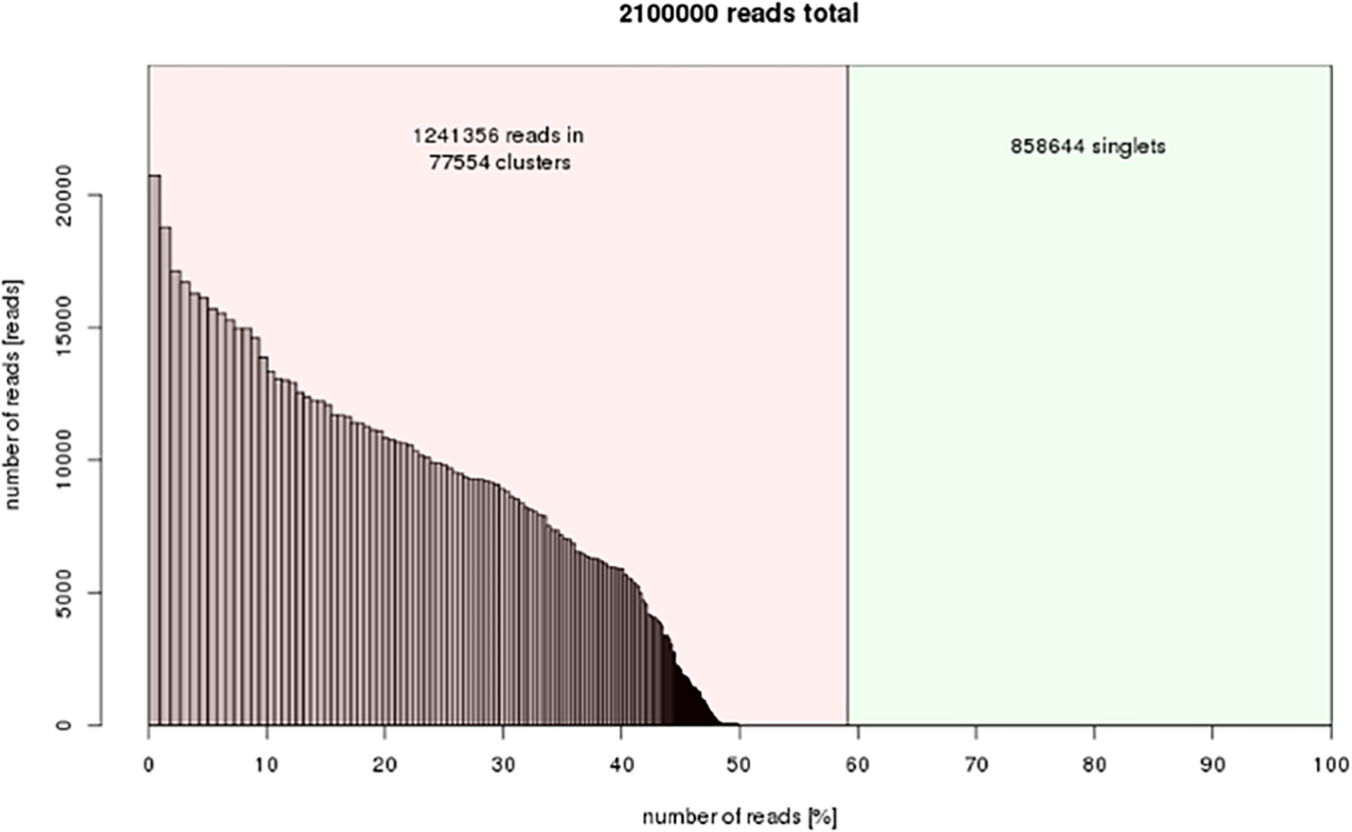
Repeat composition of clusters generated in RepeatExplorer (similarity-based partitioning) of 2.1 M reads. *x*-axis: cumulative proportion of clusters of the genome. *y*-axis: numbers of reads. The color of the bars shows the types of repetitive elements.

**Table 1:** Cytogenetics, genomic and sequencing features of *Vallisneria spinulosa*.

Species	*Vallisneria spinulosa*
Chromosome number (2n)	20
1C value (pg)	3.68
Genome size (Mbp)	3,595
Sequenced read number	70,393,278
Genome coverage (%) of reads analyzed	18
Repetitive DNA cluster numbers (≥0.01% genome proportion)	184

As shown in Fig. 1, more than 70 million paired end reads from next-generation sequencing were uploaded to the Galaxy-based RepeatExplorer platform (Novak et al., 2013). The FastQC tool was then used to verify the quality of the reads (i.e., adapter sequences, etc.). Illumina sequencing can cause bias in the beginning and end of reads (Hansen, Brenner & Dudoit, 2010). Therefore, we trimmed the first 9 bp of each read based on the FastQC results. The FASTQ to FASTA converter tool was then used to convert each read into FASTA format. RepeatExplorer can predict the repeat composition from a typical 2–5% of genome coverage sequencing data (Novak et al., 2013). Therefore, 2,100,000 DNA sequence reads were selected randomly as input, which was equal to 8.2% of the predicted genome (141 bp × 2,100,000/3,595 Mbp × 100% of the genome). All paired sequences underwent clustering, during which an “all-to-all” sequence comparison was performed, and similar sequences were grouped together into clusters. The genome proportion of each cluster was calculated as the percentage of reads. In our study, the RepeatExplorer results in 1,241,356 reads in 77,554 clusters and nearly 60% of the genome were determined to be repetitive sequences (Fig. 3), in which there were 184 separated clusters with genome proportions of no less than 0.01% each. The top 184 clusters in total represented approximately 48% of the genome (Fig. 3 and Table 2). The graphical figures and three most abundant consensus sequences of each cluster could be found in Fig. S1 and Data S1 separately. Based on the analyzed data, the frequency of singletons should represent the low copy fraction of the genome, which resulted in approximately 31% of genome in *Vallisneria spinulosa*.

**Table 2:** Repeat composition of the *Vallisneria spinulosa* genome estimated from the Illumina sequencing data.

Repeat type	Lineage	Genome proportion (%)
Retroelements		
Ty-3/Gypsy		
	Chromovirus	31.582
	Tat/Ogre	0
	Athila	0
	Unclassified	0.020
Ty-1/Copia		
	Maximus	6.427
	Angela	0
	Bianca	0
	Tork	1.905
	Ivana/Oryco	0
	AleII	0.928
	TAR	0.185
	AleI/Retrofit	1.812
	Unclassified	0.161
LINE		0.460
SINE		0
Other		0.023
DNA transposons		0.514
Satellite repeats		0.013
rDNA		0.351
Unclassified		0.977
Chloroplast		2.704
Total		48.062

**Table 3:** Repeat composition of *Vallisneria spinulosa* compared with other monocot plant genomes.

Genome size		*Vallisneria spinulosa* 3,595 Mbp	*Sorghum bicolor* 730 Mbp	*Lemna minor* 481 Mbp	*Oryza sativa* 430 Mbp	*Brachypodium distachyon* 355 Mbp	*Spirodela polyrhiza* 158 Mbp
Retroelements (%)		43.50	54.50	31.20	32.10	23.30	13.06
	LTR	43.02	54.47	29.57	30.85	21.39	13.06
	Gypsy	31.60	19.00	10.59	9.06	13.46	6.06
	Copia	11.42	5.18	18.79	3.32	5.13	1.72
	Gypsy/copia ratio	2.77	3.67	0.56	2.73	2.62	3.50
	non-LTR	0.46	0.06	1.62	1.24	1.94	n.a.
Transposon (%)		0.51	7.50	5.08	10.10	4.80	n.a.

**Notes.**

Values are represented as percentage of genome.

### Characterization of the LTR-retrotransposons of *Vallisneria spinulosa*

The top 184 clusters were further characterized and annotated by searching the sequence-similarity of the assembled contigs against GenBank using Blastn and Blastx (Altschul et al., 1990). No coding gene was found except ribosomal RNA gene and plastid genes among top 184 clusters. The genome proportions of each type of repetitive sequence in *Vallisneria spinulosa* are shown and detailed in Table 2. The majority of the repeats are LTR-retrotransposons, representing in 43% of the genome. Concerning the two main superfamilies of LTR-retrotransposons, Gypsy-related contigs are more represented than Copia ones in this species. Gypsy elements belong to three main lineages, while Copia ones belong to seven lineages (Wicker et al., 2007). The greatest majority of repeats divided by lineage is the Chromovirus, in which repeats comprise greater than 31.5% of the genome proportion. Furthermore, we compared *Vallisneria spinulosa* Chromoviruses with known plant clades (Llorens et al., 2011) (Fig. S2). All the Chromovirus in *Vallisneria spinulosa* are from Tekay (Del) clade. It has been reported that Tekay is the most abundant Chromovirus in Orobanche (Piednoël, Carrete-Vega & Renner, 2013) and banana (Hribová et al., 2010). But in *Rumex acetosa*, CRM clade is the more abundant than rest of Chromovirus (Steflova et al., 2013). The most abundant Ty-1/Copia element is Maximus, which accounts for 6.4% of the genome proportion. The genome consists of 0.46% LINEs, 0.514% DNA transposons, 0.013% Satellite repeats and 0.351% rDNA. Several comparative analysis of repeats from different species revealed that there are quantitative differences and sequence variations detected for classified repeat families (Novák et al., 2014; Kelly et al., 2015; Macas et al., 2015). But it is still possible that some differences in repeat abundance estimates can be also attributed to incompleteness of assembly and biased composition of sequences in the genome assembly (Novák et al., 2014). In addition, 2.7% chloroplast DNA was found. It has been reported that chloroplast DNA could be found integrated into the nuclear genome (Roark et al., 2010). However, the significantly high proportion of chloroplast DNA suggests that it might have come from the DNA extraction process. Since it was reported B chromosomes in plant are enriched with chloroplast and mitochondria DNA (Klemme et al., 2013). FISH using plastid DNA as a probe could be performed to test either possibility. Many elements in the repeat lineages were not found by our analysis, such as Tat/Ogra, Athila, Angela, Bianca and Ivana/Oryco, as we only annotated repeats with genome proportions greater than 0.01% of the genome. Therefore, these elements are likely to be present in the genome, but with minor proportions of each. Moreover, approximately 1% of the genome is unclassified to any of major lineages according to the RepeatExplorer analysis. More extensive sequencing efforts will be required to conclusively annotate these repeats. Additionally, we compared repeat composition of *Vallisneria spinulosa* with other well-studied monocot plant genomes (Table 3). It has been reported that in small-size (<1 Gb) plant genomes, there is a linear dependency between genome size and LTR retrotransposon content (Table 3) (Wang et al., 2014). The genome size of *Vallisneria spinulosa* is almost five times as big as that of *Sorghum bicolor*. But the proportion of retroelements in *Vallisneria spinulosa* is less than that of *Sorghum bicolor*. *Vallisneria spinulosa* appear to contain the highest proportion of Gypsy elements compared to other plant genomes (Table 3). But the Gypsy/copia ratio is not as high as *Sorghum bicolor* (Table 3). The proportion of other transposons in *Vallisneria spinulosa* is much lower than that of the rest analyzed plant genomes. It is possible that most of the transposons are not present as high copy number in the genome of *Vallisneria spinulosa*. They might be in the repeat catalogue that contains the repetitive sequences with the grequency of less than 0.01% of the genome. Differences in TE content, especially the LTR retrotransposons make up the difference in genome size variation in angiosperms. For example, repeat content explains 94.5% of the genome size difference between *Spirodela polyrhiza* (2*n* = 40; 158 Mbp) and *Lemna minor* genome (2*n* = 40; 481 Mbp). But our clustering data only explained approximately 60% of the genome in *Vallisneria spinulosa* (Fig. 3). There are considerable amounts of sequences are neither repetitive sequence detected by RepeatExplorer nor genes. Because genomic repeat abundances contain phylogenetic signatures (Dodsworth et al., 2015), the dynamics of LTR retrotransposons and their contributions to genome evolution could be used to analyze the speciation of *Vallisneria* in the future once more sequence information is available for this genus. Since *Vallisneria spinulosa* is a member of the order Alismatales, which are basal monocots, genome sequence of *Vallisneria spinulosa* can also contribute to the understanding of the genome evolution of monocots as well.

**Figure 4:**
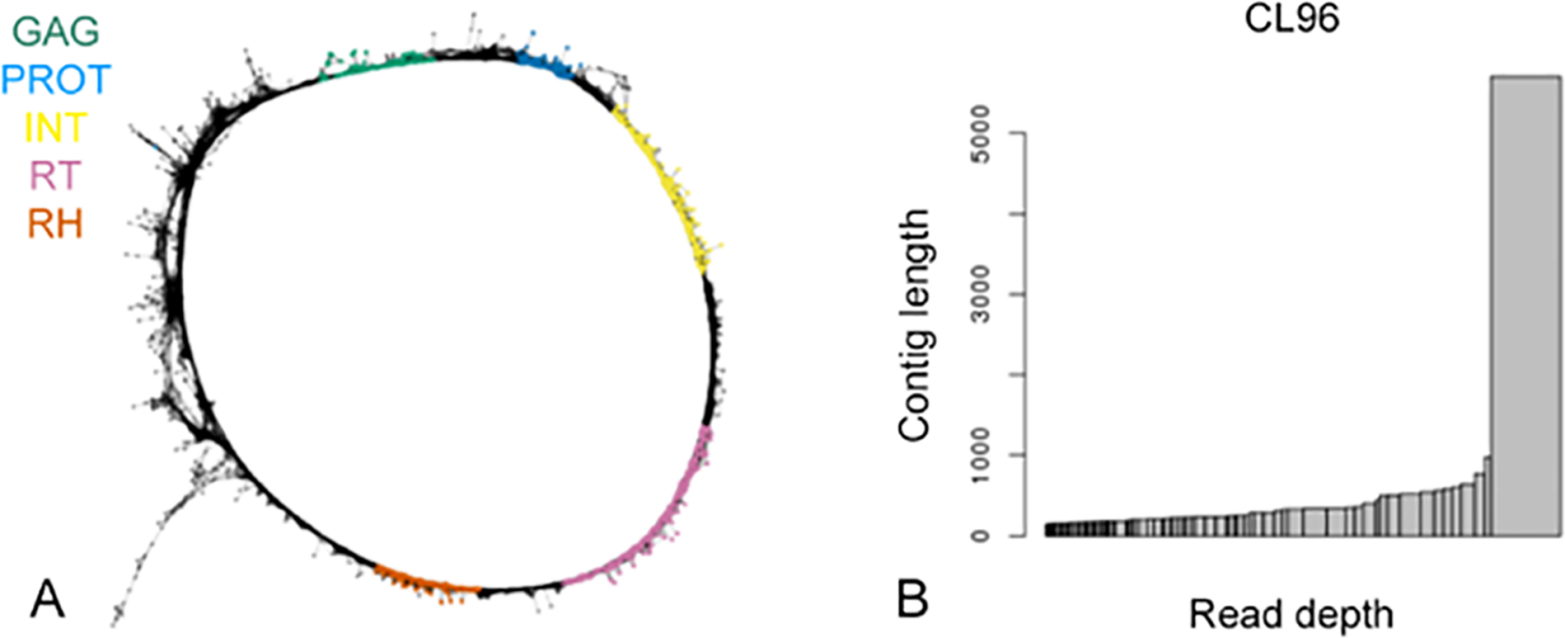
Annotation of a TAR element VsCL96. (A) Graphical 2D projection of the structure of VsCL96, a Ty1/copia TAR repeat with complete protein domains including GAG, PROT, INT, RT and RH in order. Each dot represents a sequence read and each line represents similarity hits between dots. The protein domains are highlighted in different colors. (B) Read depth of each contig within CL96. *x*-axial: read depth, *y*-axial: contig length.

### Annotation of a typical Ty1/copia TAR repeat

The position of the reverse transcriptase (RT) gene in relation to the integrase (INT) gene of *pol* was used to classify the retrotransposon families into Ty1-copia (PROT-INT-RT) and Ty3-gypsy (PROT-RT-INT) (Wicker et al., 2007). Figure 4A shows the plotted graph of VpCL96, in which each dot represents a sequence read and each line represents similarity hits between dots. This shows a Ty1/copia TAR repeats with complete typical copia protein domains, including GAG, PROT, INT, RT and RH in order. The LTRs at both ends have high similarity as shown in a circle graph (Fig. 4A). It has been reported that 52 copia families from Triticeae, rice, and Arabidopsis could be classified into six ancient lineages (Bianca, TAR, Angela, Ale, Ivana, and Maximus). While many of the contigs from RepeatExplorer clusters are truncated without having the protein domains. CL96 is intact, with a dominant contig that is shown as a wider bar in Fig. 4B. Additionally, the length of the contig is greater than 5,000 bp (Fig. 4B).

Our database of repetitive sequences can be a useful resource for further investigation of localization and visualization of these sequences in the chromosomes of *Vallisneria spinulosa* chromosomes (Becher et al., 2014).

## Conclusion

Although the amount of sequencing data of *Vallisneria spinulosa* used in this study was not sufficient for whole-genome assembly, it still enabled us to generate an overview of representative elements in the genome. We also measured the genome size of this aquatic plant. The genome size and the genomic data described here will become a valuable resource for further studies of *Vallisneria spinulosa* and other species of the genus.

##  Supplemental Information

10.7717/peerj.3982/supp-1 Figure S1 The graphical figures of each annotated cluster

10.7717/peerj.3982/supp-2 Figure S2 Phylogenetic relationships in Ty3/Gypsy Chromoviruses elements in *Vallisneria spinulosa*

10.7717/peerj.3982/supp-3 Data S1 Three most abundant consensus sequences of each annotated cluster

## Additional Information and Declarations

### DNA Deposition

The following information was supplied regarding the deposition of DNA sequences: NCBI SRA: SRR6038670.

### Data Availability

The following information was supplied regarding data availability: Wang, Qishuo (2017): F_H33NLALXX_L3_1.clean.fq.gz. figshare. https://dx.doi.org/10.6084/m9.figshare.5387008.v1. Wang, Qishuo (2017): F_H33NLALXX_L3_2.clean.fq.gz. figshare. https://dx.doi.org/10.6084/m9.figshare.5387041.v1.
