# Supplementary material for: Genome size and identification of abundant repetitive sequences in Vallisneria spinulosa
Source: PeerJ. 2017 Oct 31;5:e3982. doi: 10.7717/peerj.3982 (PMC5669256; doi:10.7717/peerj.3982)
Supplement: Figure S1 [file peerj-05-3982-s001.pdf]

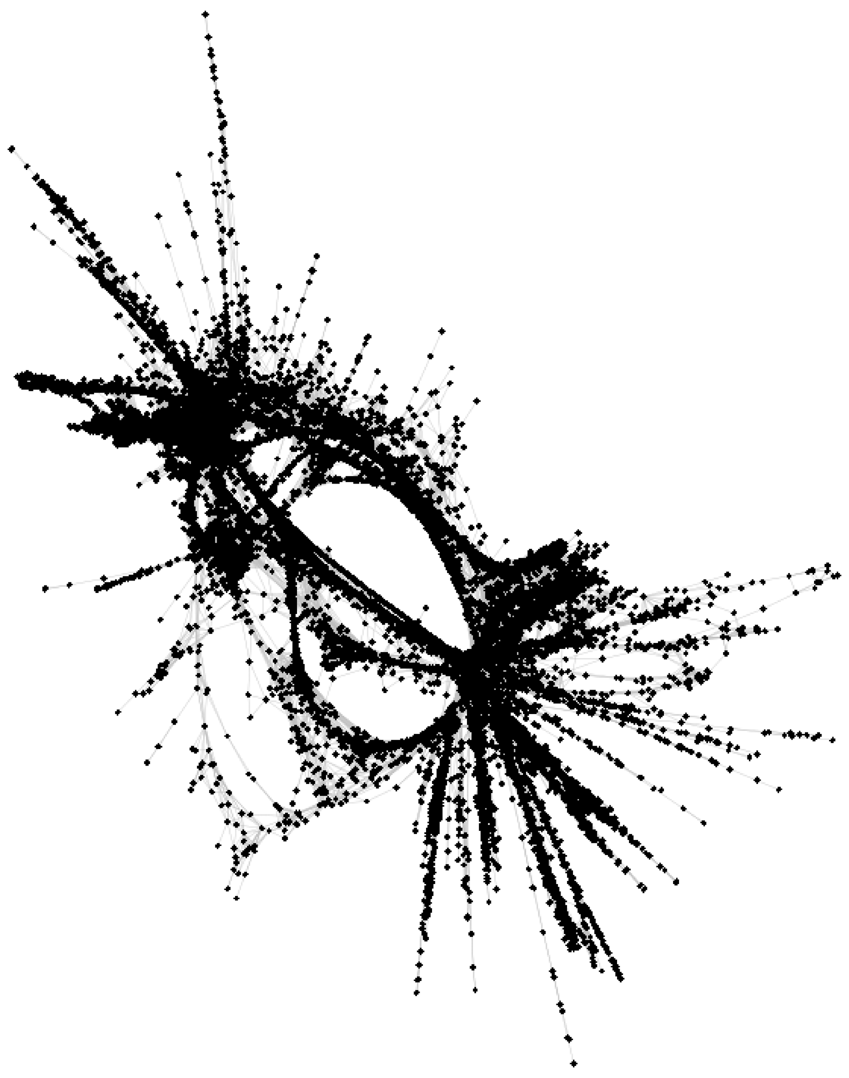

CL1

Number of reads: 20732
Number of pairs: 1126241
Density: 0.005241
Diameter: NA
Mean edge weigth: 156.78
Max. degree: 829

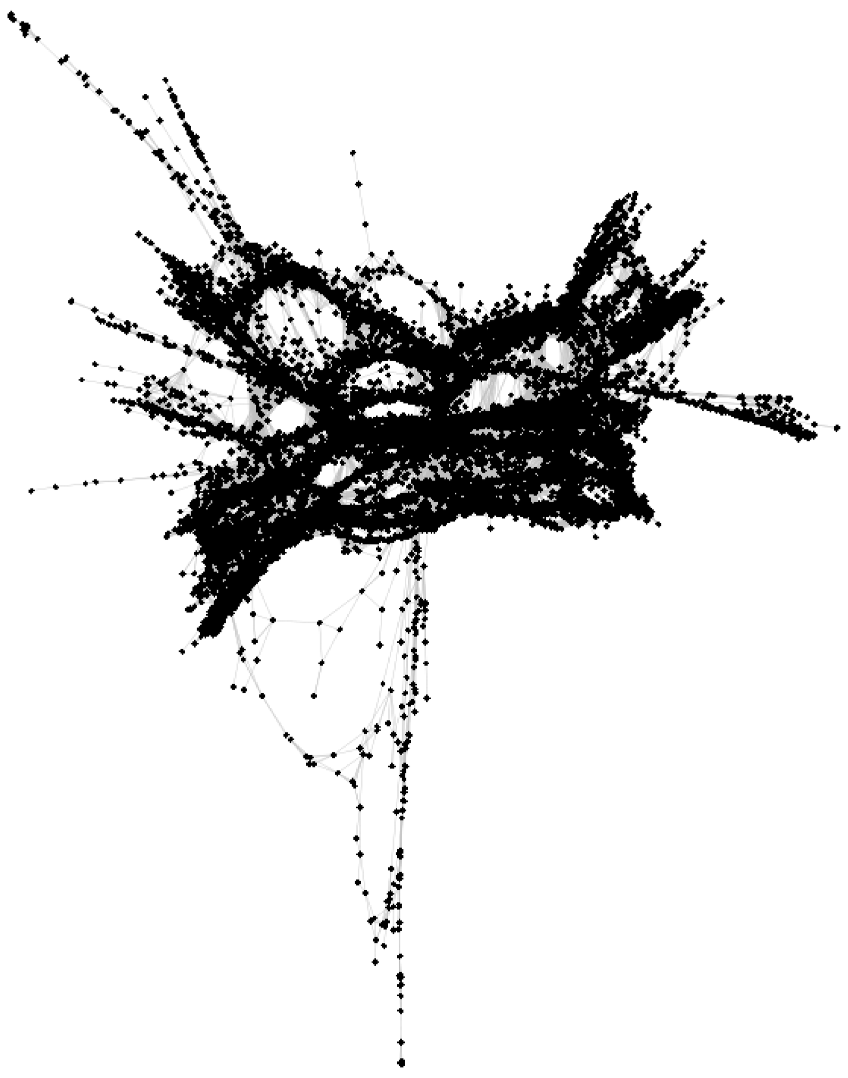

CL2

Number of reads: 18778
Number of pairs: 985935
Density: 0.005592
Diameter: NA
Mean edge weigth: 154.75
Max. degree: 484

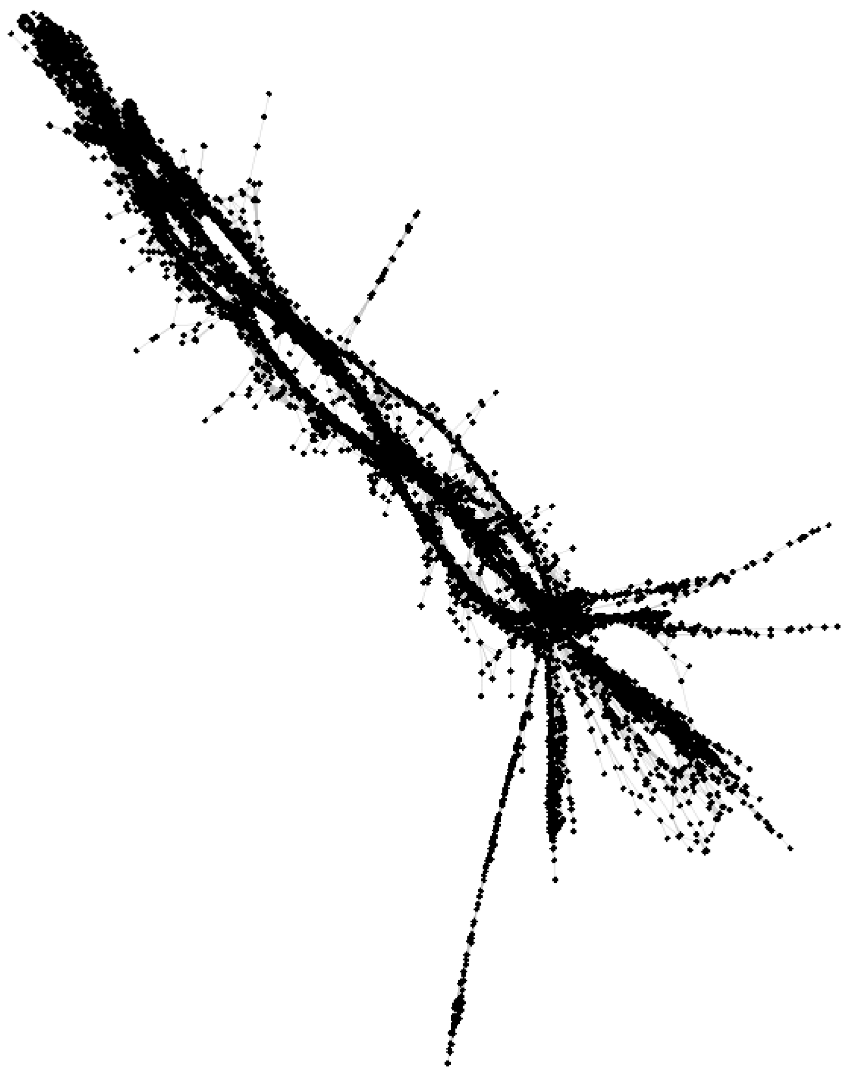

CL3

Number of reads: 17131
Number of pairs: 1049951
Density: 0.007156
Diameter: NA
Mean edge weigth: 160.3
Max. degree: 412

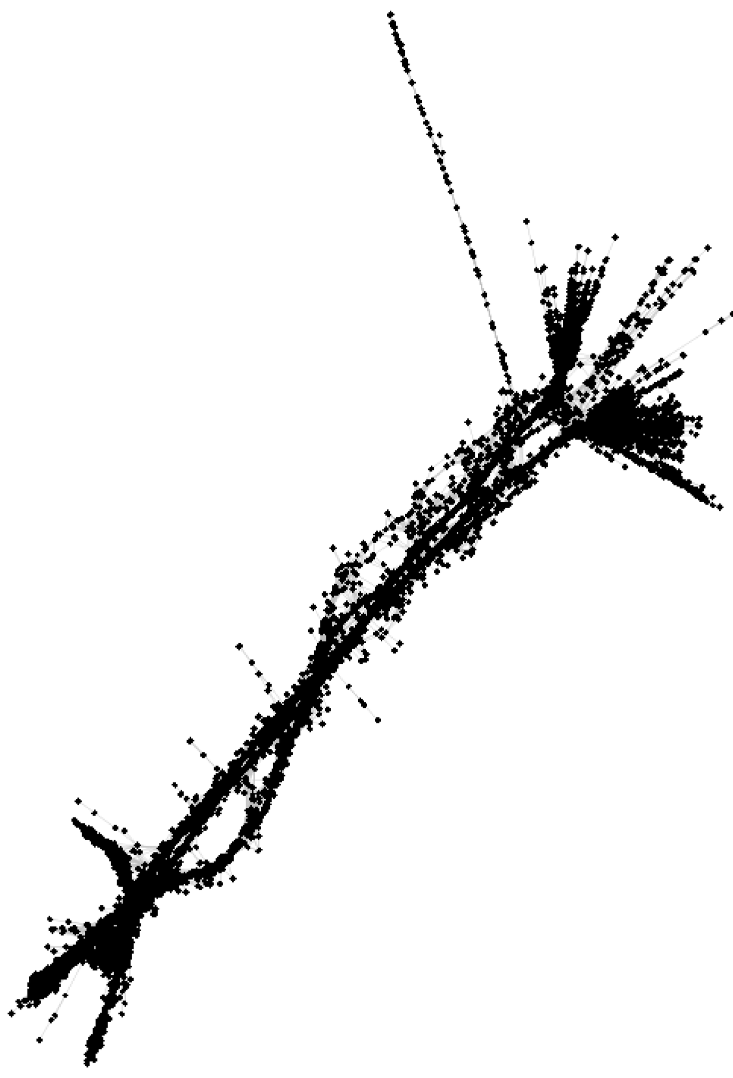

CL4

Number of reads: 16708
Number of pairs: 1235523
Density: 0.008852
Diameter: NA
Mean edge weigth: 160.24
Max. degree: 531

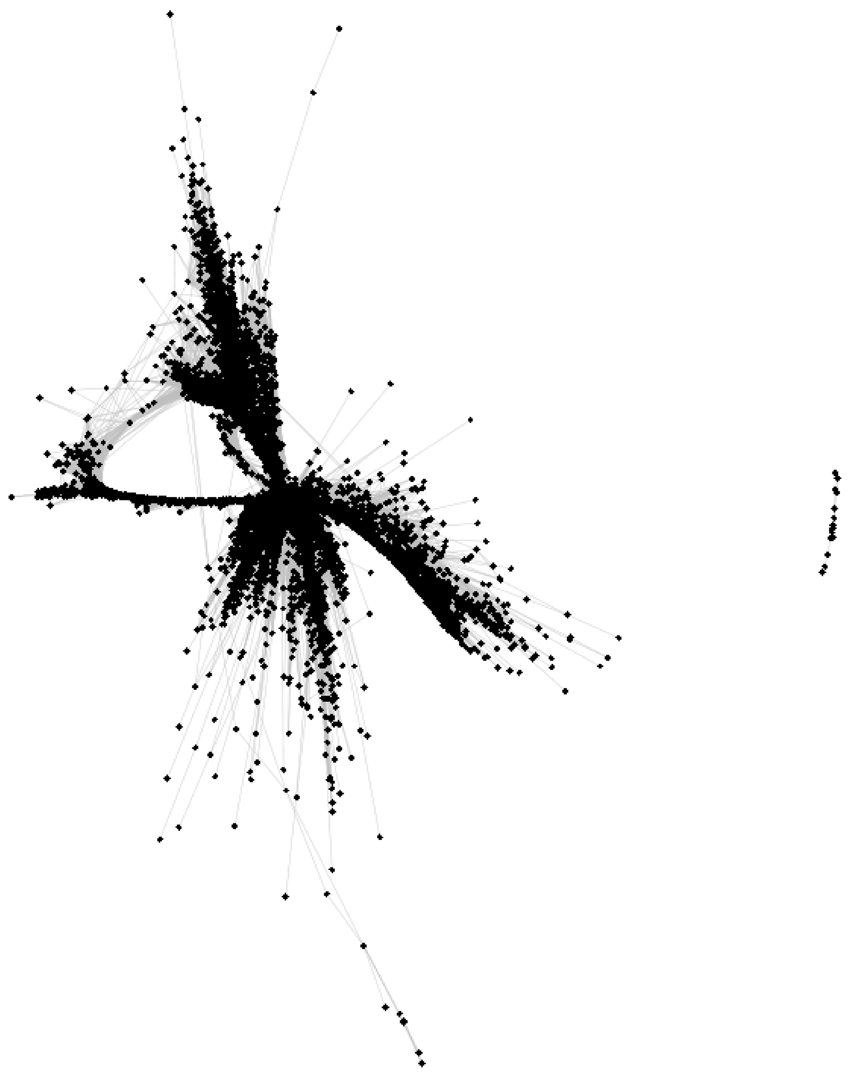

CL5

Number of reads: 16288
Number of pairs: 5218529
Density: 0.03934
Diameter: NA
Mean edge weigth: 156.78
Max. degree: 3199

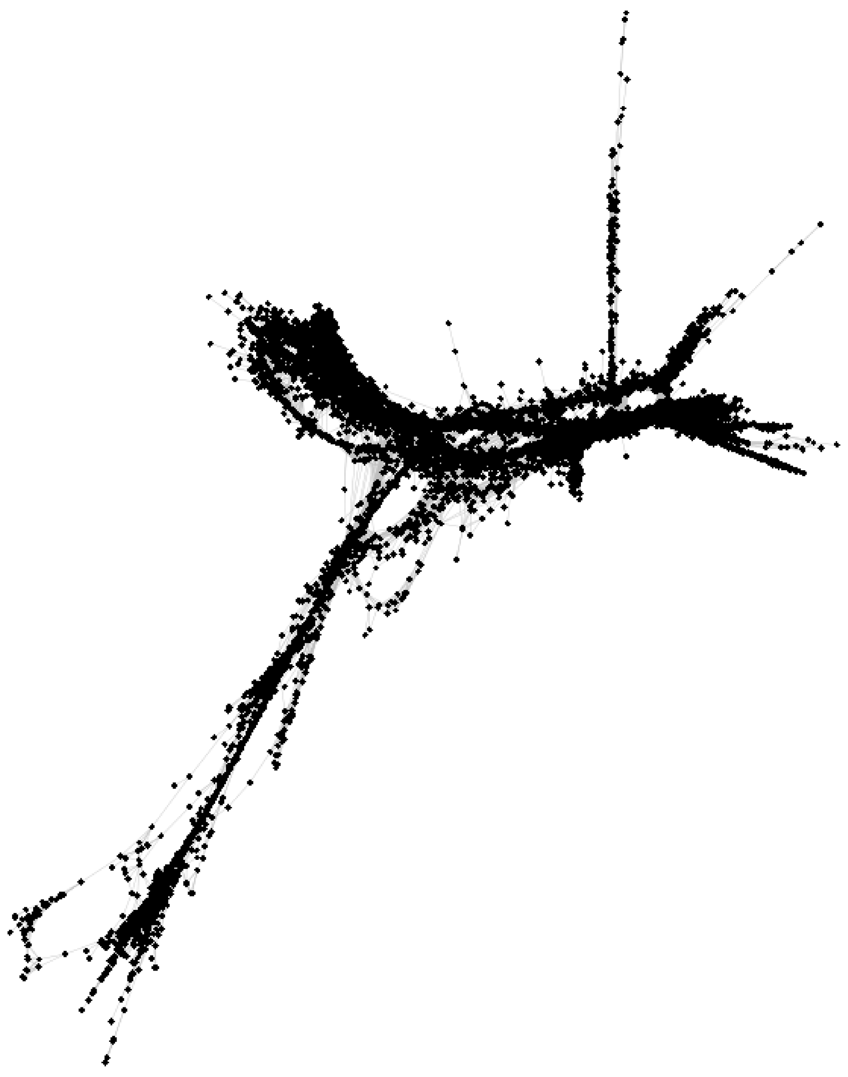

CL6

Number of reads: 16110
Number of pairs: 2024528
Density: 0.0156
Diameter: NA
Mean edge weigth: 159.68
Max. degree: 1095

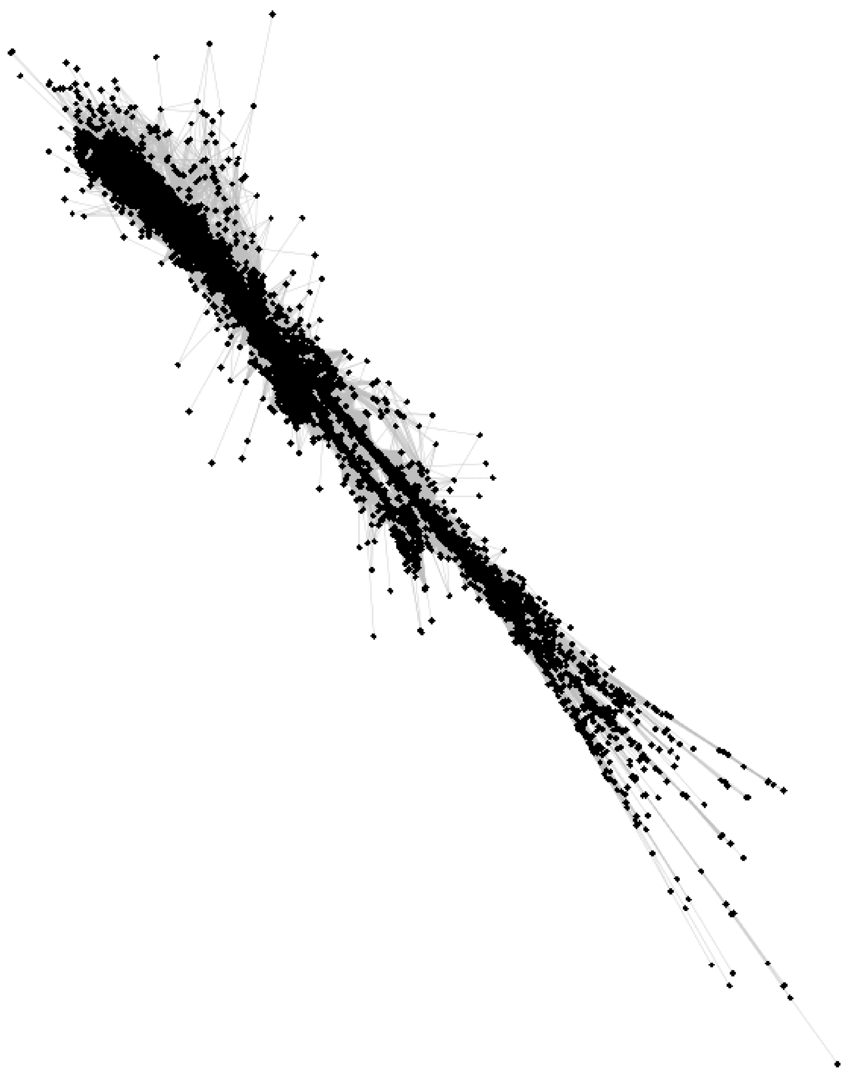

CL7

Number of reads: 15720
Number of pairs: 7636138
Density: 0.06181
Diameter: NA
Mean edge weigth: 160.97
Max. degree: 2458

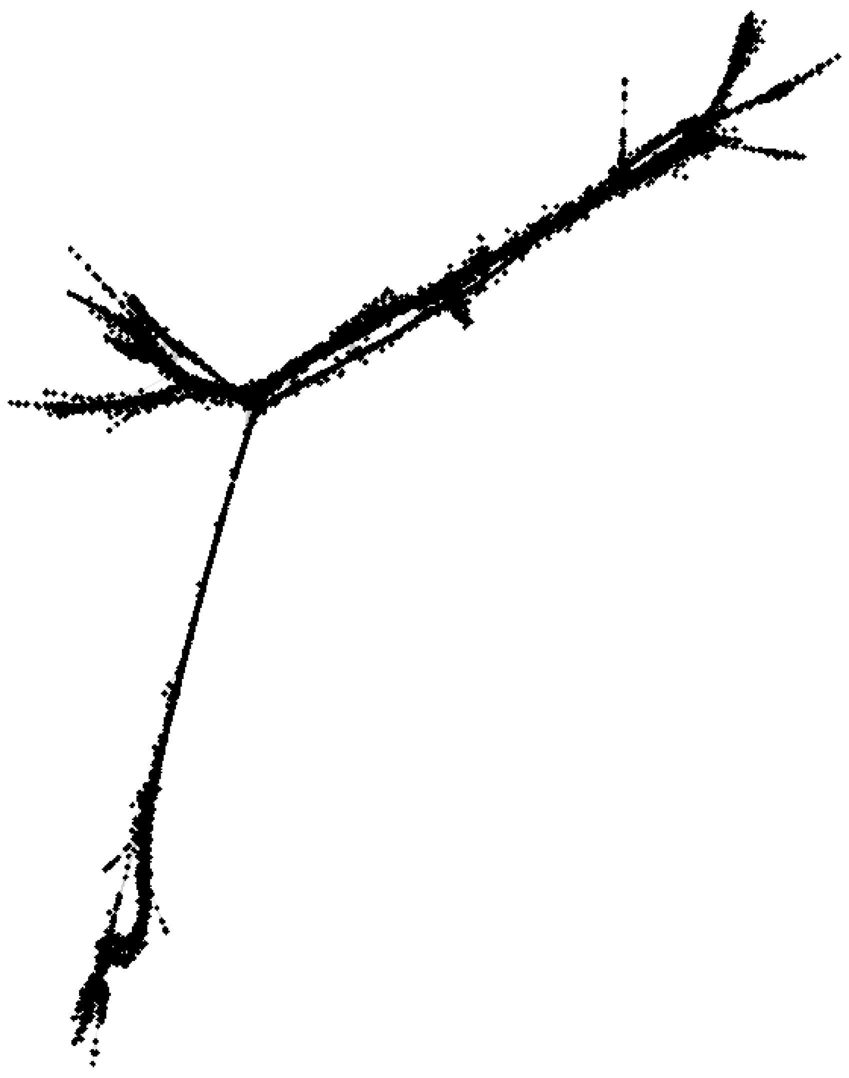

CL8

Number of reads: 15537
Number of pairs: 606351
Density: 0.005024
Diameter: NA
Mean edge weigth: 165.32
Max. degree: 279

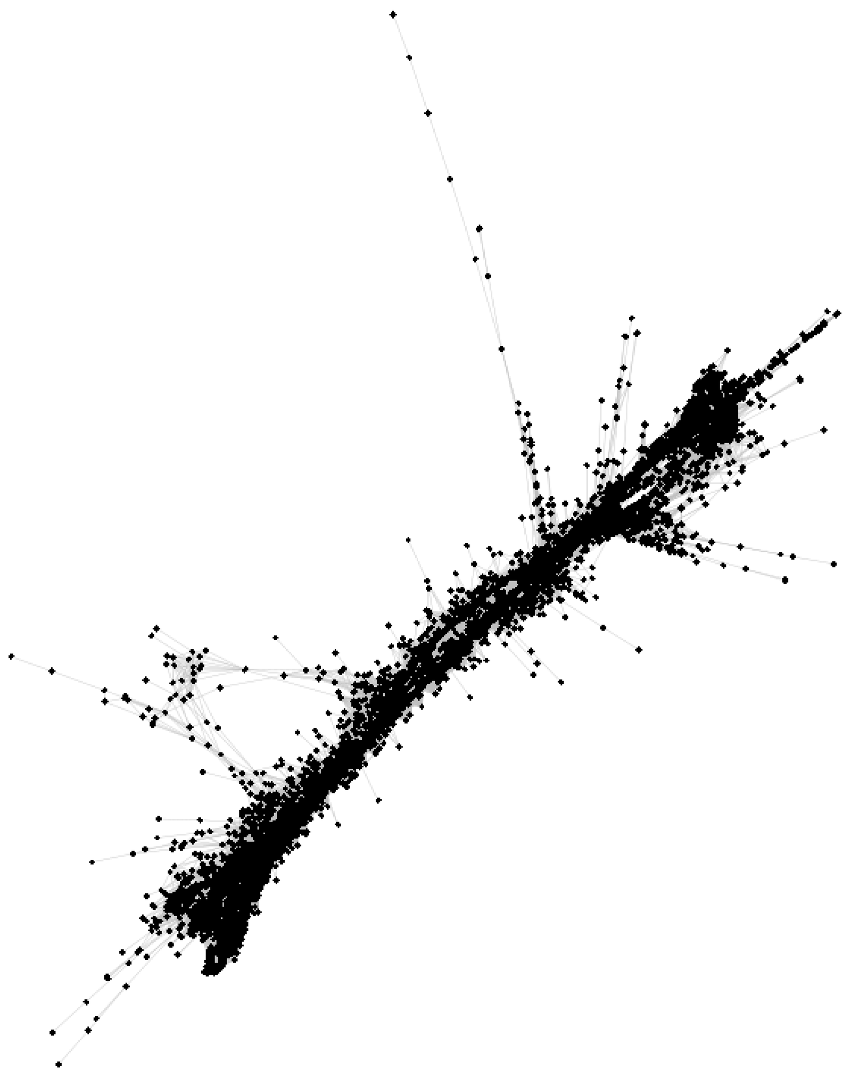

CL9

Number of reads: 15281
Number of pairs: 3630522
Density: 0.0311
Diameter: NA
Mean edge weigth: 163.69
Max. degree: 1194

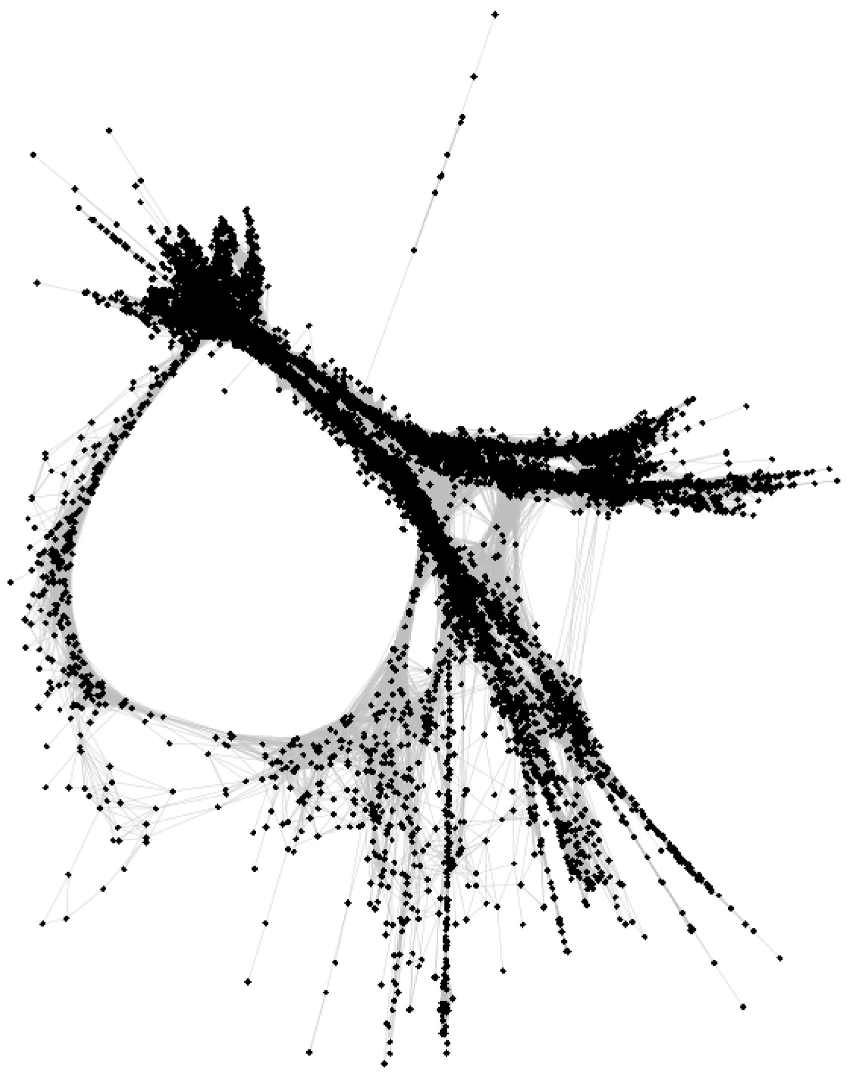

CL10

Number of reads: 14982
Number of pairs: 3261777
Density: 0.02907
Diameter: NA
Mean edge weigth: 156.98
Max. degree: 1681

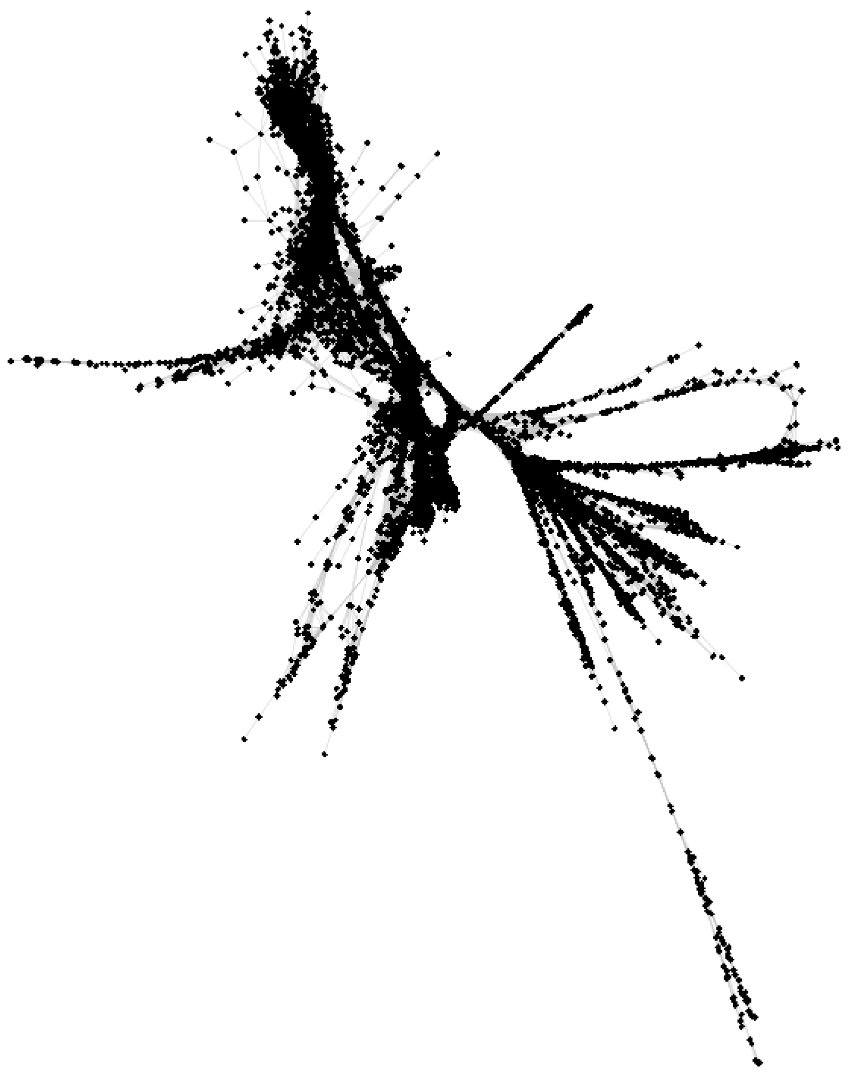

CL11

Number of reads: 14981
Number of pairs: 1696169
Density: 0.01512
Diameter: NA
Mean edge weigth: 156.26
Max. degree: 1023

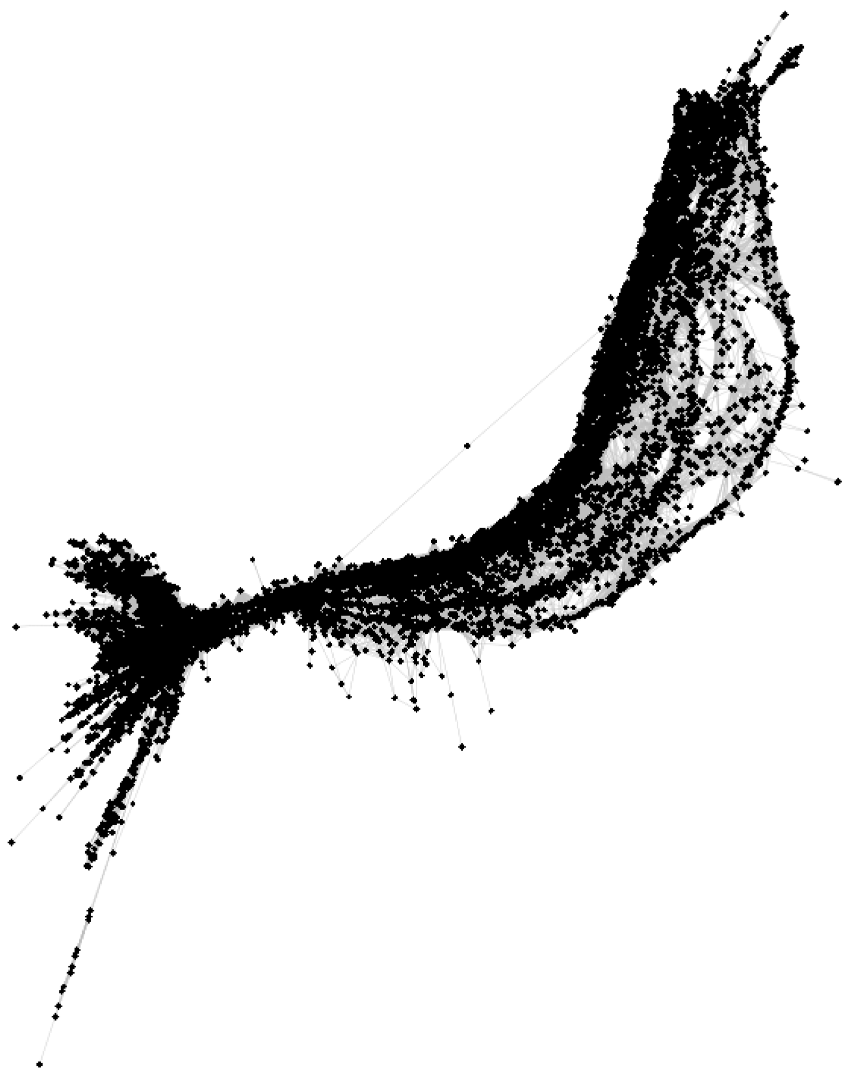

CL12

Number of reads: 14609
Number of pairs: 1465923
Density: 0.01374
Diameter: NA
Mean edge weigth: 157.17
Max. degree: 830

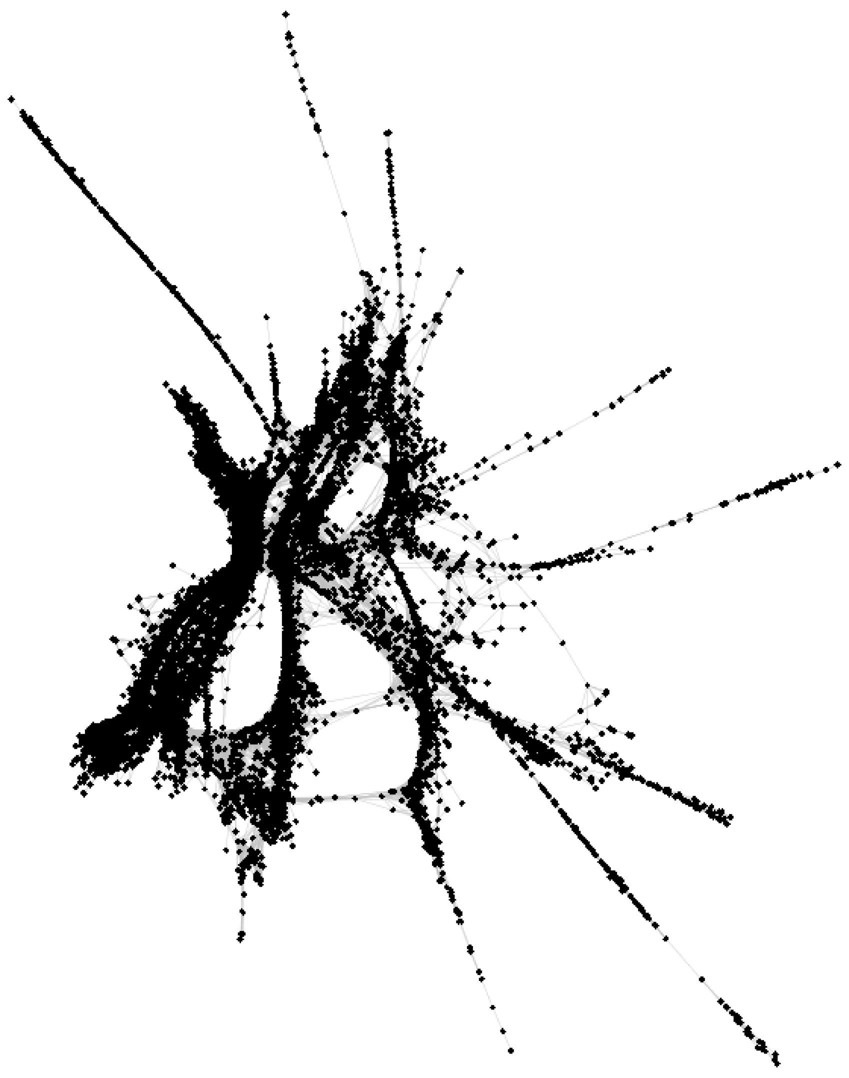

CL13

Number of reads: 13883
Number of pairs: 664510
Density: 0.006896
Diameter: NA
Mean edge weigth: 149.59
Max. degree: 684

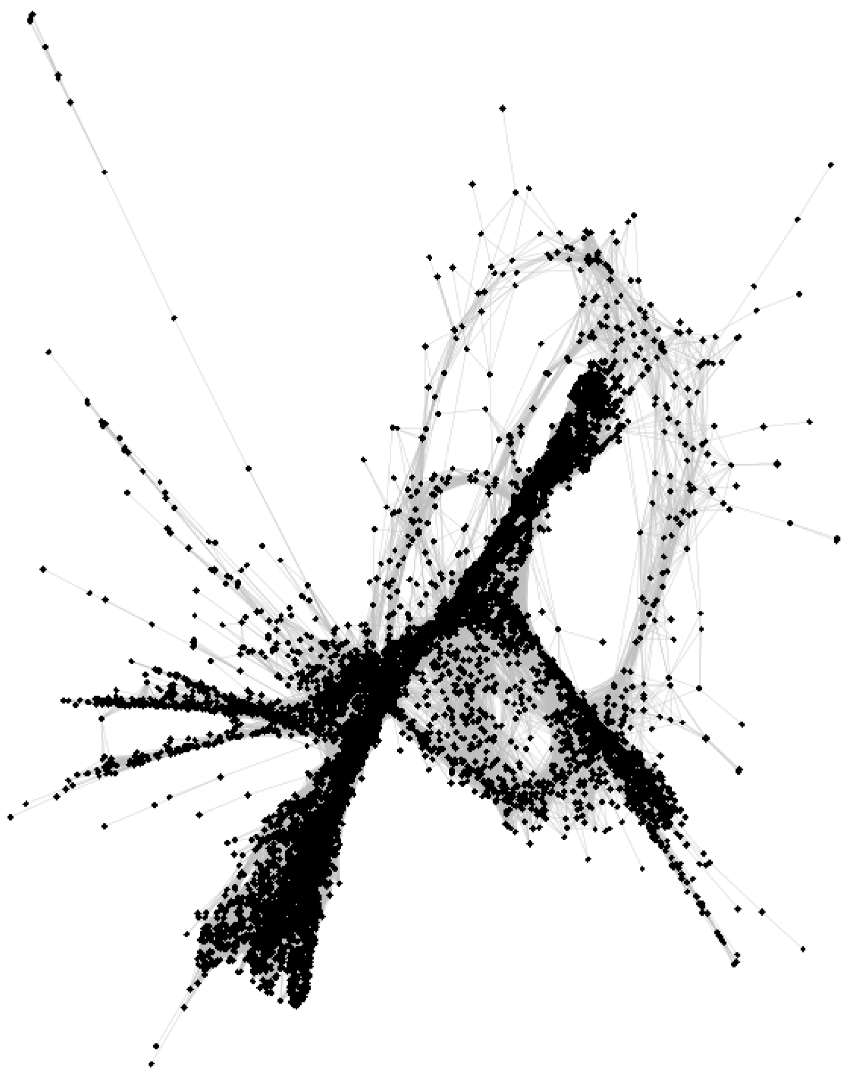

CL14

Number of reads: 13337
Number of pairs: 2885807
Density: 0.03245
Diameter: NA
Mean edge weigth: 159.1
Max. degree: 1309

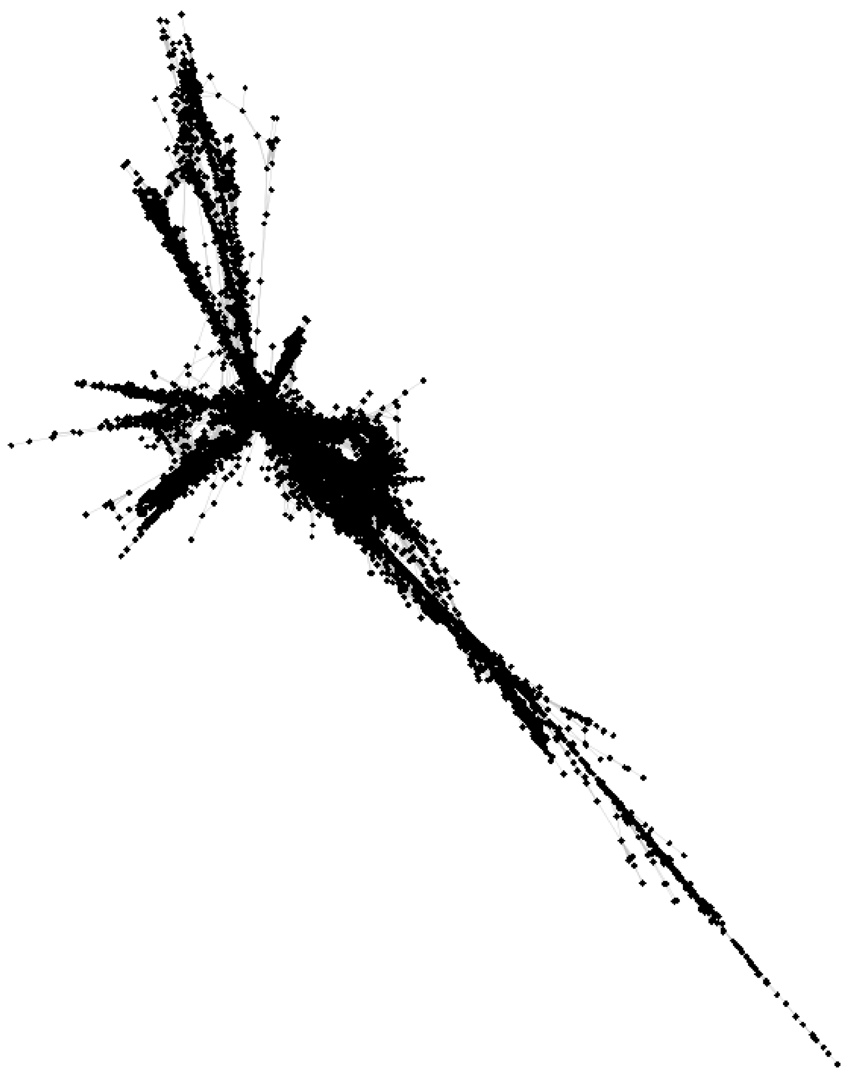

CL15

Number of reads: 13075
Number of pairs: 684315
Density: 0.008006
Diameter: NA
Mean edge weigth: 154.08
Max. degree: 924

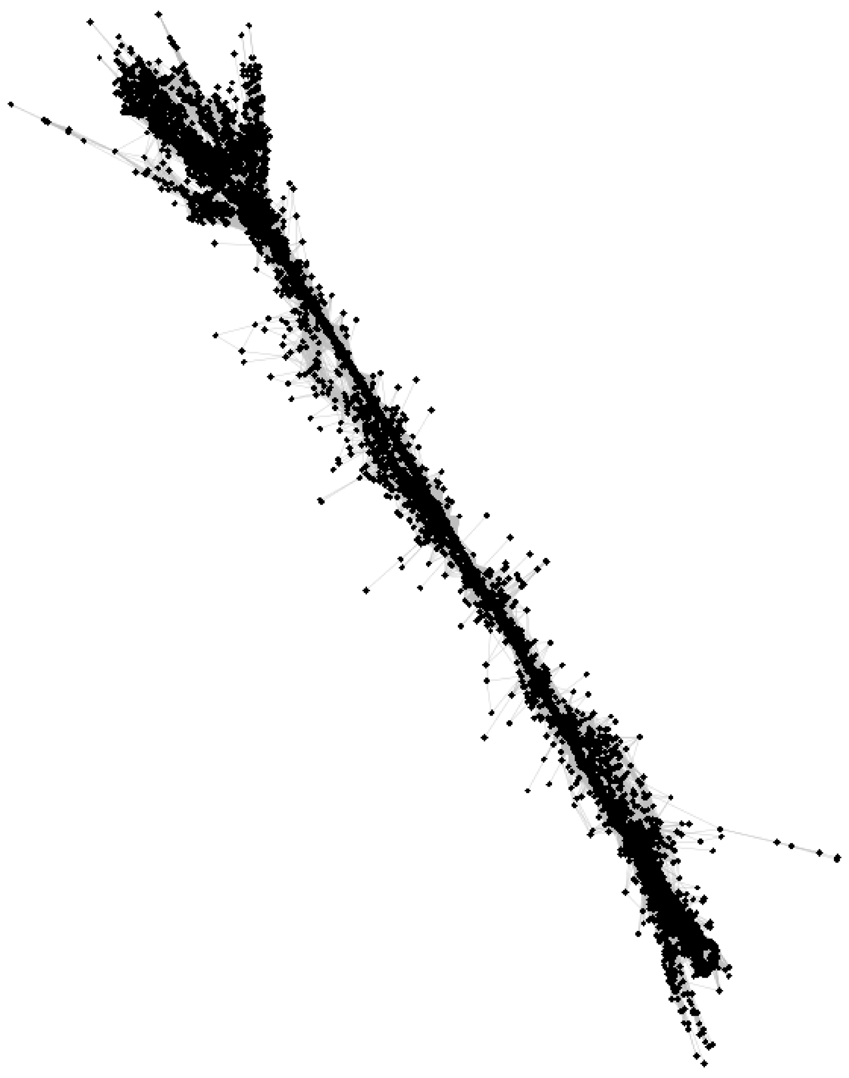

CL16

Number of reads: 13027
Number of pairs: 3656488
Density: 0.0431
Diameter: NA
Mean edge weigth: 166.66
Max. degree: 997

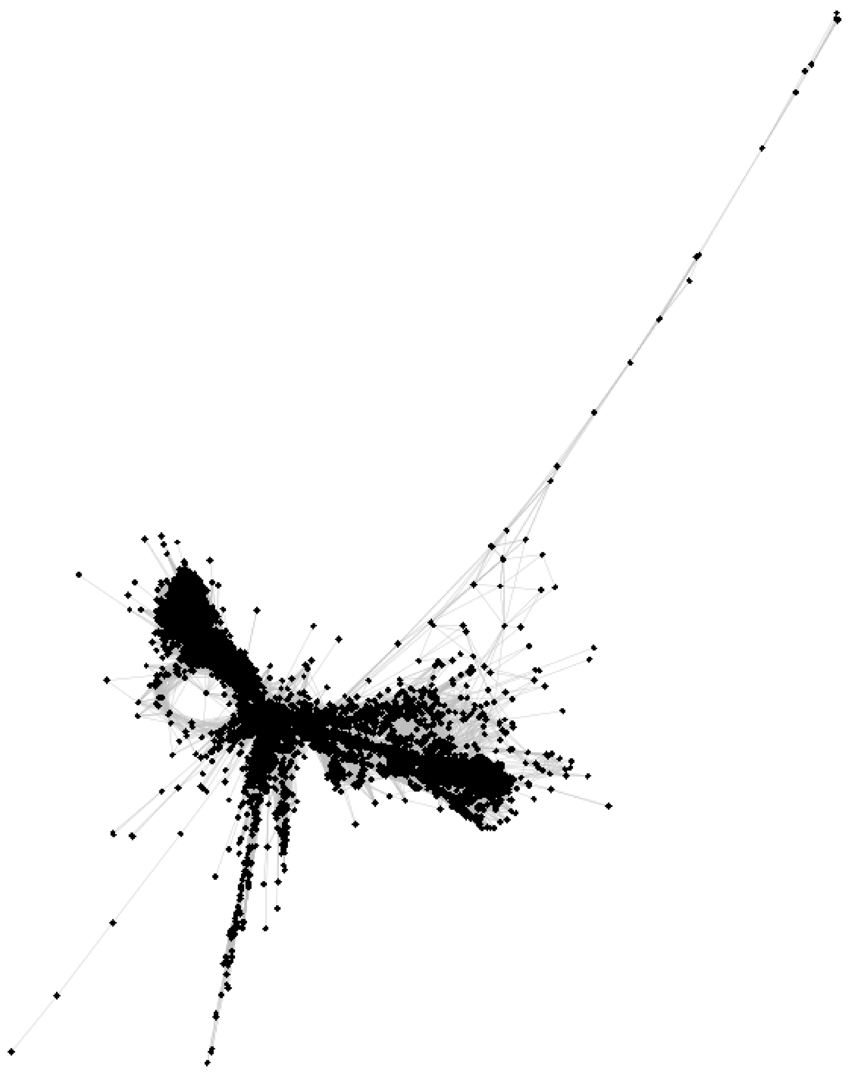

CL17

Number of reads: 12920
Number of pairs: 6162919
Density: 0.07385
Diameter: NA
Mean edge weigth: 160.09
Max. degree: 2590

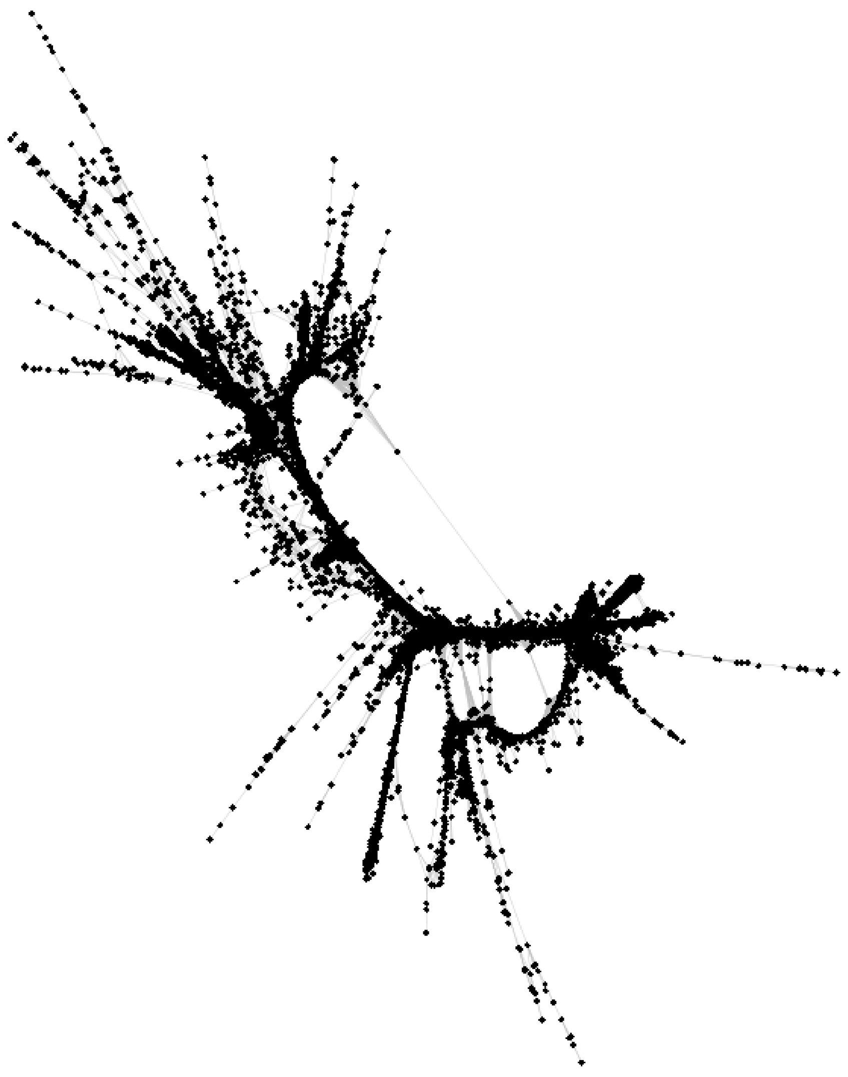

CL18

Number of reads: 12557
Number of pairs: 1010630
Density: 0.01282
Diameter: NA
Mean edge weigth: 156.53
Max. degree: 828

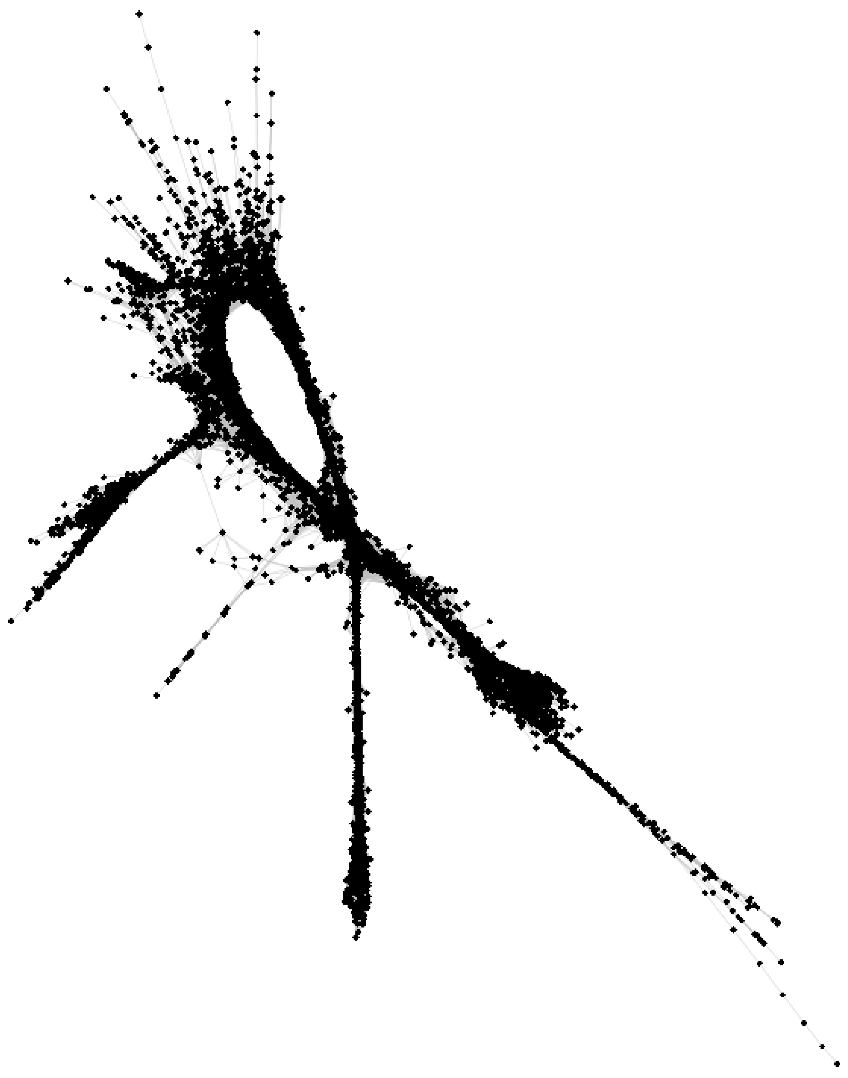

CL19

Number of reads: 12389
Number of pairs: 1164245
Density: 0.01517
Diameter: NA
Mean edge weigth: 159.14
Max. degree: 722

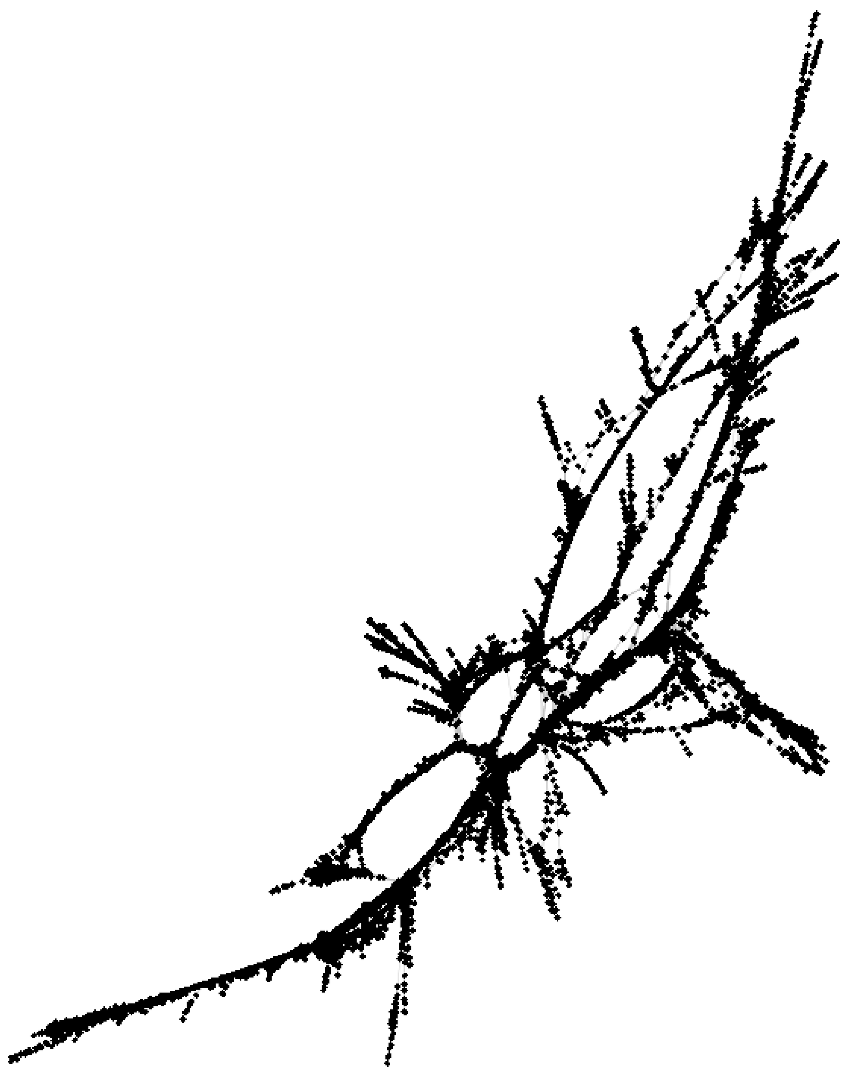

CL20

Number of reads: 12258
Number of pairs: 208028
Density: 0.002769
Diameter: NA
Mean edge weigth: 157.98
Max. degree: 216

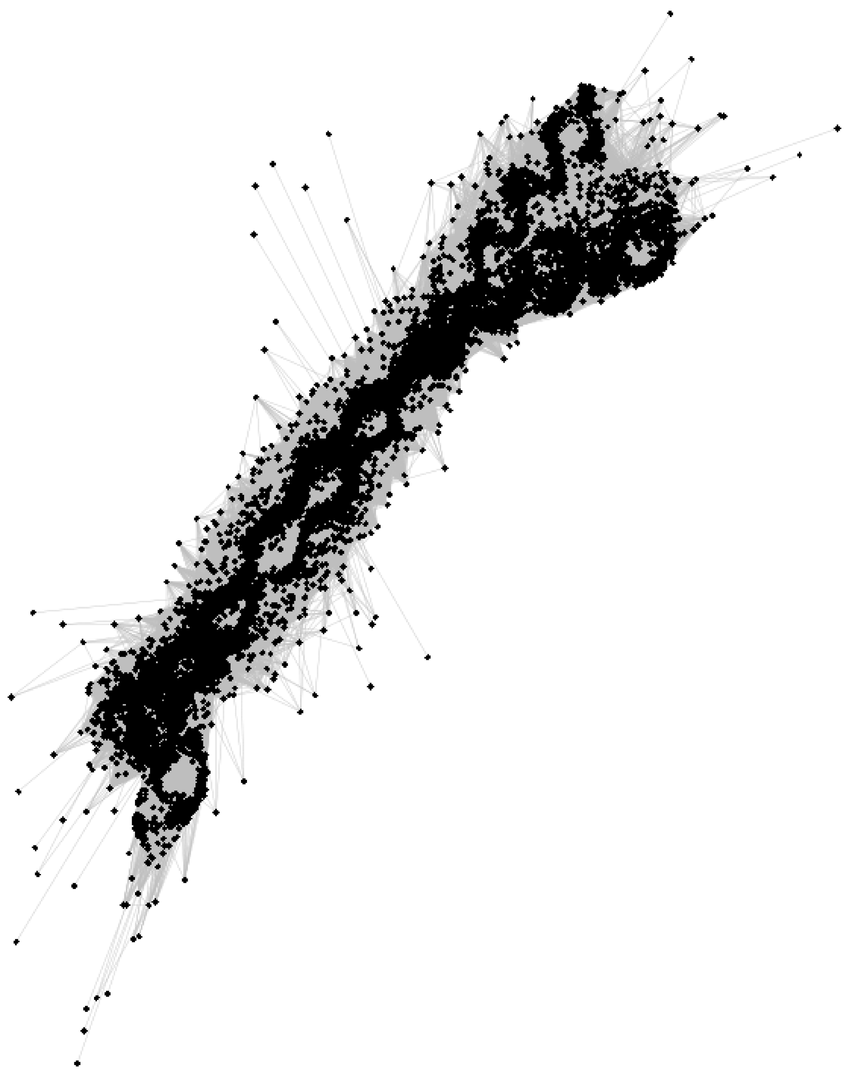

CL21

Number of reads: 12236
Number of pairs: 5608820
Density: 0.07493
Diameter: NA
Mean edge weigth: 166.68
Max. degree: 2353

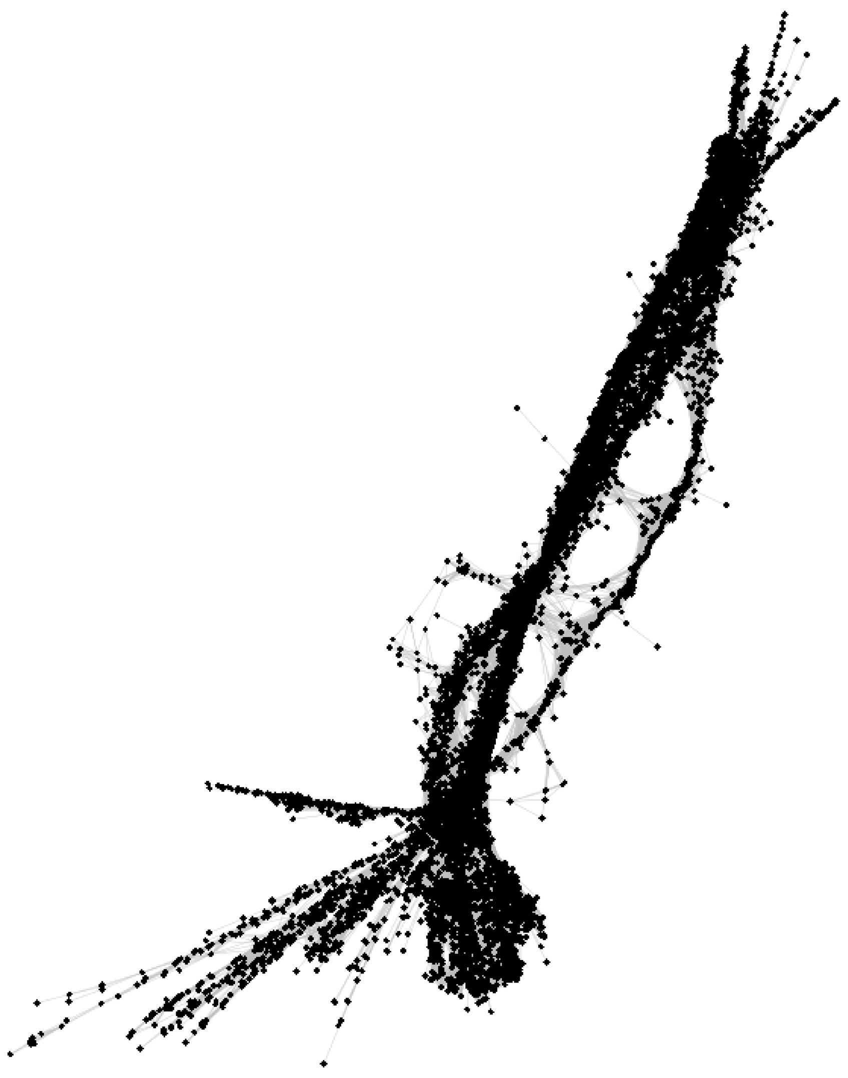

CL22

Number of reads: 12088
Number of pairs: 923764
Density: 0.01264
Diameter: NA
Mean edge weigth: 156.78
Max. degree: 523

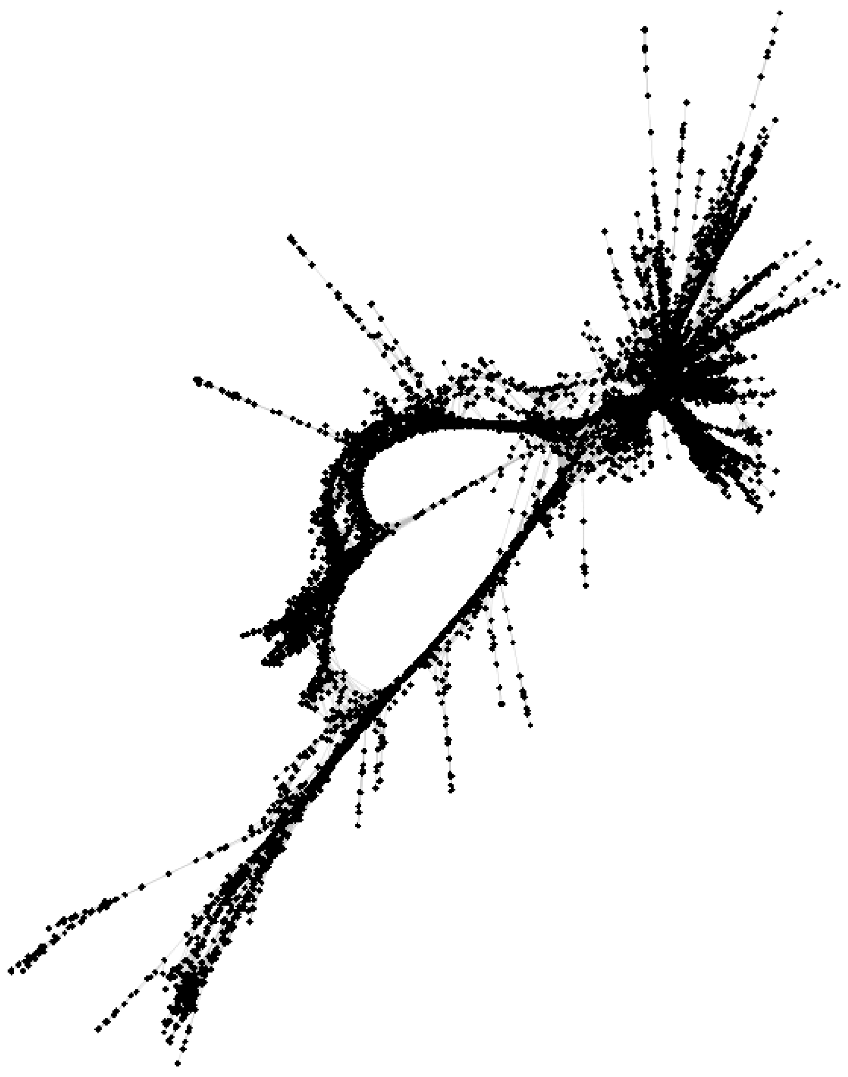

CL23

Number of reads: 11711
Number of pairs: 432370
Density: 0.006306
Diameter: NA
Mean edge weigth: 155.34
Max. degree: 612

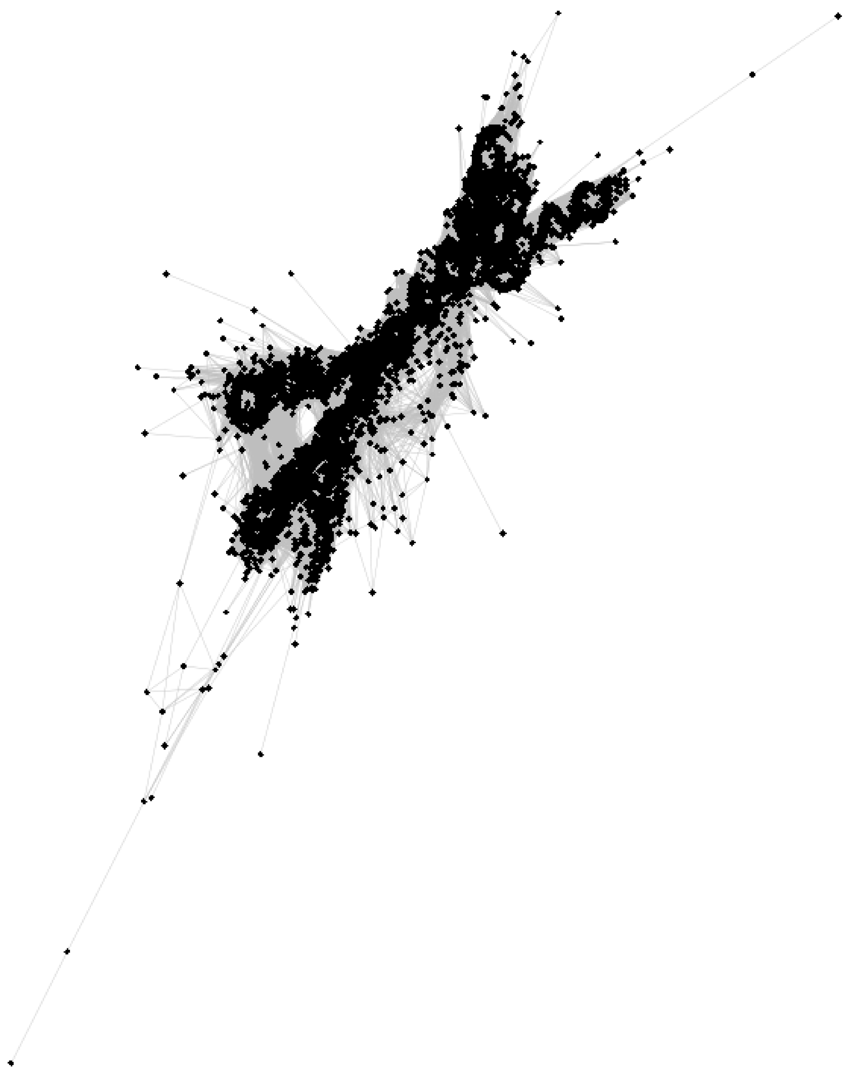

CL24

Number of reads: 11697
Number of pairs: 5585042
Density: 0.08165
Diameter: NA
Mean edge weigth: 166.82
Max. degree: 2256

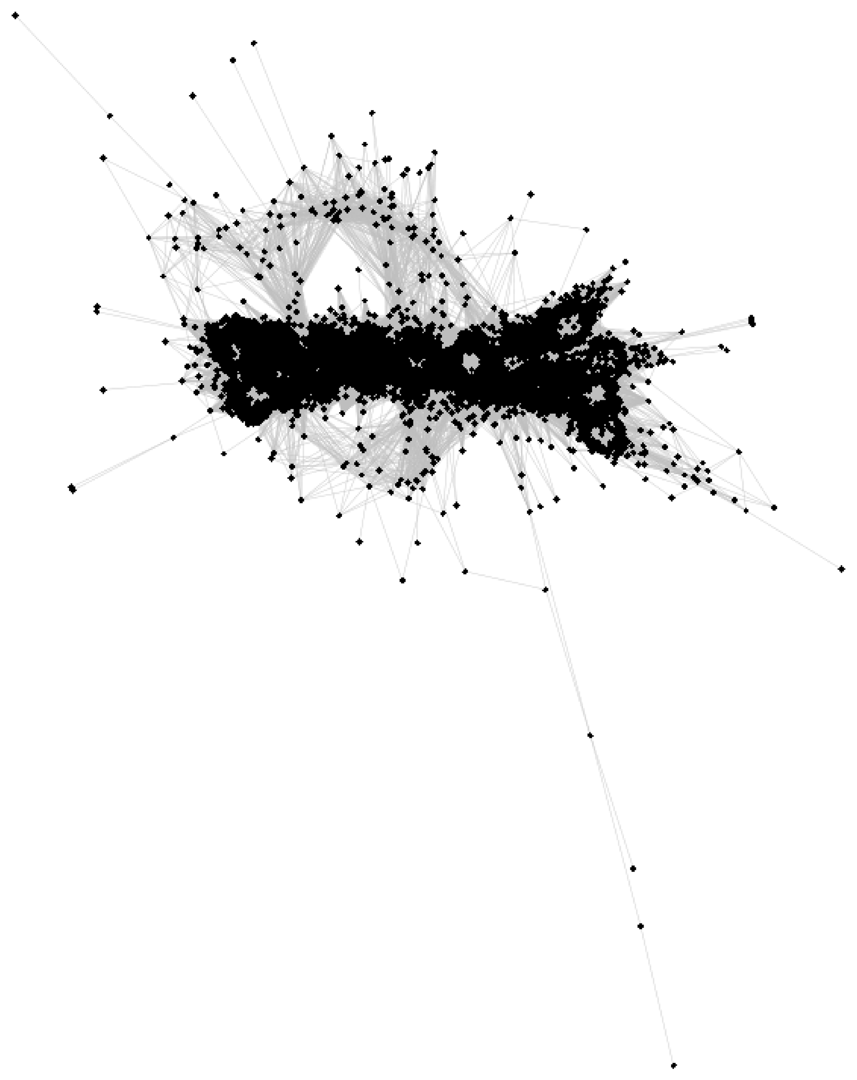

CL25

Number of reads: 11651
Number of pairs: 6109731
Density: 0.09002
Diameter: NA
Mean edge weigth: 159.04
Max. degree: 2430

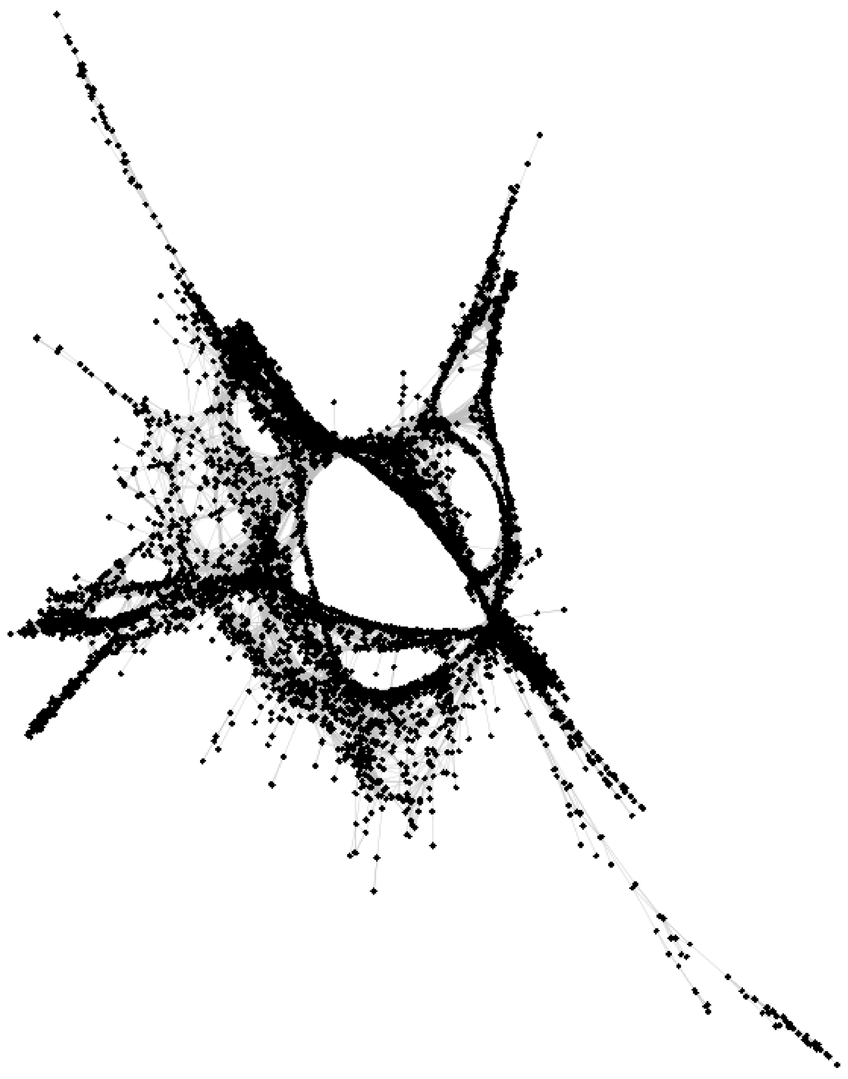

CL26

Number of reads: 11414
Number of pairs: 762795
Density: 0.01171
Diameter: NA
Mean edge weigth: 158.78
Max. degree: 638

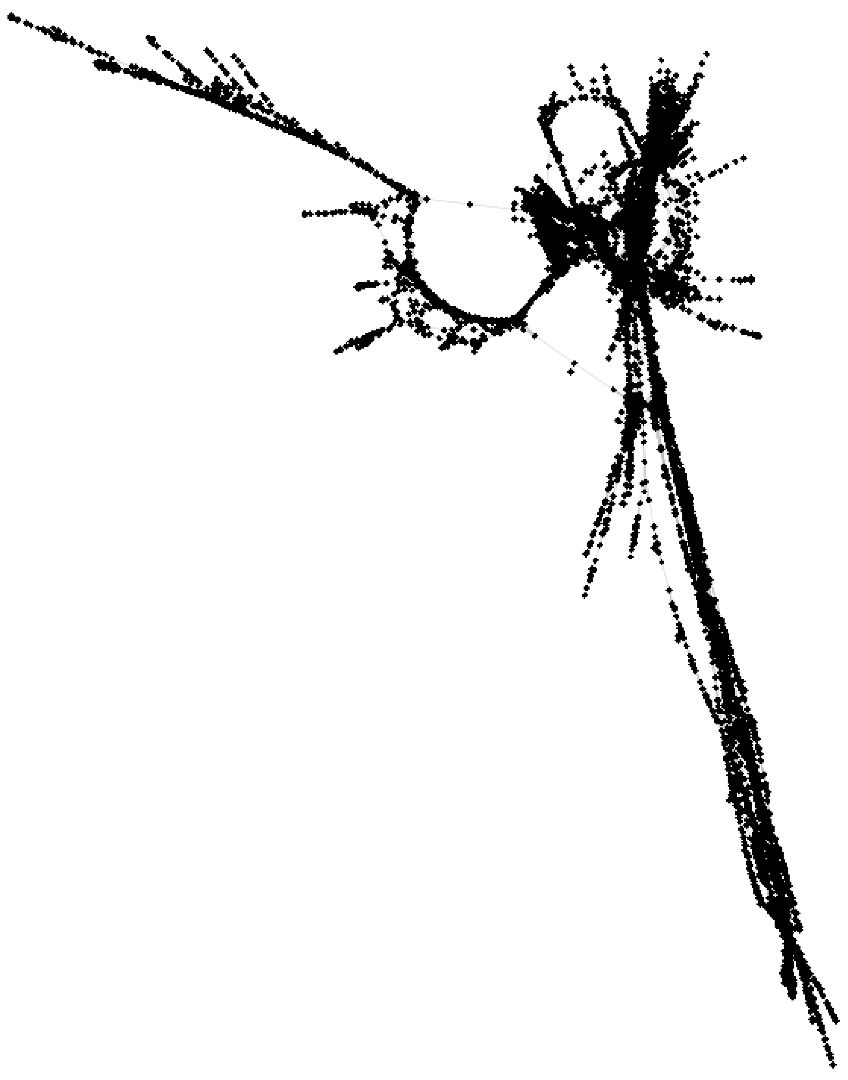

CL27

Number of reads: 11400
Number of pairs: 251020
Density: 0.003863
Diameter: NA
Mean edge weigth: 153.65
Max. degree: 403

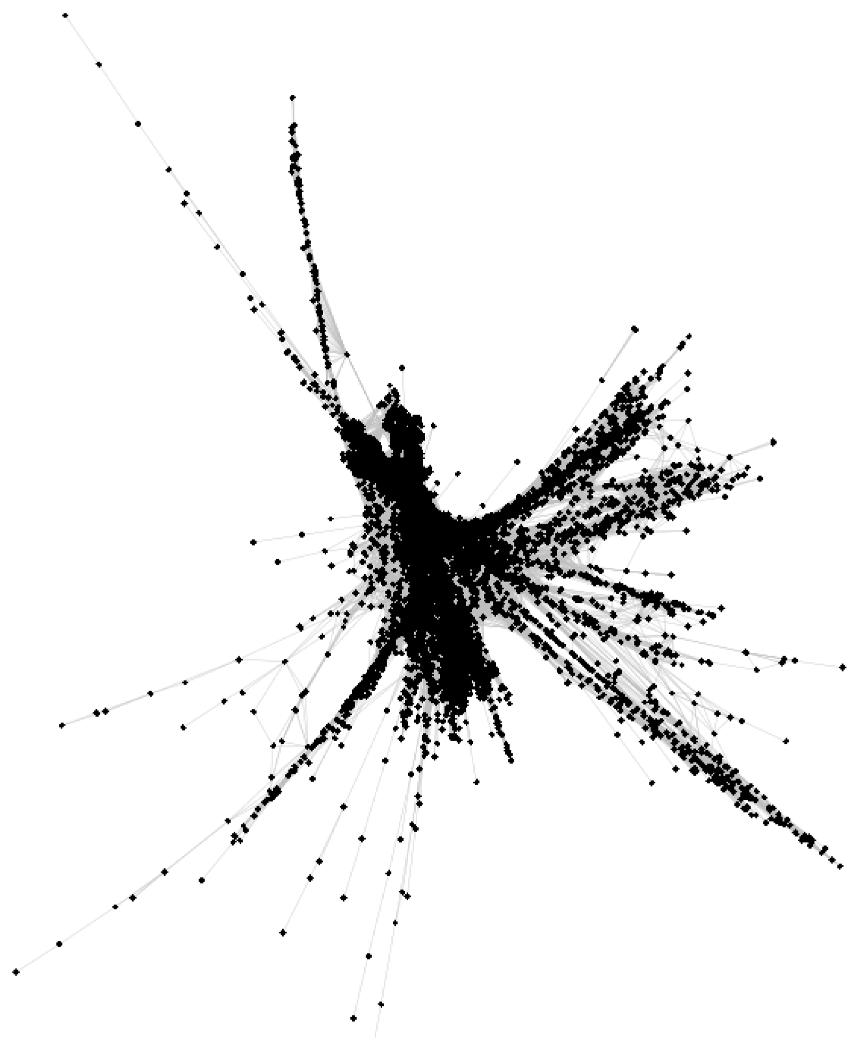

CL28

Number of reads: 11273
Number of pairs: 2572037
Density: 0.04048
Diameter: NA
Mean edge weigth: 153.48
Max. degree: 1575

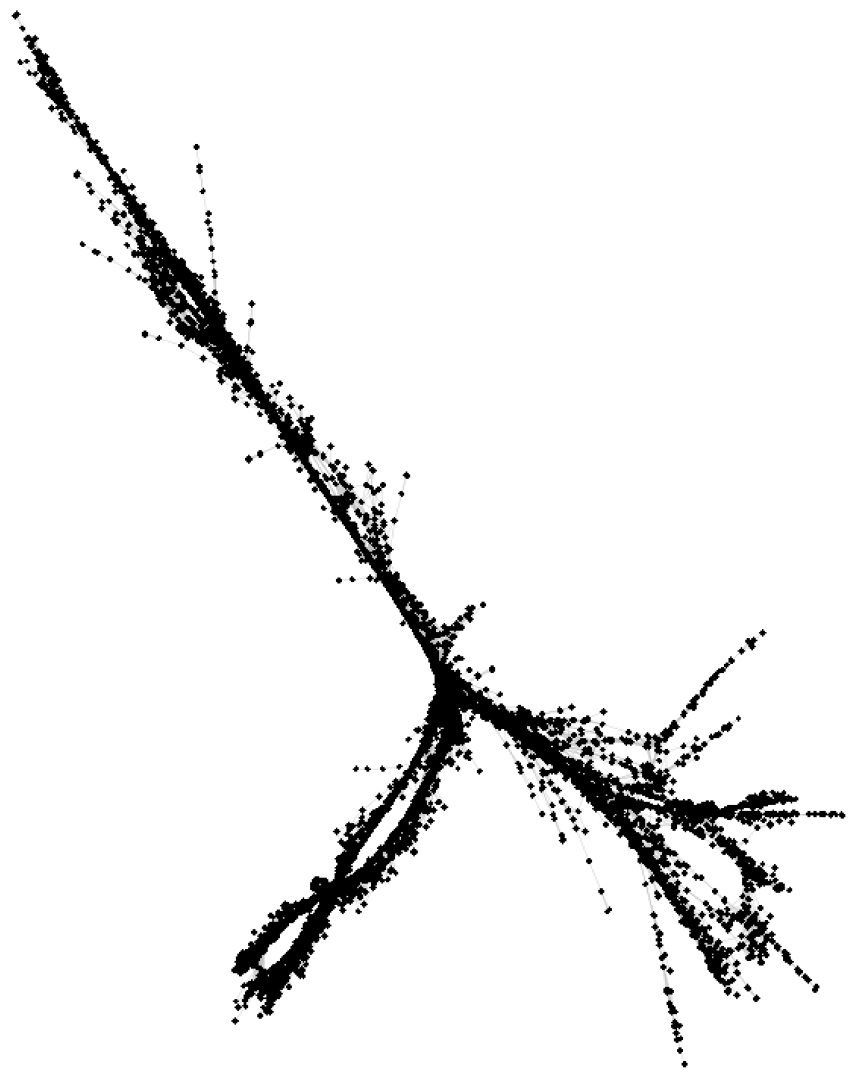

CL29

Number of reads: 11144
Number of pairs: 724522
Density: 0.01167
Diameter: NA
Mean edge weigth: 165.76
Max. degree: 483

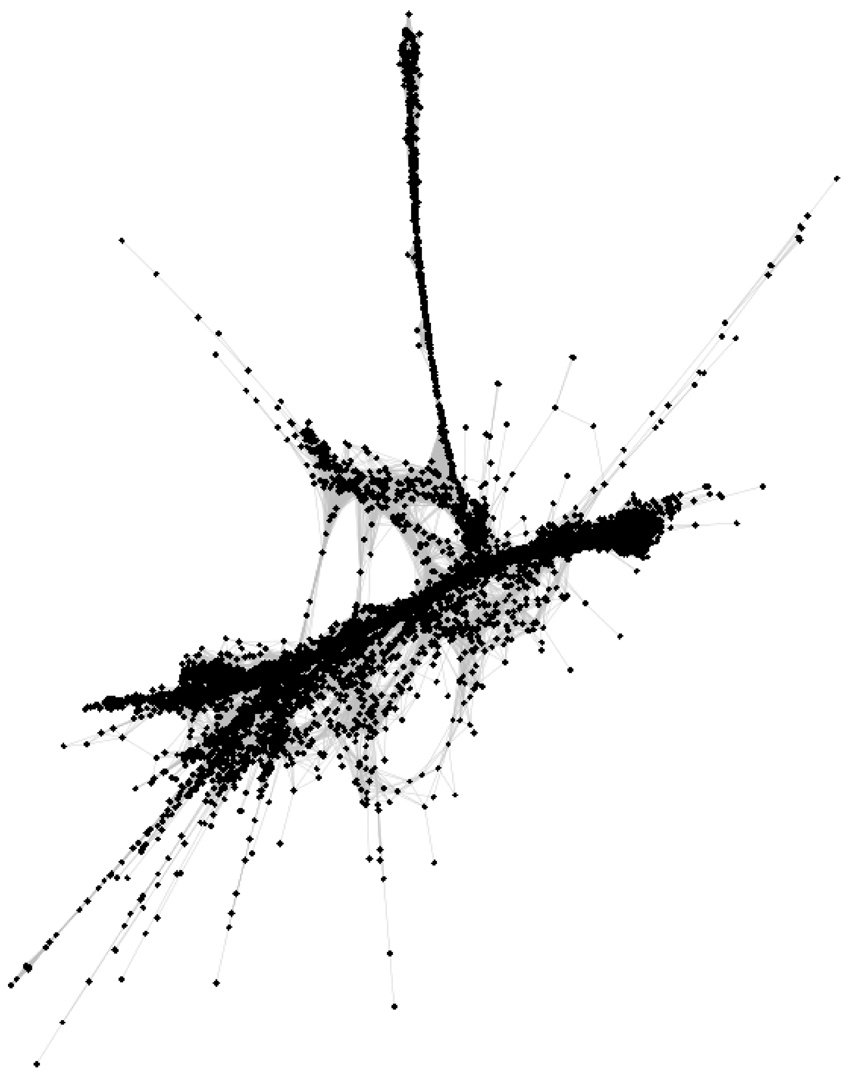

CL30

Number of reads: 11116
Number of pairs: 1991594
Density: 0.03224
Diameter: NA
Mean edge weigth: 158.54
Max. degree: 1013

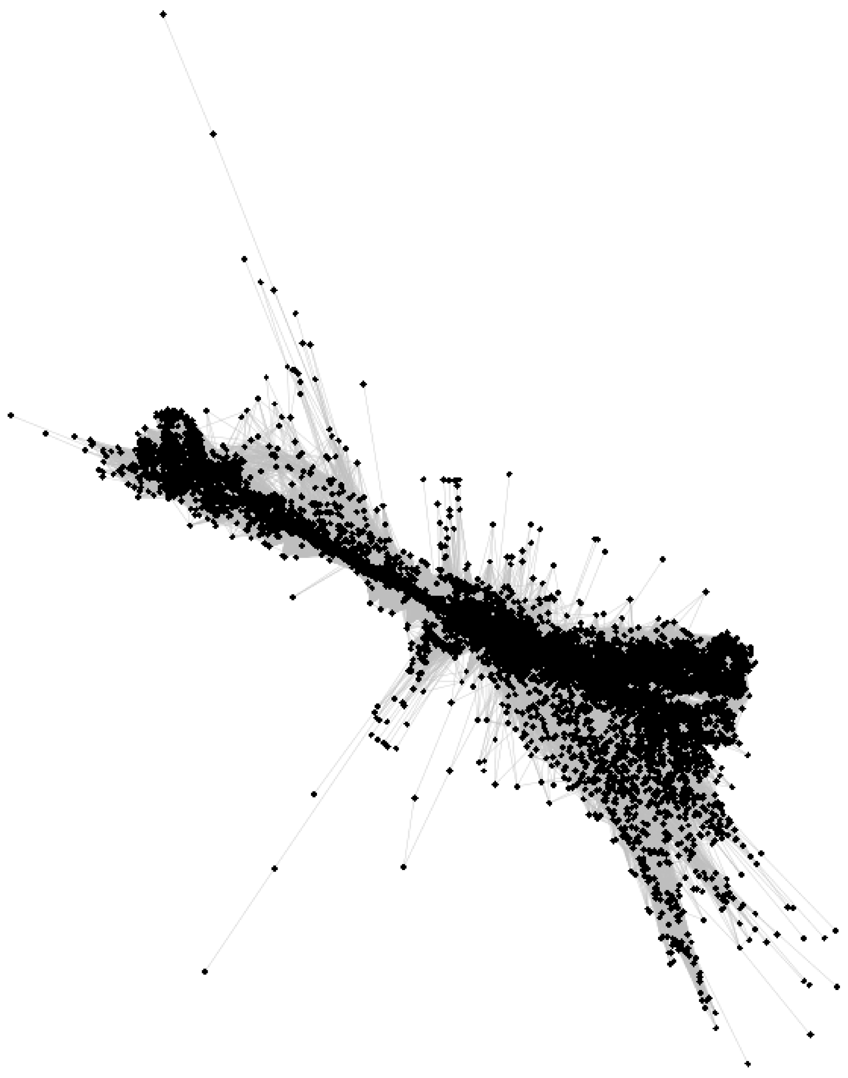

CL31

Number of reads: 10833
Number of pairs: 4071709
Density: 0.0694
Diameter: NA
Mean edge weigth: 159.32
Max. degree: 2015

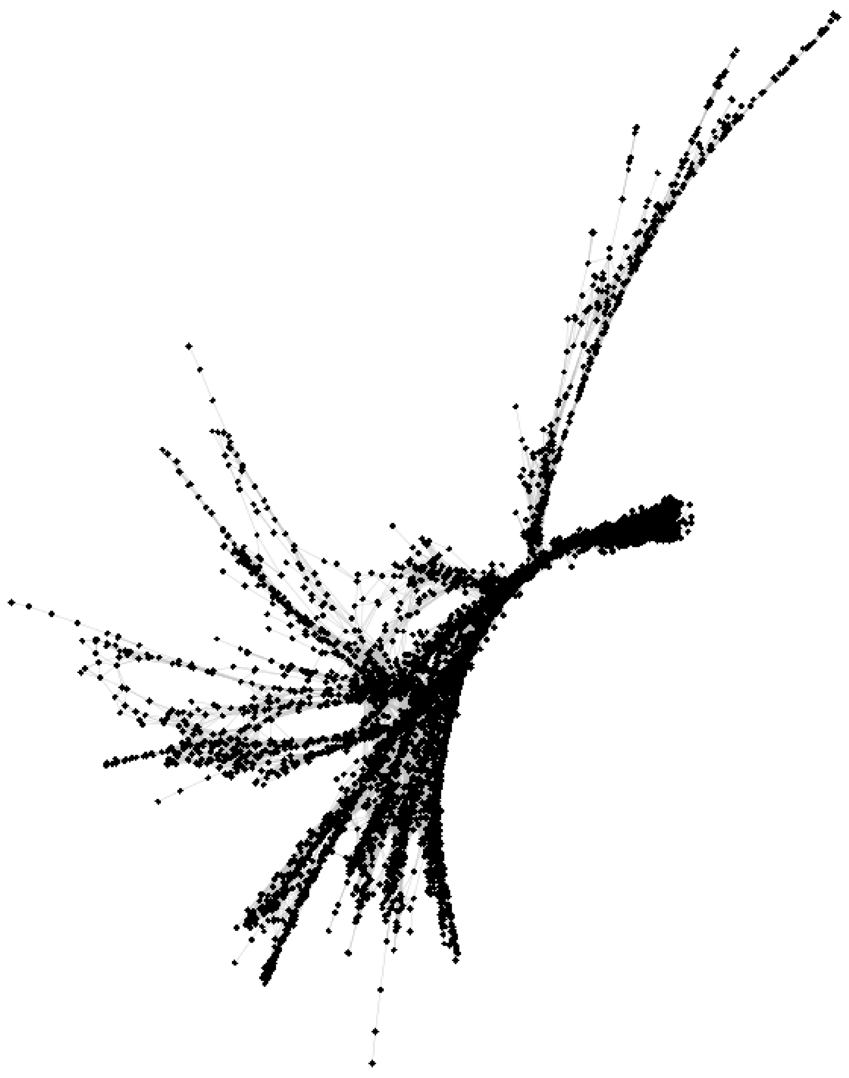

CL32

Number of reads: 10774
Number of pairs: 1739416
Density: 0.02997
Diameter: NA
Mean edge weigth: 166.14
Max. degree: 908

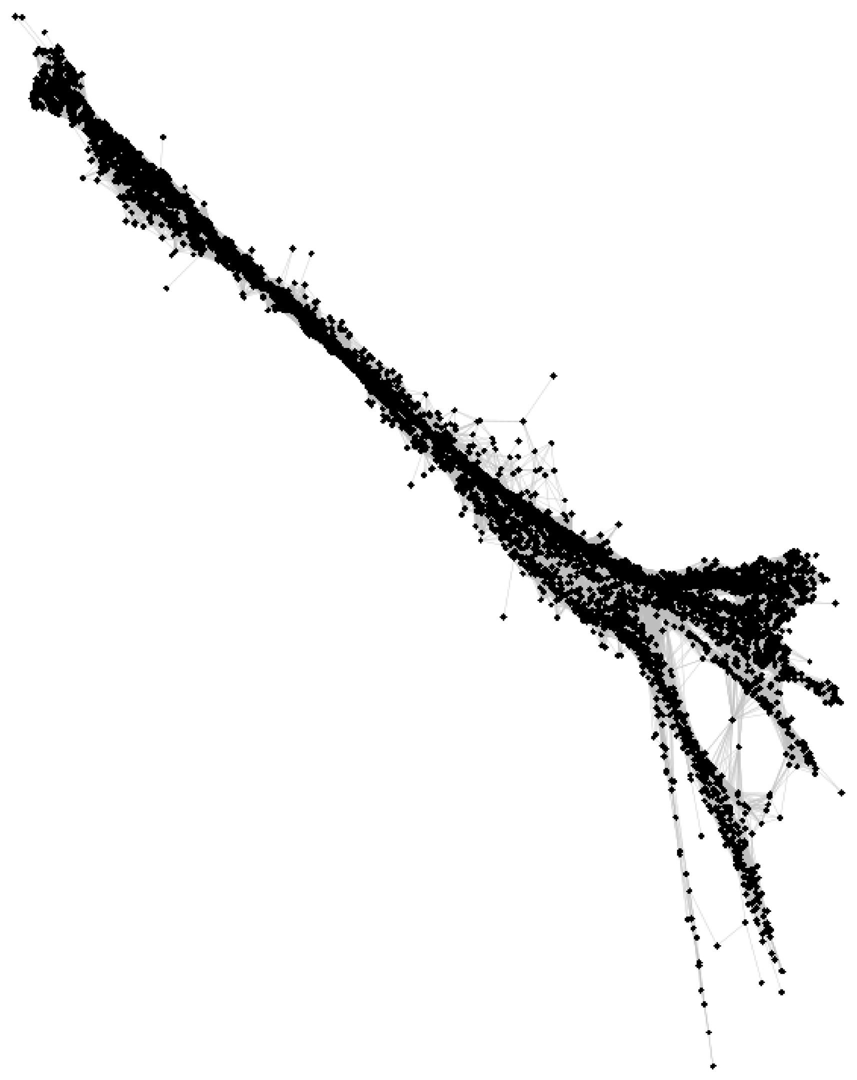

CL33

Number of reads: 10676
Number of pairs: 1934314
Density: 0.03395
Diameter: NA
Mean edge weigth: 168.1
Max. degree: 772

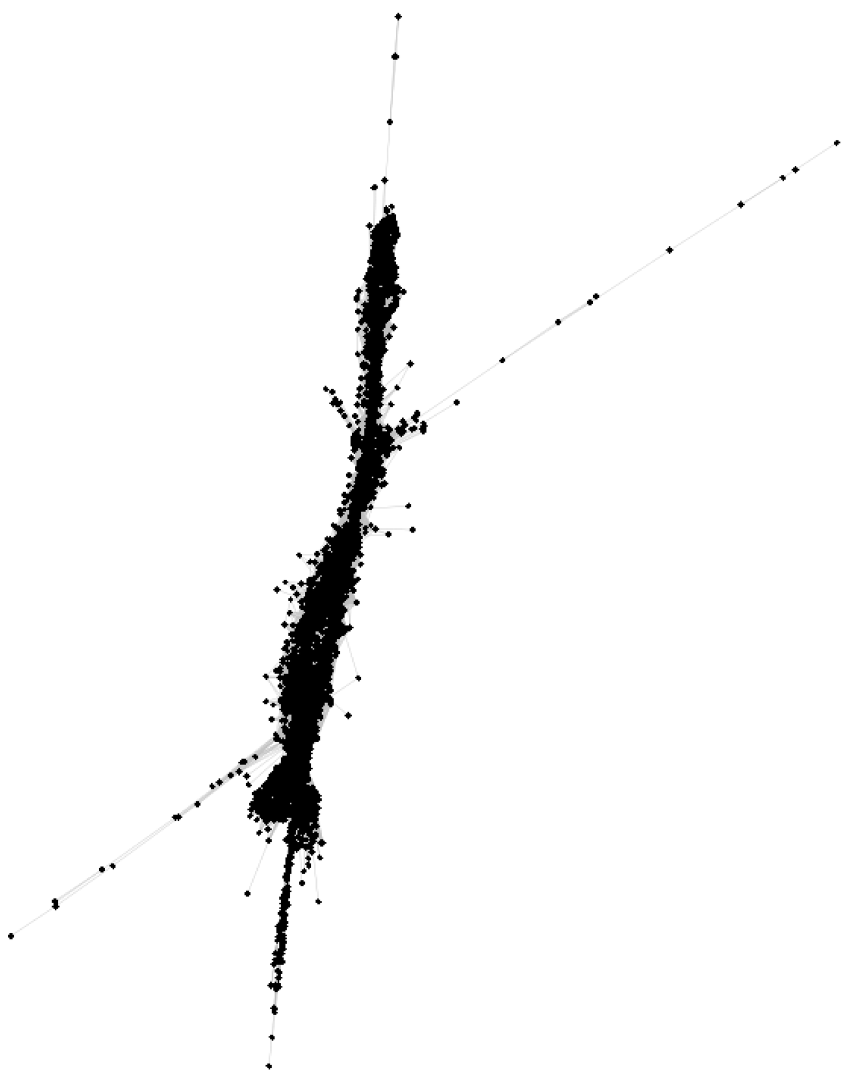

CL34

Number of reads: 10655
Number of pairs: 2646736
Density: 0.04663
Diameter: NA
Mean edge weigth: 157.36
Max. degree: 1414

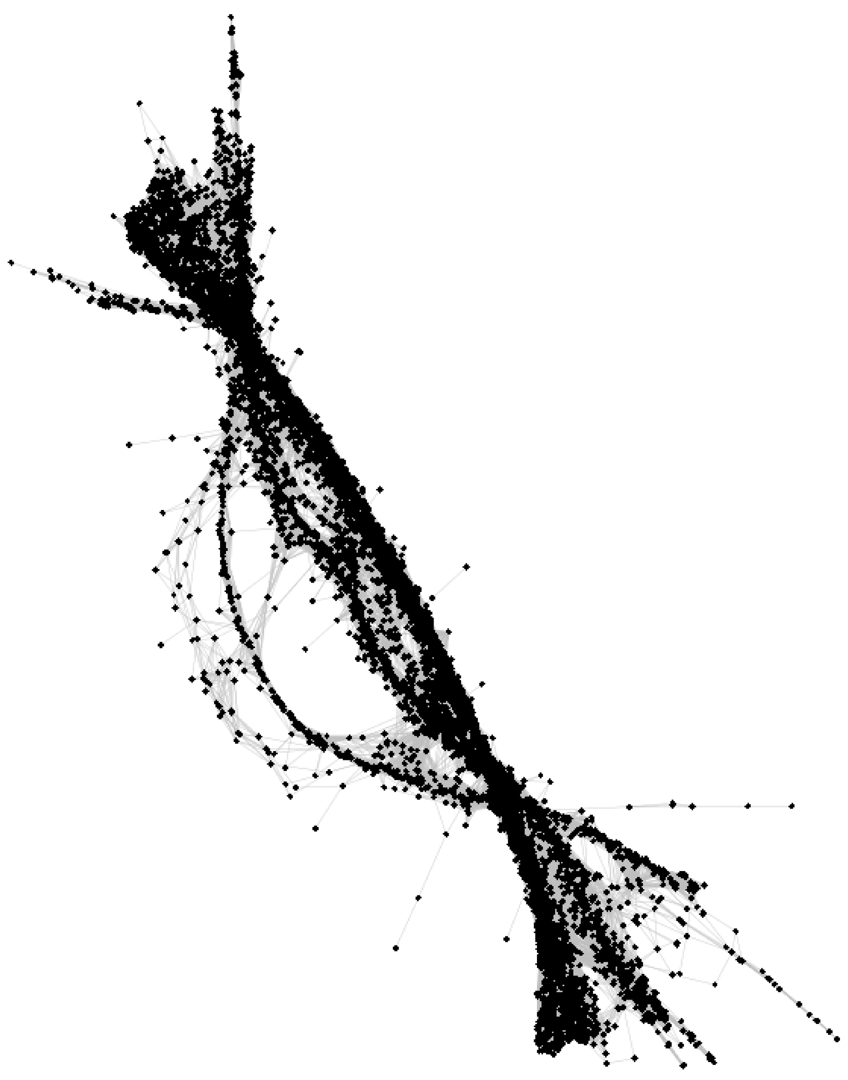

CL35

Number of reads: 10553
Number of pairs: 948746
Density: 0.01704
Diameter: NA
Mean edge weigth: 157.42
Max. degree: 504

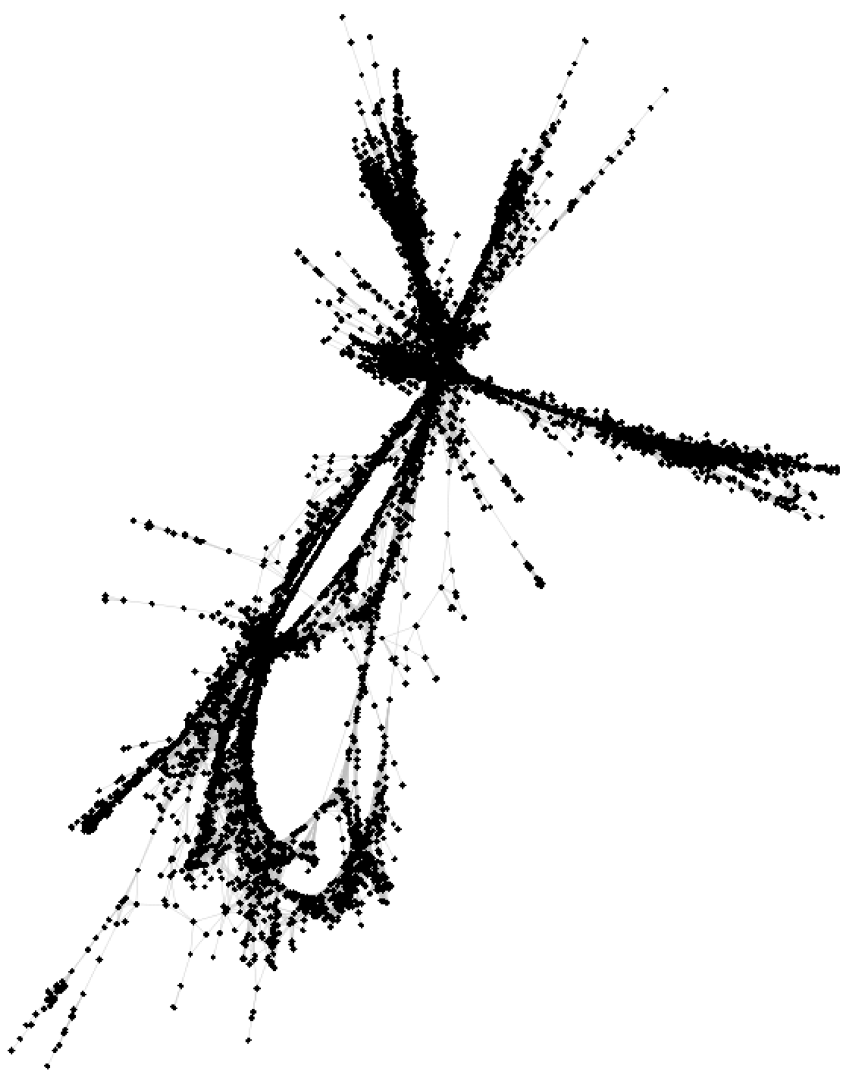

CL36

Number of reads: 10343
Number of pairs: 390573
Density: 0.007303
Diameter: NA
Mean edge weigth: 159.63
Max. degree: 391

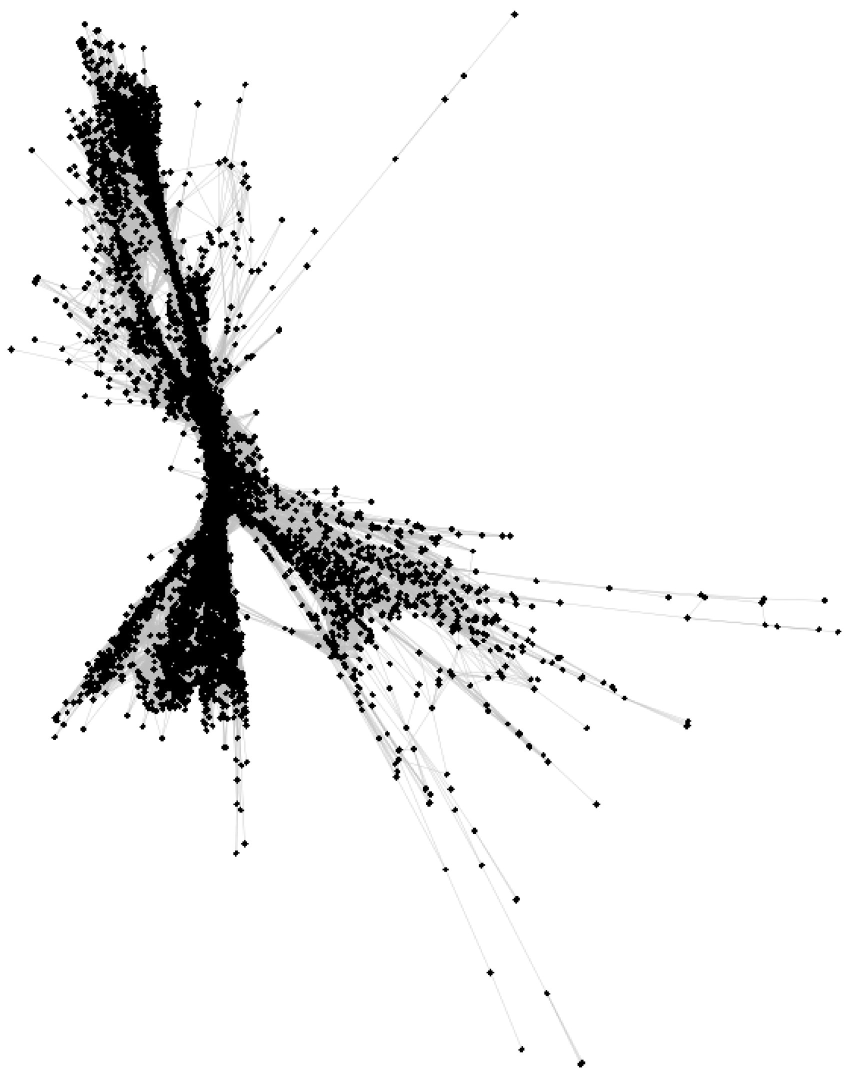

CL37

Number of reads: 10178
Number of pairs: 2998725
Density: 0.0579
Diameter: NA
Mean edge weigth: 165.98
Max. degree: 1805

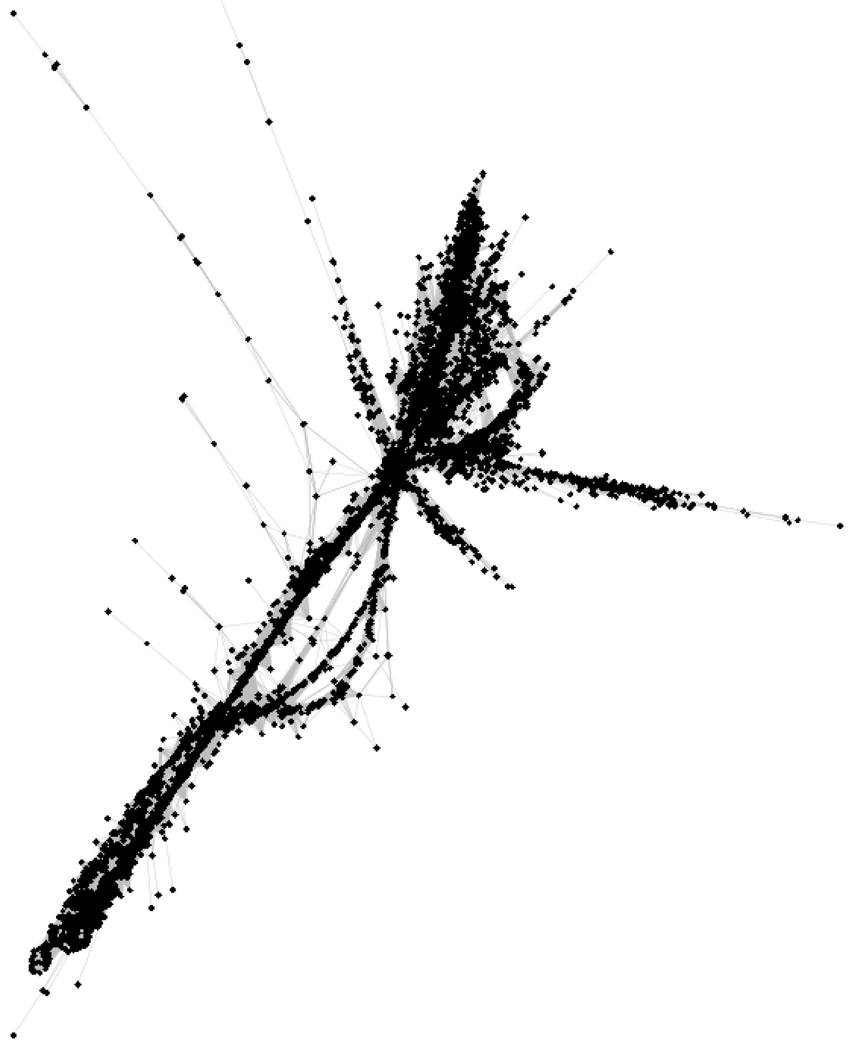

CL38

Number of reads: 10096
Number of pairs: 1843501
Density: 0.03618
Diameter: NA
Mean edge weigth: 165.25
Max. degree: 779

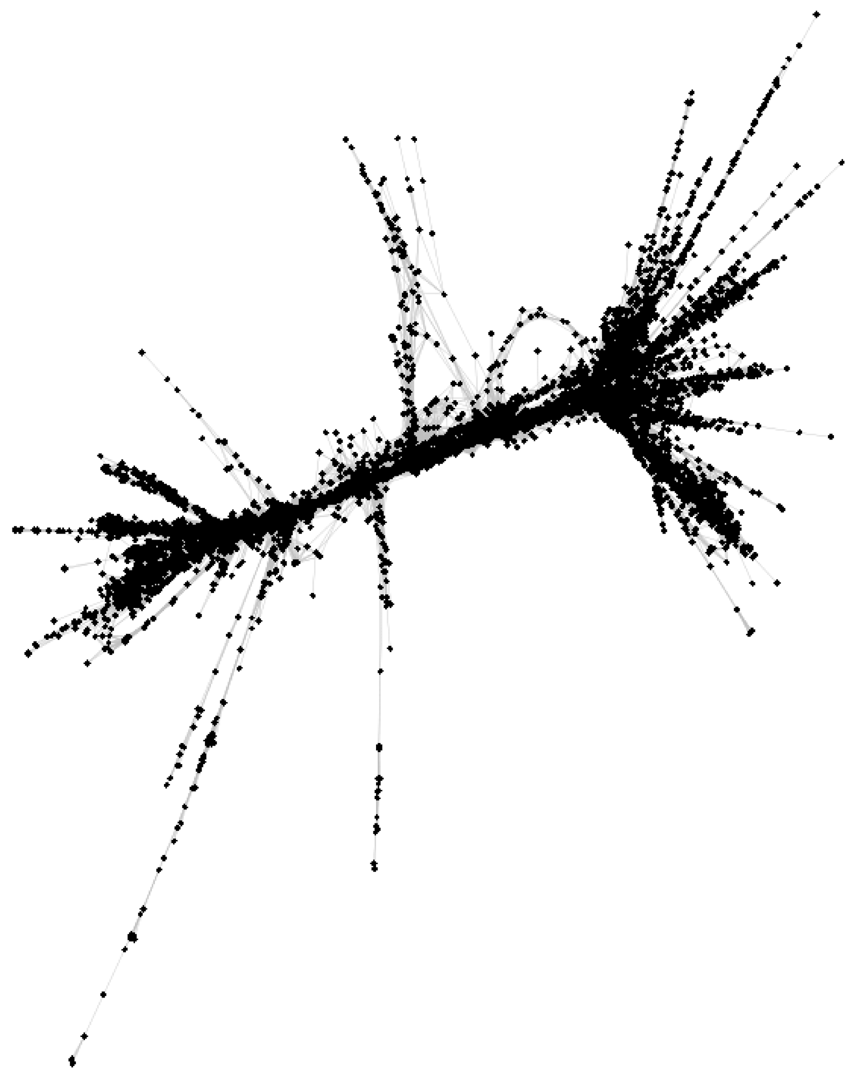

CL39

Number of reads: 9907
Number of pairs: 1119156
Density: 0.02281
Diameter: NA
Mean edge weigth: 156.56
Max. degree: 789

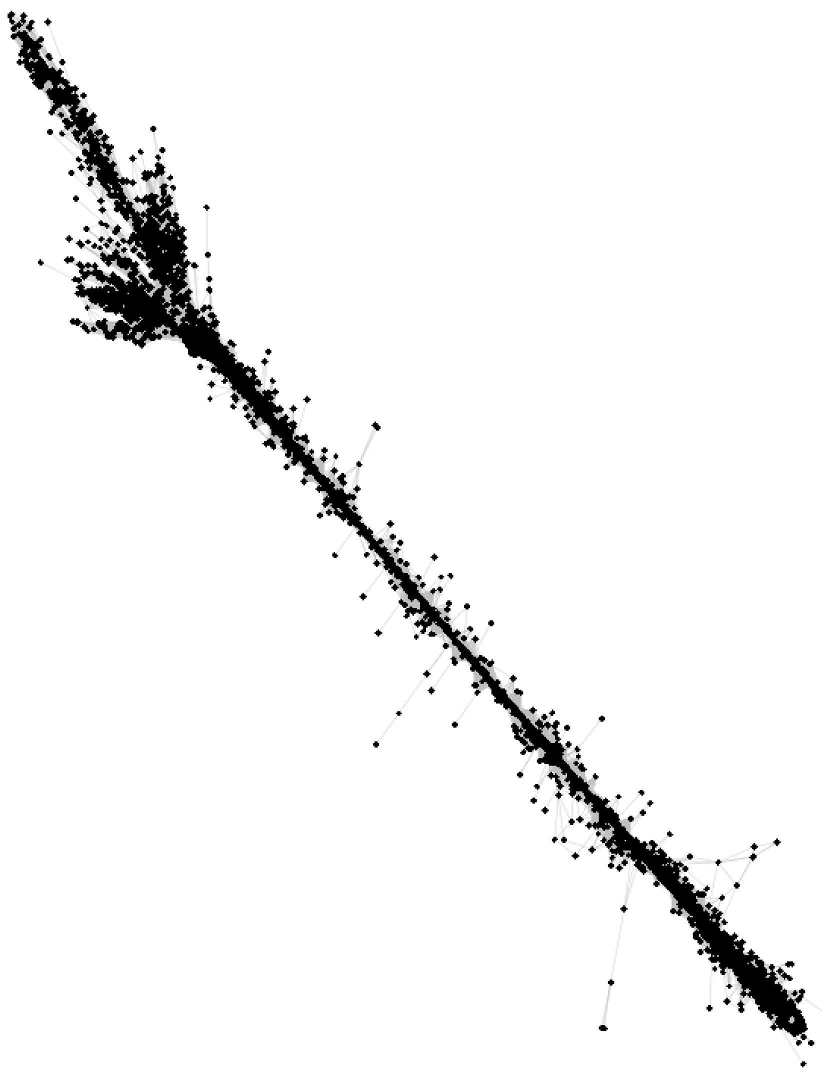

CL40

Number of reads: 9897
Number of pairs: 1843570
Density: 0.03765
Diameter: NA
Mean edge weigth: 167.77
Max. degree: 630

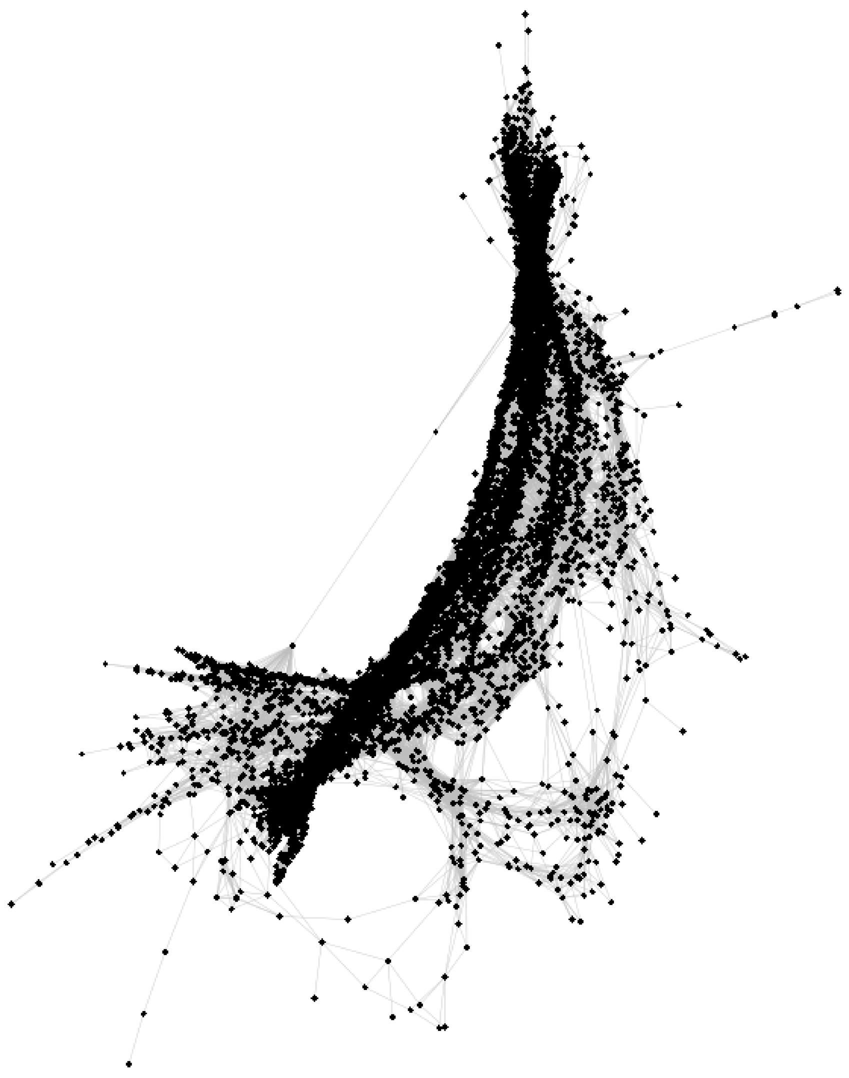

CL41

Number of reads: 9816
Number of pairs: 844615
Density: 0.01753
Diameter: NA
Mean edge weigth: 162.62
Max. degree: 564

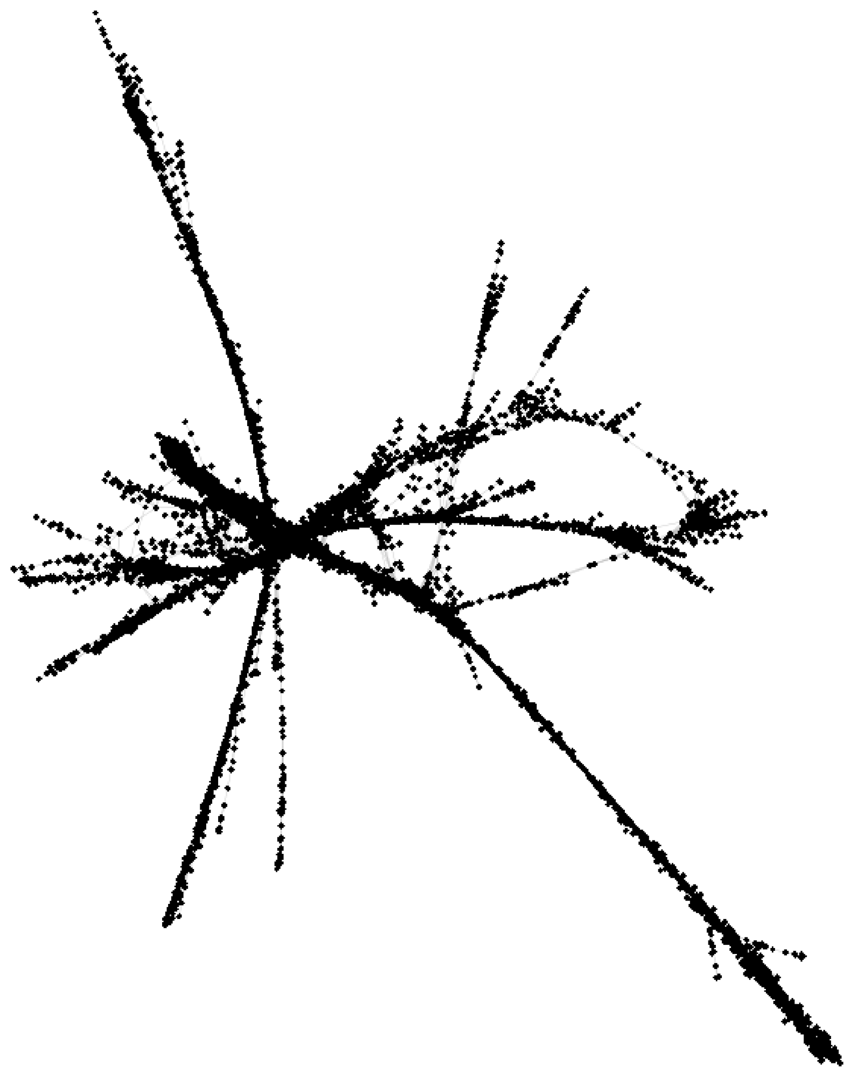

CL42

Number of reads: 9710
Number of pairs: 204335
Density: 0.004335
Diameter: NA
Mean edge weigth: 157.09
Max. degree: 214

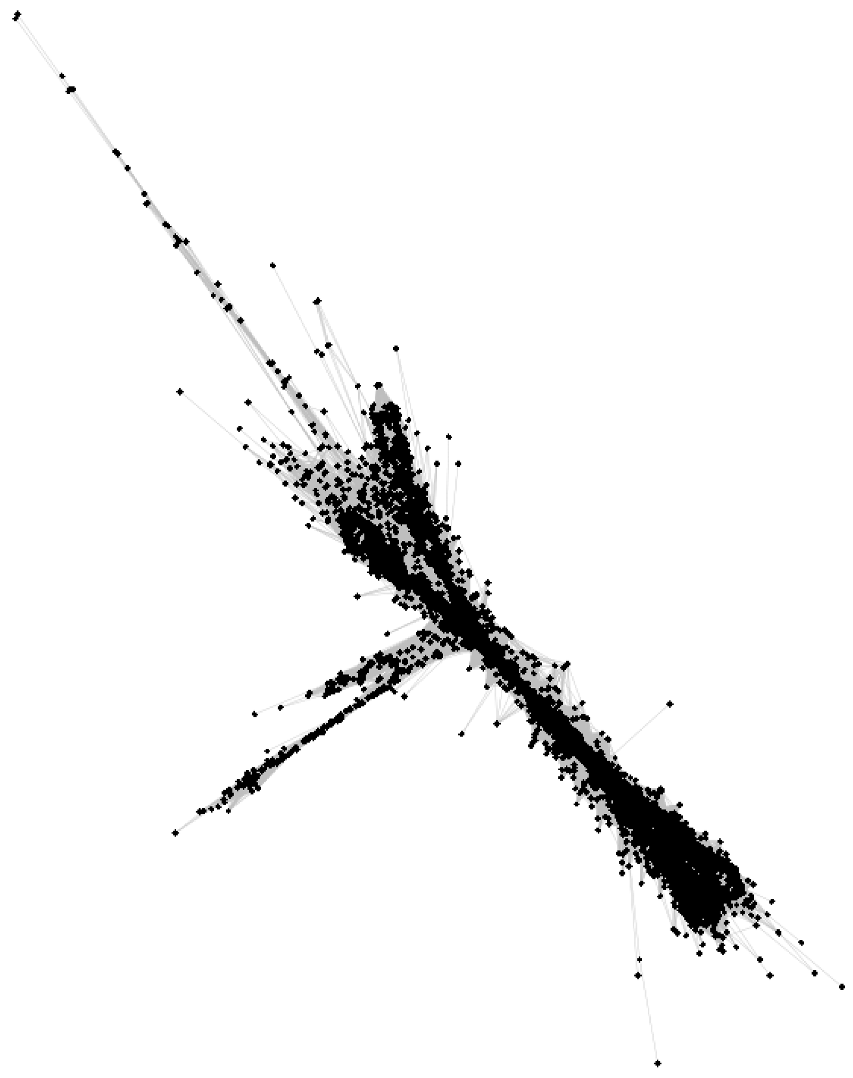

CL43

Number of reads: 9527
Number of pairs: 3824824
Density: 0.08429
Diameter: NA
Mean edge weigth: 164.84
Max. degree: 1479

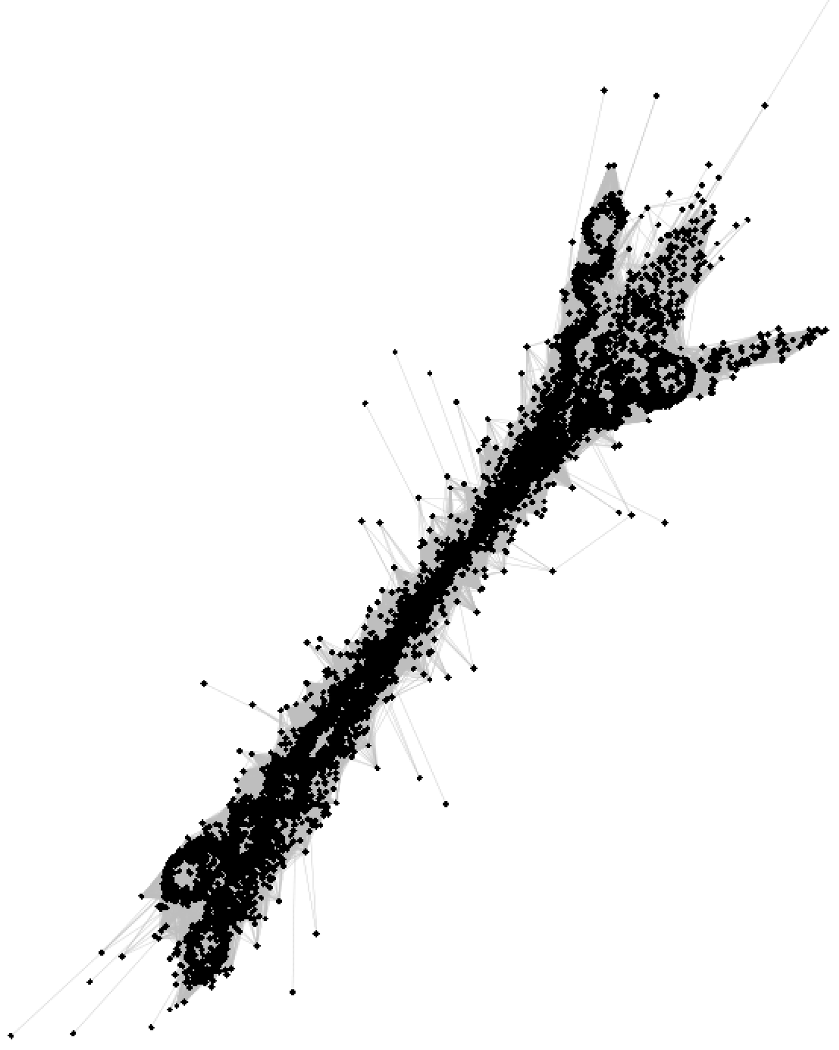

CL44

Number of reads: 9482
Number of pairs: 3887334
Density: 0.08648
Diameter: NA
Mean edge weigth: 164.22
Max. degree: 1511

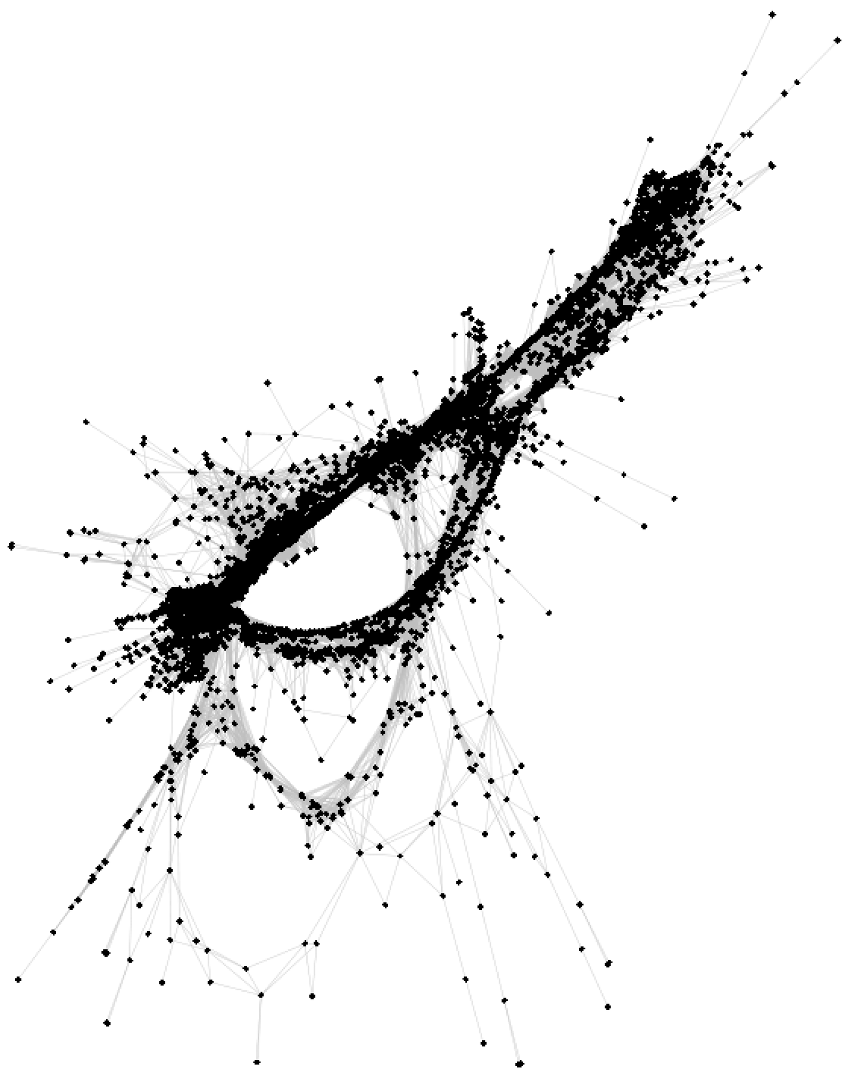

CL45

Number of reads: 9348
Number of pairs: 1665060
Density: 0.03811
Diameter: NA
Mean edge weigth: 159.83
Max. degree: 1310

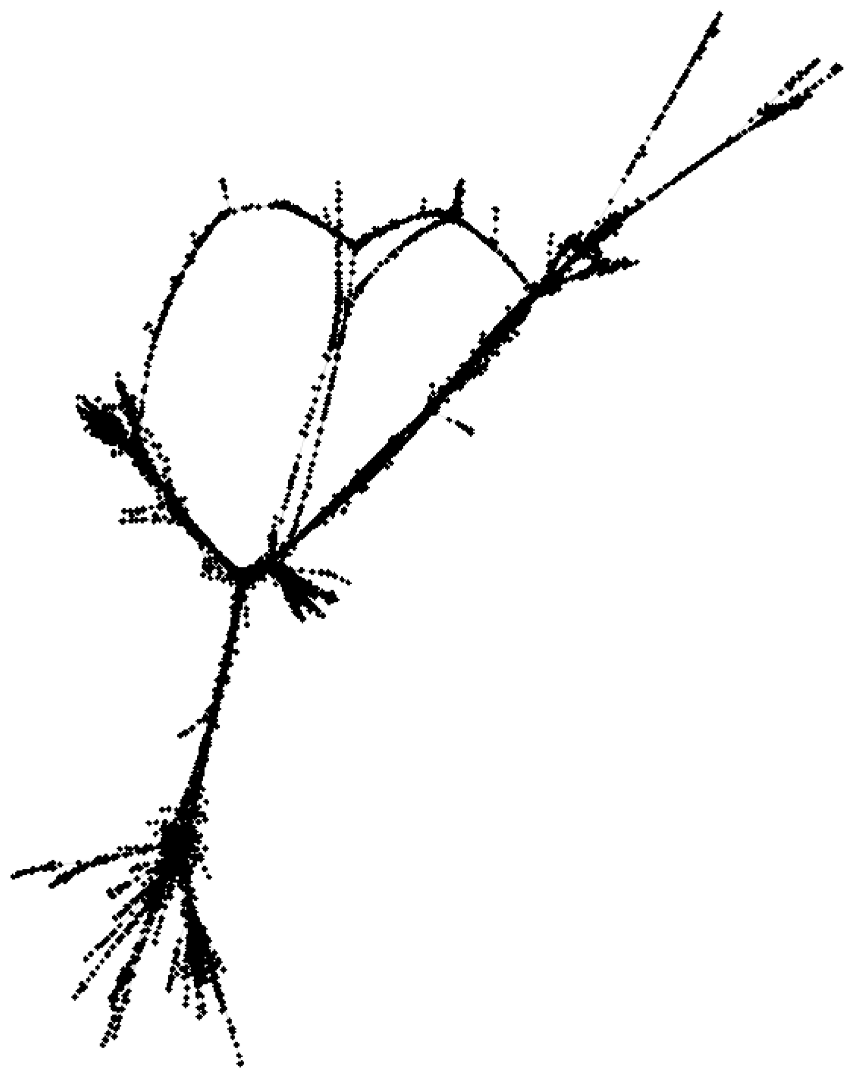

CL46

Number of reads: 9289
Number of pairs: 256012
Density: 0.005935
Diameter: NA
Mean edge weigth: 164.67
Max. degree: 284

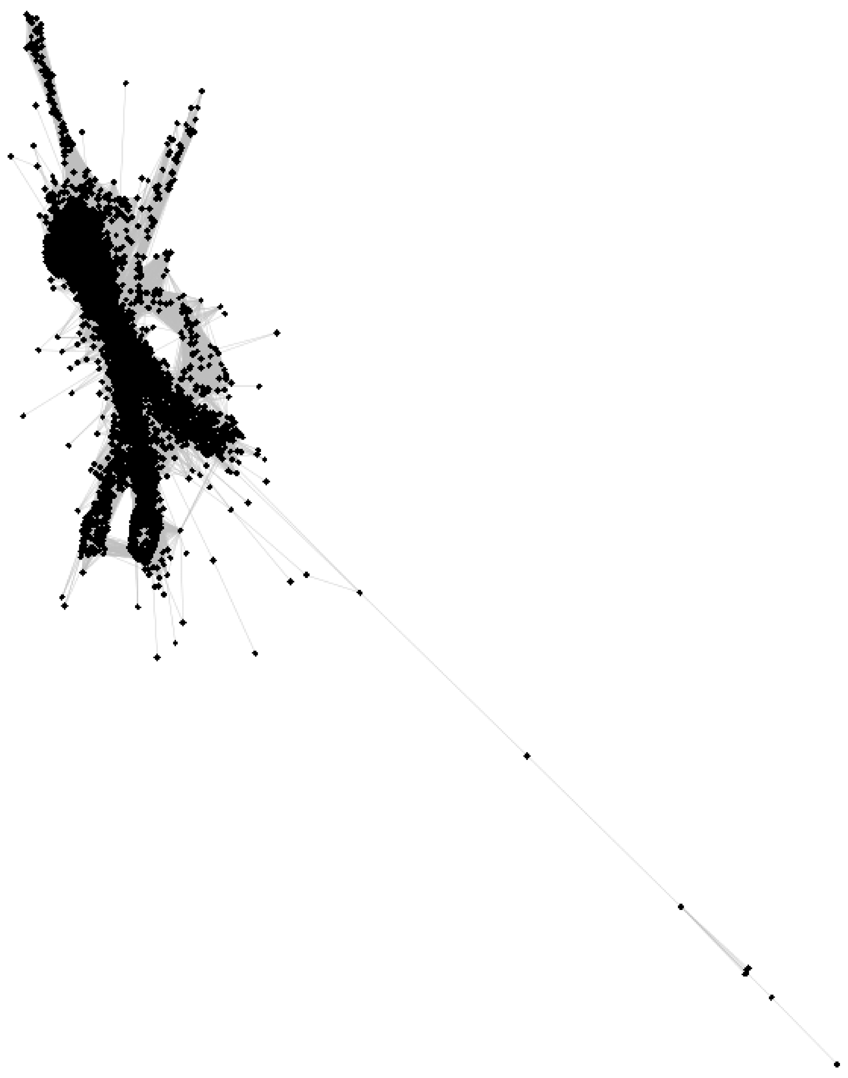

CL47

Number of reads: 9284
Number of pairs: 4711641
Density: 0.1093
Diameter: NA
Mean edge weigth: 165.48
Max. degree: 2182

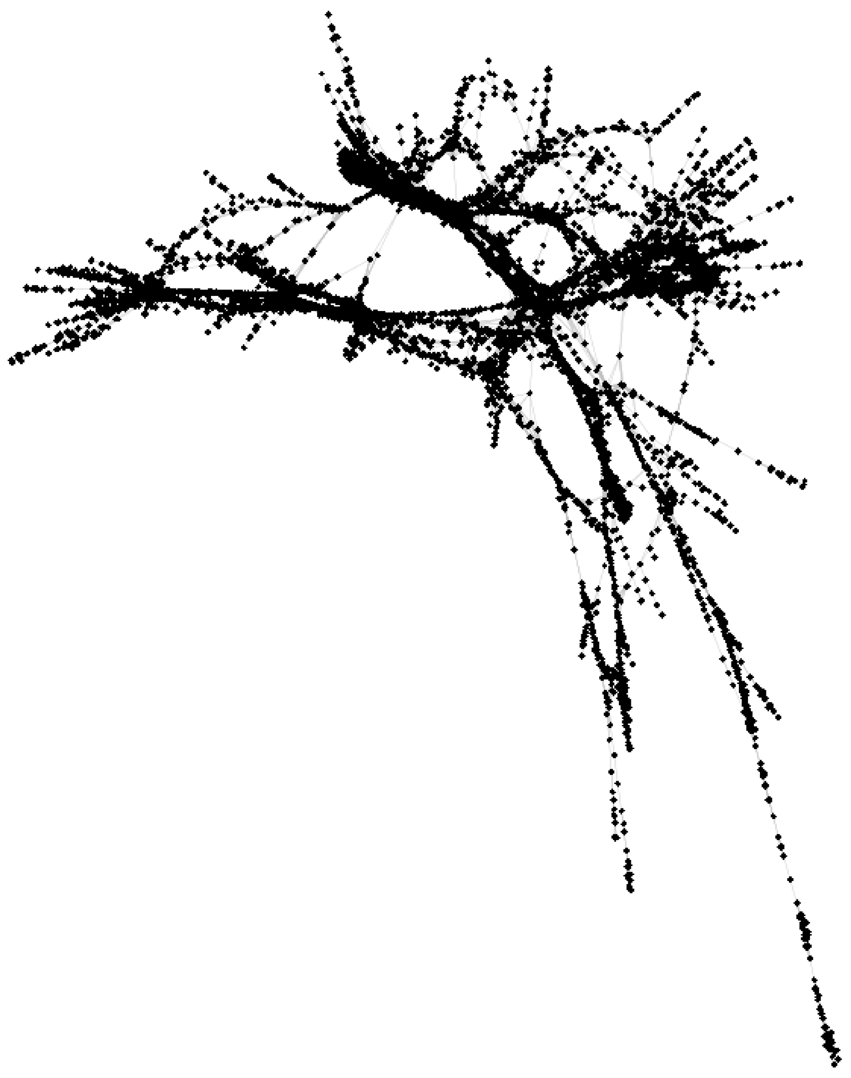

CL48

Number of reads: 9266
Number of pairs: 173654
Density: 0.004046
Diameter: NA
Mean edge weigth: 156.78
Max. degree: 269

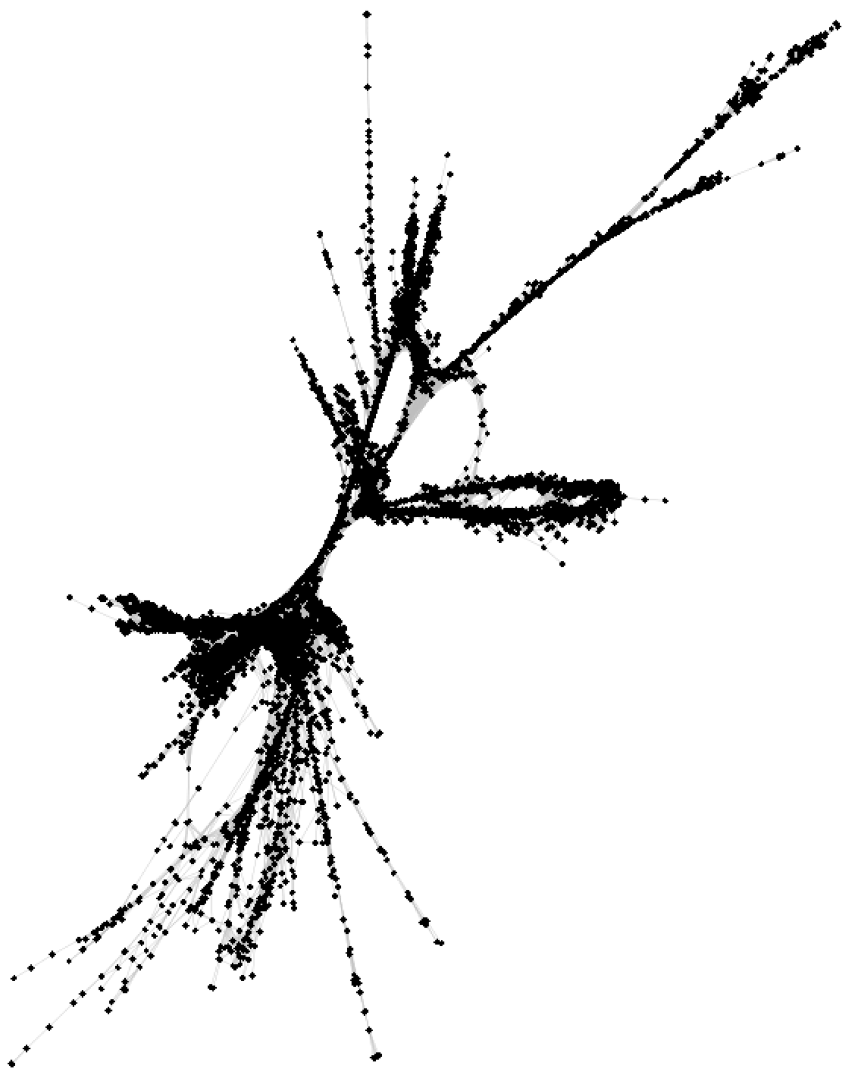

CL49

Number of reads: 9210
Number of pairs: 505955
Density: 0.01193
Diameter: NA
Mean edge weigth: 162.74
Max. degree: 486

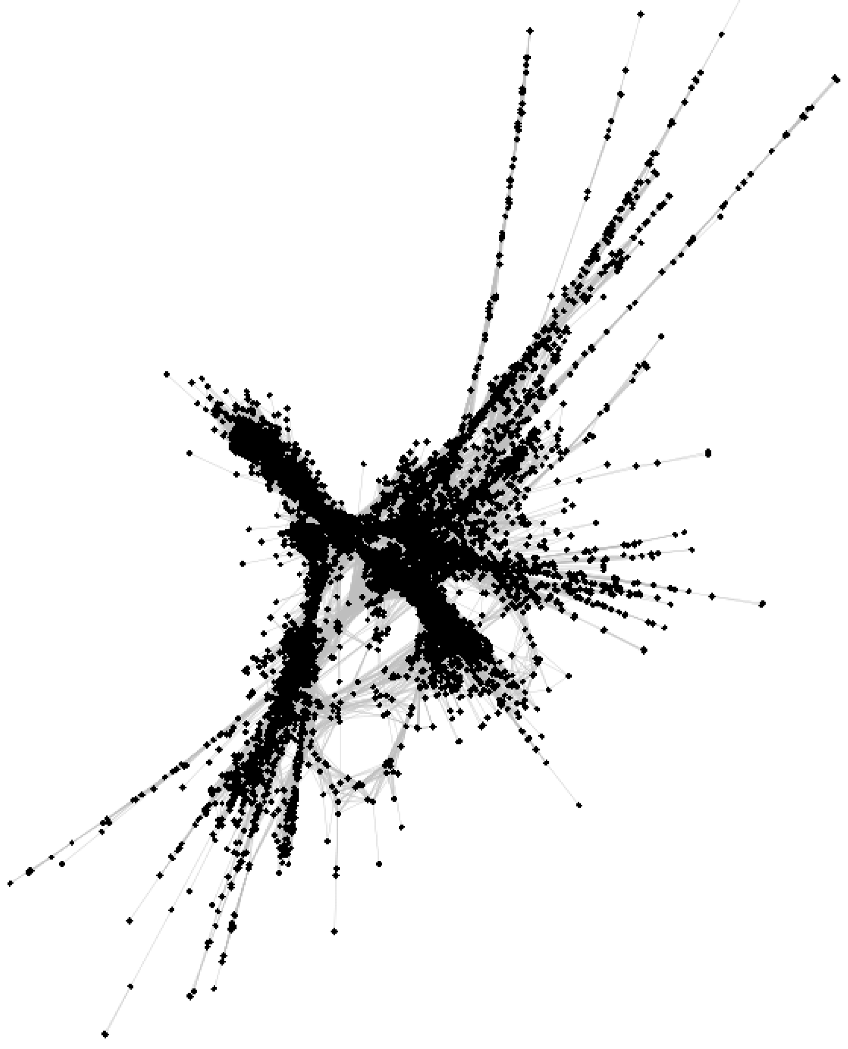

CL50

Number of reads: 9171
Number of pairs: 1178633
Density: 0.02803
Diameter: NA
Mean edge weigth: 158.51
Max. degree: 934

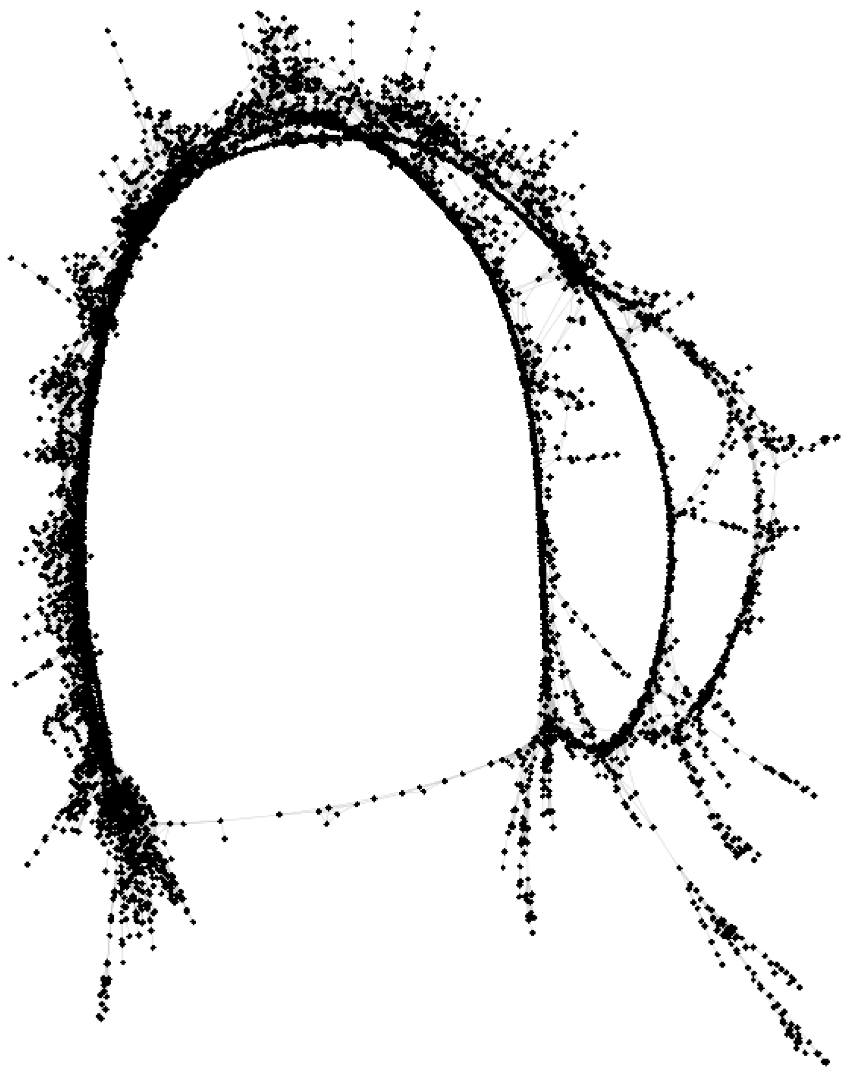

CL51

Number of reads: 9086
Number of pairs: 222345
Density: 0.005387
Diameter: NA
Mean edge weigth: 161.5
Max. degree: 231

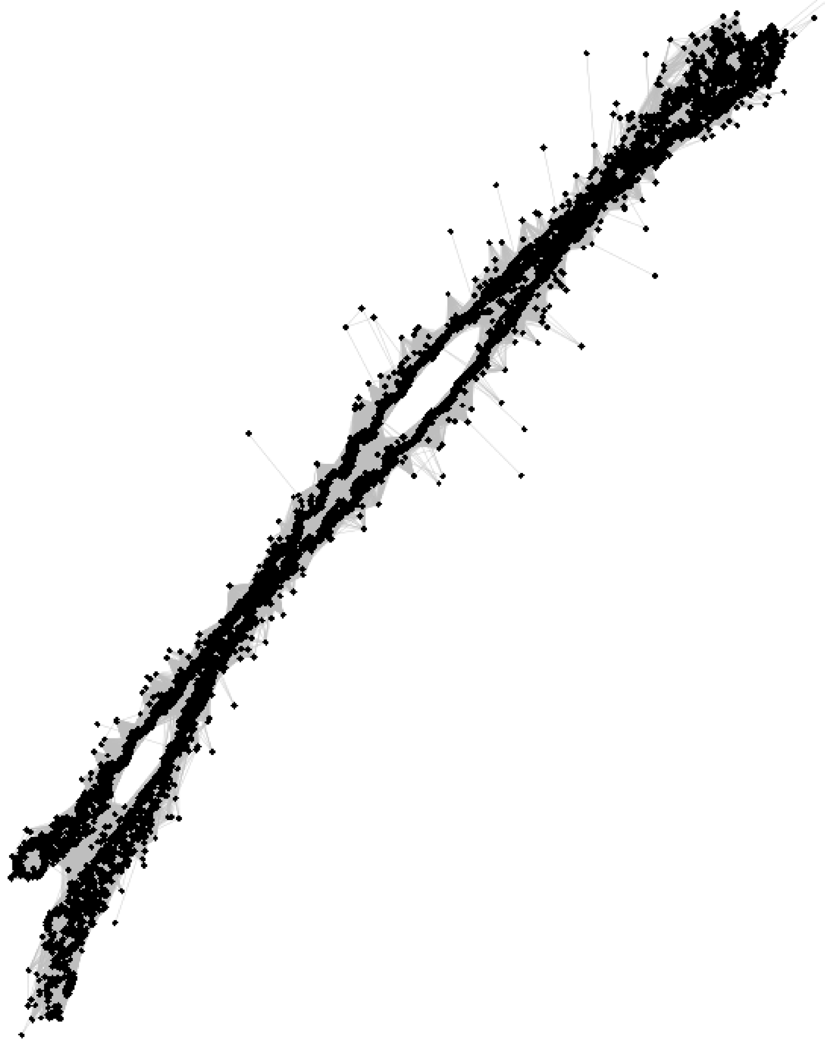

CL52

Number of reads: 8922
Number of pairs: 1559658
Density: 0.03919
Diameter: NA
Mean edge weigth: 168.43
Max. degree: 766

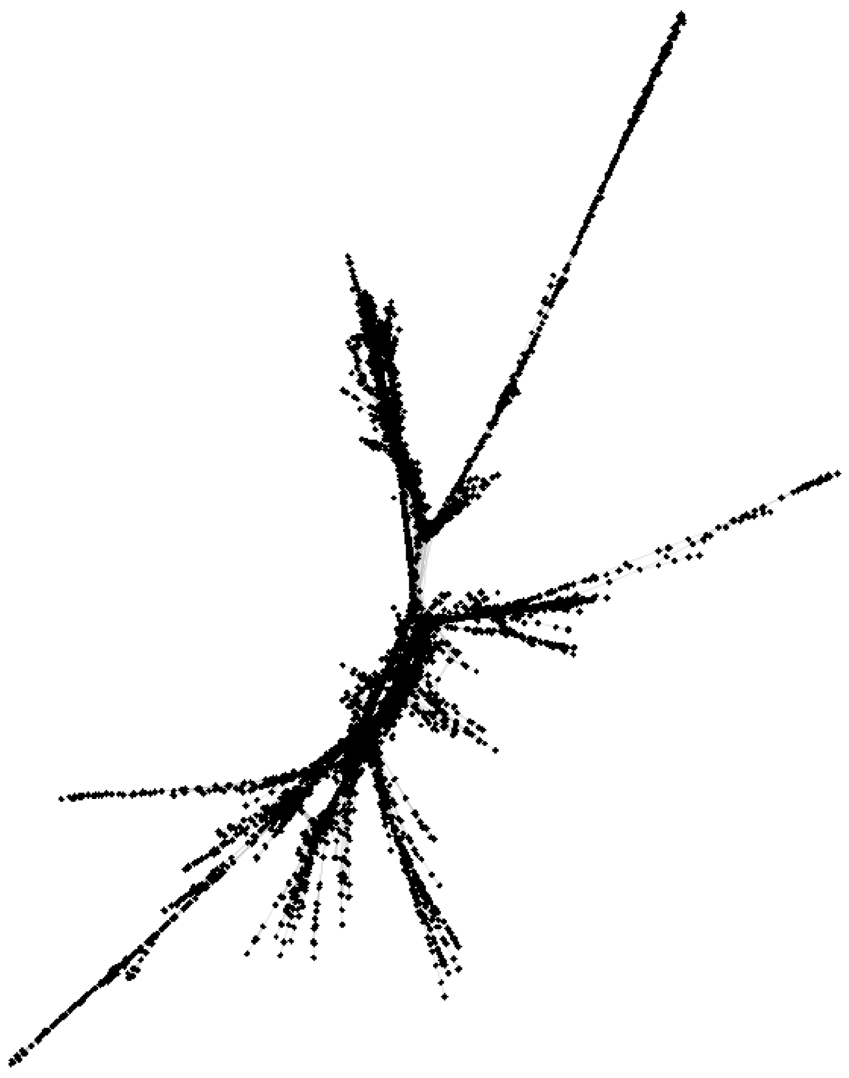

CL53

Number of reads: 8823
Number of pairs: 329086
Density: 0.008456
Diameter: NA
Mean edge weigth: 155.97
Max. degree: 457

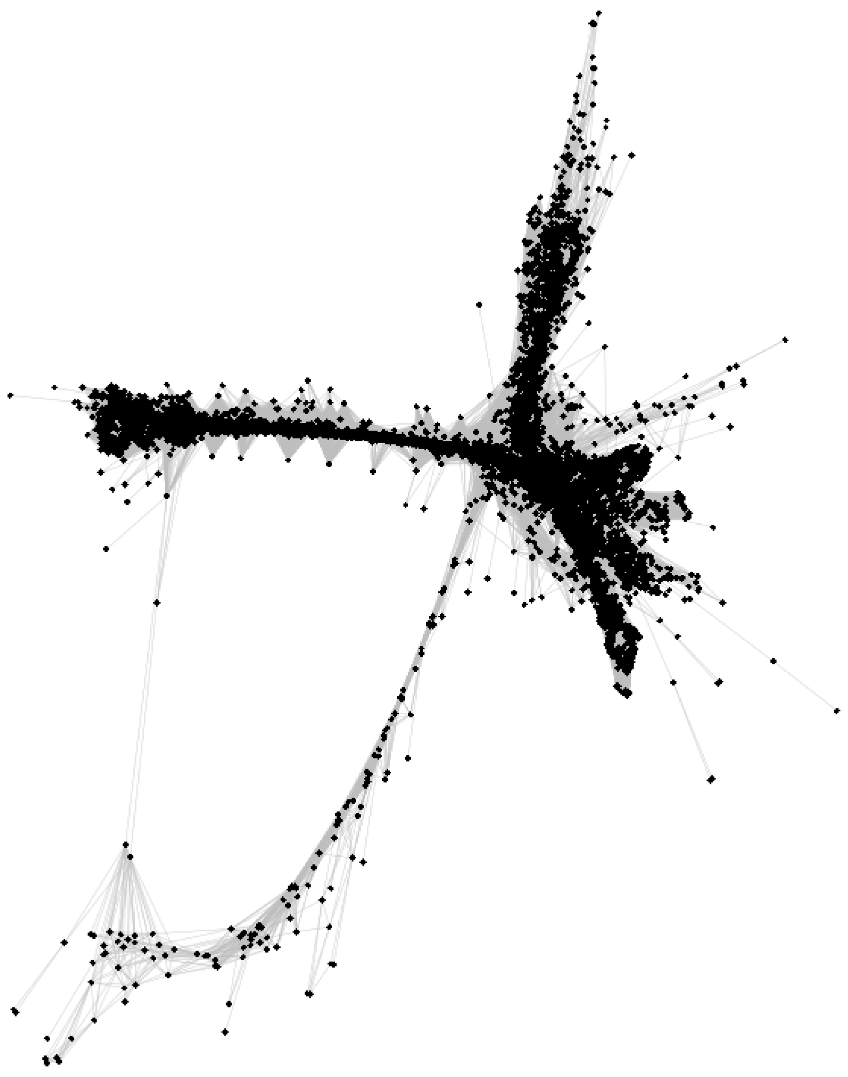

CL54

Number of reads: 8629
Number of pairs: 2354309
Density: 0.06324
Diameter: NA
Mean edge weigth: 164.89
Max. degree: 1382

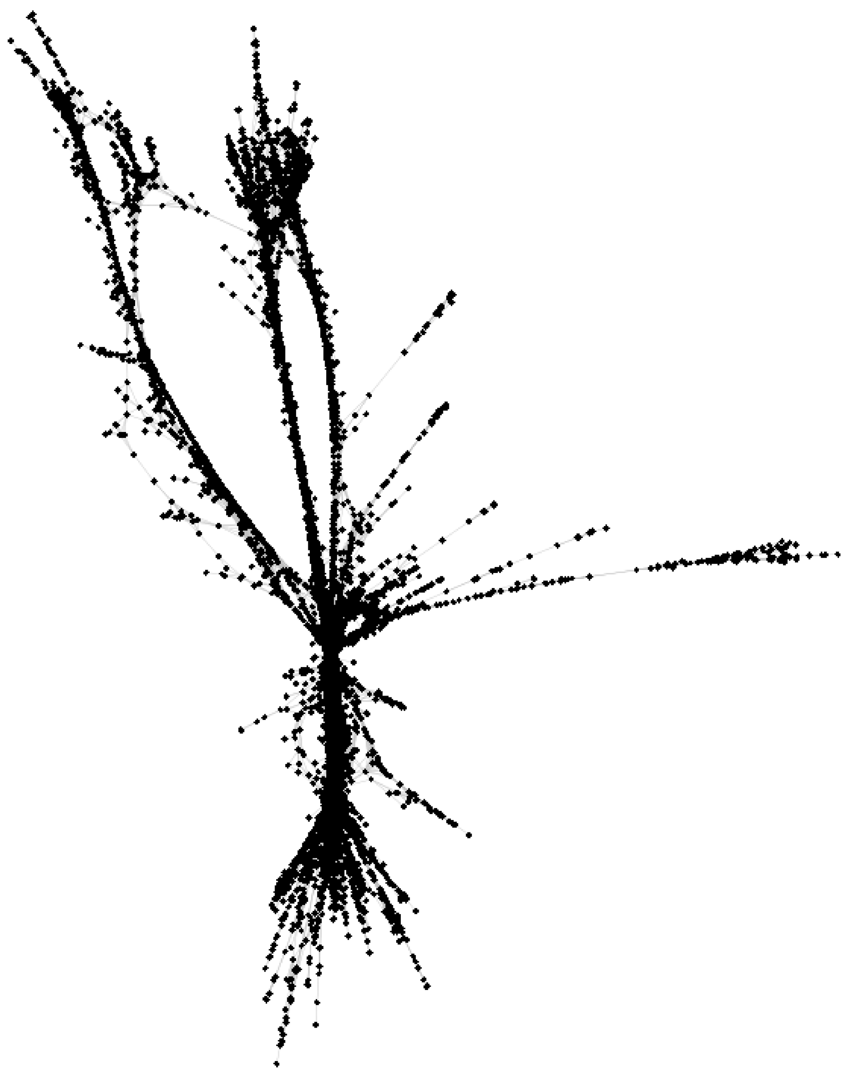

CL55

Number of reads: 8535
Number of pairs: 370717
Density: 0.01018
Diameter: NA
Mean edge weigth: 157.39
Max. degree: 678

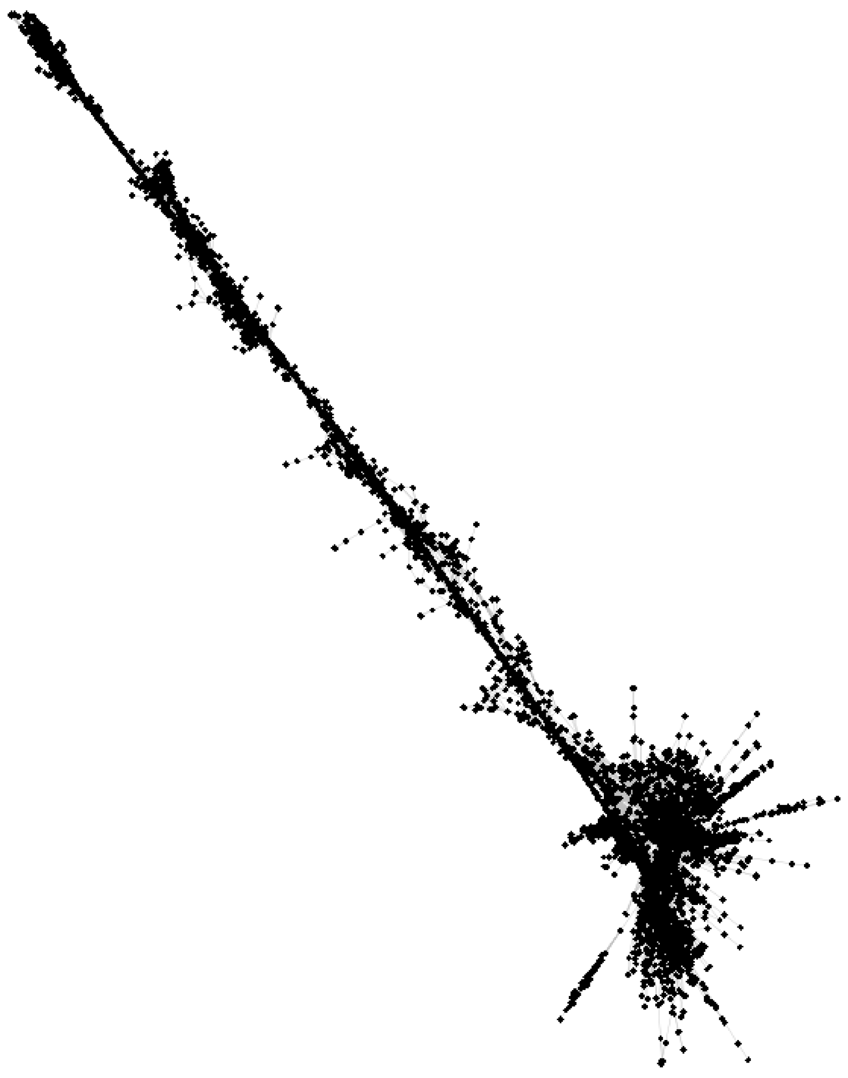

CL56

Number of reads: 8386
Number of pairs: 466121
Density: 0.01326
Diameter: NA
Mean edge weigth: 160.62
Max. degree: 649

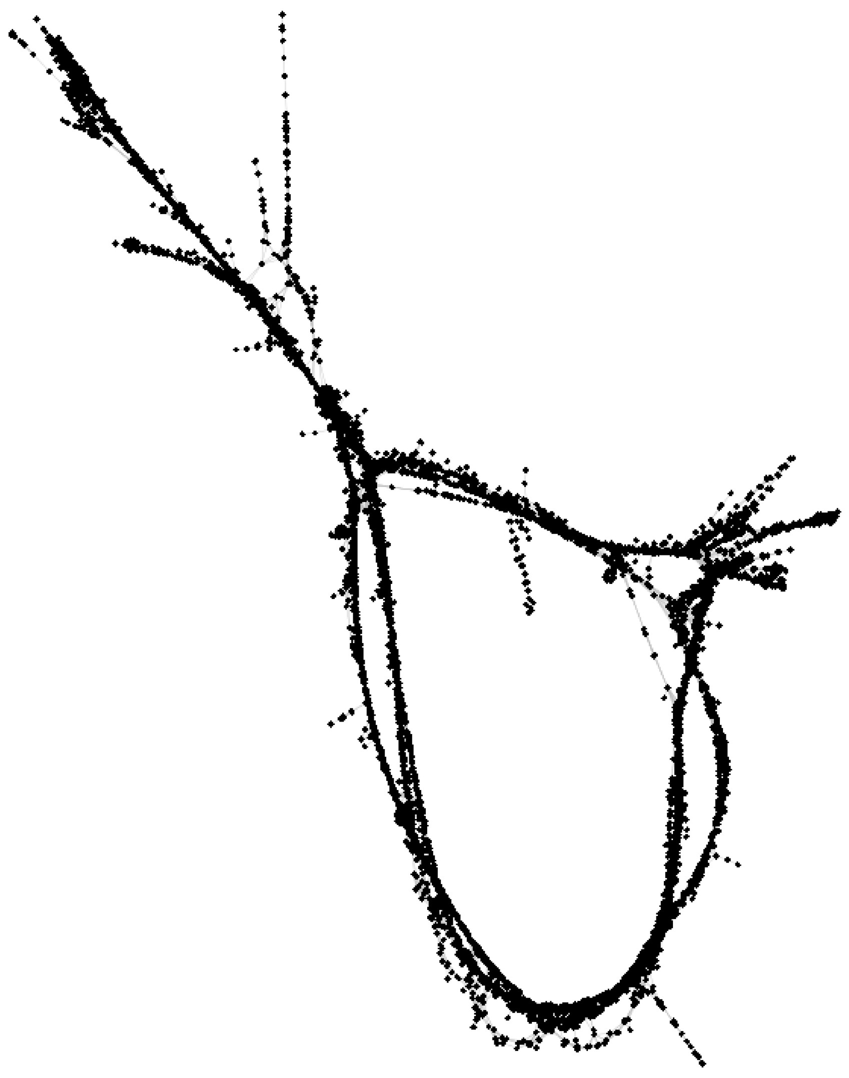

CL57

Number of reads: 8225
Number of pairs: 193301
Density: 0.005715
Diameter: NA
Mean edge weigth: 164.43
Max. degree: 232

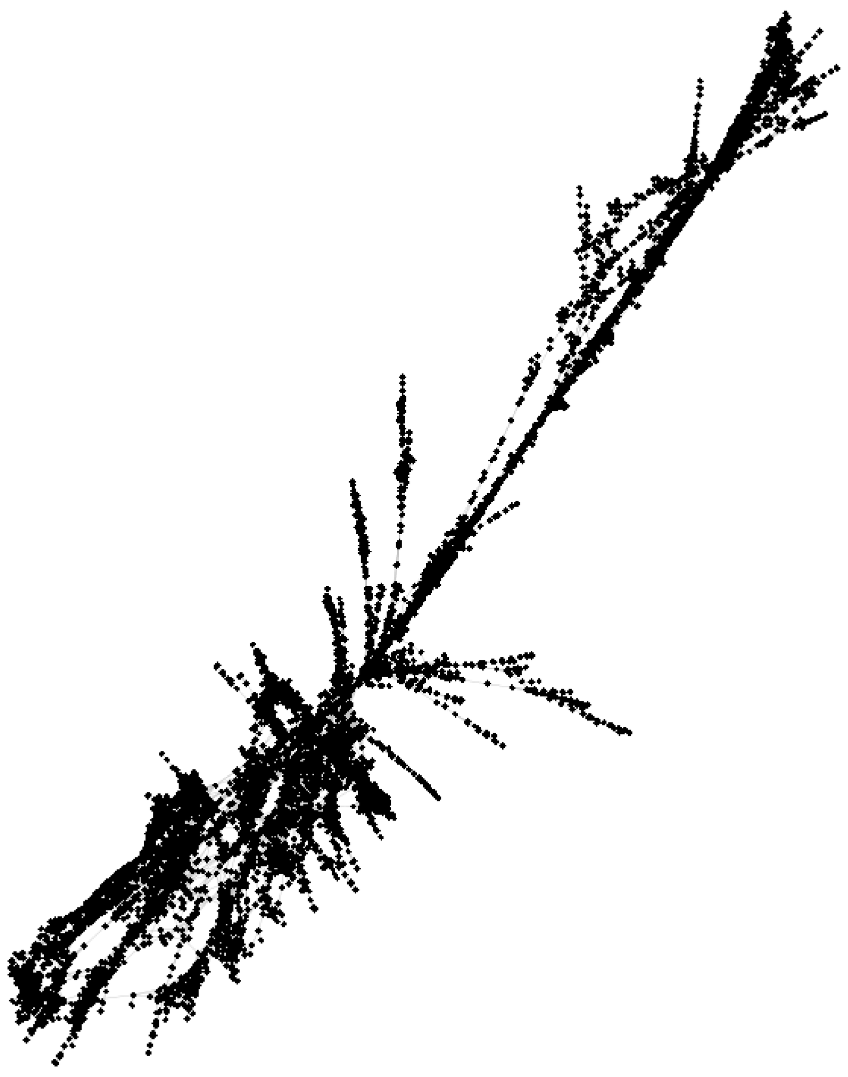

CL58

Number of reads: 8129
Number of pairs: 75817
Density: 0.002295
Diameter: NA
Mean edge weigth: 156.53
Max. degree: 104

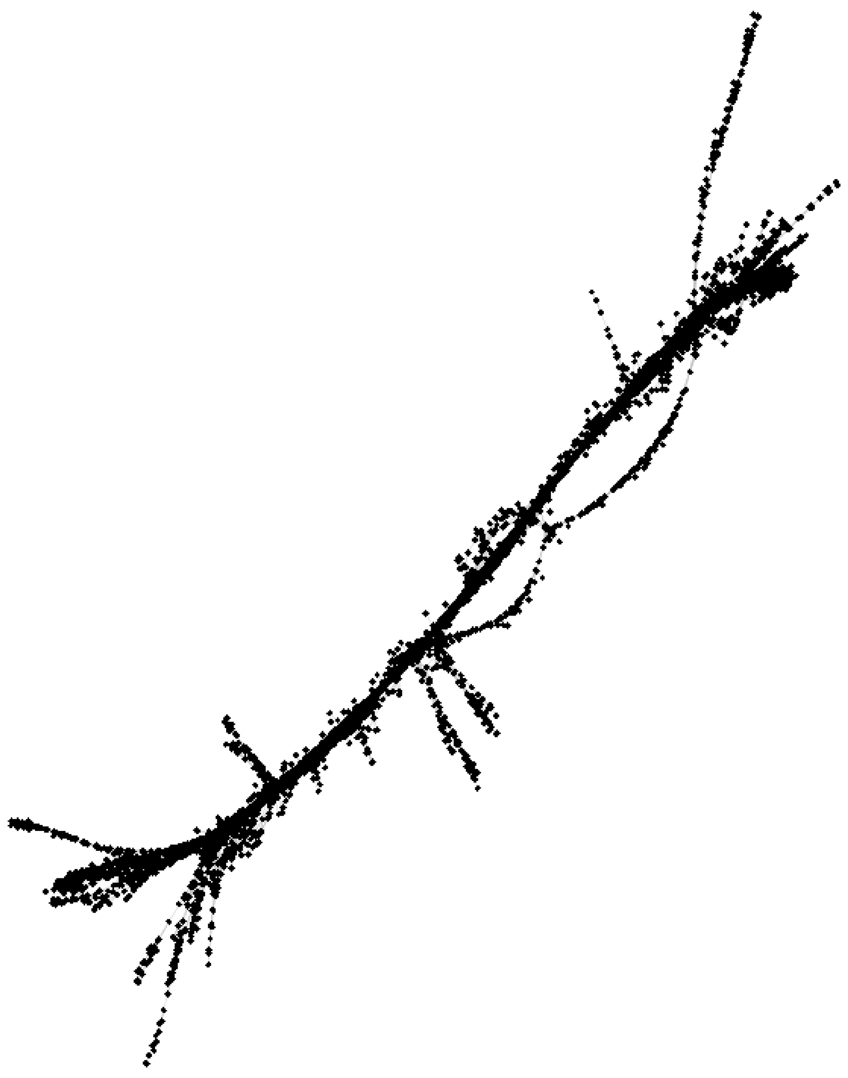

CL59

Number of reads: 8049
Number of pairs: 280544
Density: 0.008662
Diameter: NA
Mean edge weigth: 161.38
Max. degree: 268

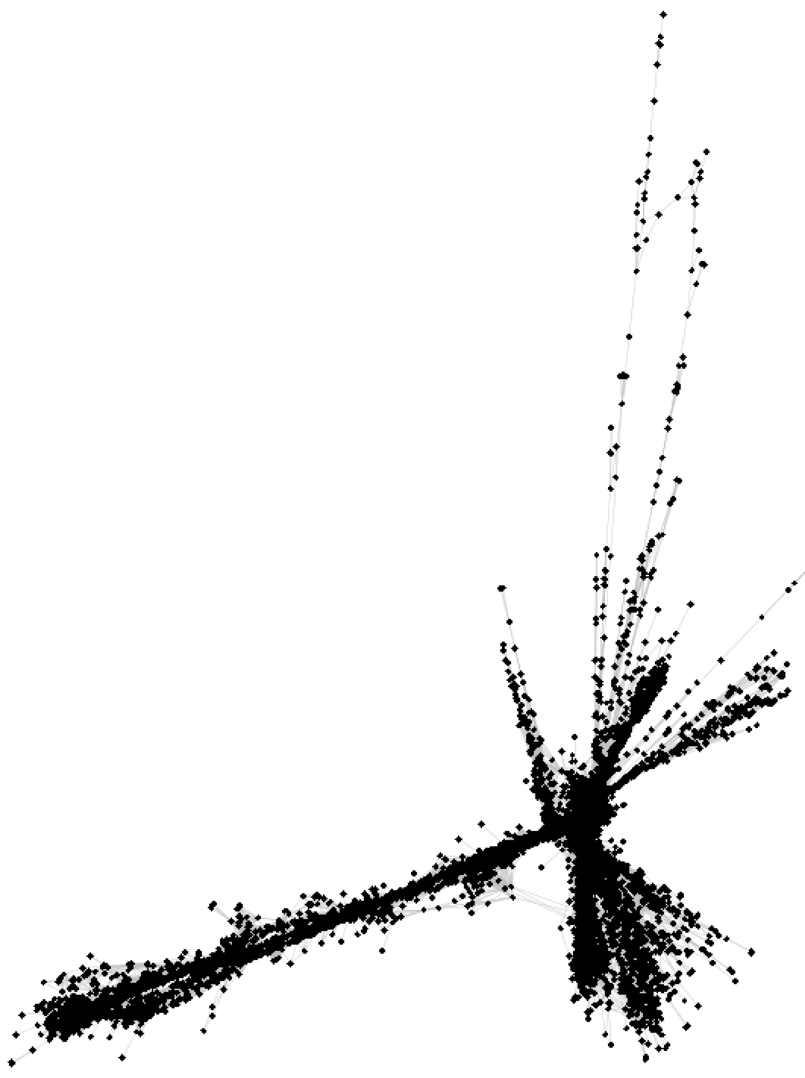

CL60

Number of reads: 7948
Number of pairs: 431868
Density: 0.01367
Diameter: NA
Mean edge weigth: 153.92
Max. degree: 671

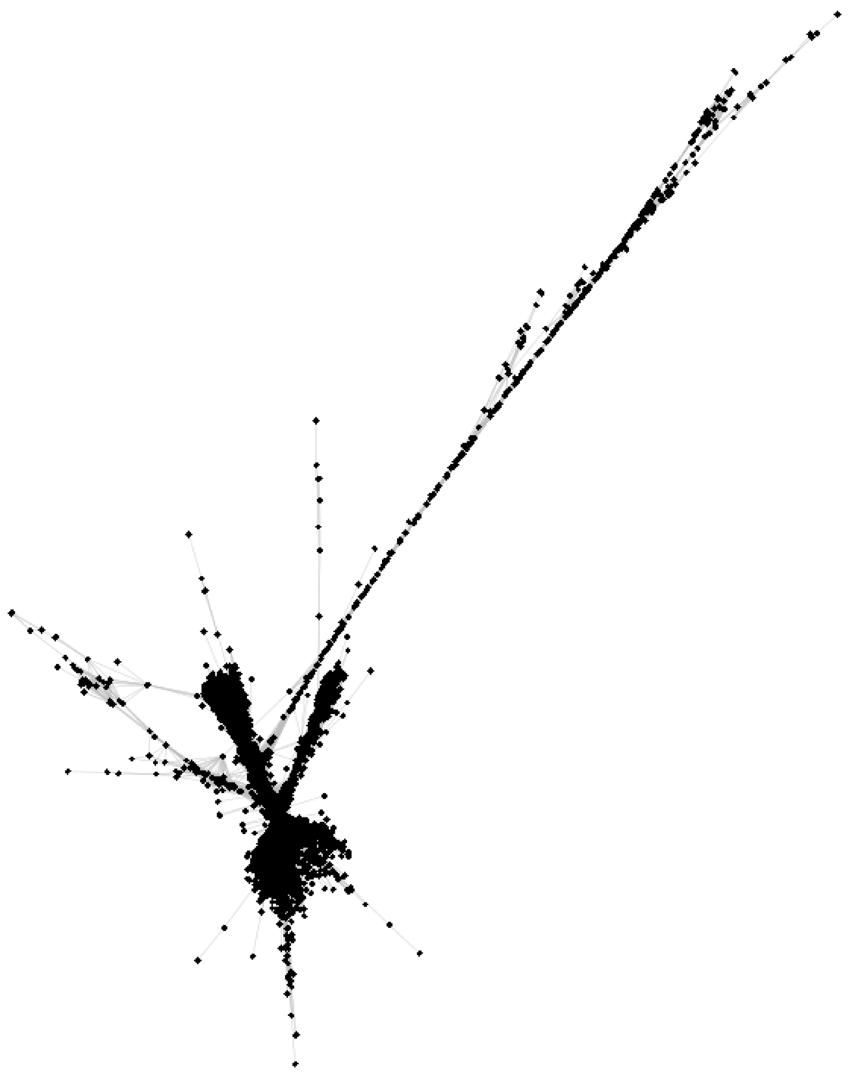

CL61

Number of reads: 7897
Number of pairs: 1855577
Density: 0.05952
Diameter: NA
Mean edge weigth: 153.7
Max. degree: 2014

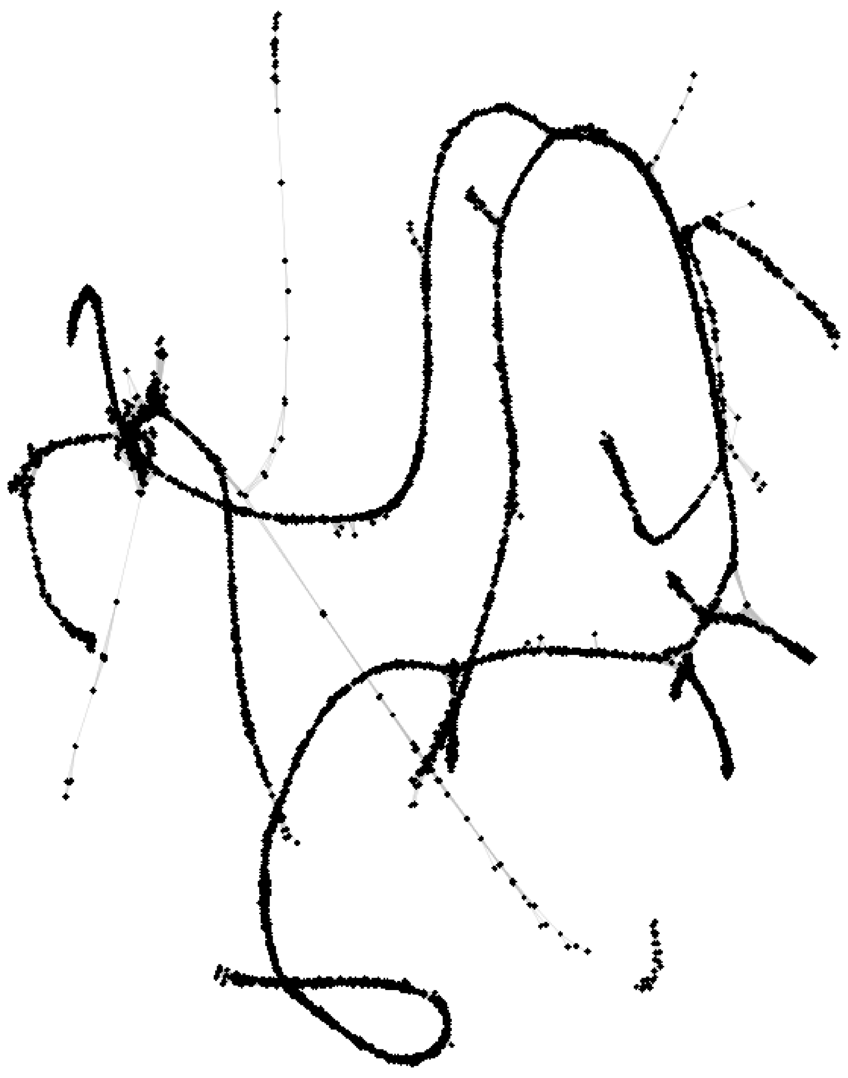

CL62

Number of reads: 7512
Number of pairs: 232587
Density: 0.008244
Diameter: NA
Mean edge weigth: 210.28
Max. degree: 230

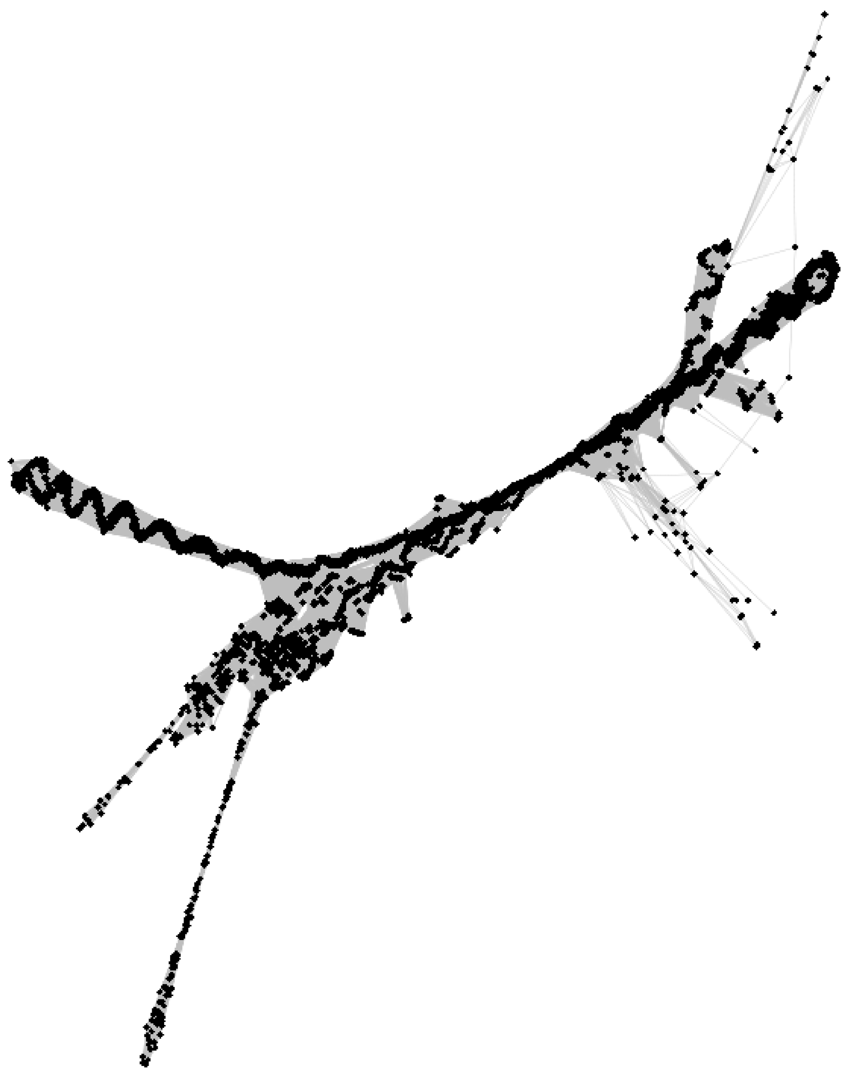

CL63

Number of reads: 7361
Number of pairs: 2014666
Density: 0.07437
Diameter: NA
Mean edge weigth: 206.38
Max. degree: 906

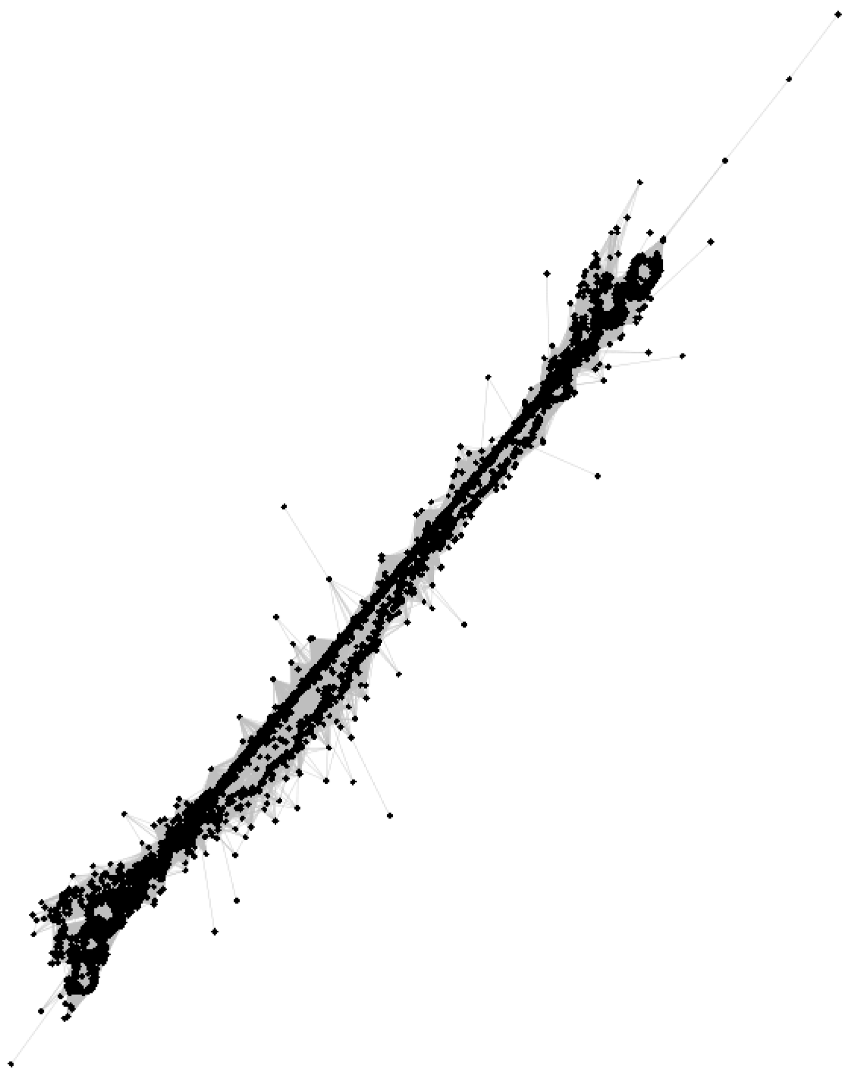

CL64

Number of reads: 7353
Number of pairs: 2032448
Density: 0.07519
Diameter: NA
Mean edge weigth: 169.74
Max. degree: 841

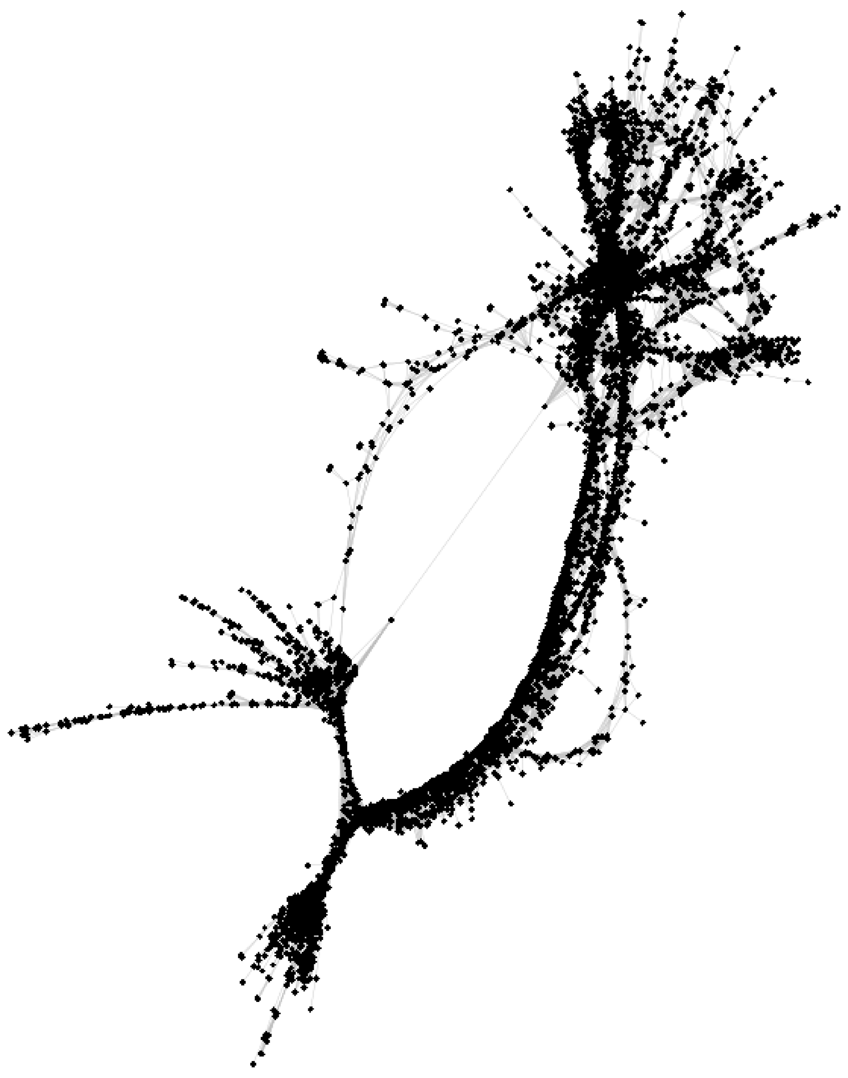

CL65

Number of reads: 7188
Number of pairs: 211960
Density: 0.008206
Diameter: NA
Mean edge weigth: 155.29
Max. degree: 274

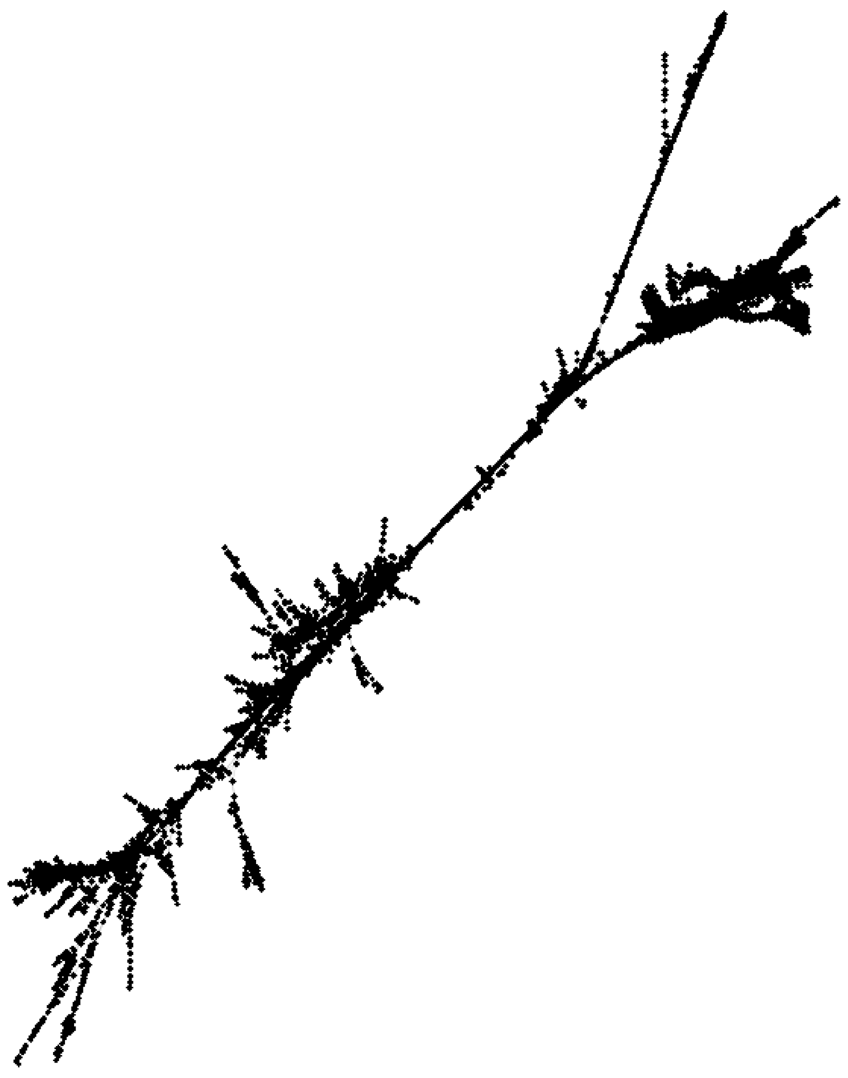

CL66

Number of reads: 7045
Number of pairs: 114150
Density: 0.004601
Diameter: NA
Mean edge weigth: 163.03
Max. degree: 205

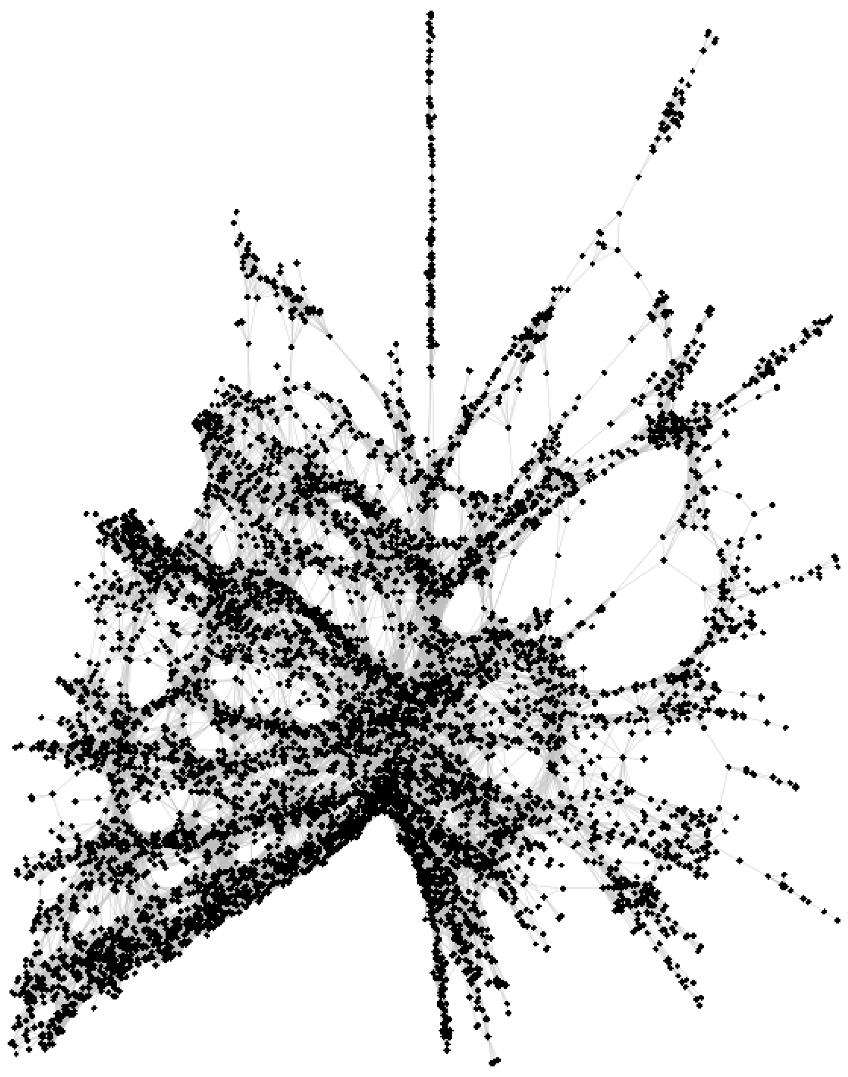

CL67

Number of reads: 7023
Number of pairs: 110965
Density: 0.0045
Diameter: NA
Mean edge weigth: 158.82
Max. degree: 113

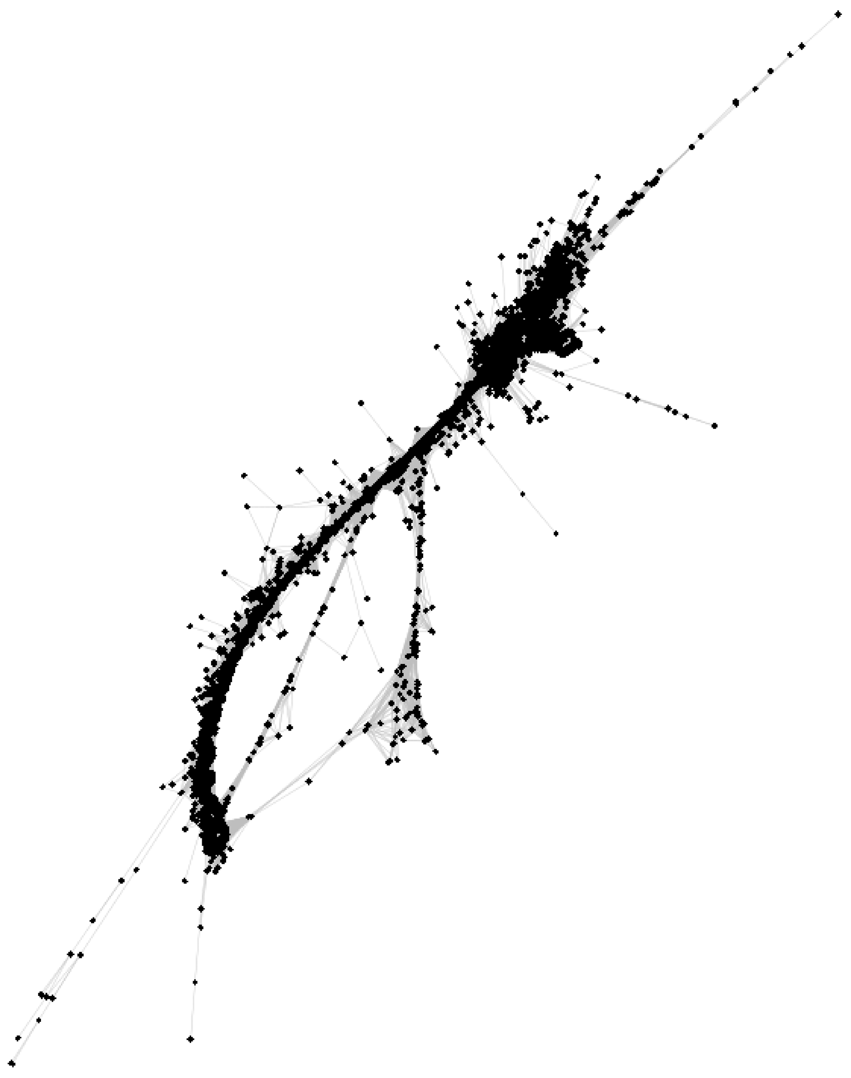

CL68

Number of reads: 6872
Number of pairs: 1272521
Density: 0.0539
Diameter: NA
Mean edge weigth: 162.68
Max. degree: 1011

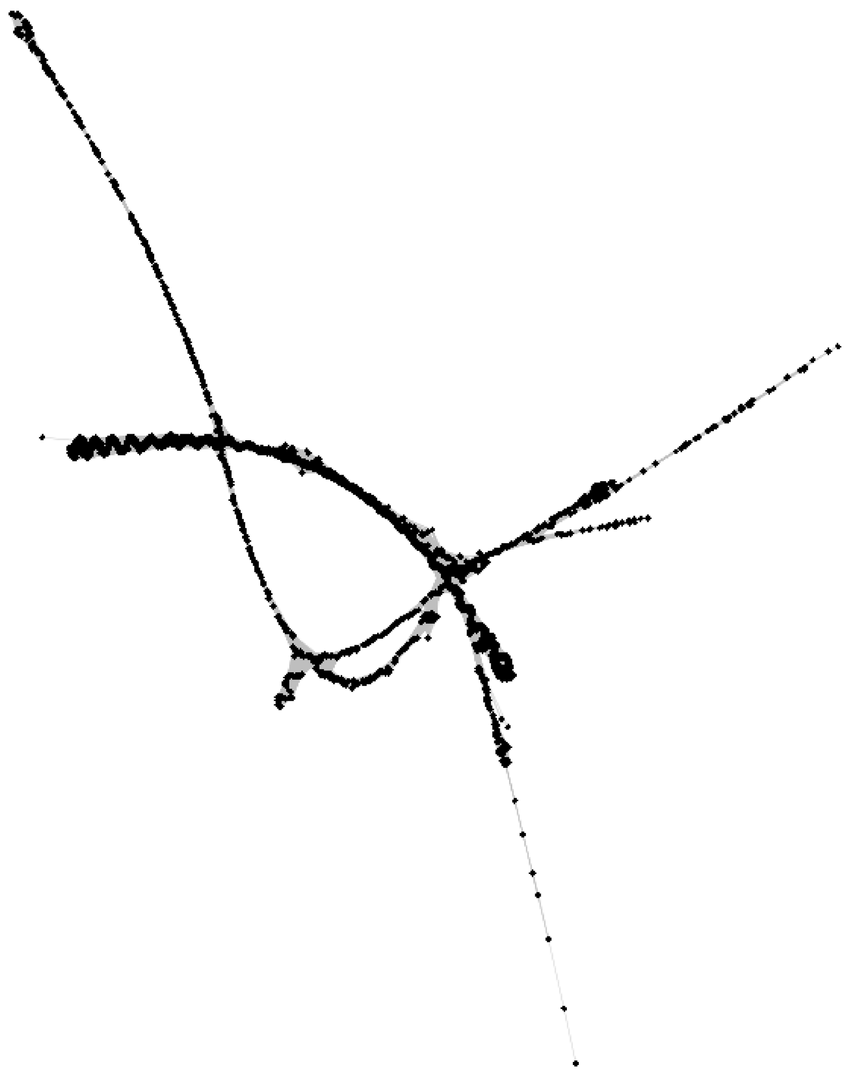

CL69

Number of reads: 6552
Number of pairs: 1405173
Density: 0.06548
Diameter: NA
Mean edge weigth: 211.06
Max. degree: 632

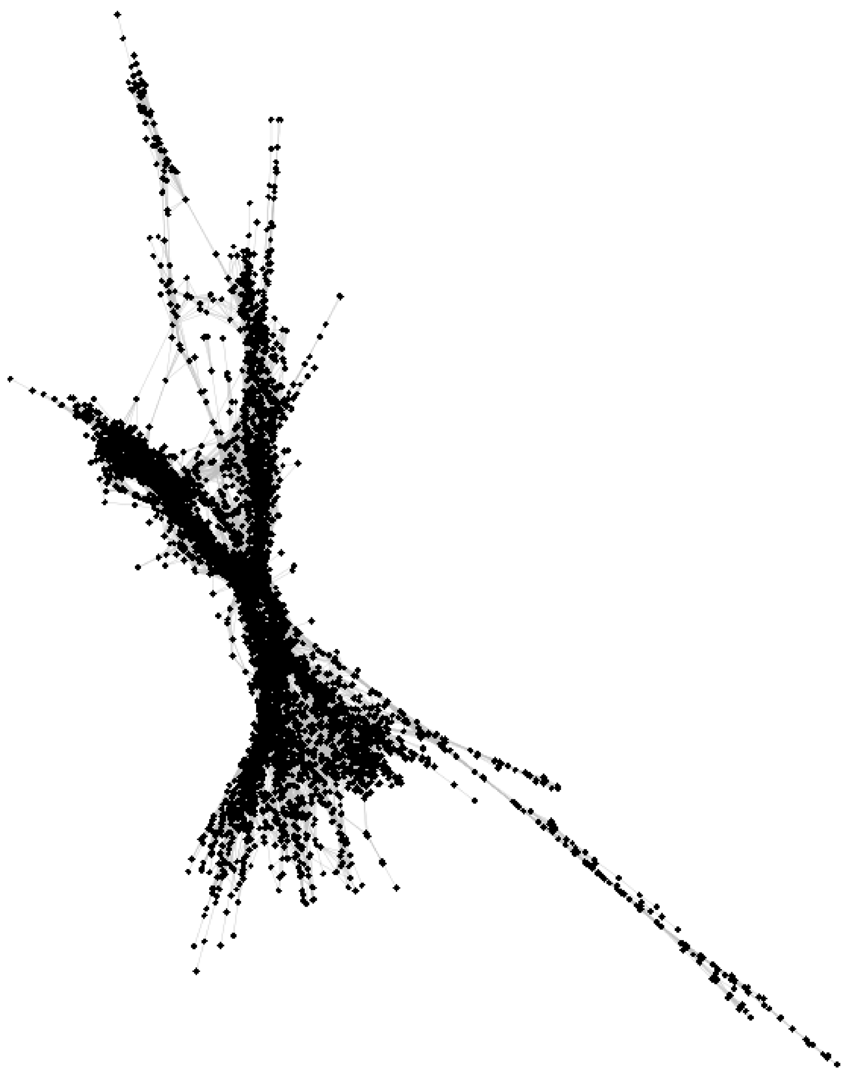

CL70

Number of reads: 6533
Number of pairs: 243125
Density: 0.01139
Diameter: NA
Mean edge weigth: 144.72
Max. degree: 416

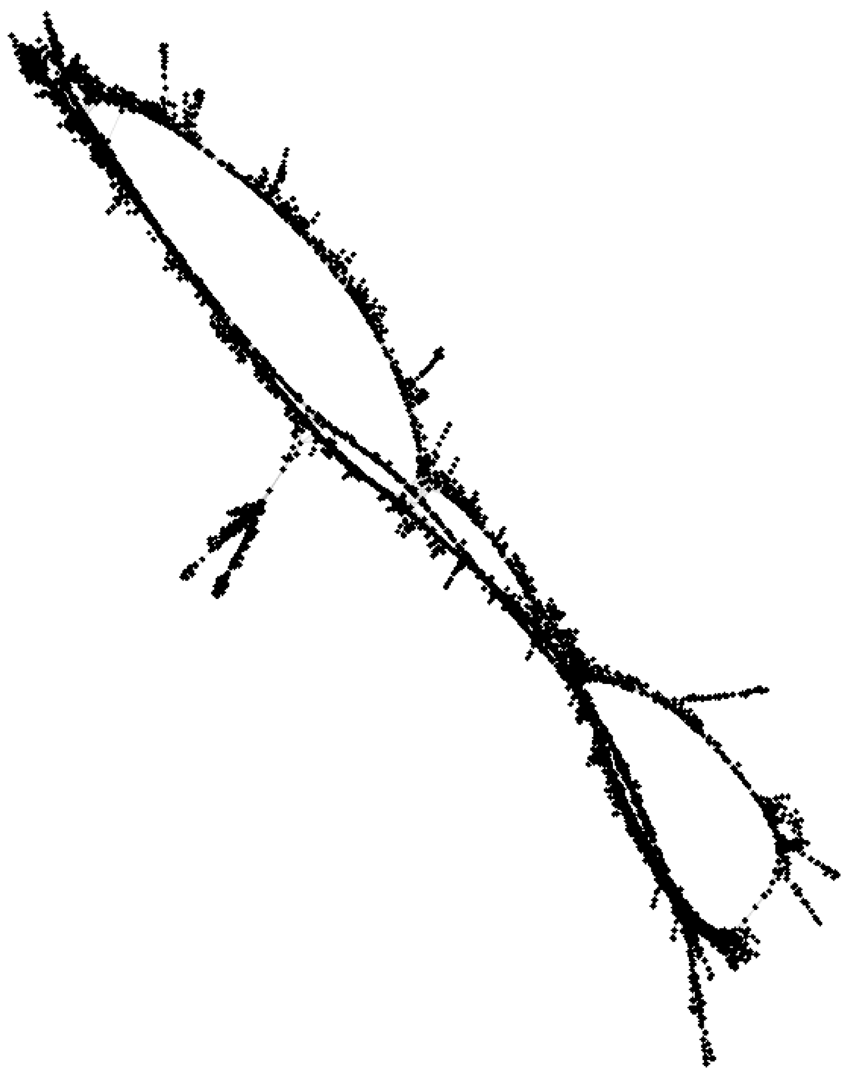

CL71

Number of reads: 6465
Number of pairs: 96847
Density: 0.004635
Diameter: NA
Mean edge weigth: 158.4
Max. degree: 119

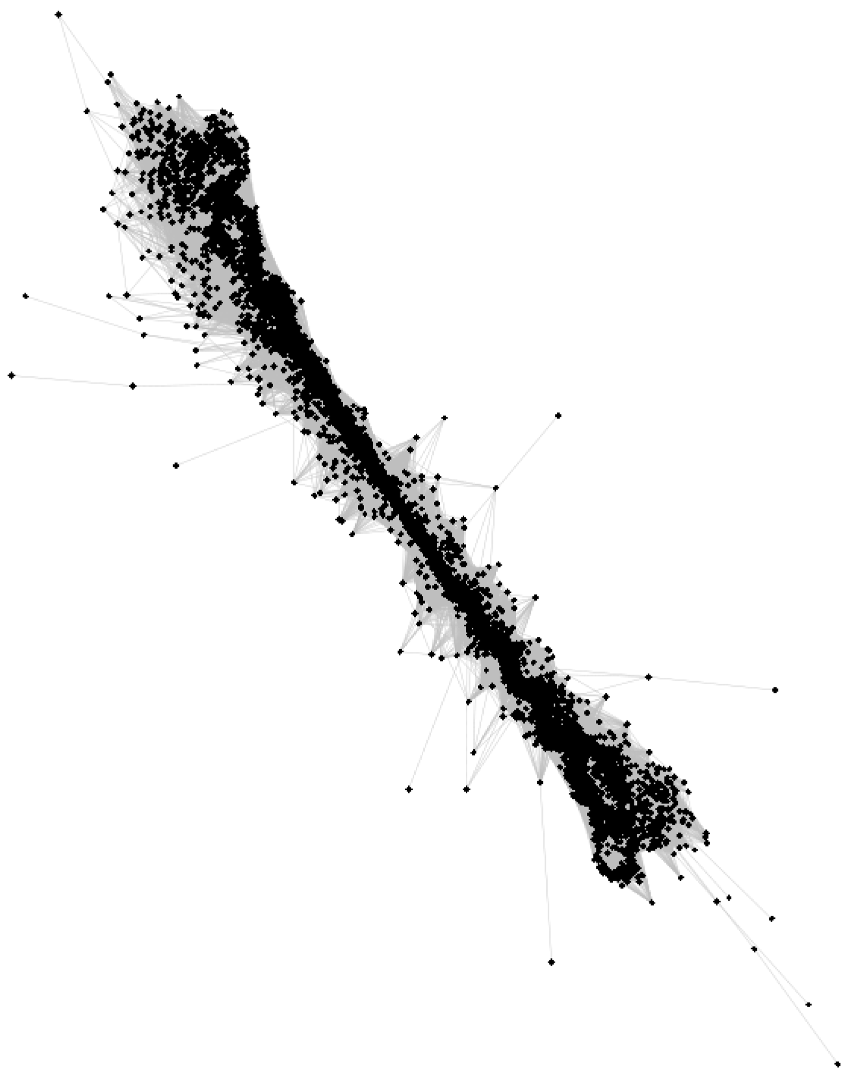

CL72

Number of reads: 6369
Number of pairs: 2482950
Density: 0.1224
Diameter: NA
Mean edge weigth: 167.48
Max. degree: 1173

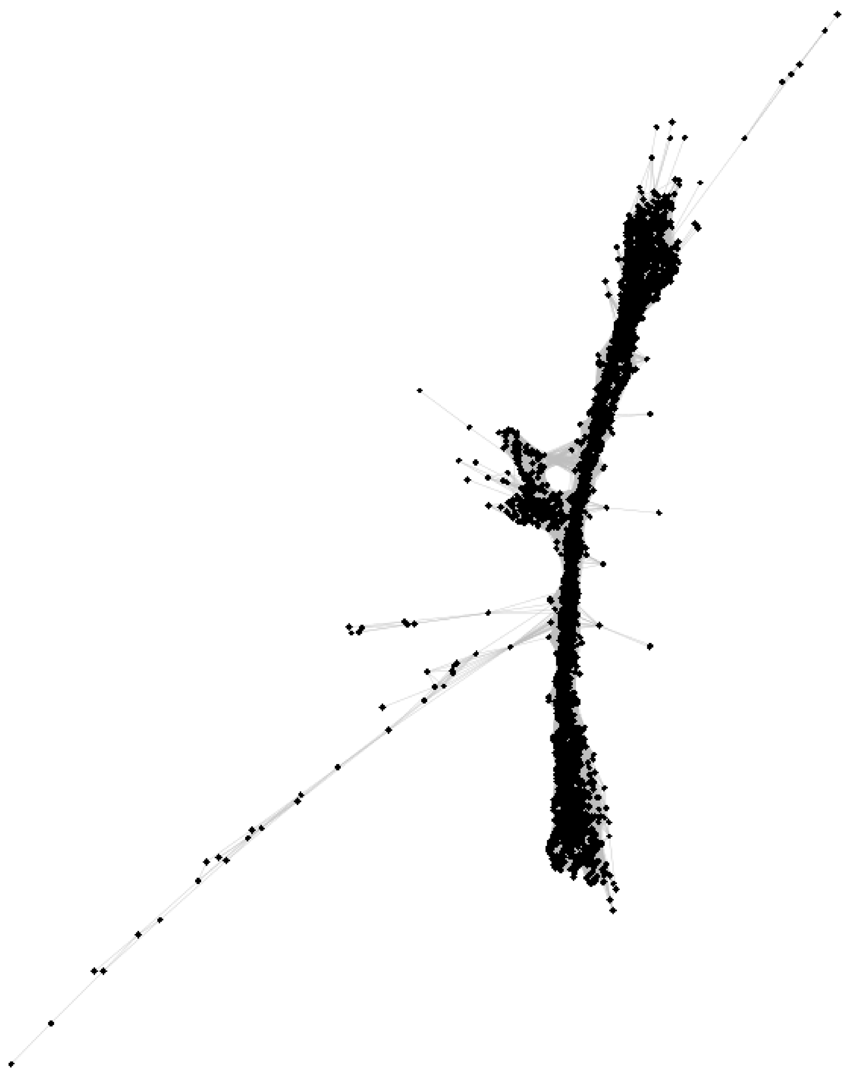

CL73

Number of reads: 6322
Number of pairs: 1426915
Density: 0.07141
Diameter: NA
Mean edge weigth: 165.64
Max. degree: 825

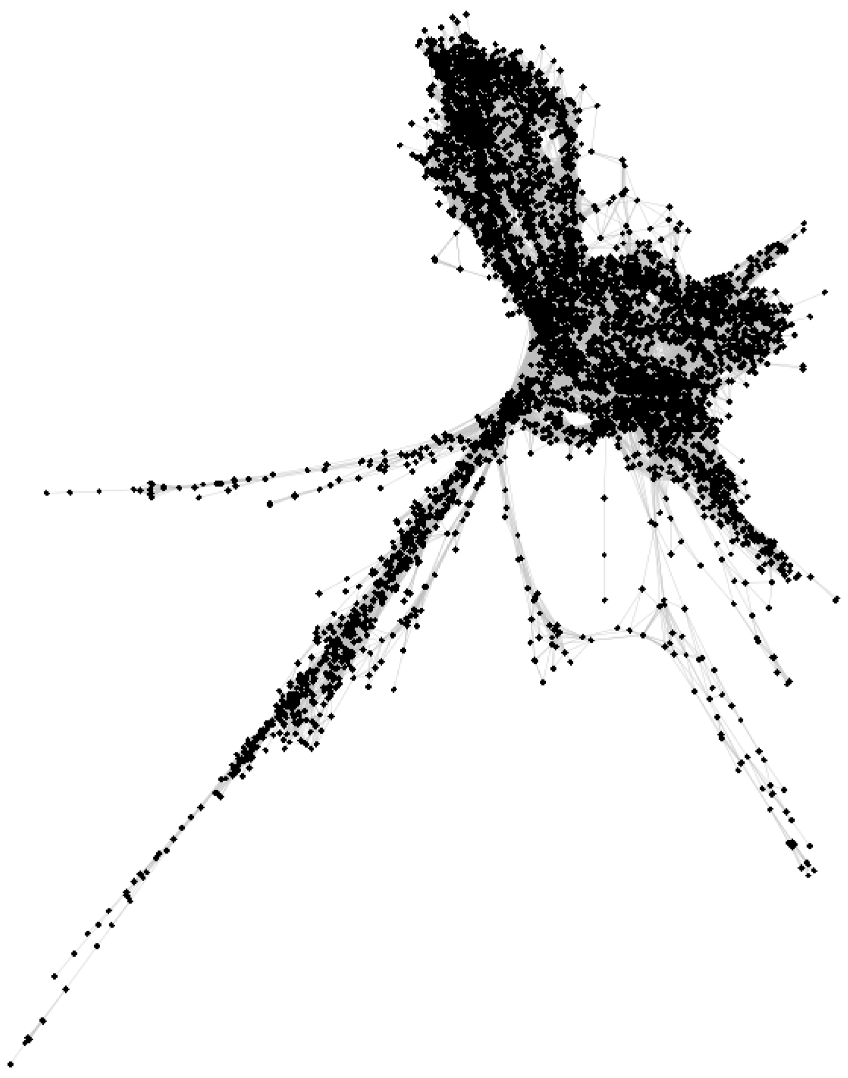

CL74

Number of reads: 6314
Number of pairs: 204852
Density: 0.01028
Diameter: NA
Mean edge weigth: 150.76
Max. degree: 274

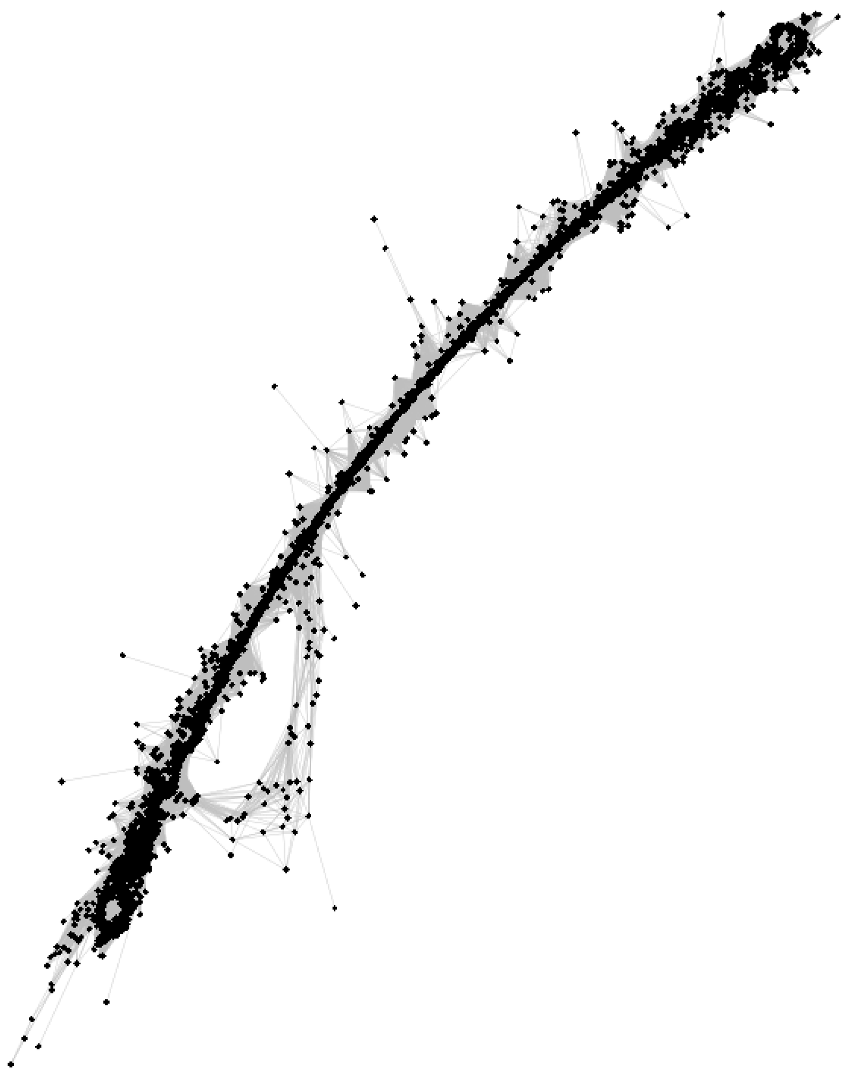

CL75

Number of reads: 6273
Number of pairs: 1443511
Density: 0.07338
Diameter: NA
Mean edge weigth: 168.13
Max. degree: 640

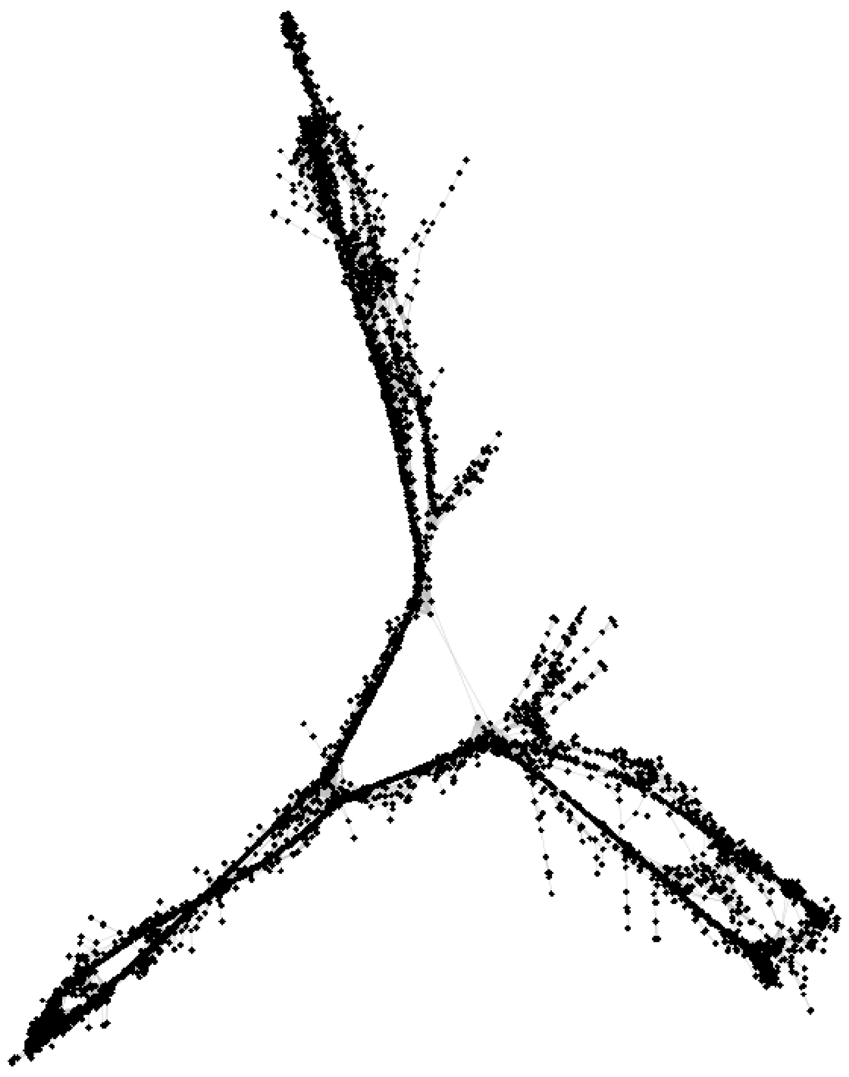

CL76

Number of reads: 6188
Number of pairs: 149922
Density: 0.007832
Diameter: NA
Mean edge weigth: 166.8
Max. degree: 168

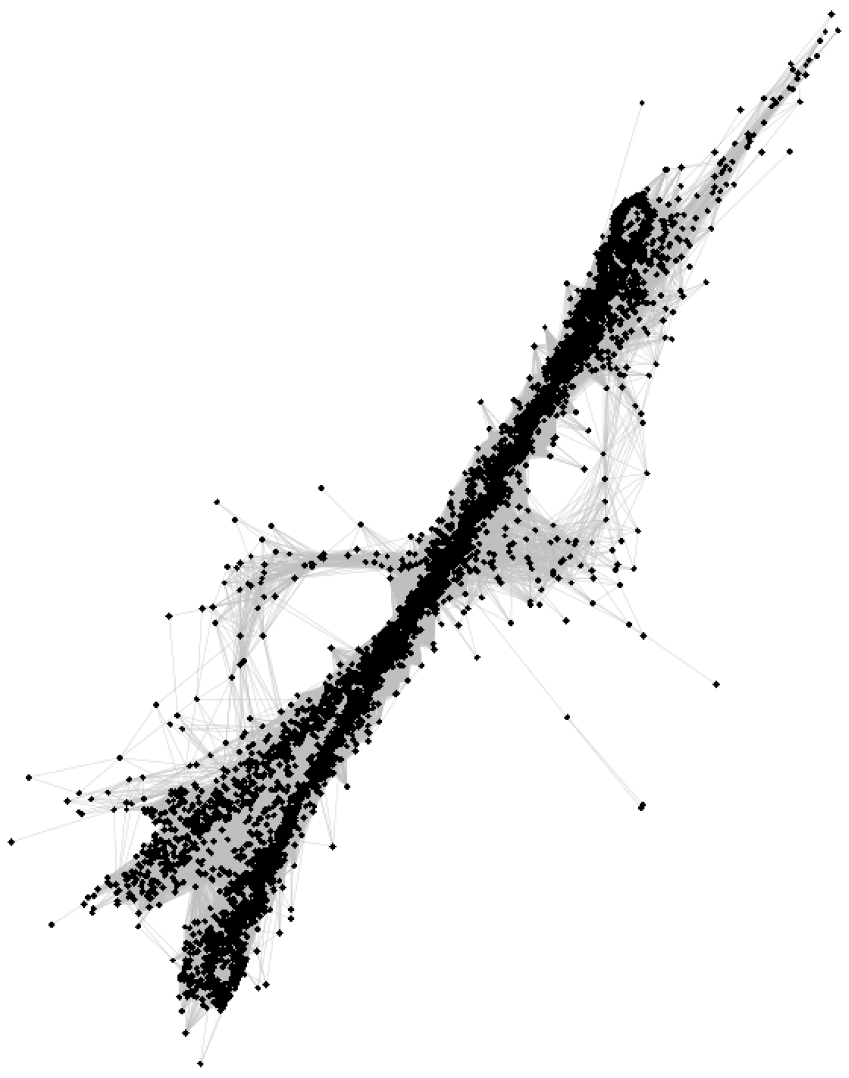

CL77

Number of reads: 6096
Number of pairs: 1512580
Density: 0.08142
Diameter: NA
Mean edge weigth: 164.31
Max. degree: 963

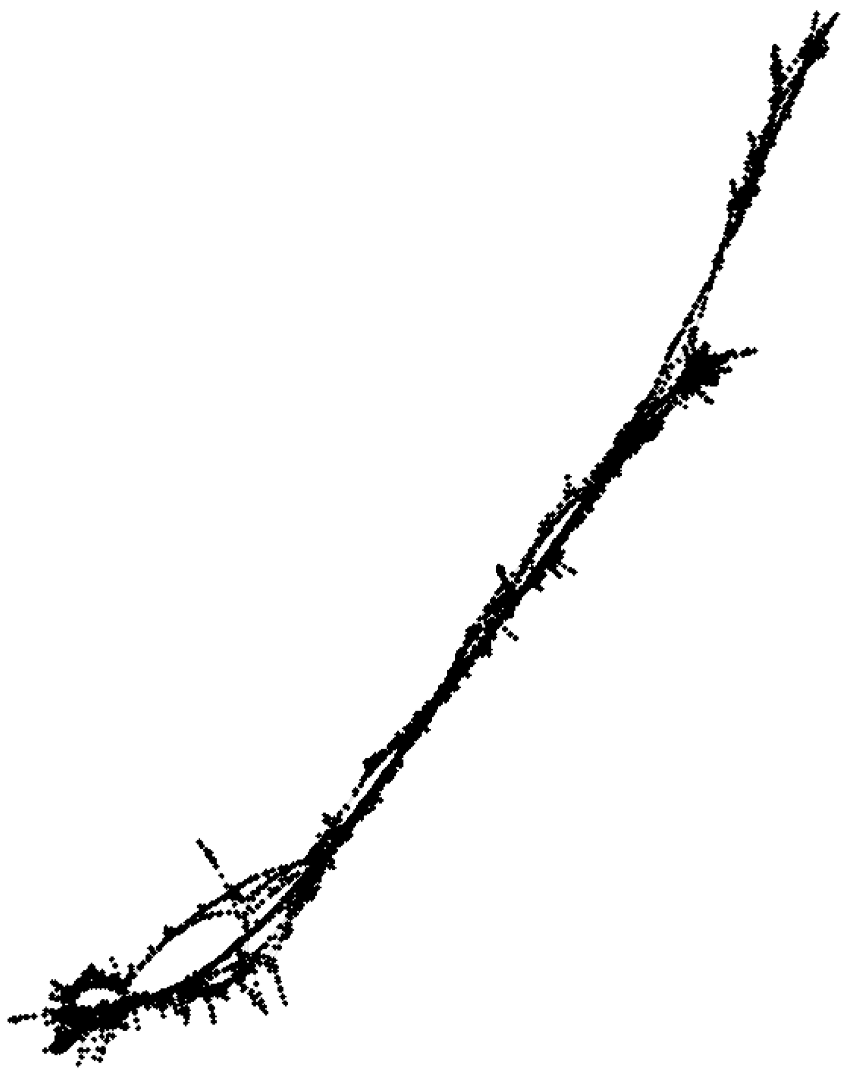

CL78

Number of reads: 5991
Number of pairs: 60031
Density: 0.003346
Diameter: NA
Mean edge weigth: 157.34
Max. degree: 94

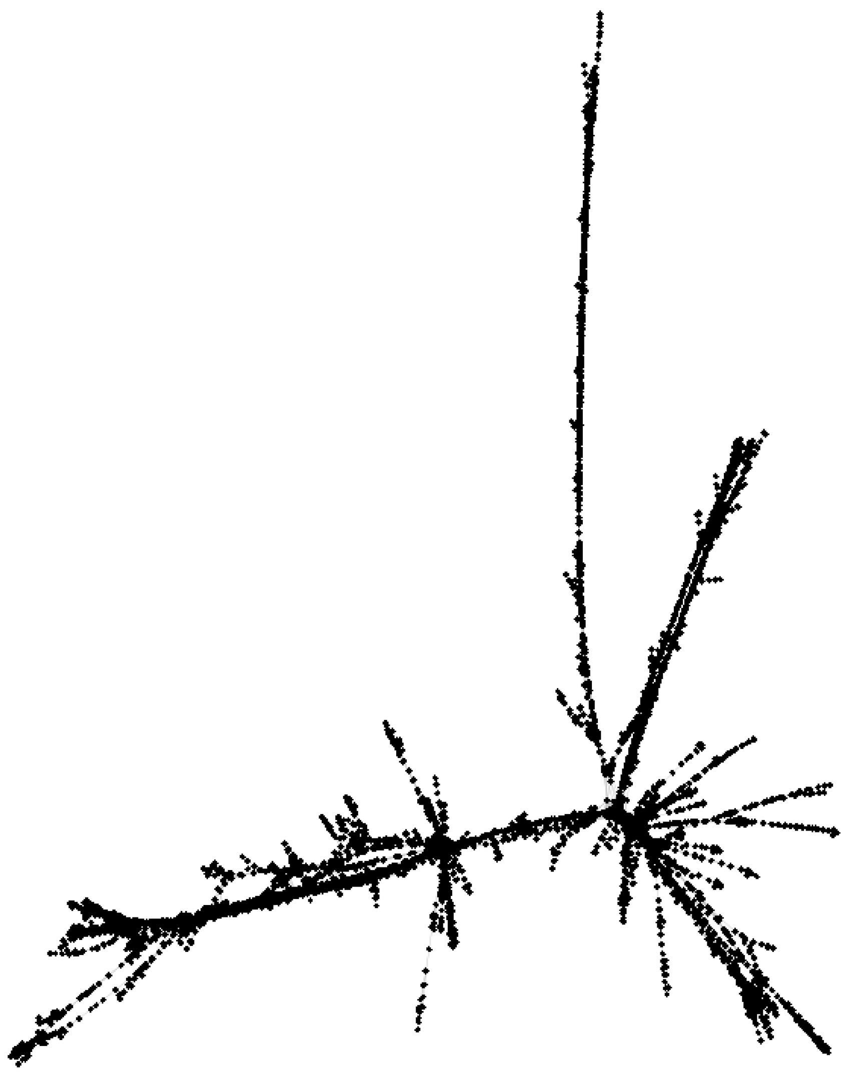

CL79

Number of reads: 5962
Number of pairs: 70563
Density: 0.003971
Diameter: NA
Mean edge weigth: 166.87
Max. degree: 128

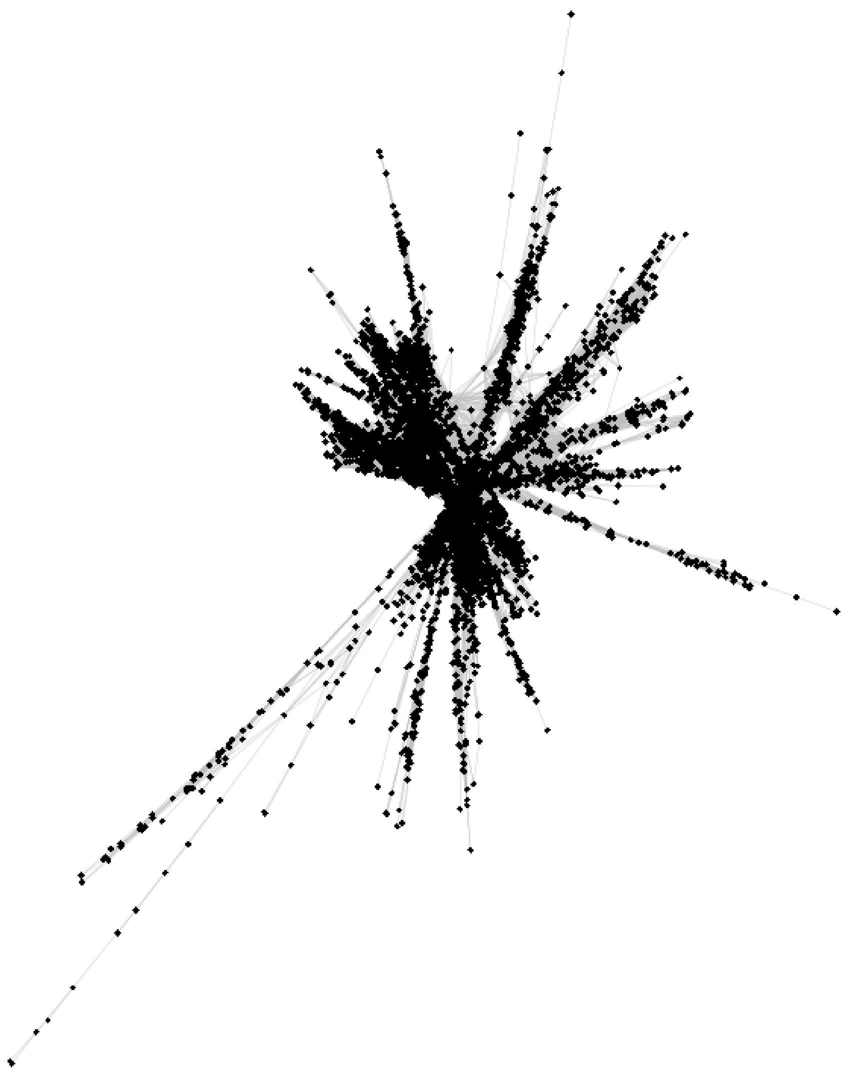

CL80

Number of reads: 5947
Number of pairs: 698182
Density: 0.03949
Diameter: NA
Mean edge weigth: 153.86
Max. degree: 1159

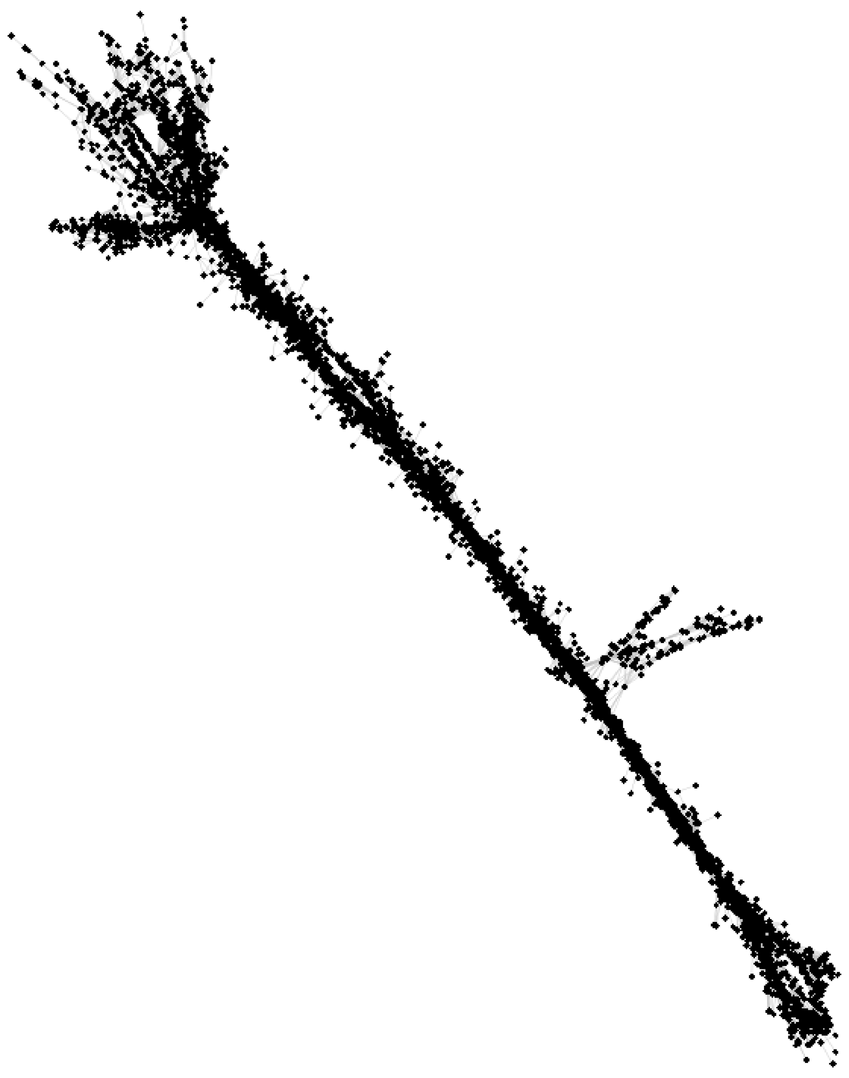

CL81

Number of reads: 5918
Number of pairs: 168353
Density: 0.009616
Diameter: NA
Mean edge weigth: 151.54
Max. degree: 234

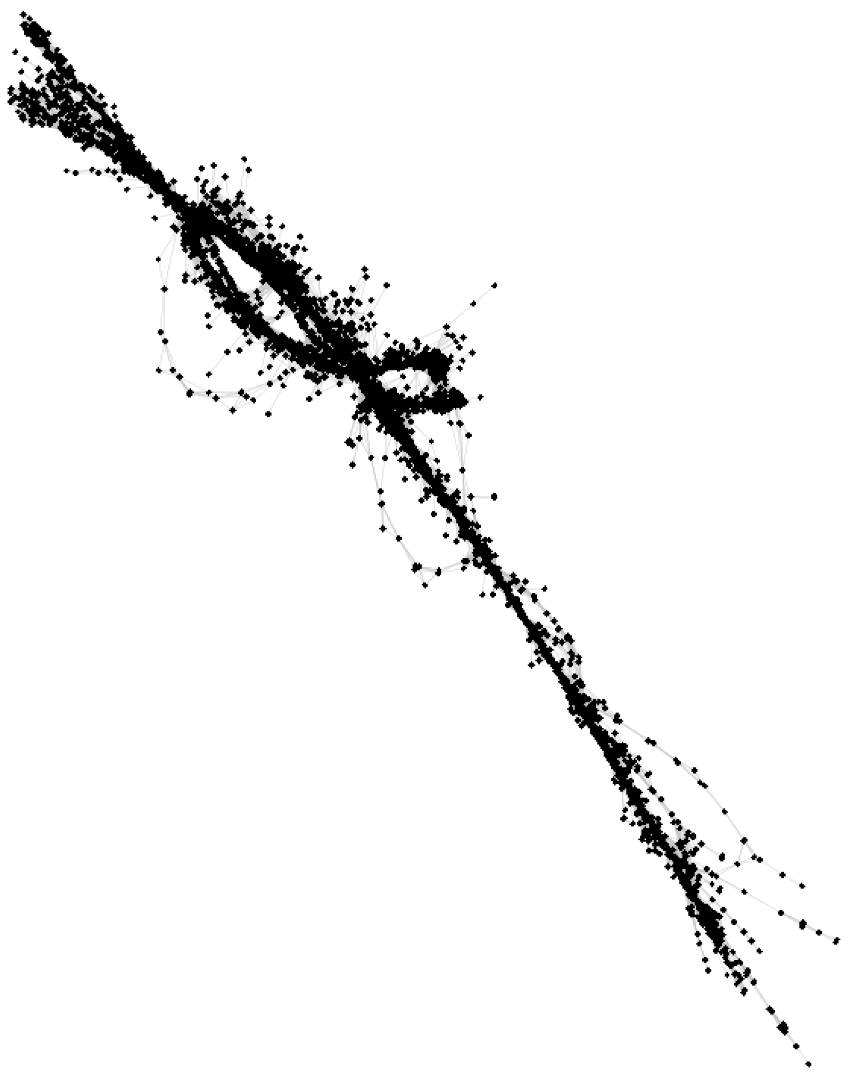

CL82

Number of reads: 5908
Number of pairs: 263881
Density: 0.01512
Diameter: NA
Mean edge weigth: 157.64
Max. degree: 337

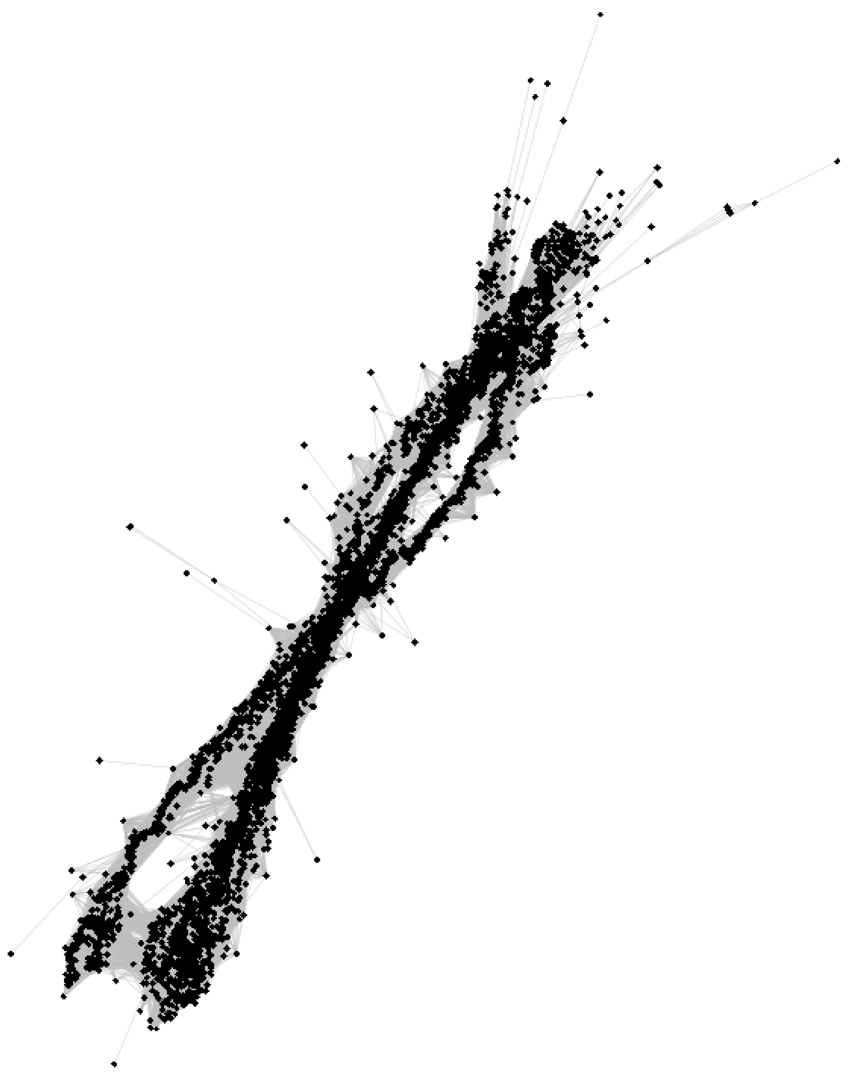

CL83

Number of reads: 5694
Number of pairs: 975254
Density: 0.06017
Diameter: NA
Mean edge weigth: 163.72
Max. degree: 815

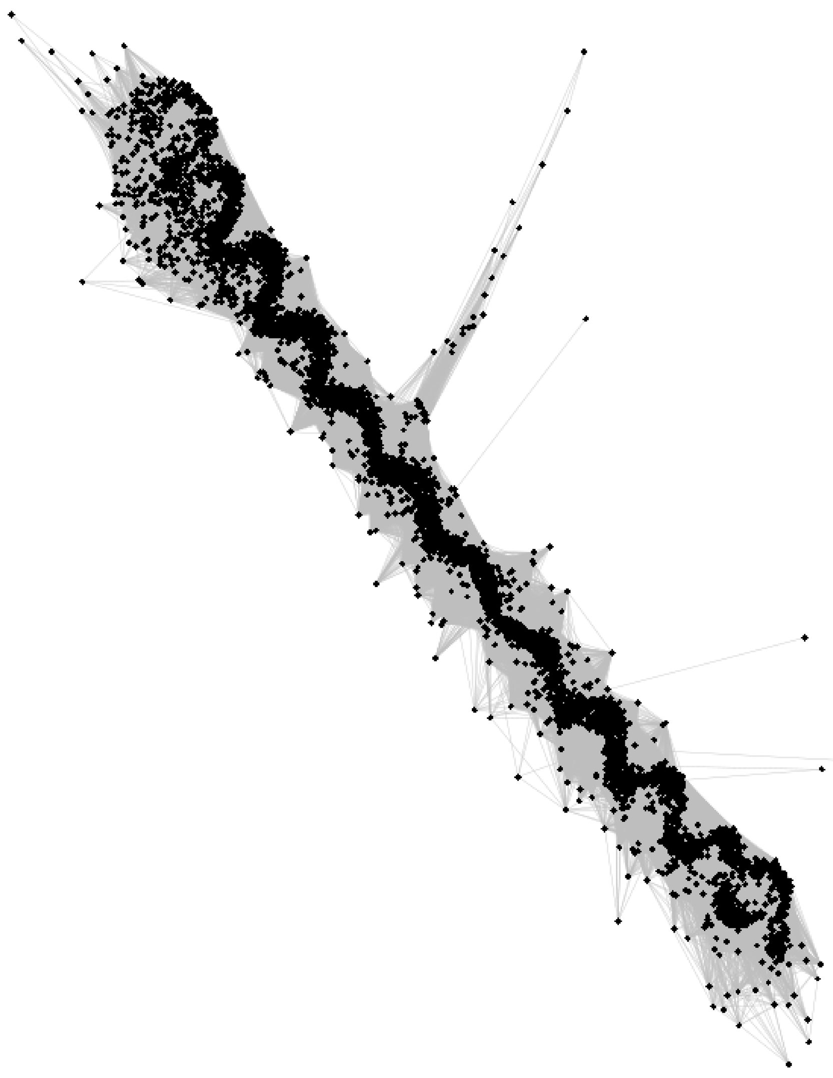

CL84

Number of reads: 5576
Number of pairs: 2492353
Density: 0.1604
Diameter: NA
Mean edge weigth: 175.85
Max. degree: 1187

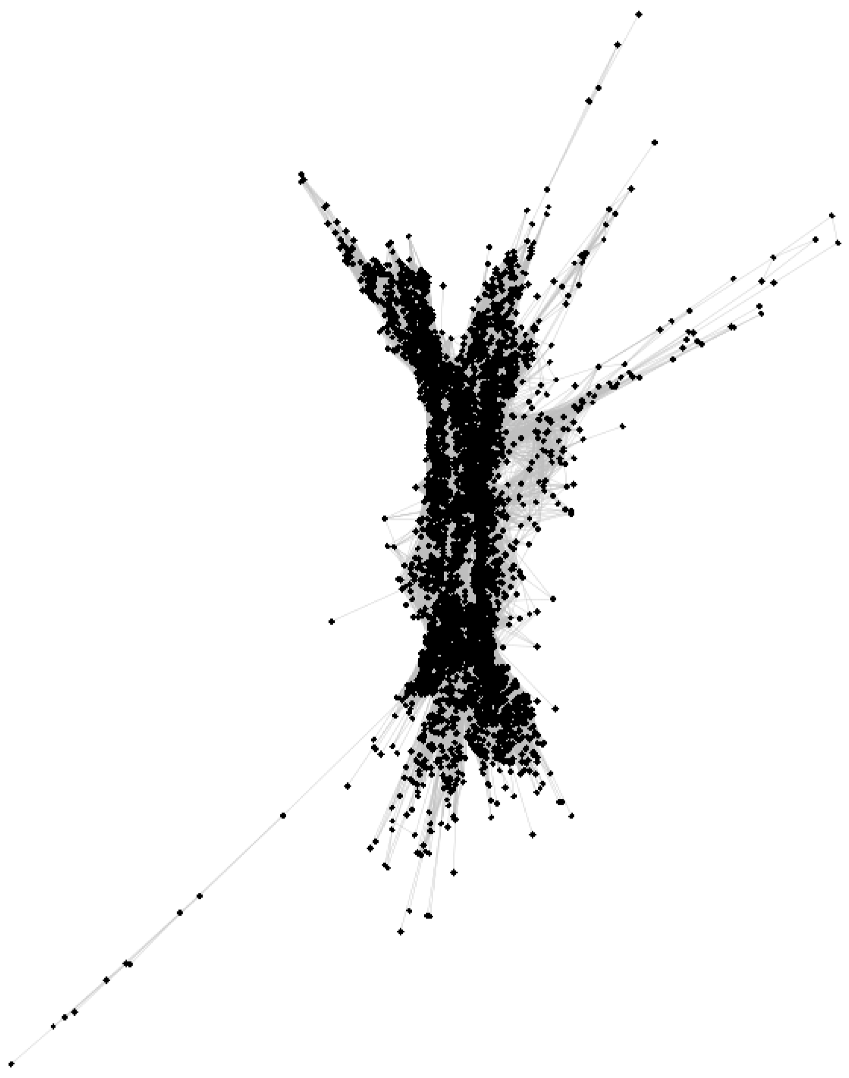

CL85

Number of reads: 5477
Number of pairs: 683406
Density: 0.04557
Diameter: NA
Mean edge weigth: 156.1
Max. degree: 975

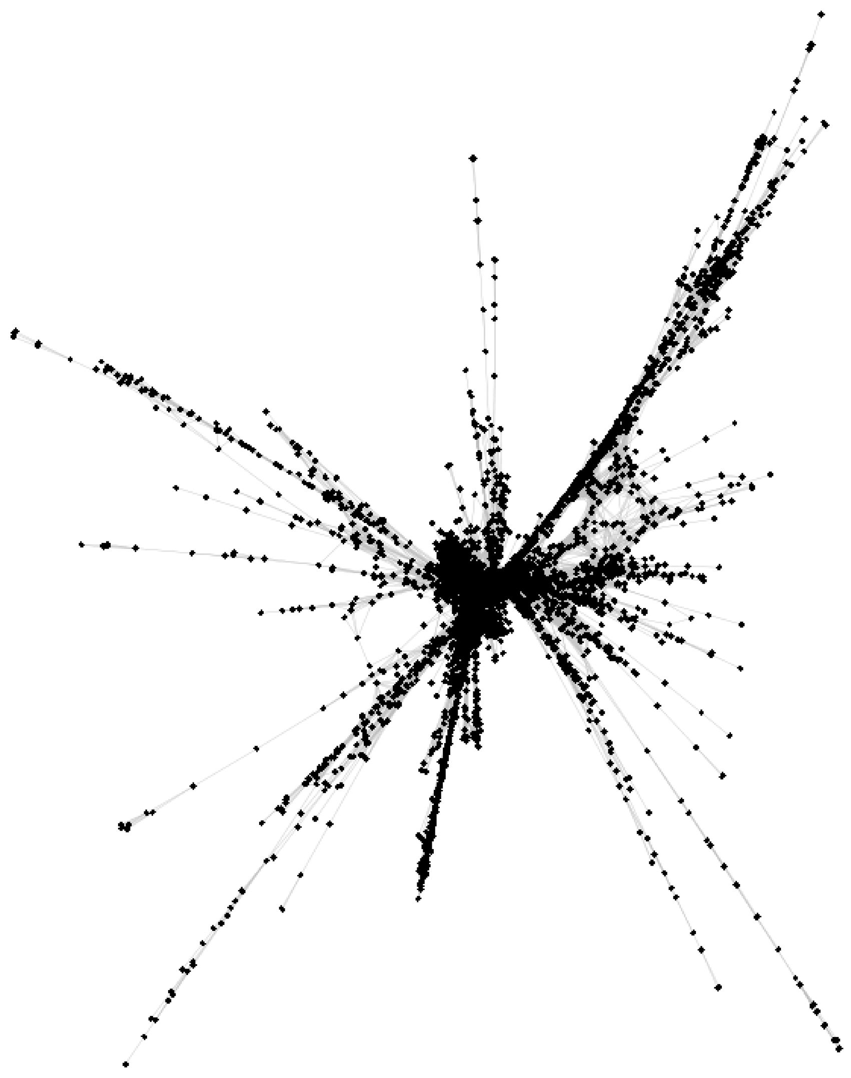

CL86

Number of reads: 5368
Number of pairs: 688024
Density: 0.04776
Diameter: NA
Mean edge weigth: 157.79
Max. degree: 1194

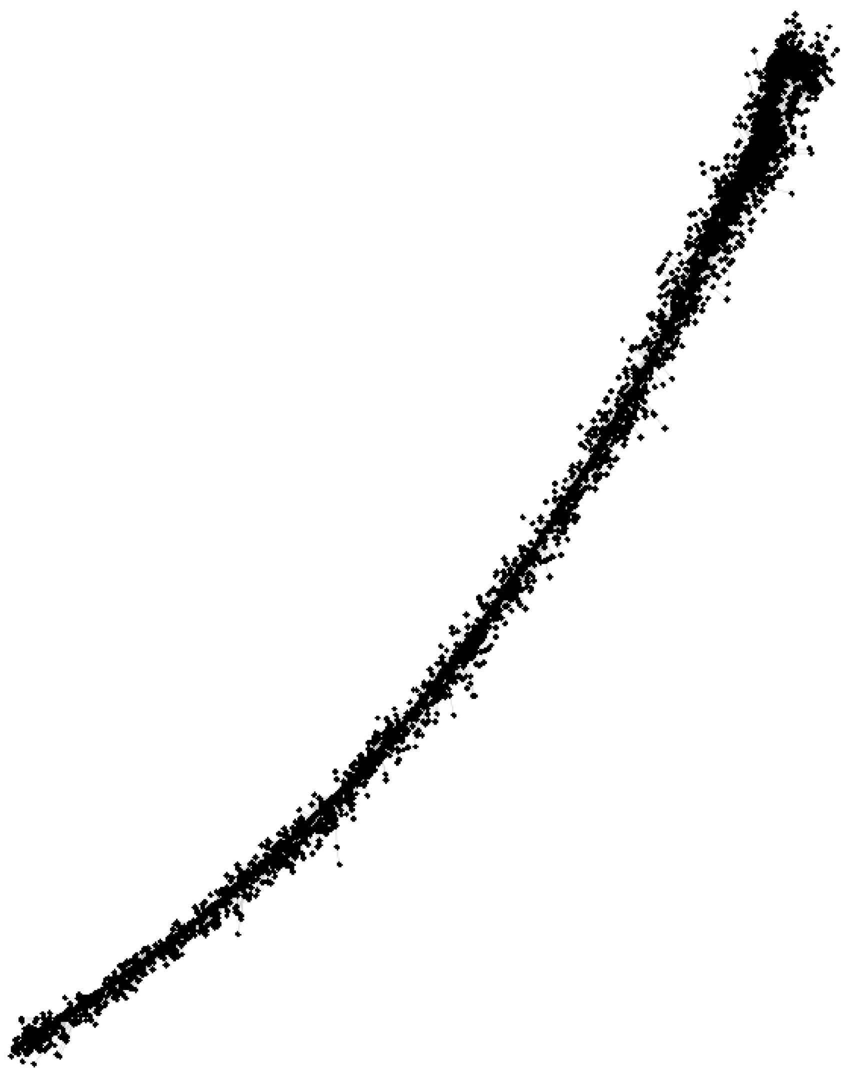

CL87

Number of reads: 5259
Number of pairs: 162272
Density: 0.01174
Diameter: NA
Mean edge weigth: 148.96
Max. degree: 264

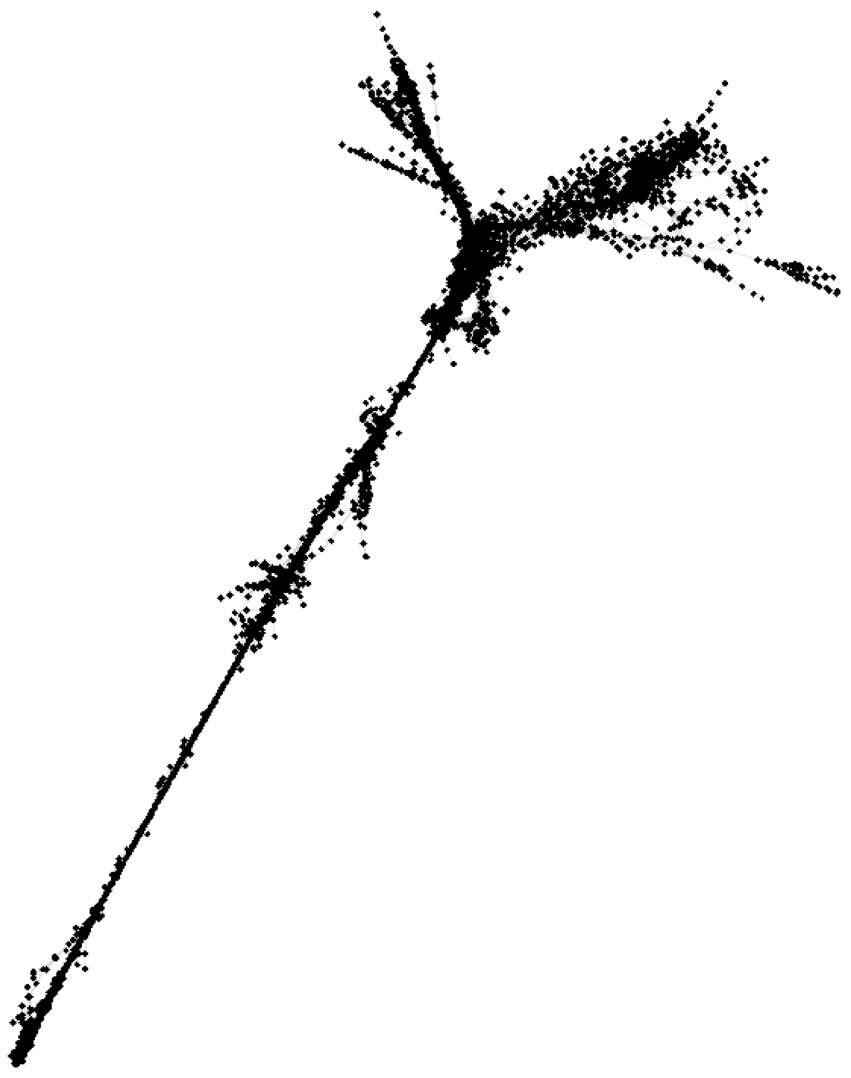

CL88

Number of reads: 4998
Number of pairs: 137966
Density: 0.01105
Diameter: NA
Mean edge weigth: 162.88
Max. degree: 212

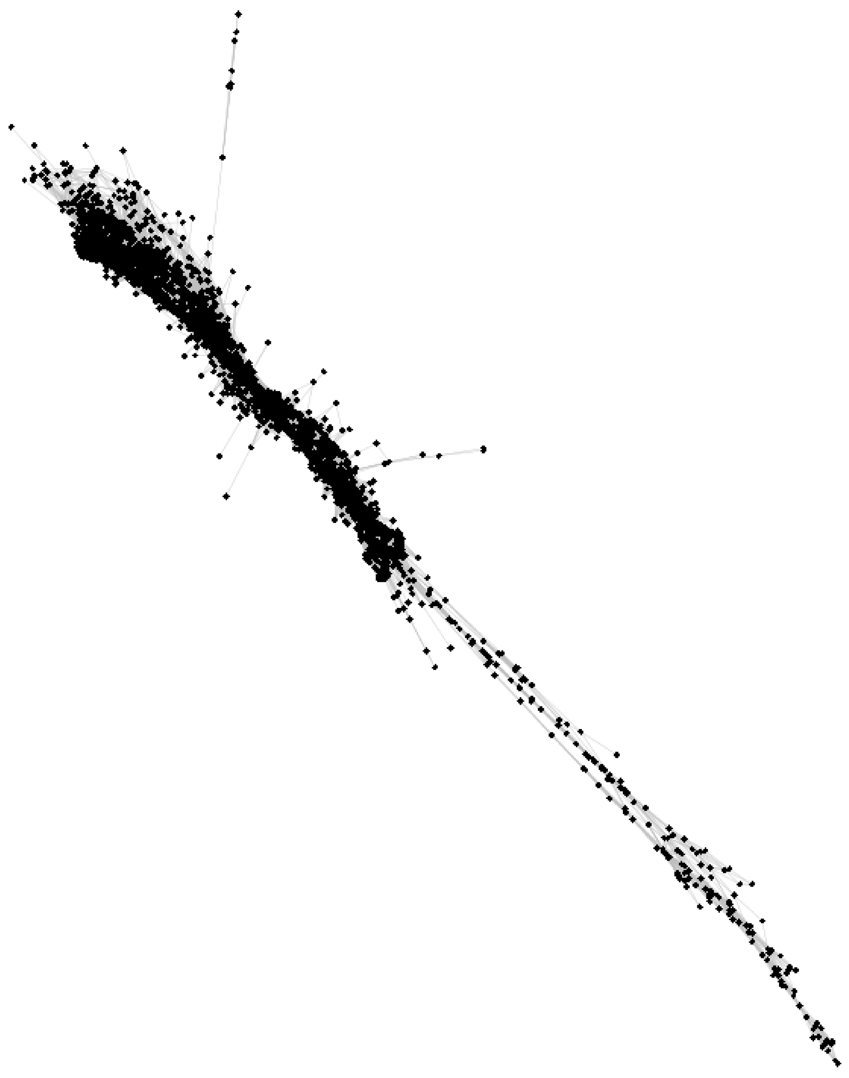

CL89

Number of reads: 4714
Number of pairs: 675891
Density: 0.06084
Diameter: NA
Mean edge weigth: 164.94
Max. degree: 563

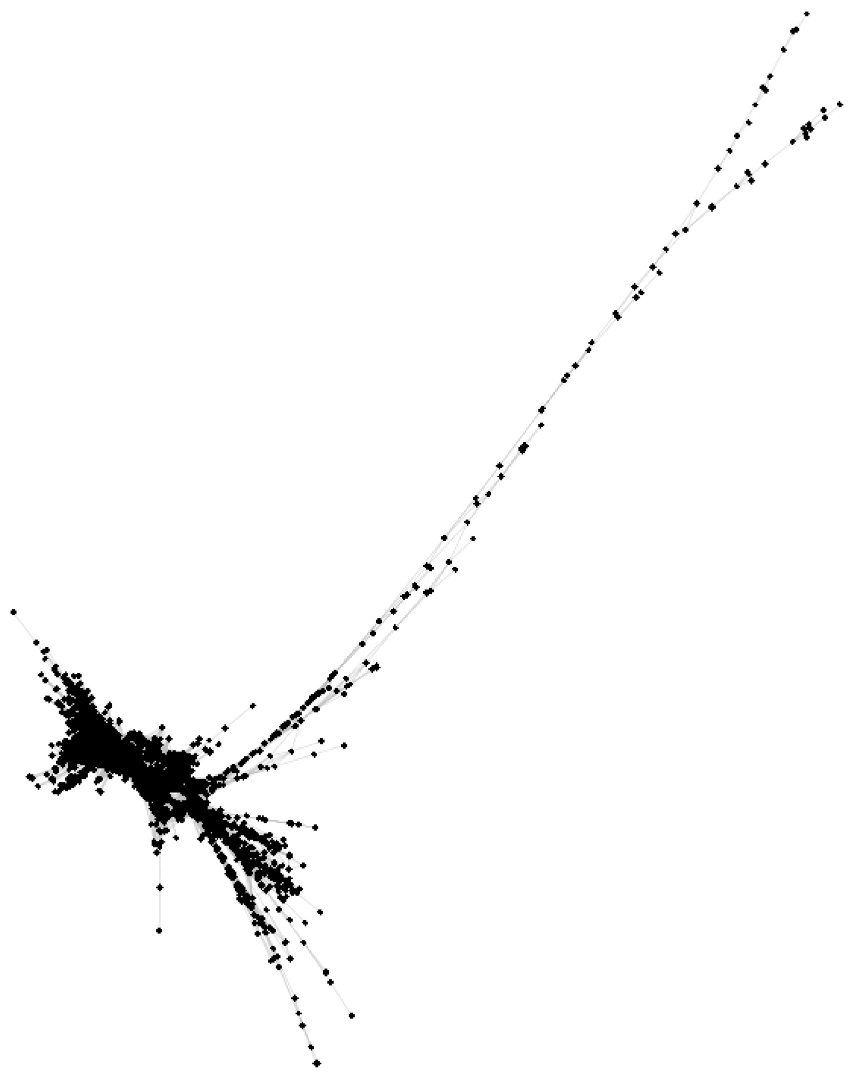

CL90

Number of reads: 4580
Number of pairs: 634290
Density: 0.06049
Diameter: NA
Mean edge weigth: 155.78
Max. degree: 843

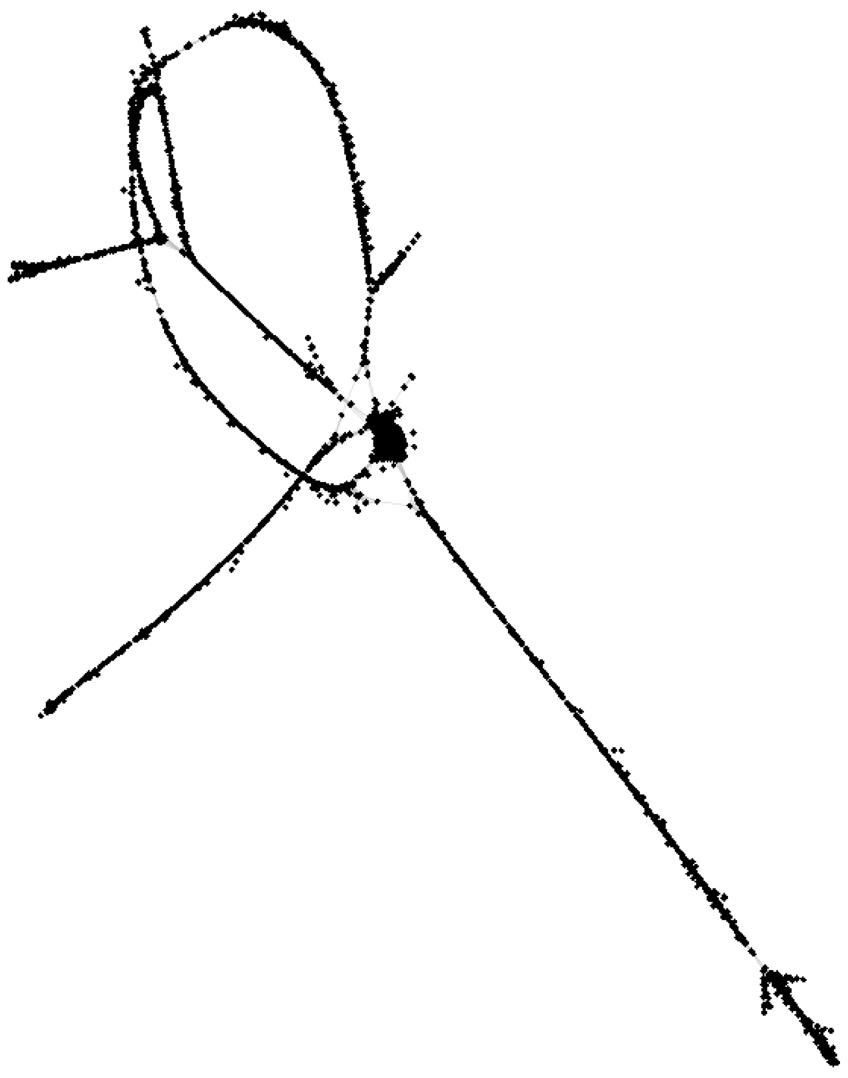

CL91

Number of reads: 4196
Number of pairs: 73585
Density: 0.008361
Diameter: NA
Mean edge weigth: 156.09
Max. degree: 580

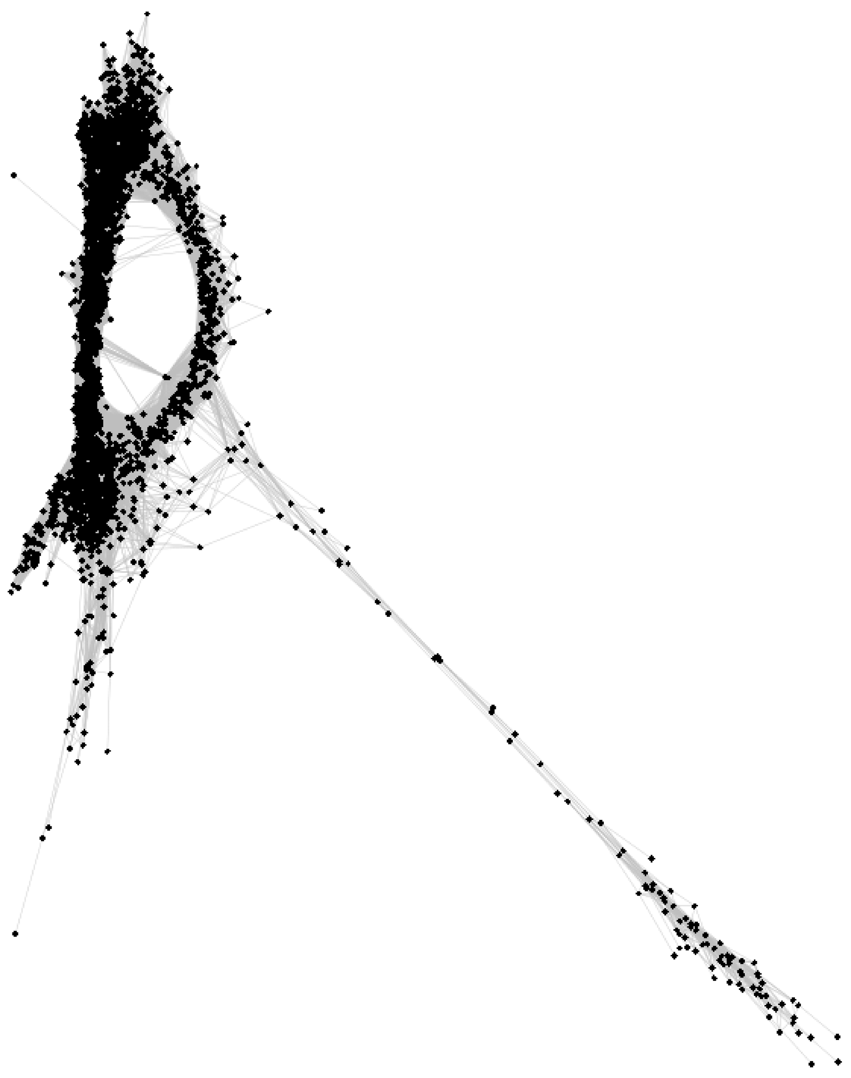

CL92

Number of reads: 4138
Number of pairs: 791986
Density: 0.09253
Diameter: NA
Mean edge weigth: 161.92
Max. degree: 789

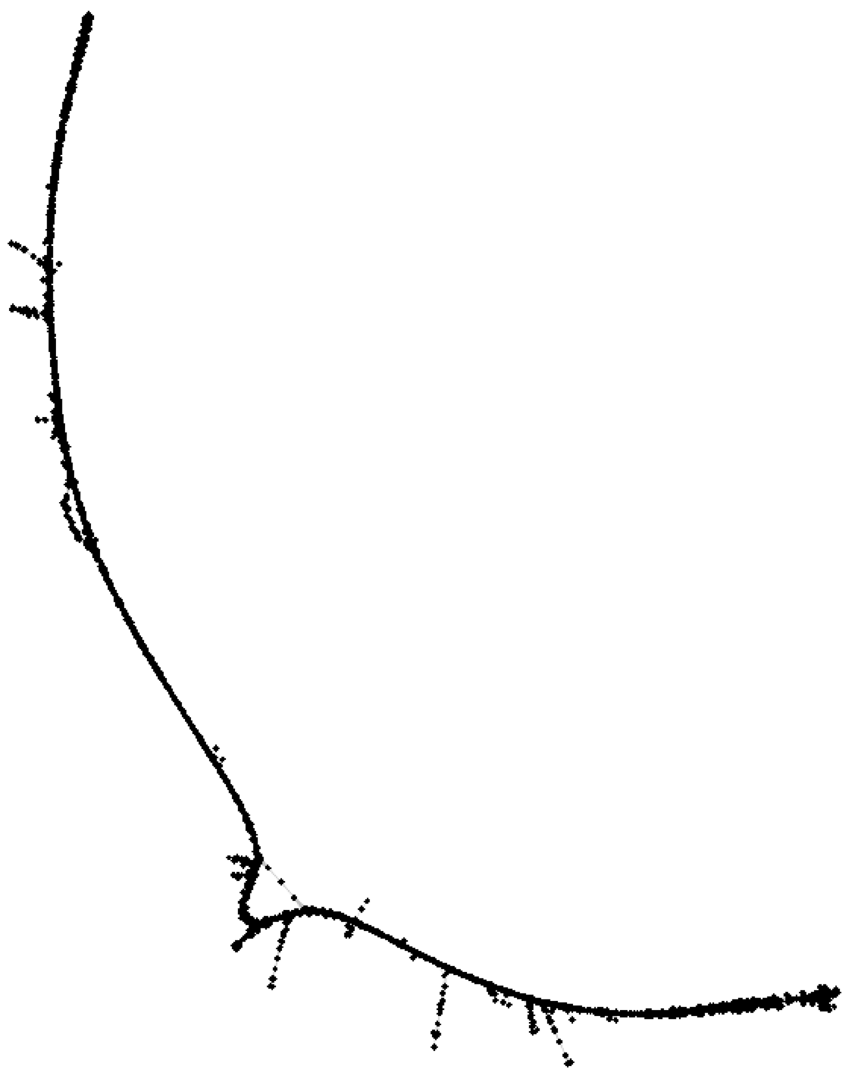

CL93

Number of reads: 4080
Number of pairs: 110423
Density: 0.01327
Diameter: NA
Mean edge weigth: 211.14
Max. degree: 87

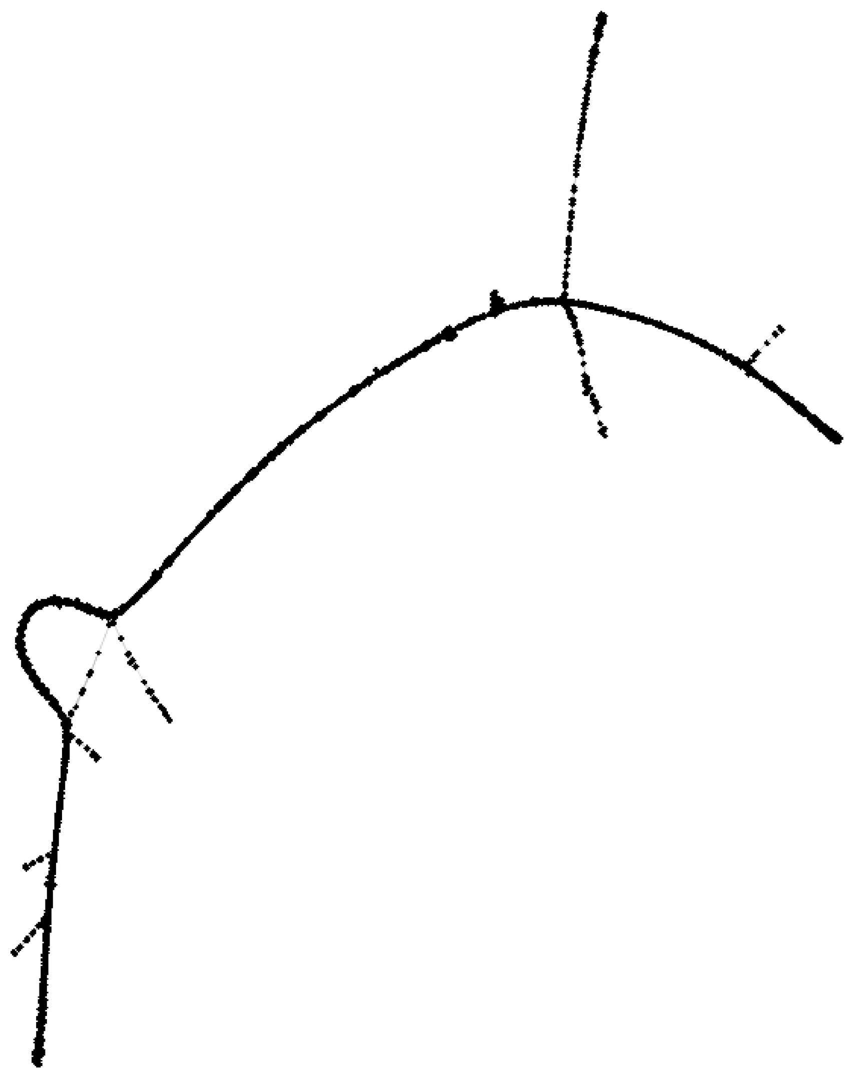

CL94

Number of reads: 4061
Number of pairs: 104654
Density: 0.01269
Diameter: NA
Mean edge weigth: 213.52
Max. degree: 92

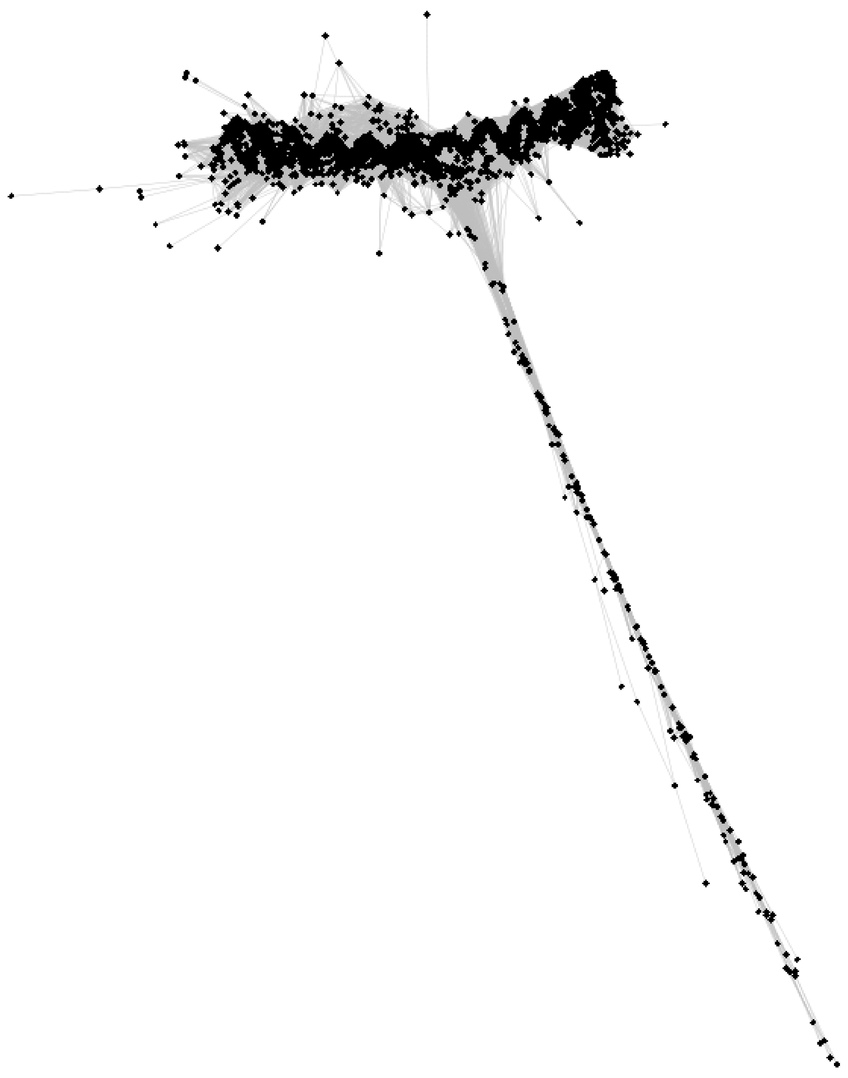

CL95

Number of reads: 3970
Number of pairs: 1150172
Density: 0.146
Diameter: NA
Mean edge weigth: 170.55
Max. degree: 843

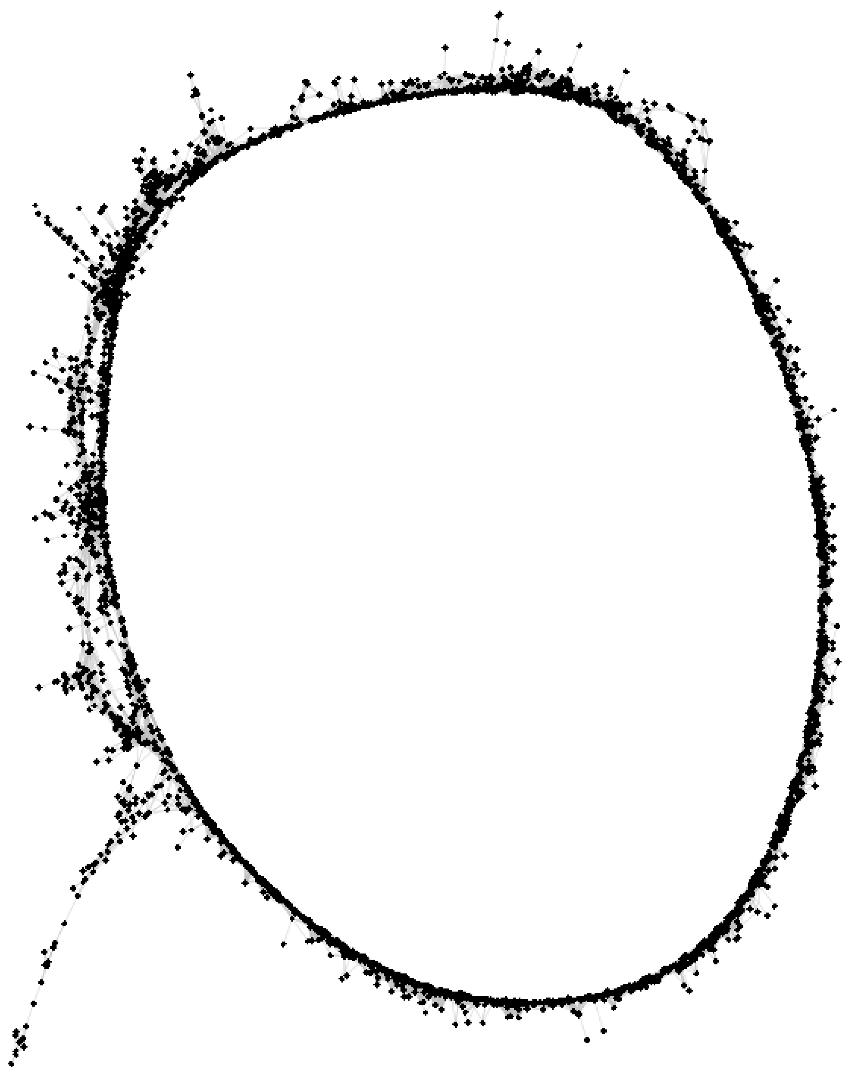

CL96

Number of reads: 3889
Number of pairs: 99597
Density: 0.01317
Diameter: NA
Mean edge weigth: 166.47
Max. degree: 132

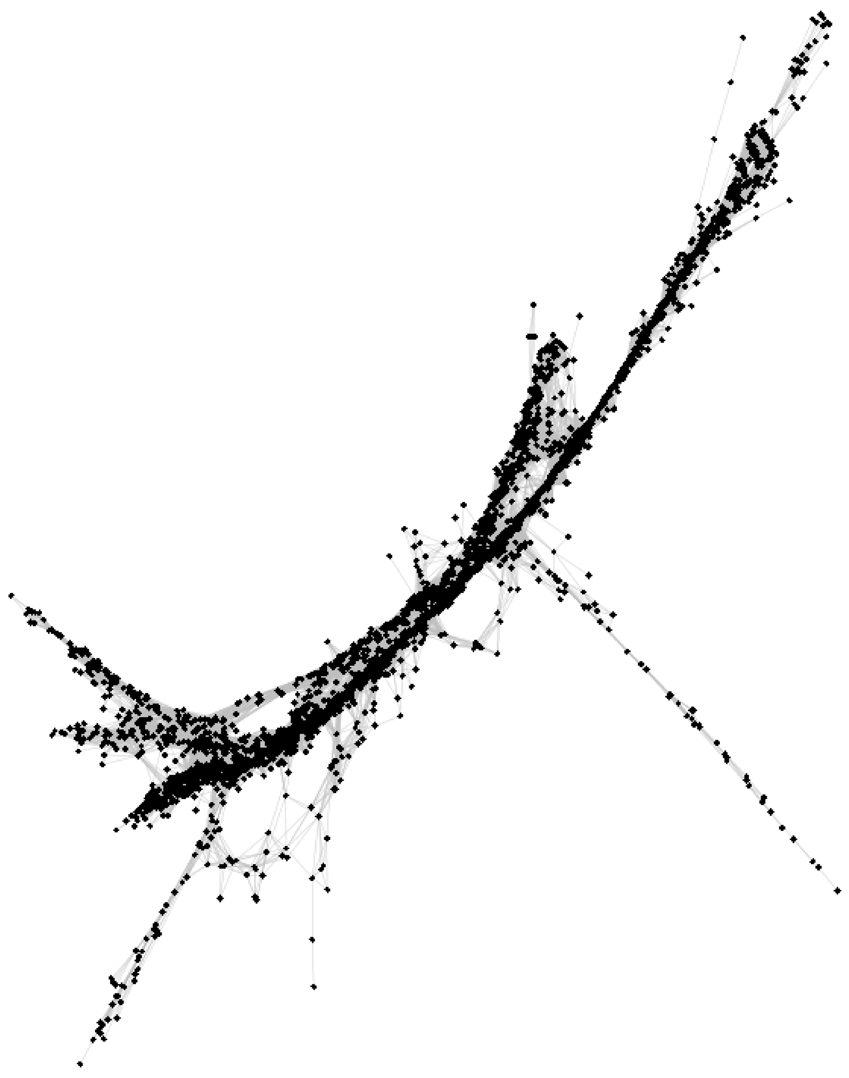

CL97

Number of reads: 3767
Number of pairs: 246914
Density: 0.03481
Diameter: NA
Mean edge weigth: 164.24
Max. degree: 305

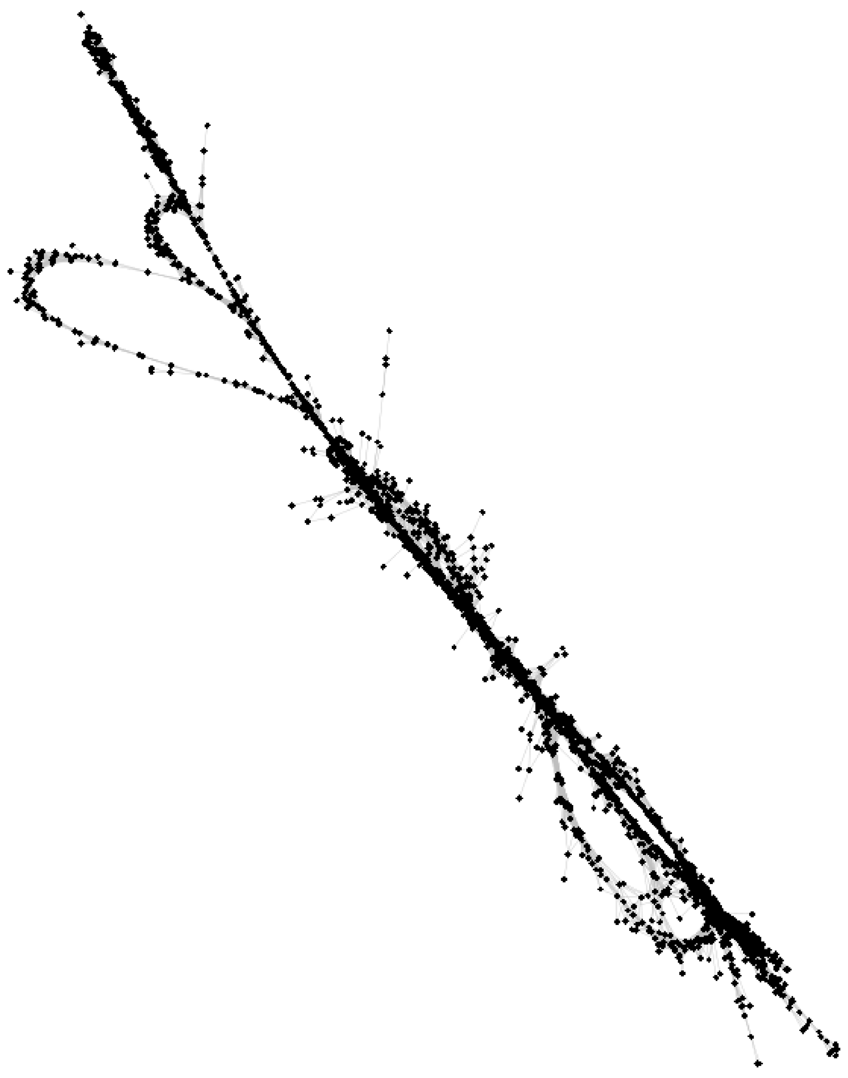

CL98

Number of reads: 3389
Number of pairs: 157039
Density: 0.02735
Diameter: NA
Mean edge weigth: 161.76
Max. degree: 222

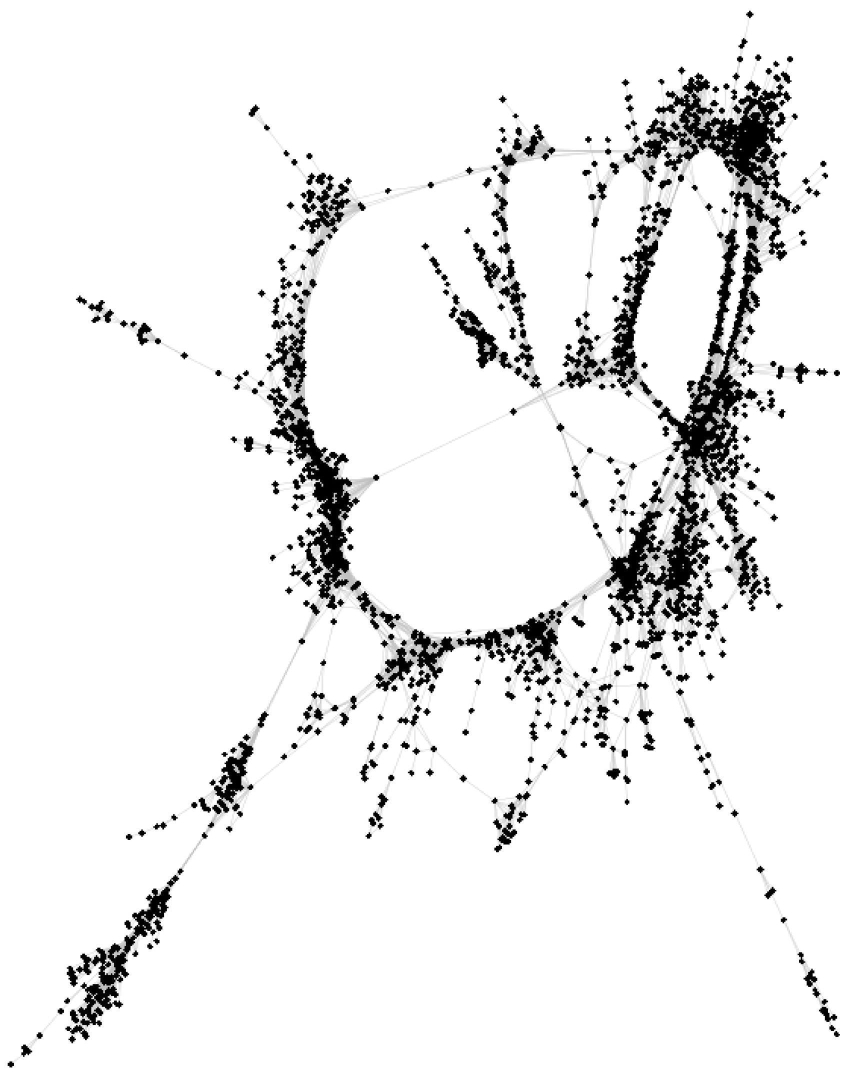

CL99

Number of reads: 3388
Number of pairs: 32863
Density: 0.005728
Diameter: NA
Mean edge weigth: 149.79
Max. degree: 100

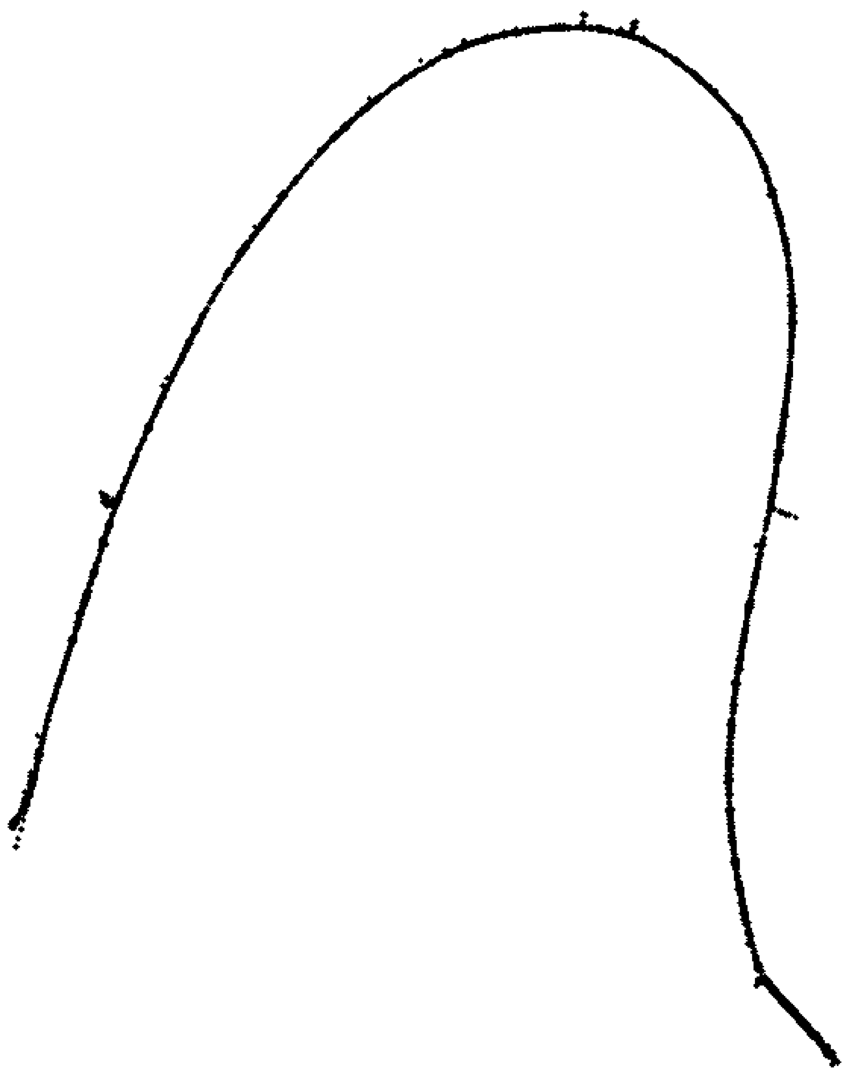

CL100

Number of reads: 3370
Number of pairs: 48269
Density: 0.008503
Diameter: NA
Mean edge weigth: 214.23
Max. degree: 48

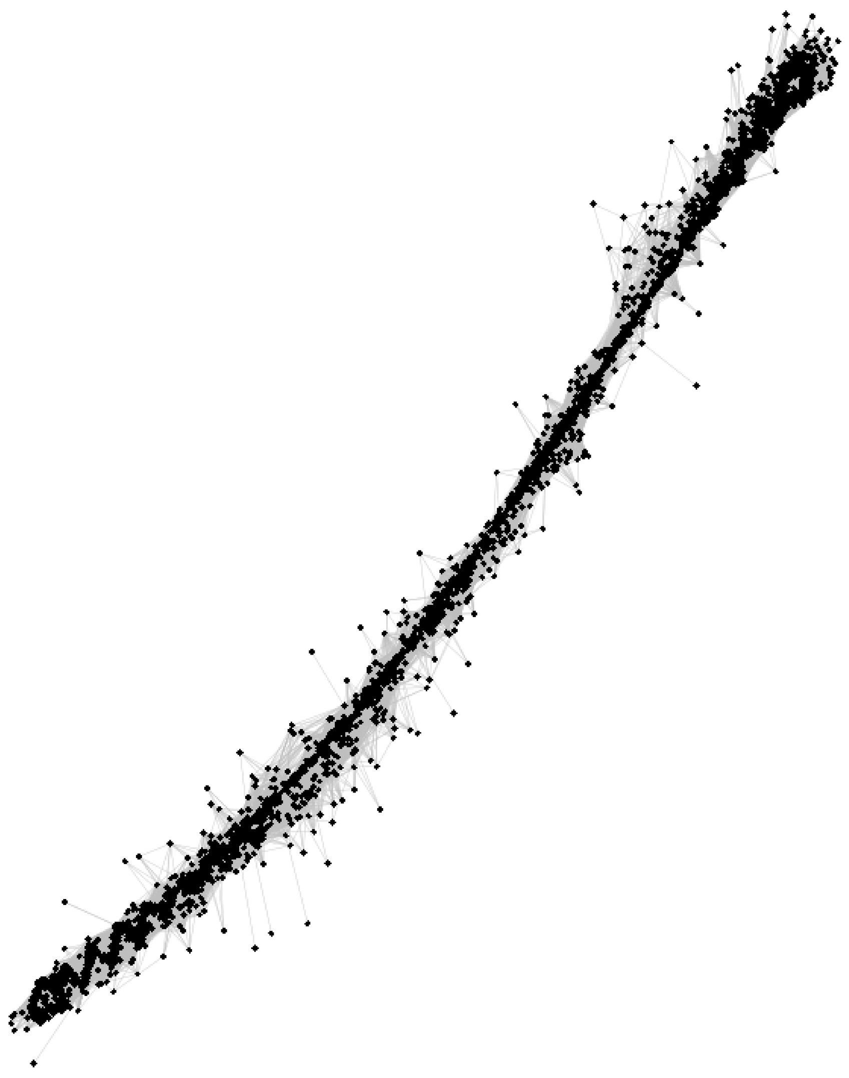

CL101

Number of reads: 3249
Number of pairs: 298541
Density: 0.05658
Diameter: NA
Mean edge weigth: 163.57
Max. degree: 327

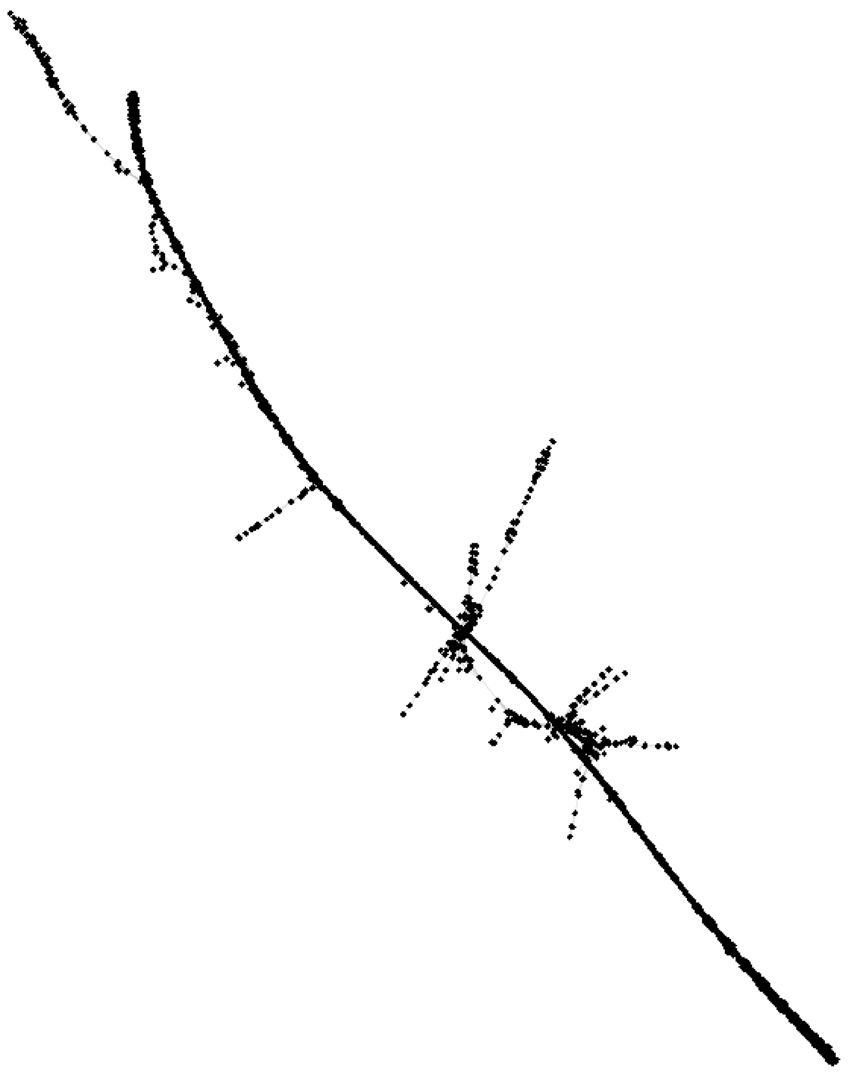

CL102

Number of reads: 3075
Number of pairs: 83174
Density: 0.0176
Diameter: NA
Mean edge weigth: 212.38
Max. degree: 91

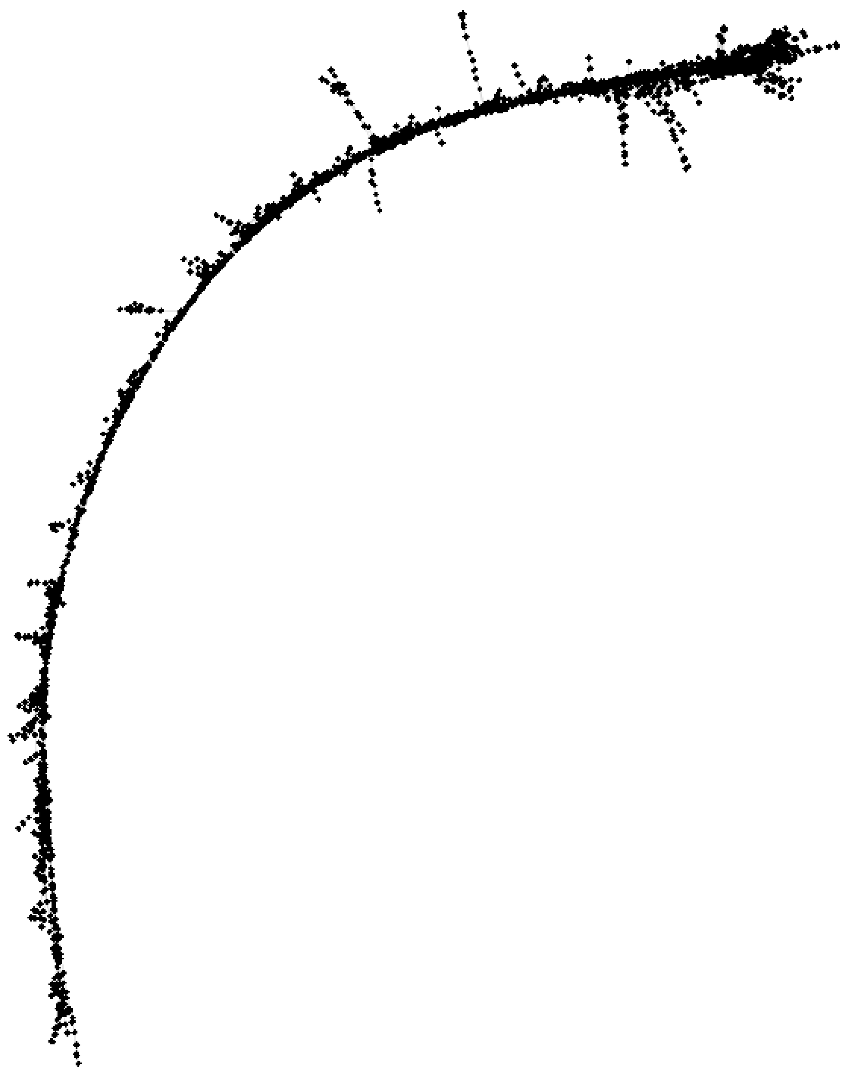

CL103

Number of reads: 2759
Number of pairs: 43696
Density: 0.01148
Diameter: NA
Mean edge weigth: 159.75
Max. degree: 96

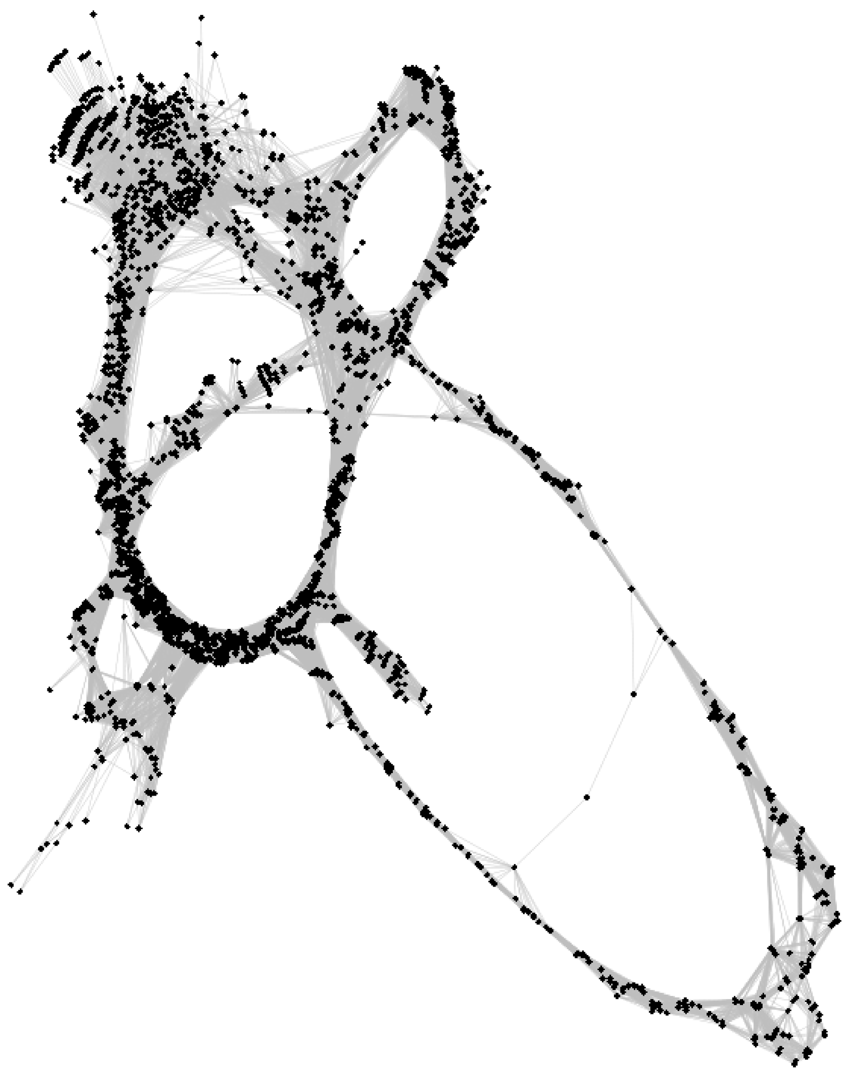

CL104

Number of reads: 2749
Number of pairs: 135405
Density: 0.03585
Diameter: NA
Mean edge weigth: 197.43
Max. degree: 383

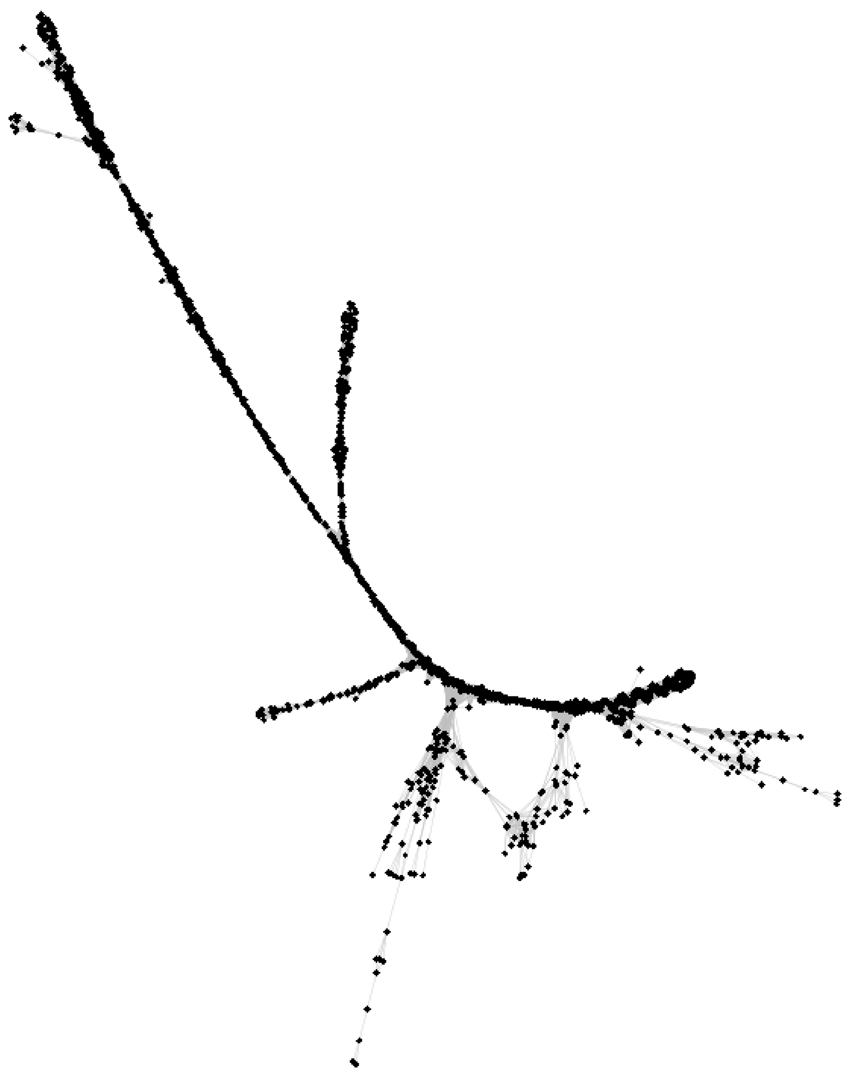

CL105

Number of reads: 2307
Number of pairs: 105684
Density: 0.03973
Diameter: NA
Mean edge weigth: 208.5
Max. degree: 180

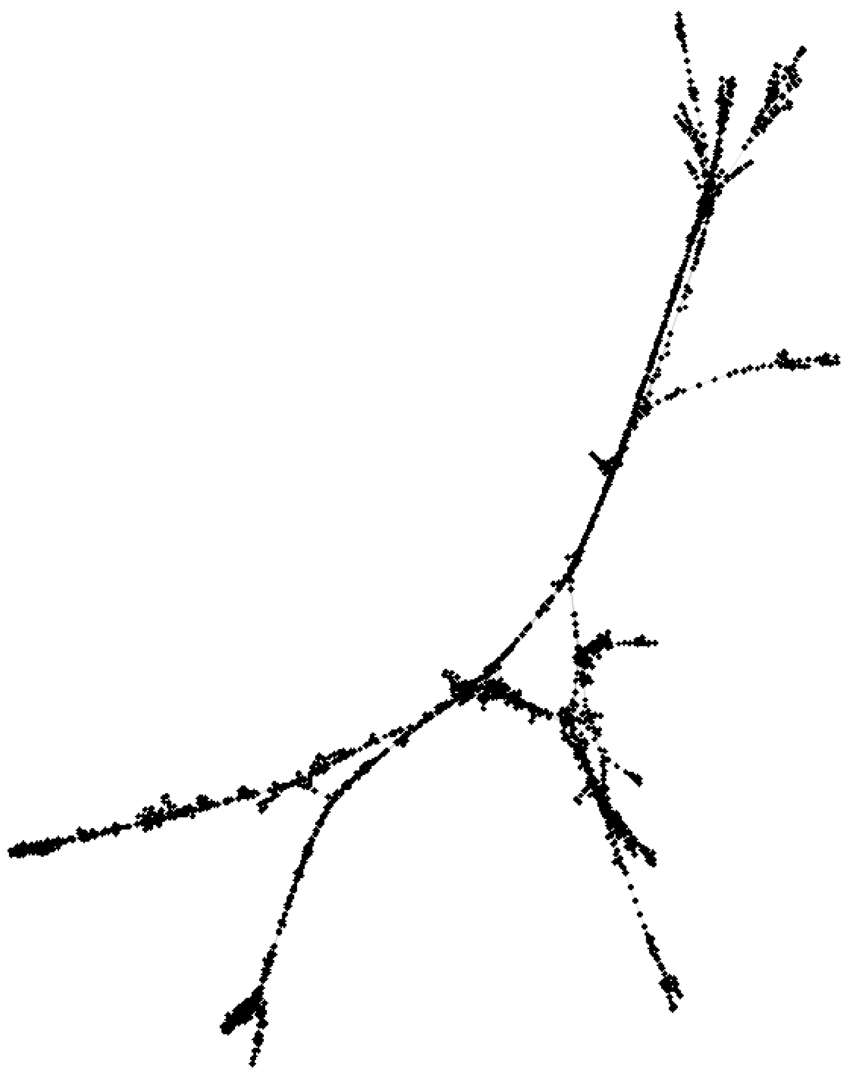

CL106

Number of reads: 2253
Number of pairs: 17337
Density: 0.006834
Diameter: NA
Mean edge weigth: 164.84
Max. degree: 72

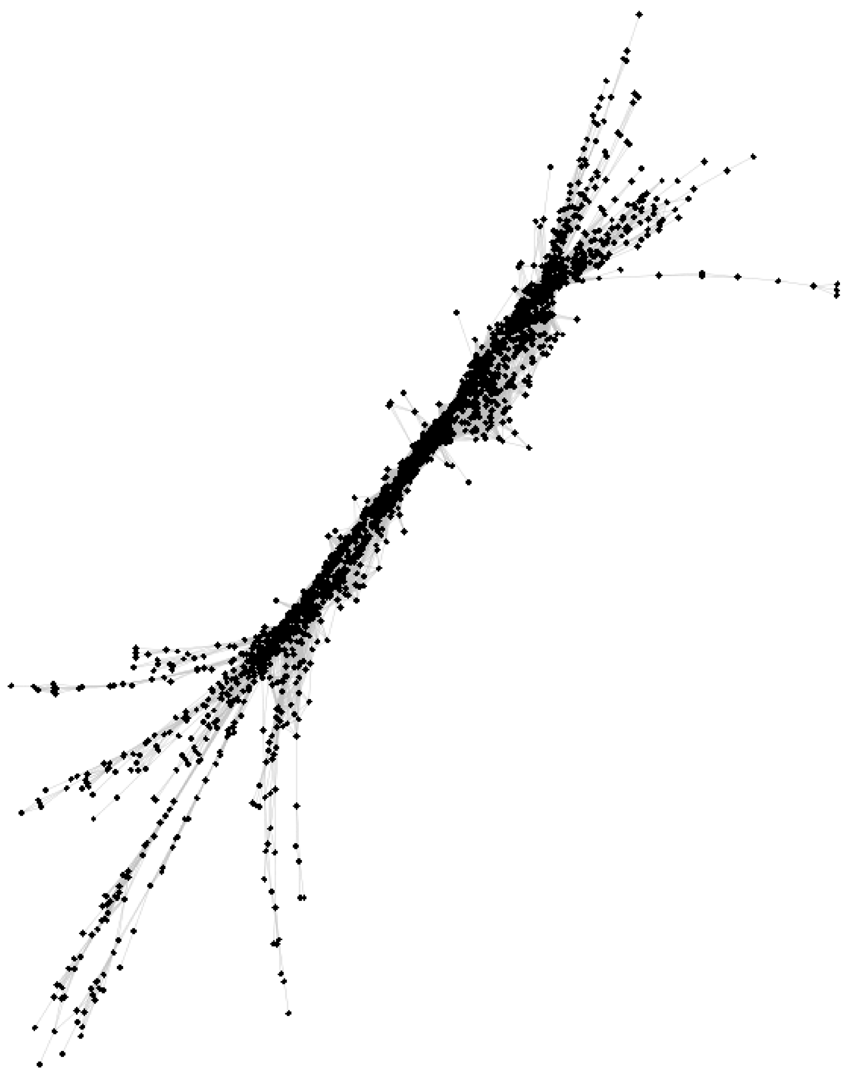

CL107

Number of reads: 2252
Number of pairs: 110889
Density: 0.04375
Diameter: NA
Mean edge weigth: 158.12
Max. degree: 268

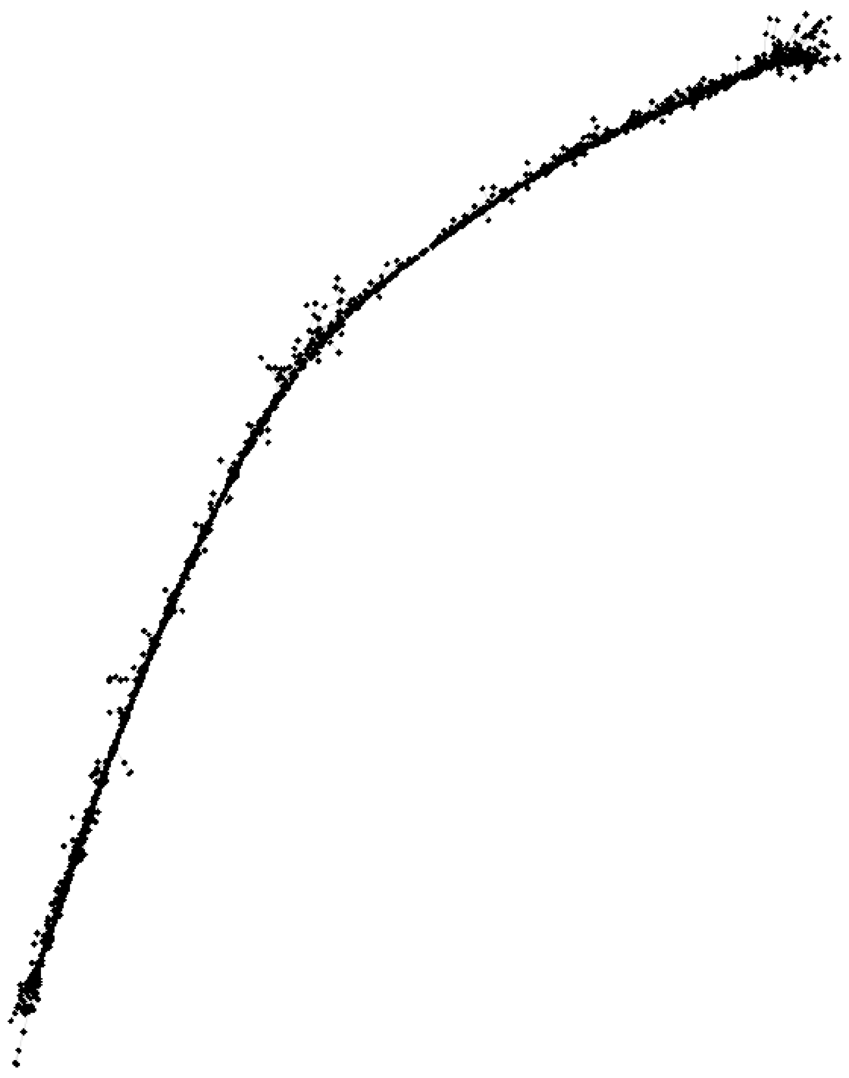

CL108

Number of reads: 2155
Number of pairs: 47036
Density: 0.02027
Diameter: NA
Mean edge weigth: 175
Max. degree: 98

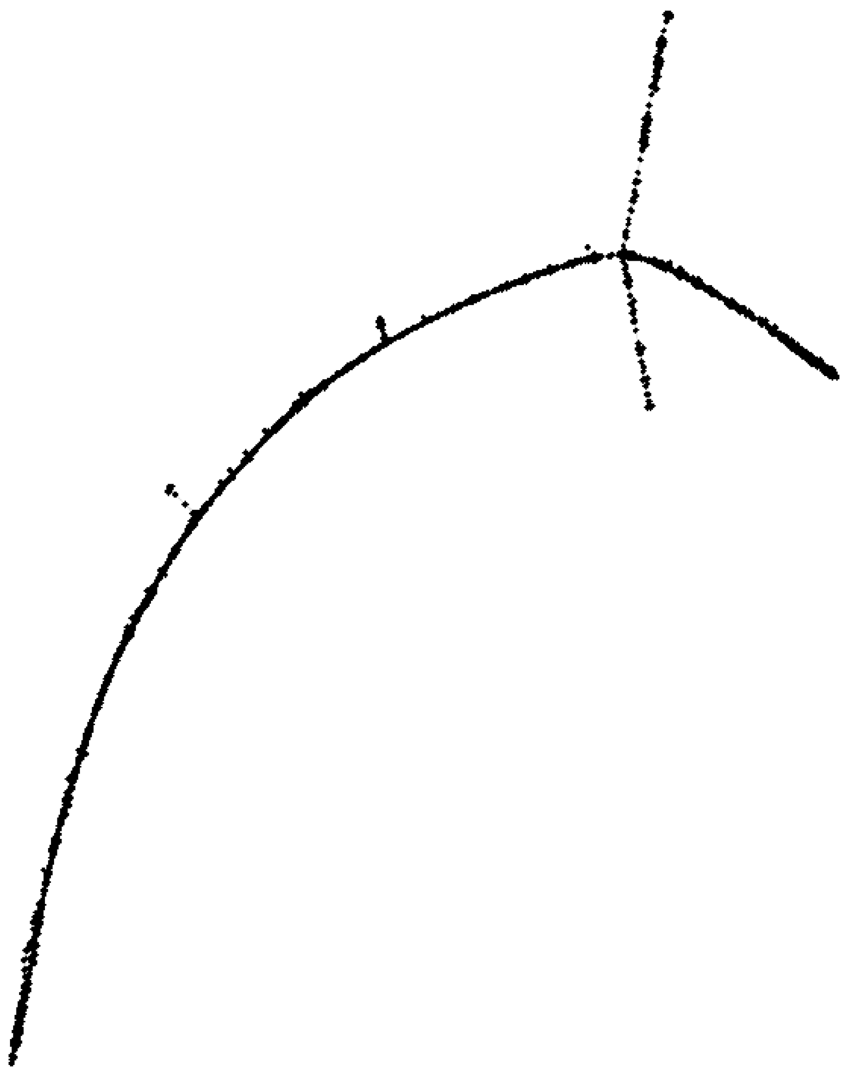

CL109

Number of reads: 2111
Number of pairs: 30967
Density: 0.0139
Diameter: NA
Mean edge weigth: 208.56
Max. degree: 49

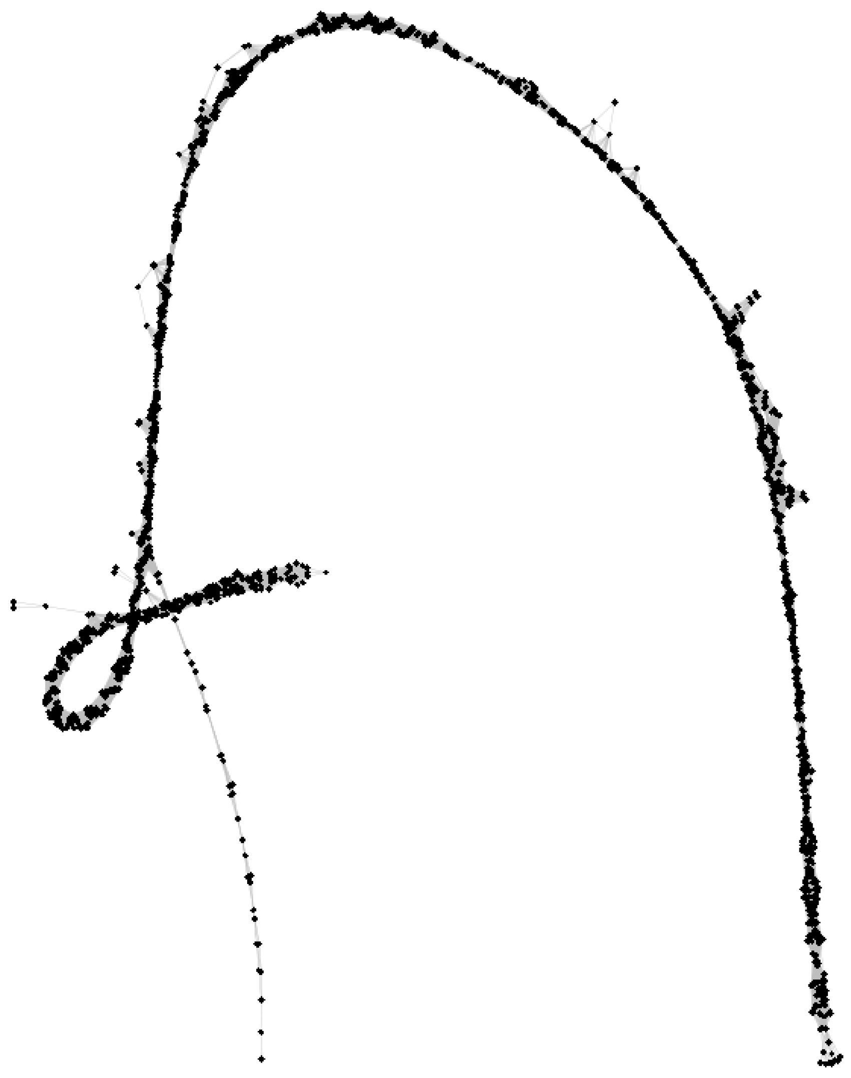

CL110

Number of reads: 1937
Number of pairs: 51251
Density: 0.02733
Diameter: NA
Mean edge weigth: 212.16
Max. degree: 91

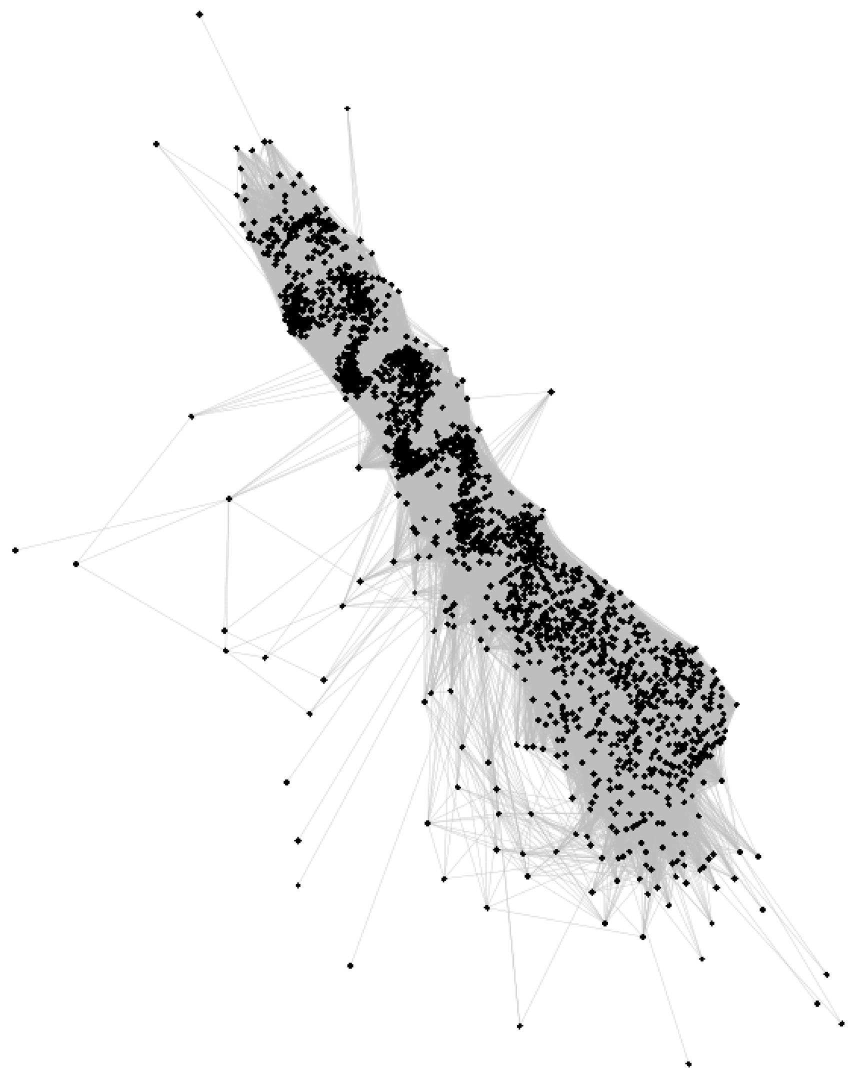

CL111

Number of reads: 1906
Number of pairs: 413824
Density: 0.2279
Diameter: NA
Mean edge weigth: 171.7
Max. degree: 652

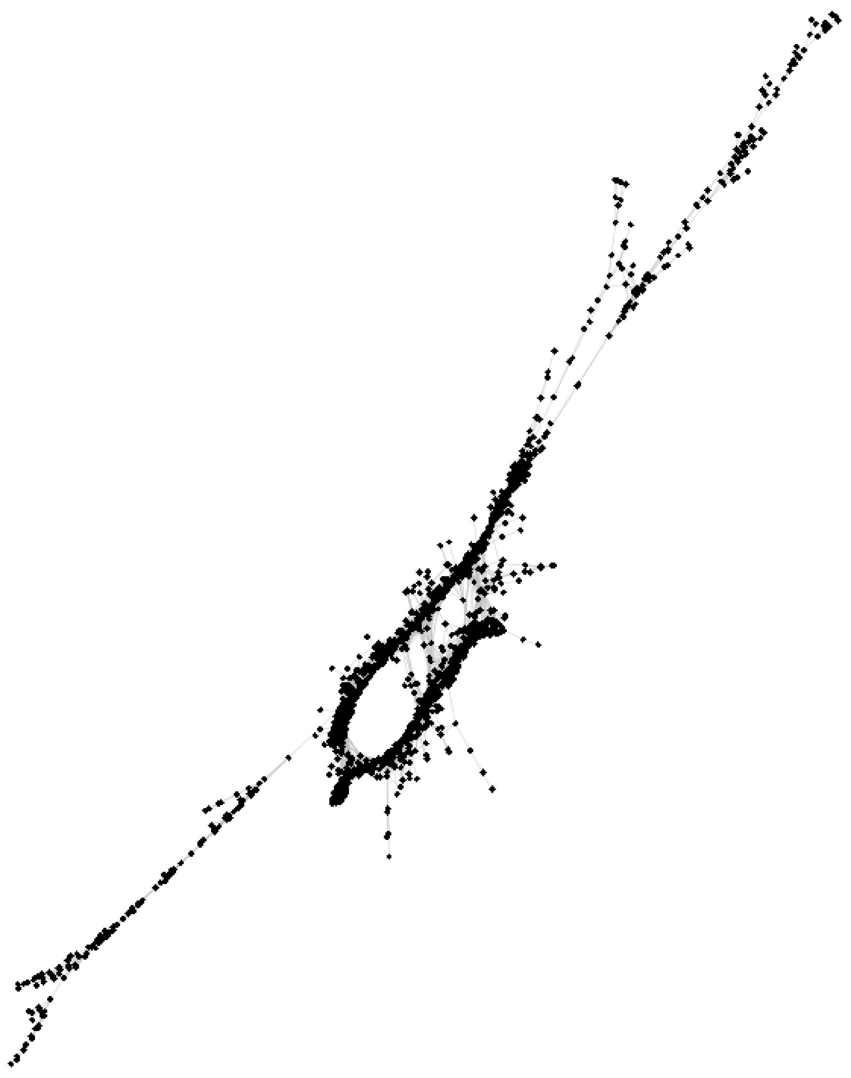

CL112

Number of reads: 1873
Number of pairs: 47592
Density: 0.02715
Diameter: NA
Mean edge weigth: 165.29
Max. degree: 114

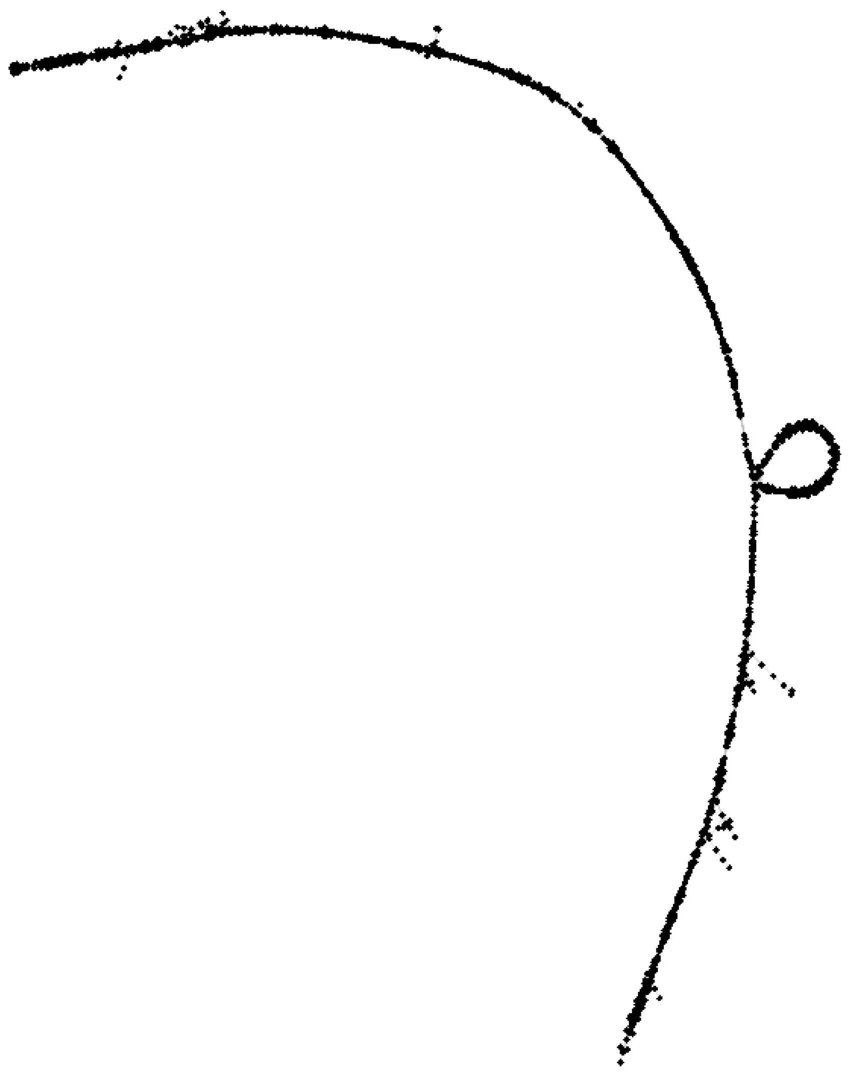

CL113

Number of reads: 1851
Number of pairs: 25733
Density: 0.01503
Diameter: NA
Mean edge weigth: 213.46
Max. degree: 49

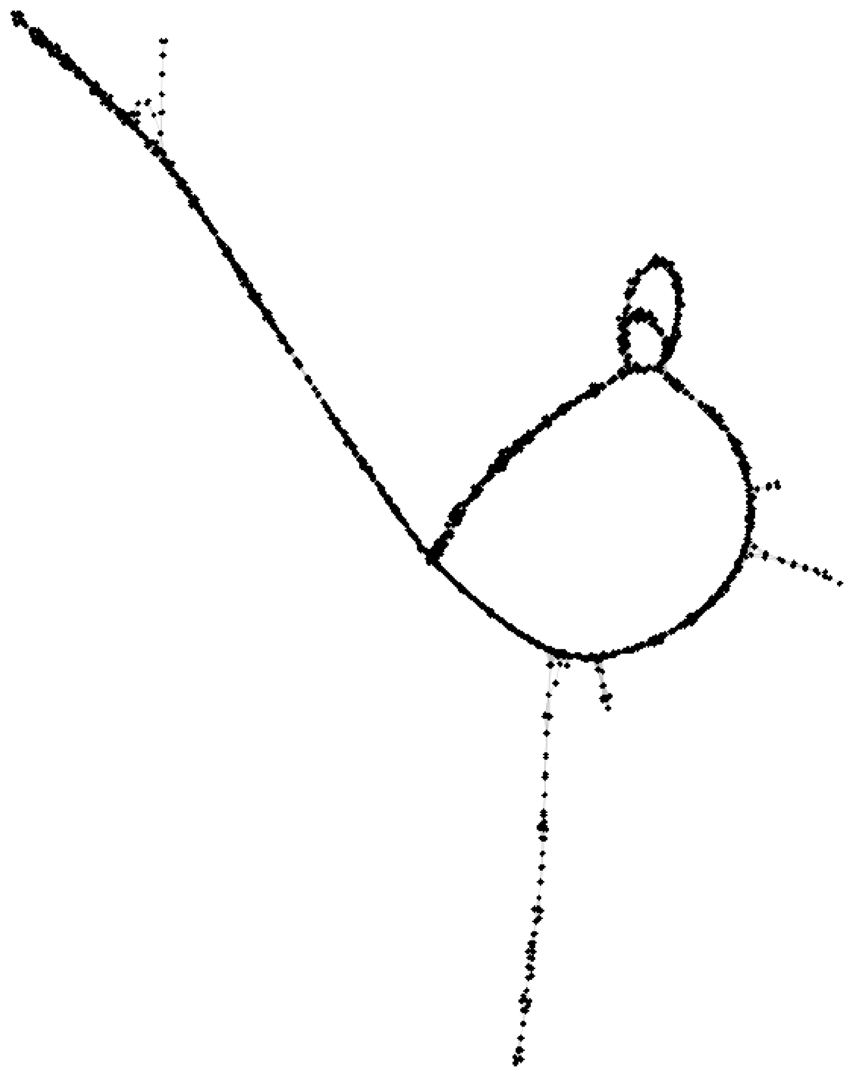

CL114

Number of reads: 1837
Number of pairs: 27750
Density: 0.01646
Diameter: NA
Mean edge weigth: 212.12
Max. degree: 70

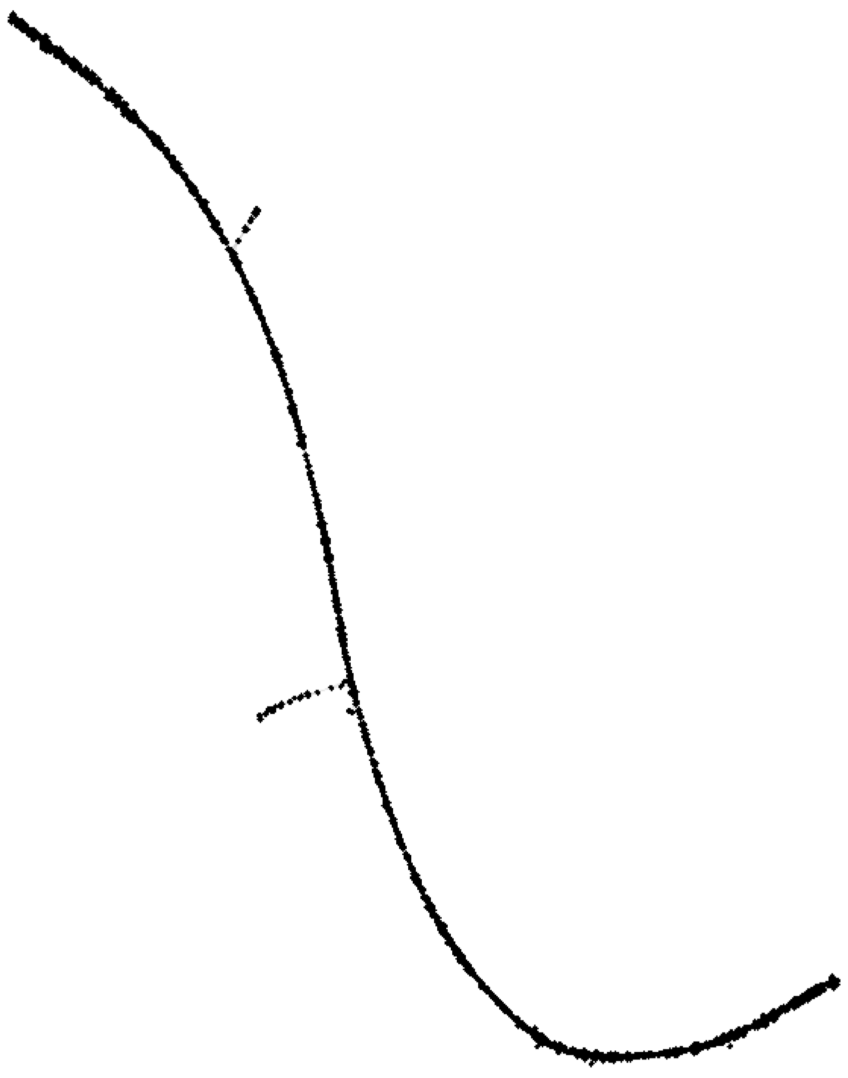

CL115

Number of reads: 1812
Number of pairs: 26439
Density: 0.01611
Diameter: NA
Mean edge weigth: 214.36
Max. degree: 45

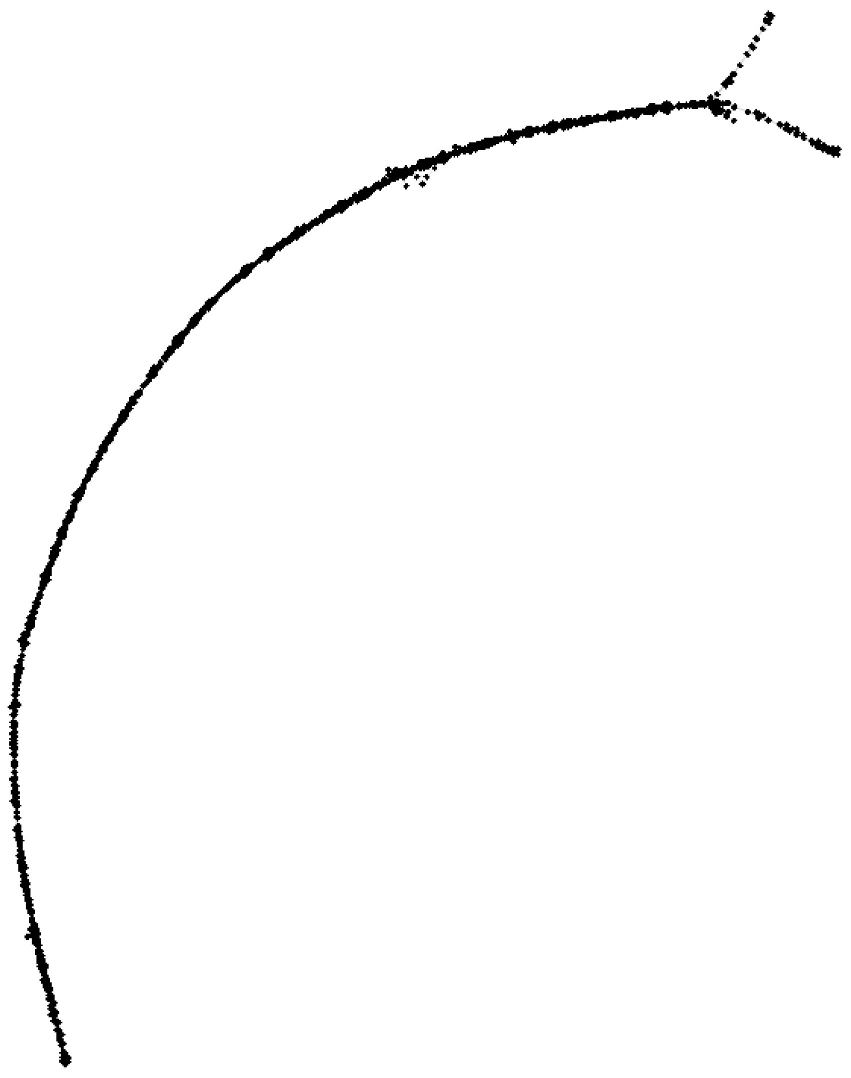

CL116

Number of reads: 1761
Number of pairs: 24715
Density: 0.01595
Diameter: NA
Mean edge weigth: 214.01
Max. degree: 49

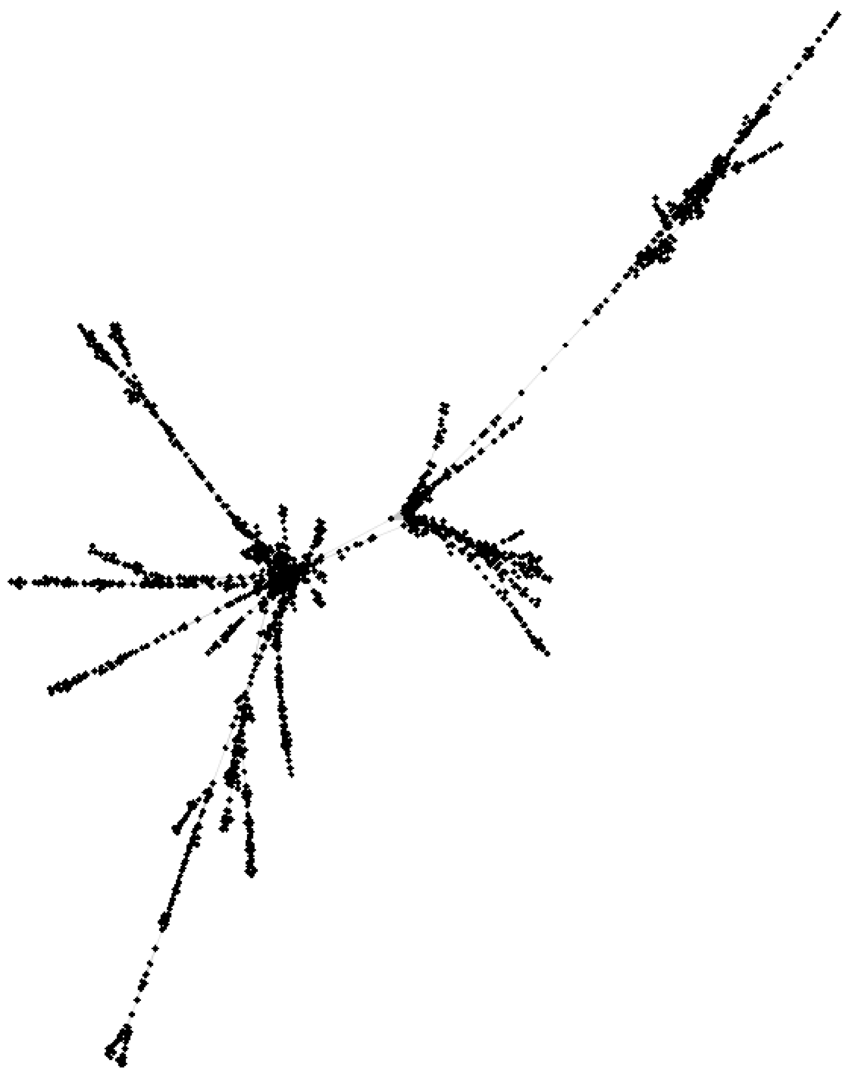

CL117

Number of reads: 1713
Number of pairs: 8414
Density: 0.005738
Diameter: NA
Mean edge weigth: 156.54
Max. degree: 67

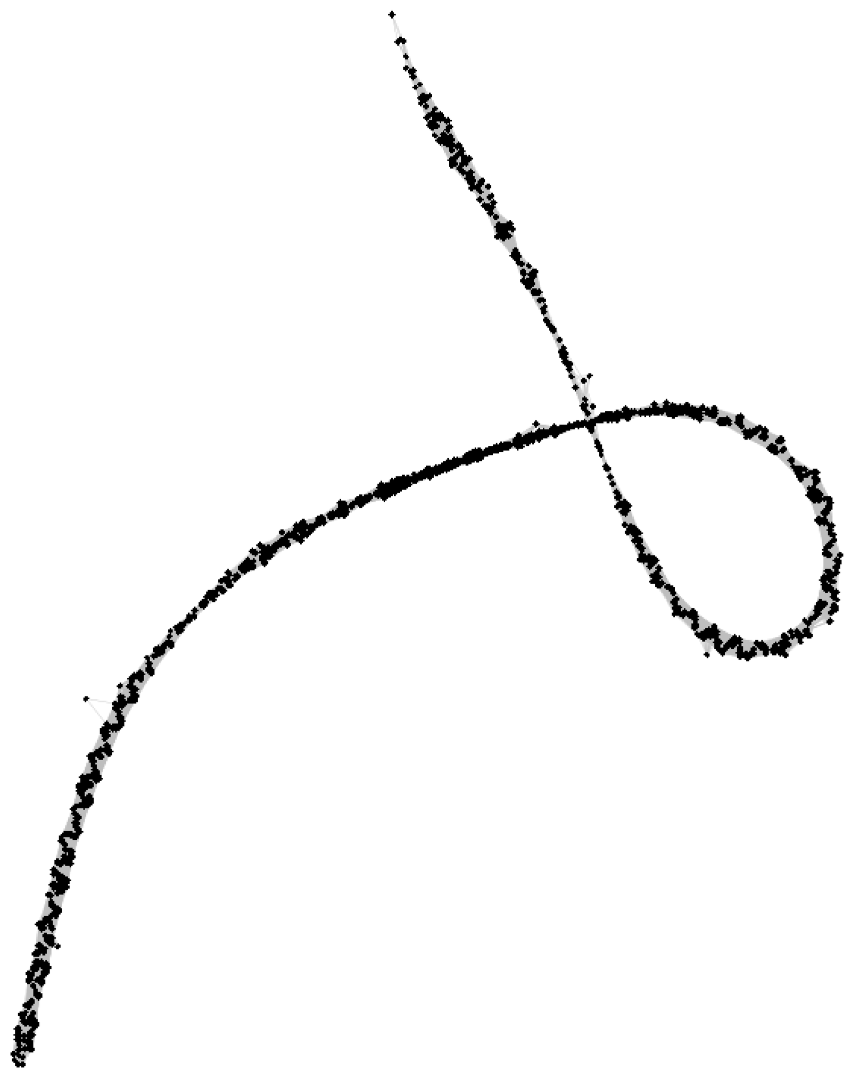

CL118

Number of reads: 1617
Number of pairs: 46058
Density: 0.03525
Diameter: NA
Mean edge weigth: 215.42
Max. degree: 86

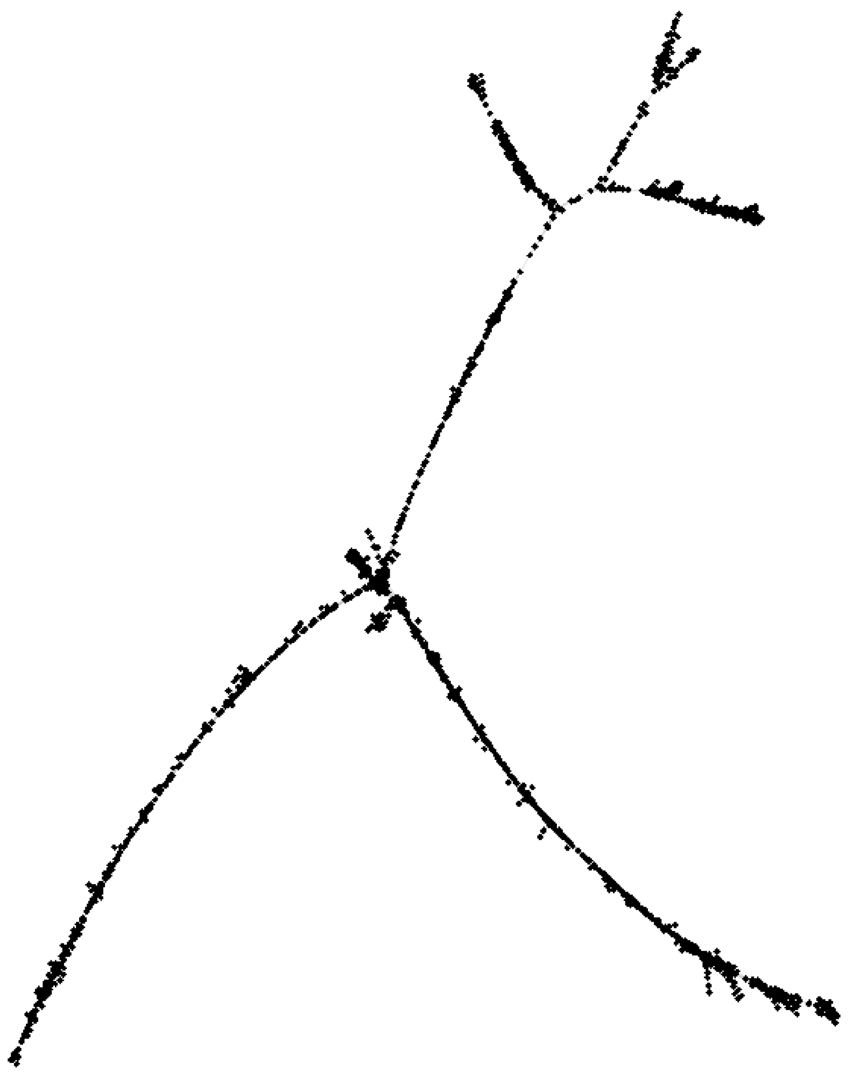

CL119

Number of reads: 1569
Number of pairs: 11334
Density: 0.009214
Diameter: NA
Mean edge weigth: 172.74
Max. degree: 41

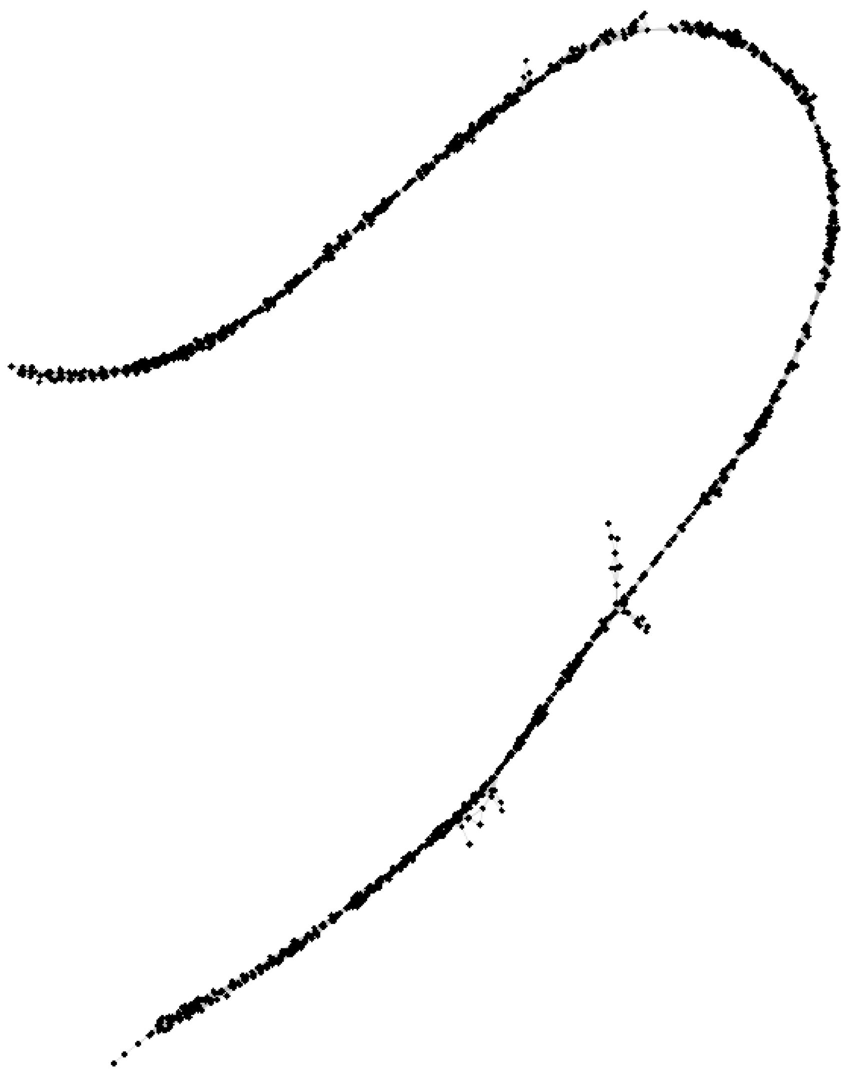

CL120

Number of reads: 1486
Number of pairs: 19969
Density: 0.0181
Diameter: NA
Mean edge weigth: 213.78
Max. degree: 44

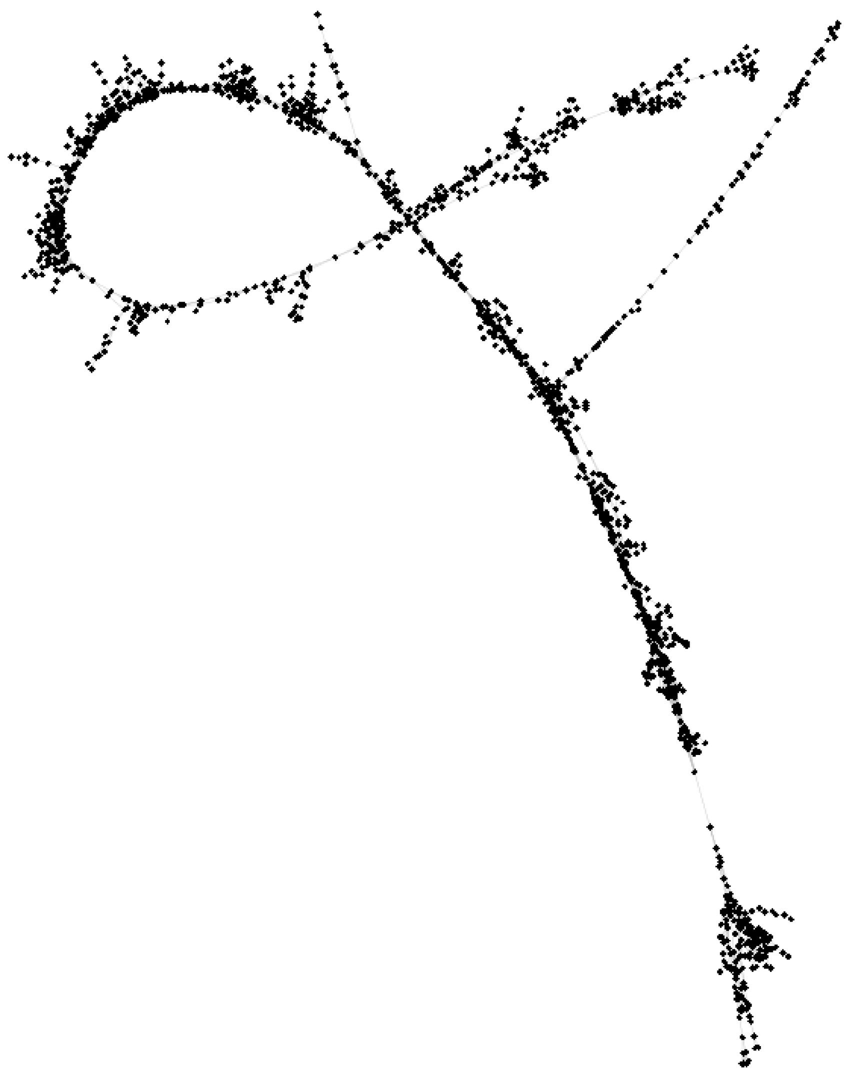

CL121

Number of reads: 1452
Number of pairs: 6587
Density: 0.006253
Diameter: NA
Mean edge weigth: 153.1
Max. degree: 43

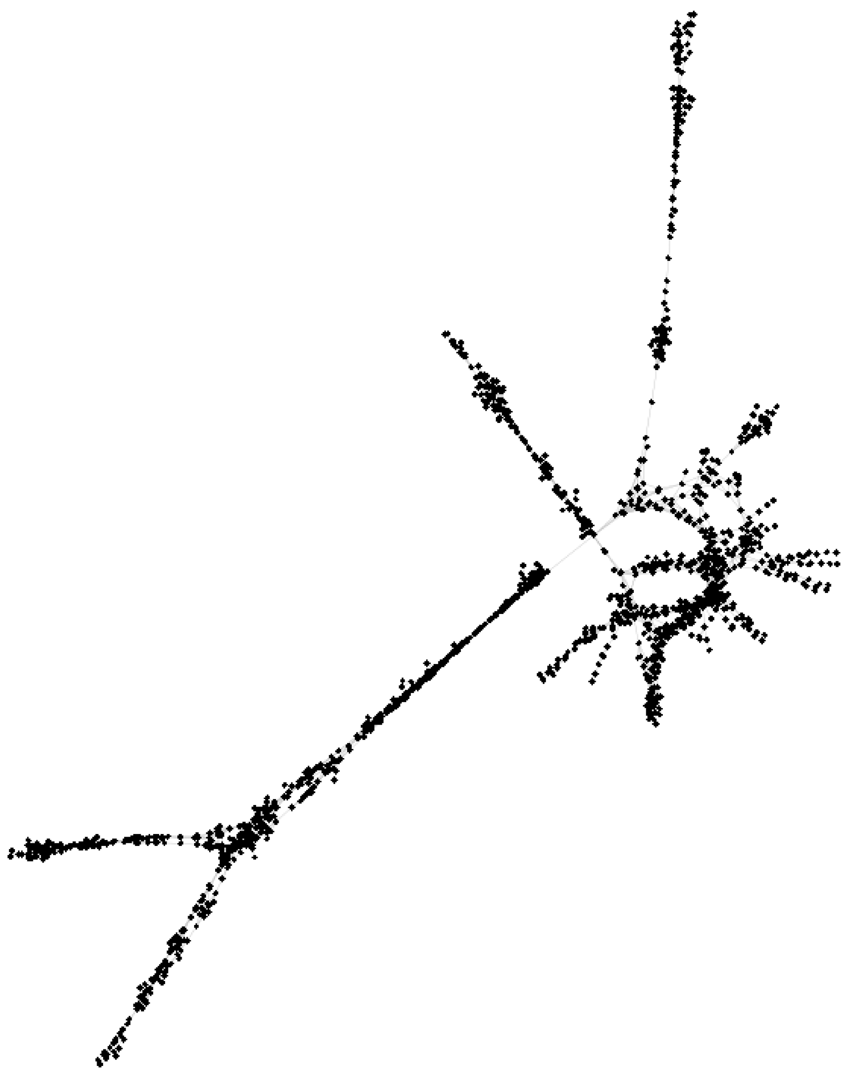

CL122

Number of reads: 1447
Number of pairs: 7778
Density: 0.007435
Diameter: NA
Mean edge weigth: 158.18
Max. degree: 49

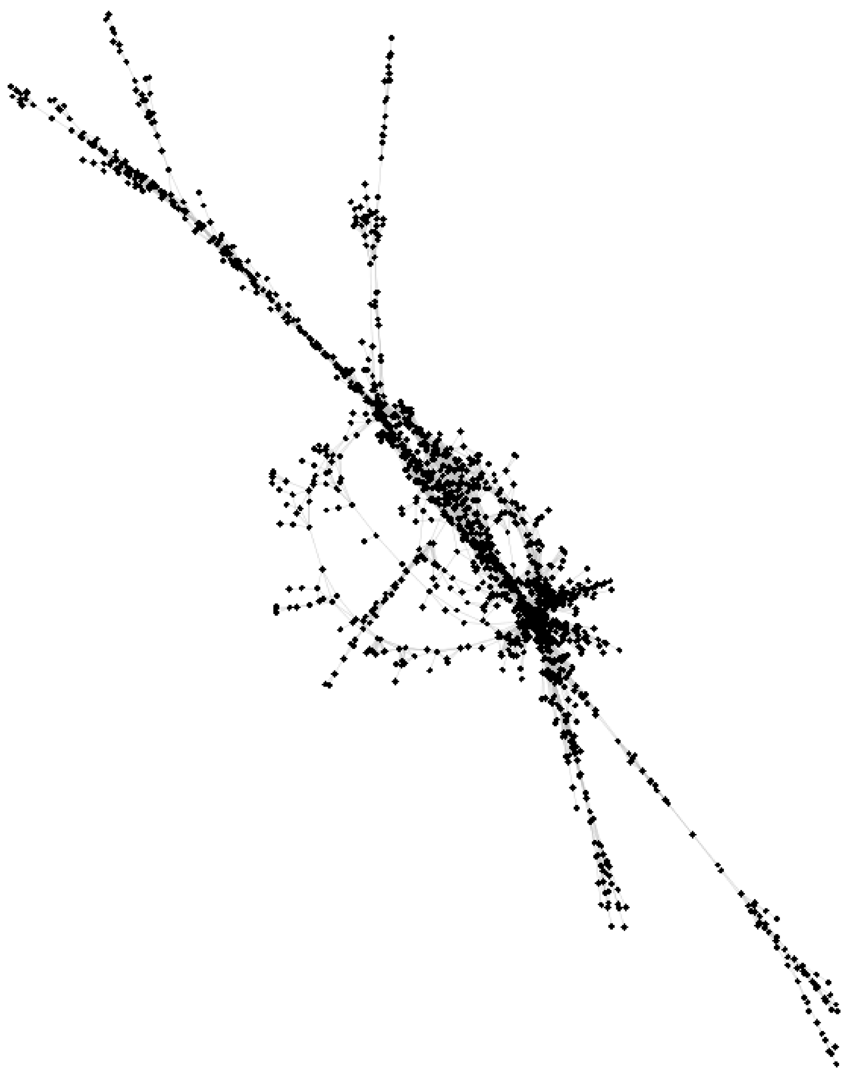

CL123

Number of reads: 1446
Number of pairs: 12750
Density: 0.0122
Diameter: NA
Mean edge weigth: 156.17
Max. degree: 133

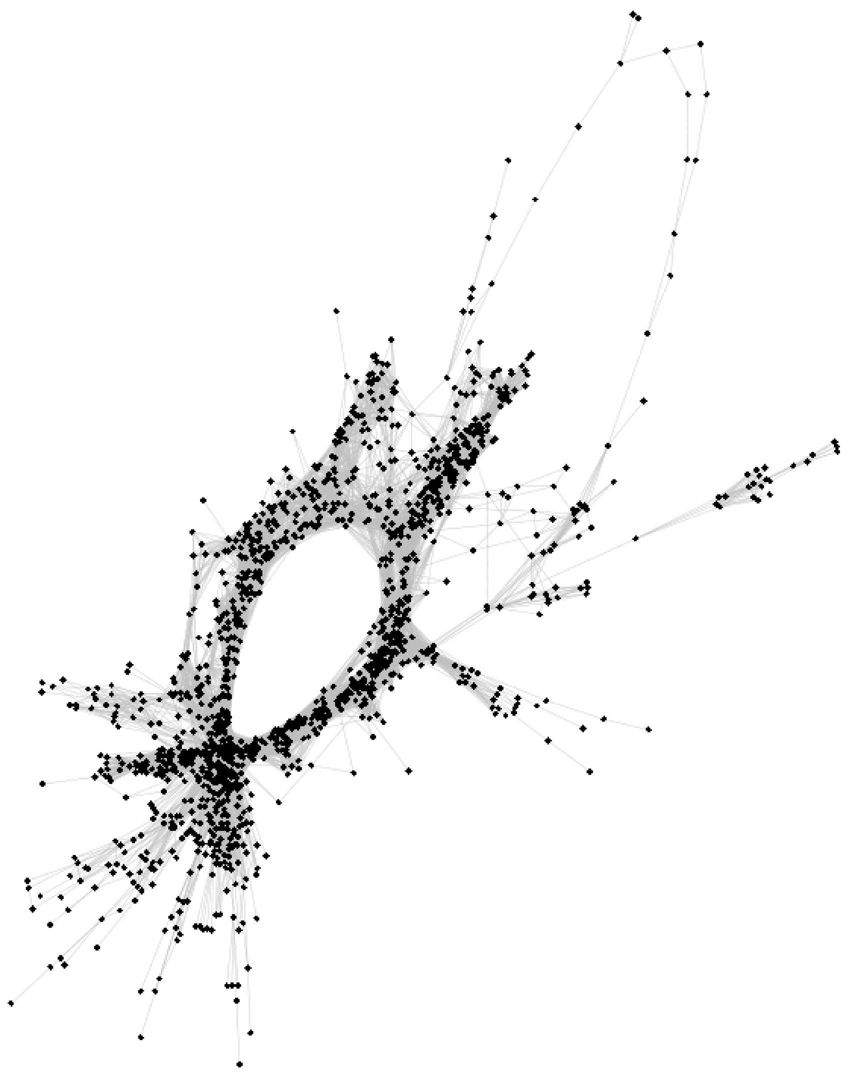

CL124

Number of reads: 1417
Number of pairs: 32321
Density: 0.03222
Diameter: NA
Mean edge weigth: 156.39
Max. degree: 190

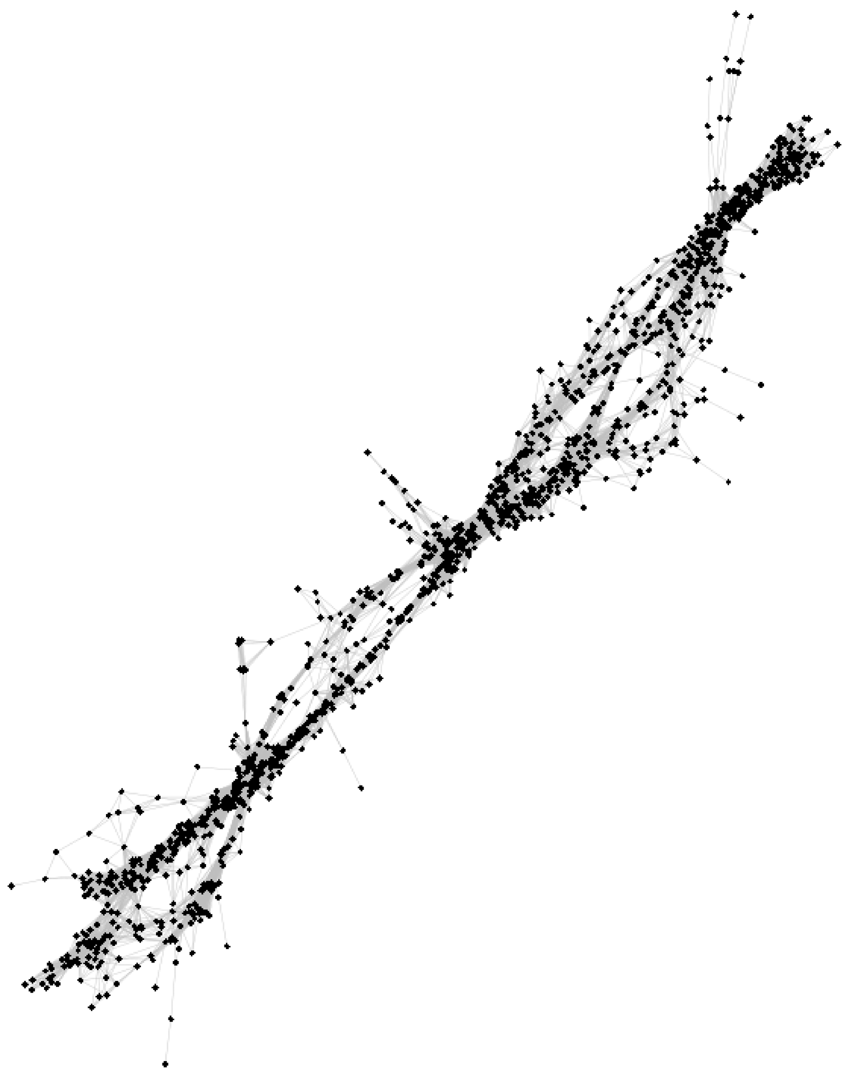

CL125

Number of reads: 1405
Number of pairs: 27751
Density: 0.02814
Diameter: NA
Mean edge weigth: 160.71
Max. degree: 107

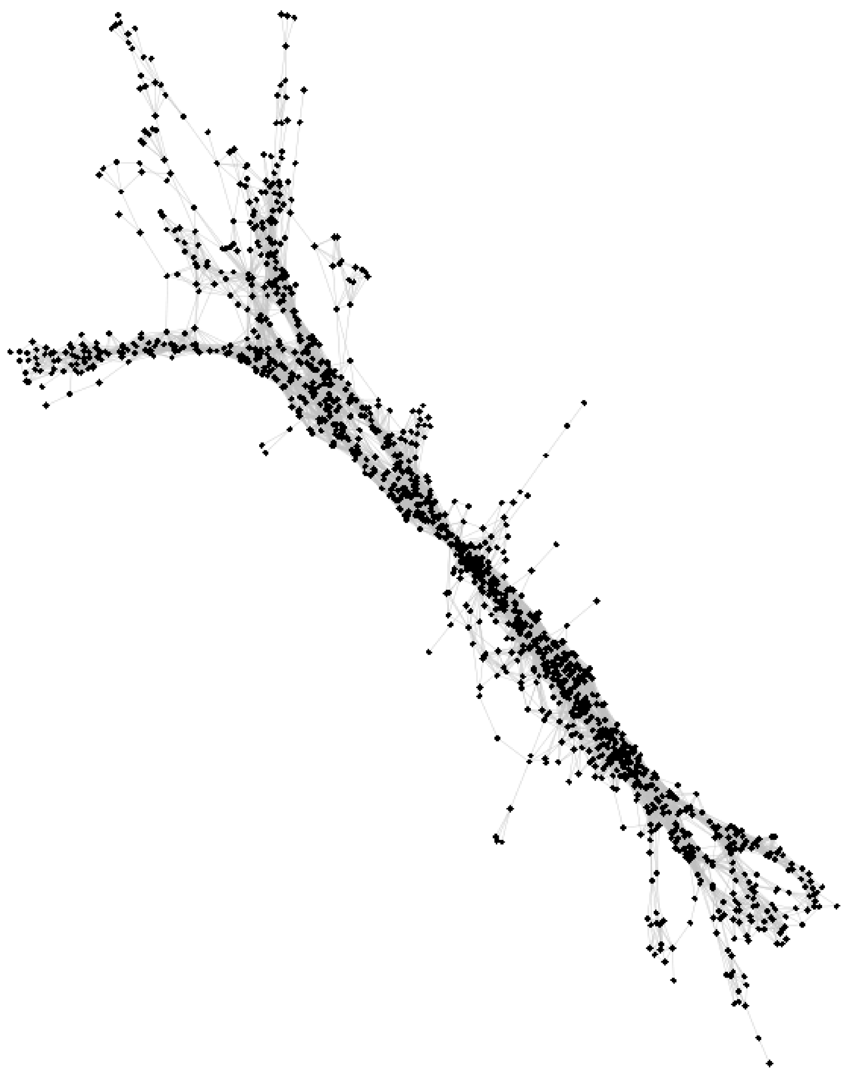

CL126

Number of reads: 1321
Number of pairs: 19165
Density: 0.02198
Diameter: NA
Mean edge weigth: 156.79
Max. degree: 88

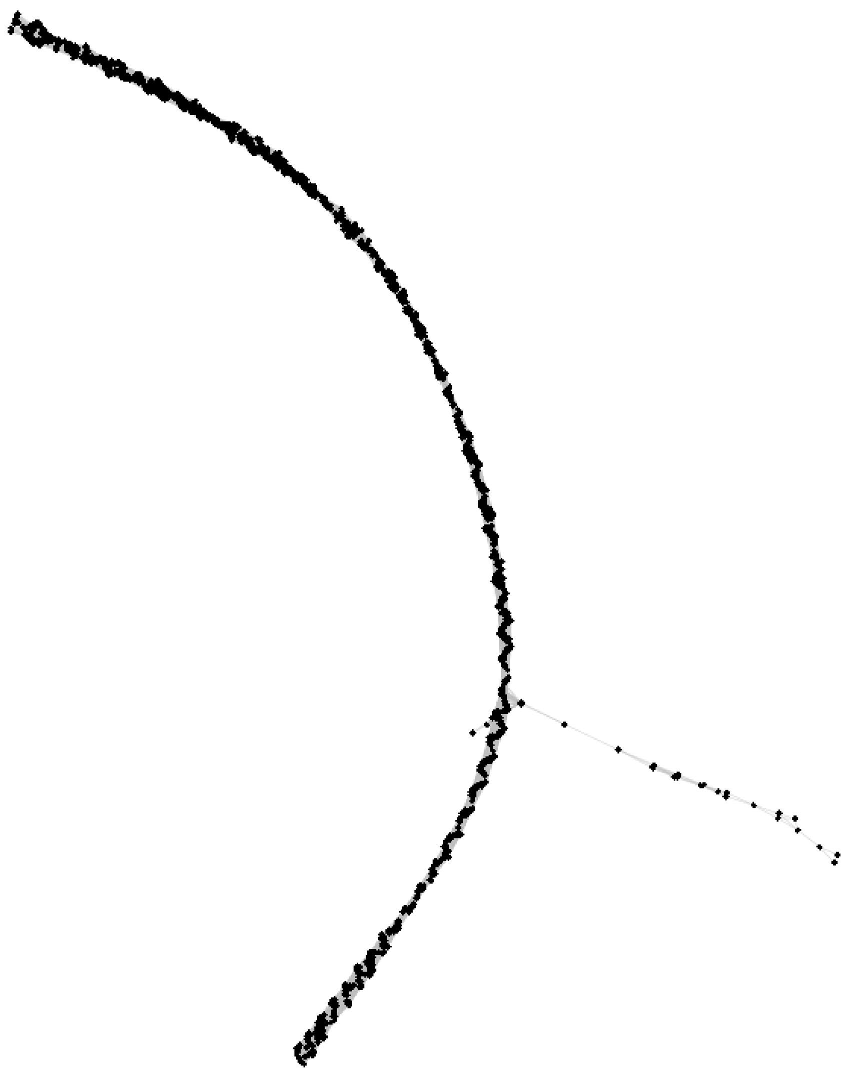

CL127

Number of reads: 1317
Number of pairs: 37175
Density: 0.0429
Diameter: NA
Mean edge weigth: 215
Max. degree: 82

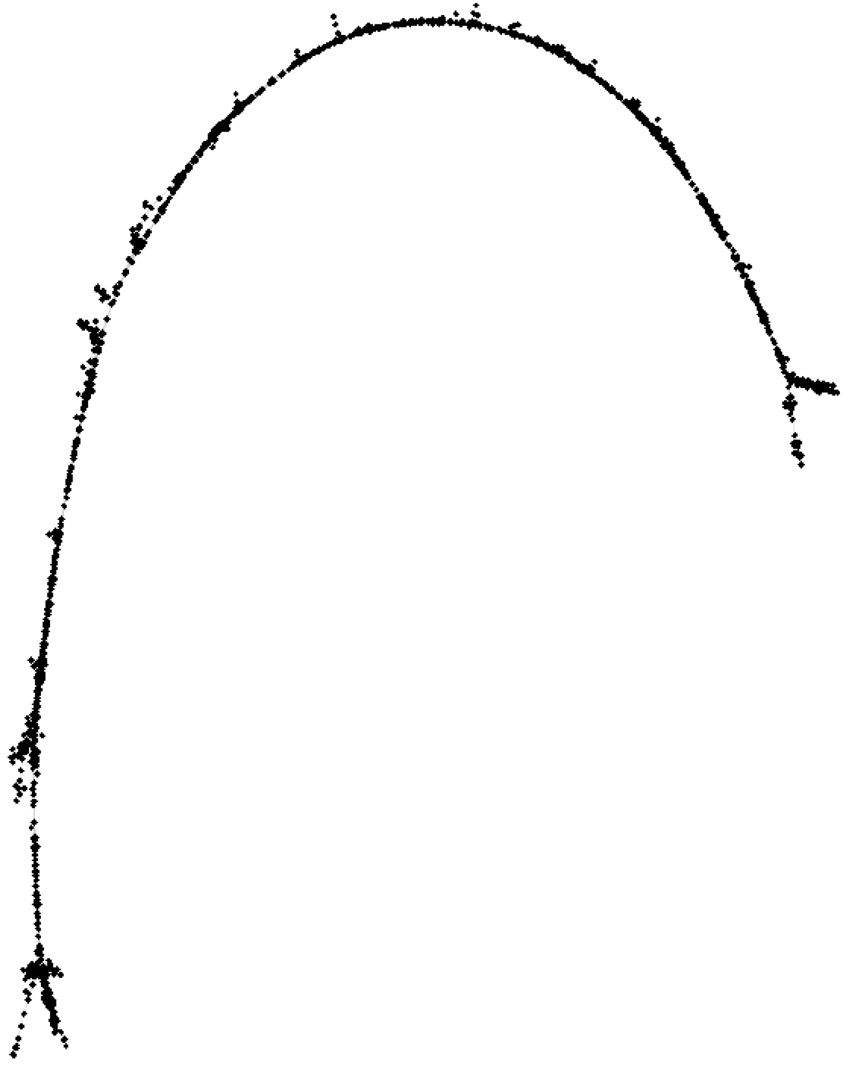

CL128

Number of reads: 1298
Number of pairs: 10212
Density: 0.01213
Diameter: NA
Mean edge weigth: 181.53
Max. degree: 48

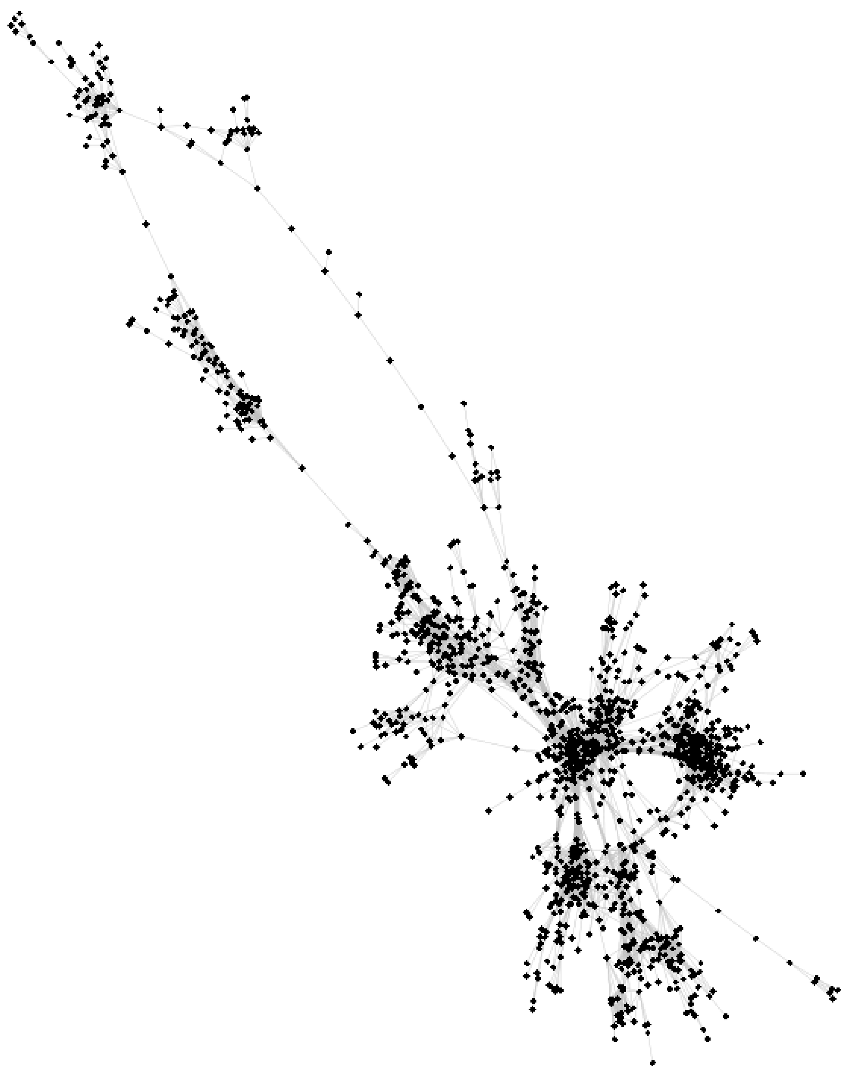

CL129

Number of reads: 1297
Number of pairs: 8441
Density: 0.01004
Diameter: NA
Mean edge weigth: 141.4
Max. degree: 105

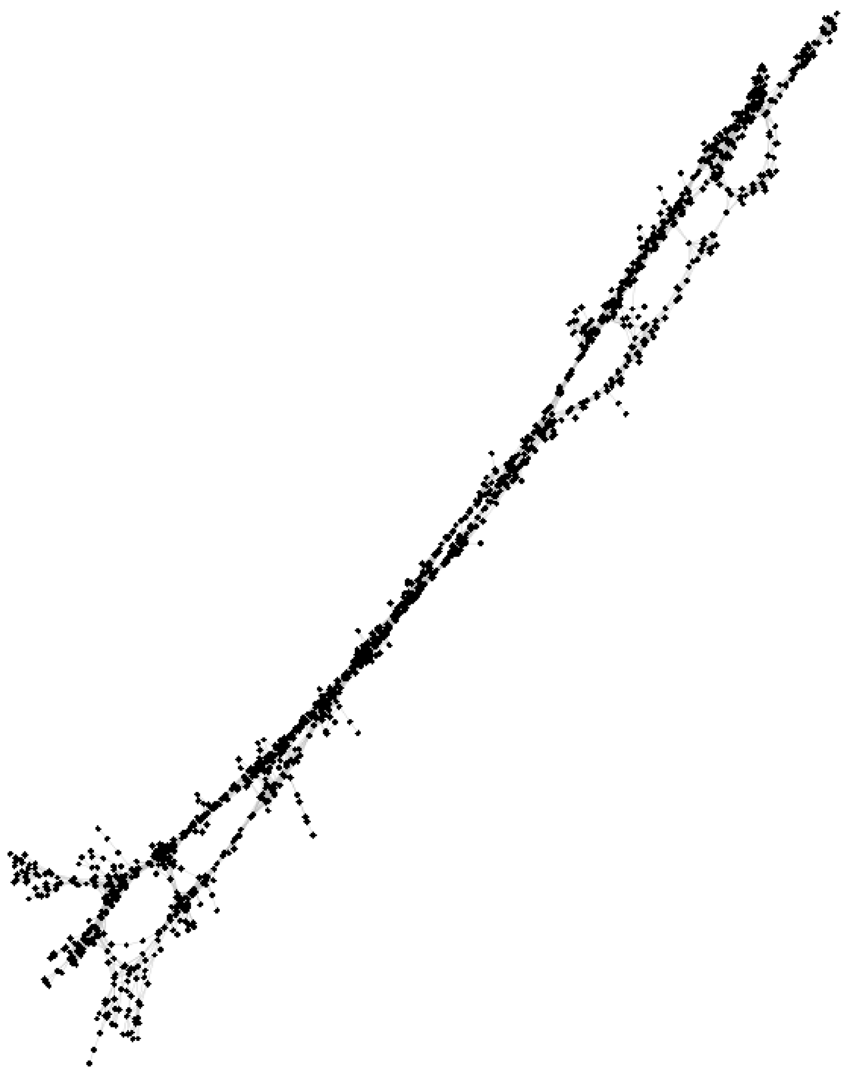

CL130

Number of reads: 1269
Number of pairs: 10648
Density: 0.01323
Diameter: NA
Mean edge weigth: 164.56
Max. degree: 44

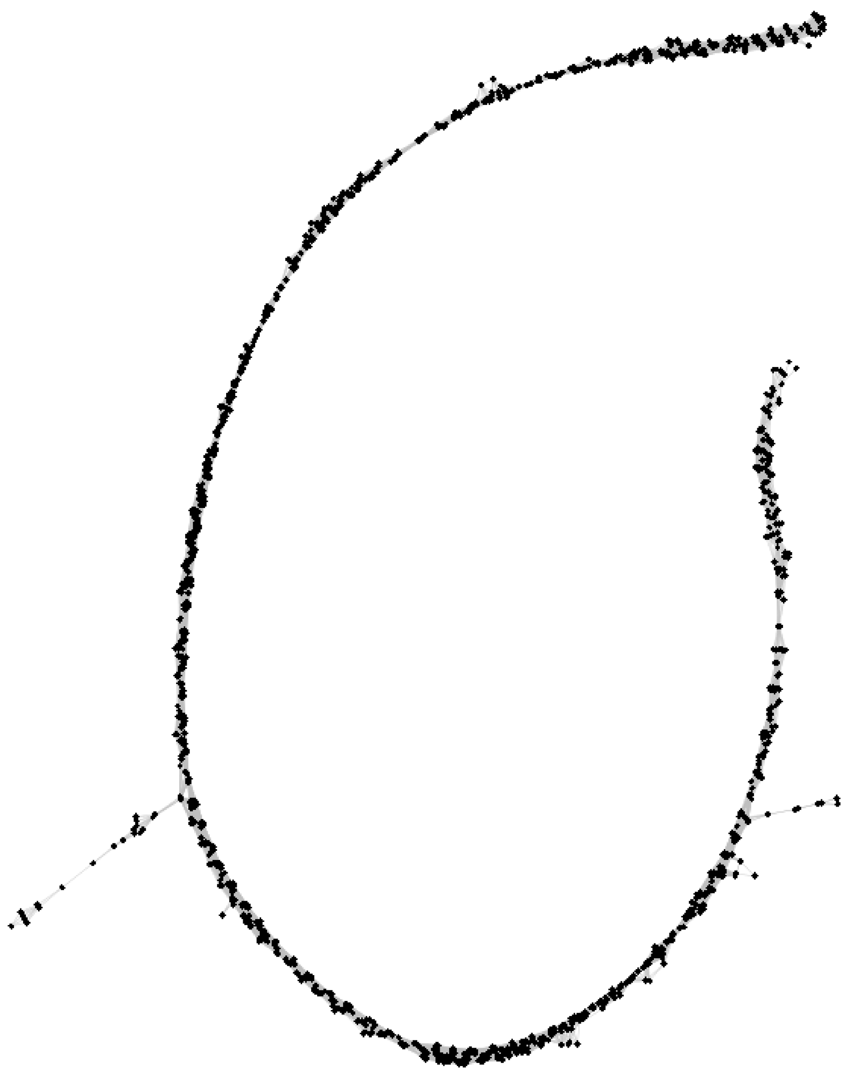

CL131

Number of reads: 1216
Number of pairs: 17384
Density: 0.02353
Diameter: NA
Mean edge weigth: 214.38
Max. degree: 49

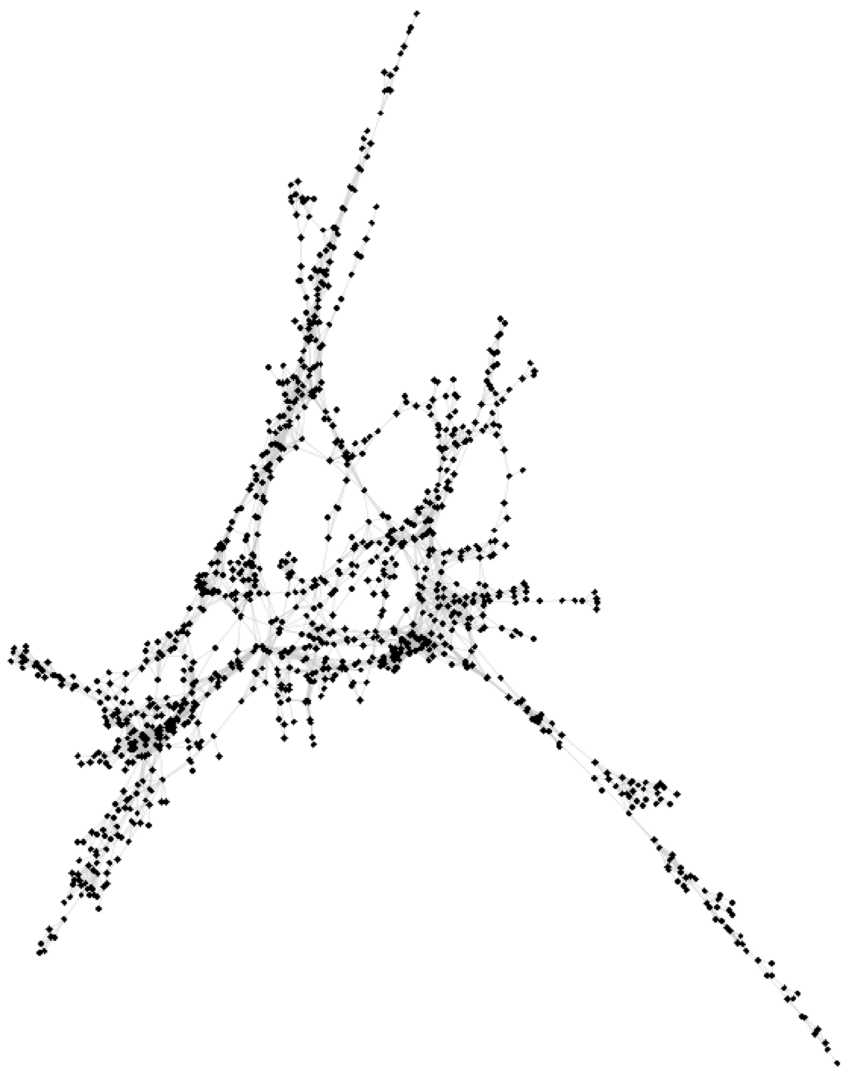

CL132

Number of reads: 1043
Number of pairs: 4444
Density: 0.008178
Diameter: NA
Mean edge weigth: 160.55
Max. degree: 35

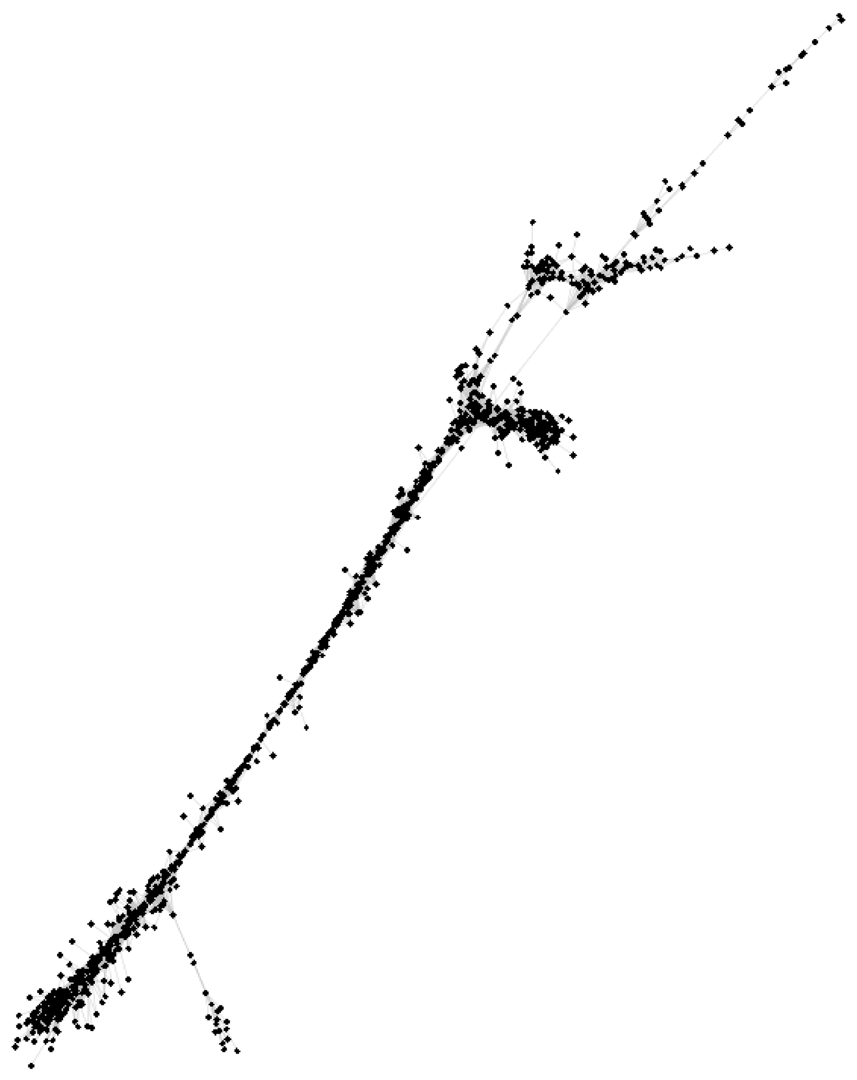

CL133

Number of reads: 995
Number of pairs: 13700
Density: 0.0277
Diameter: NA
Mean edge weigth: 153.16
Max. degree: 81

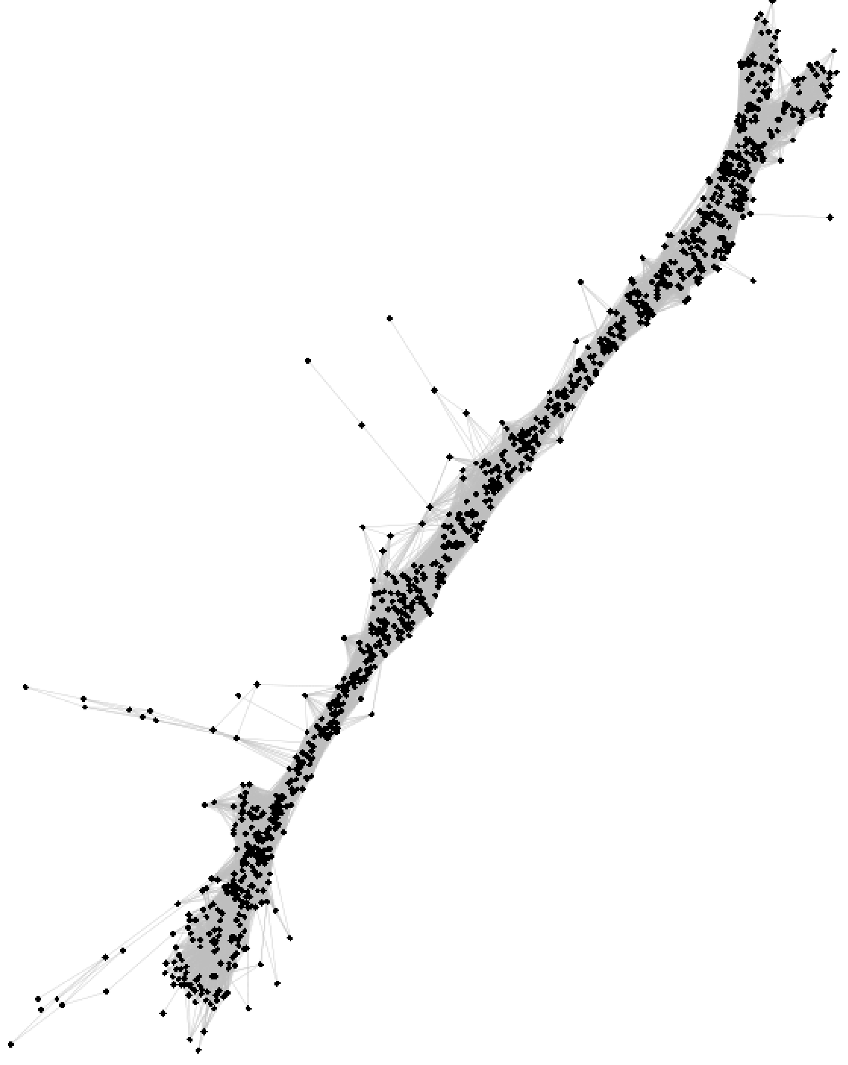

CL134

Number of reads: 995
Number of pairs: 39049
Density: 0.07896
Diameter: NA
Mean edge weigth: 170.92
Max. degree: 161

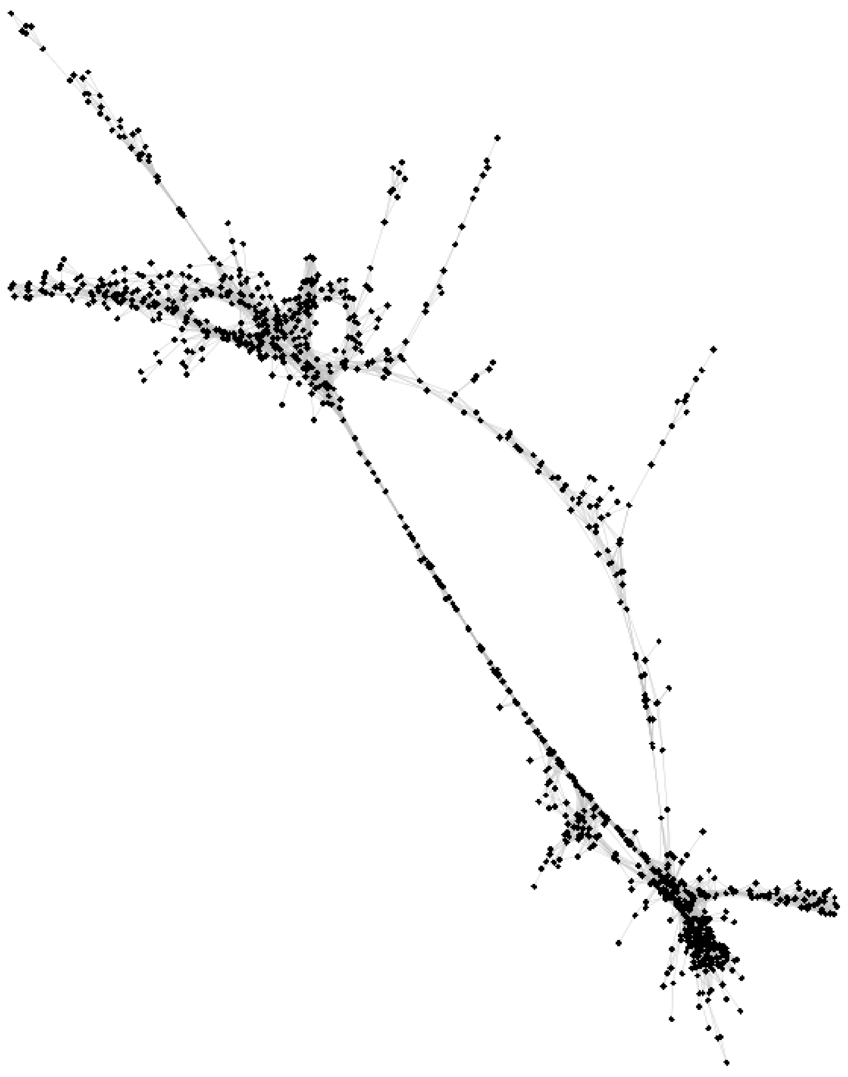

CL135

Number of reads: 980
Number of pairs: 9942
Density: 0.02073
Diameter: NA
Mean edge weigth: 153.88
Max. degree: 106

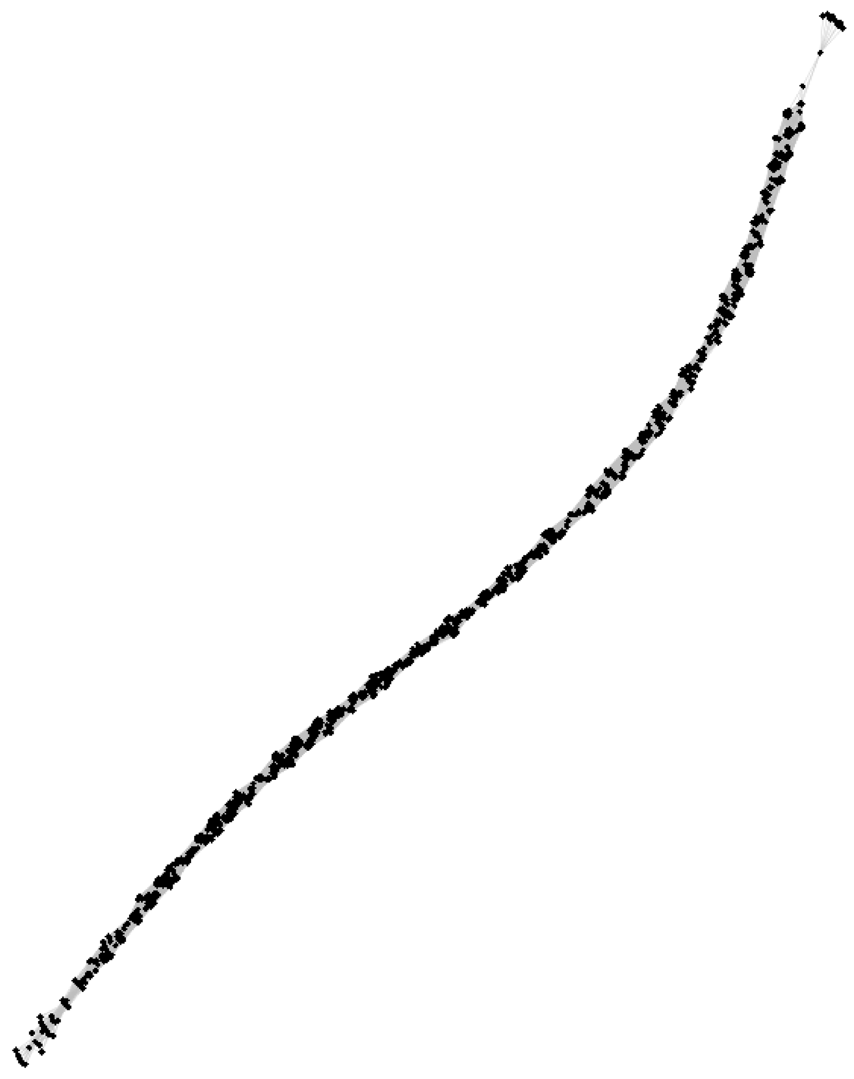

CL136

Number of reads: 974
Number of pairs: 25659
Density: 0.05415
Diameter: NA
Mean edge weigth: 214.94
Max. degree: 79

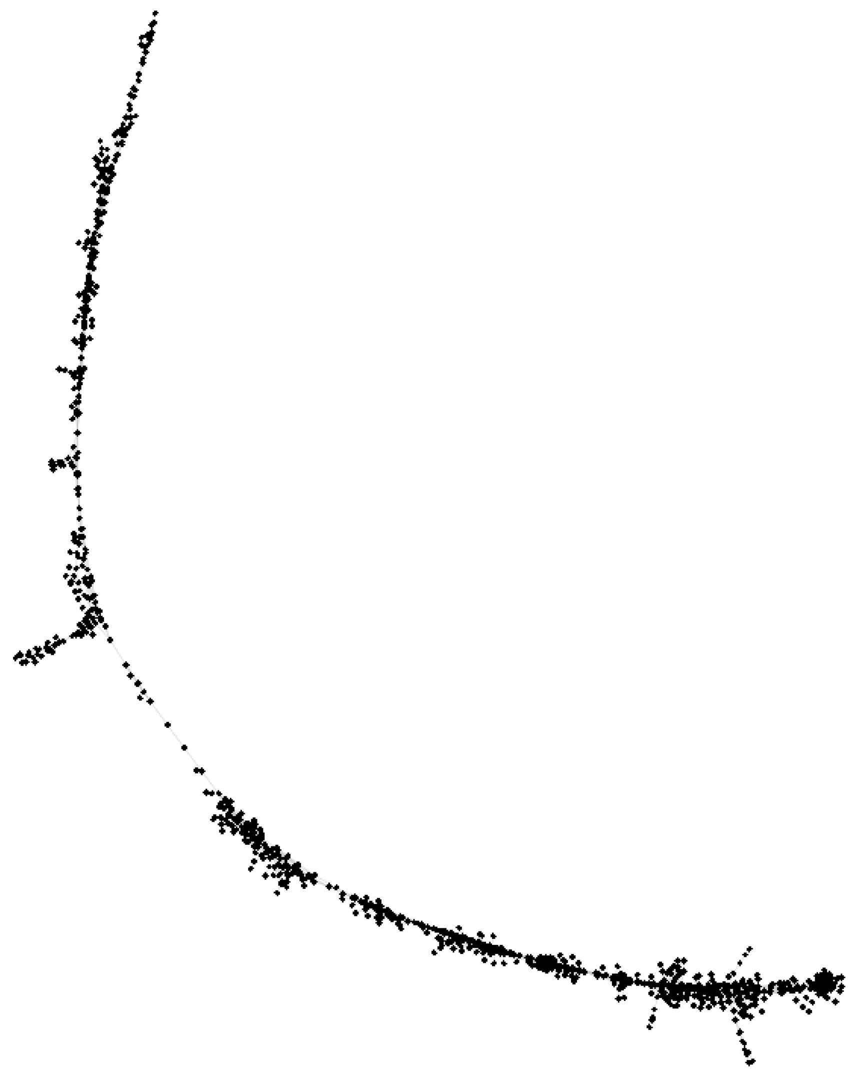

CL137

Number of reads: 959
Number of pairs: 4163
Density: 0.009063
Diameter: NA
Mean edge weigth: 148.25
Max. degree: 35

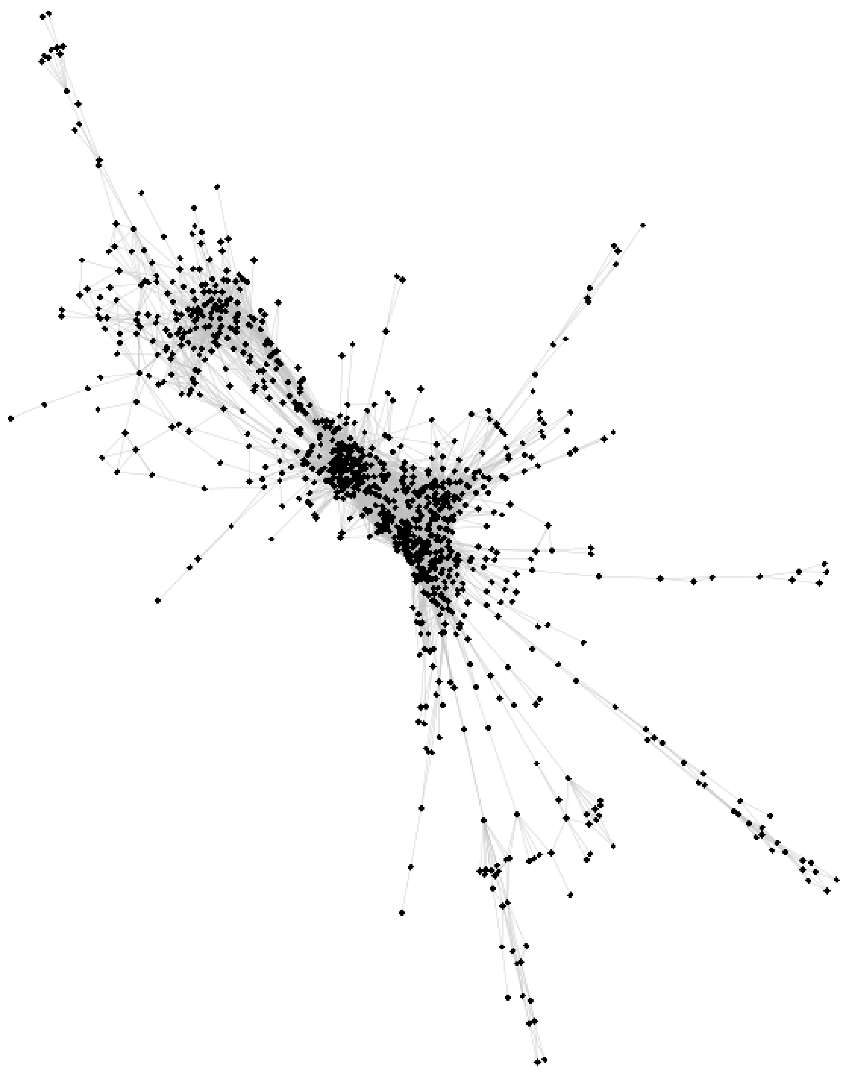

CL138

Number of reads: 944
Number of pairs: 12900
Density: 0.02898
Diameter: NA
Mean edge weigth: 145.78
Max. degree: 141

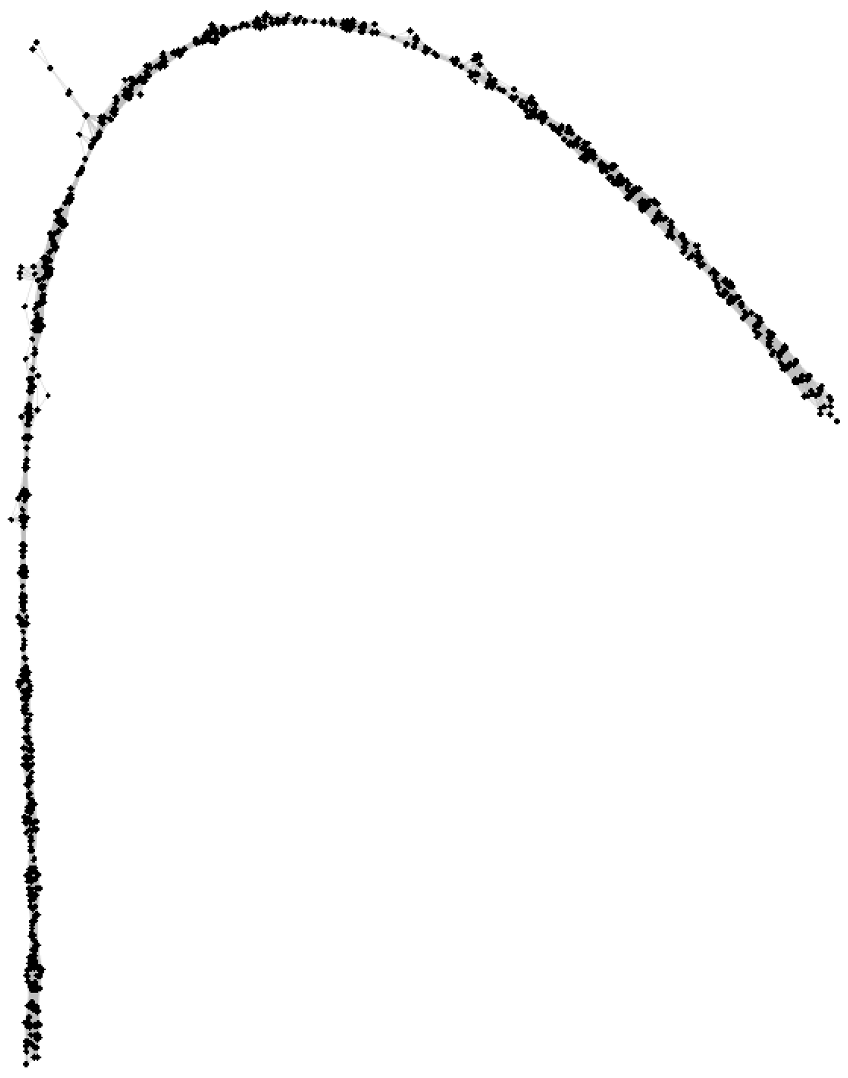

CL139

Number of reads: 936
Number of pairs: 13634
Density: 0.03116
Diameter: NA
Mean edge weigth: 215.08
Max. degree: 49

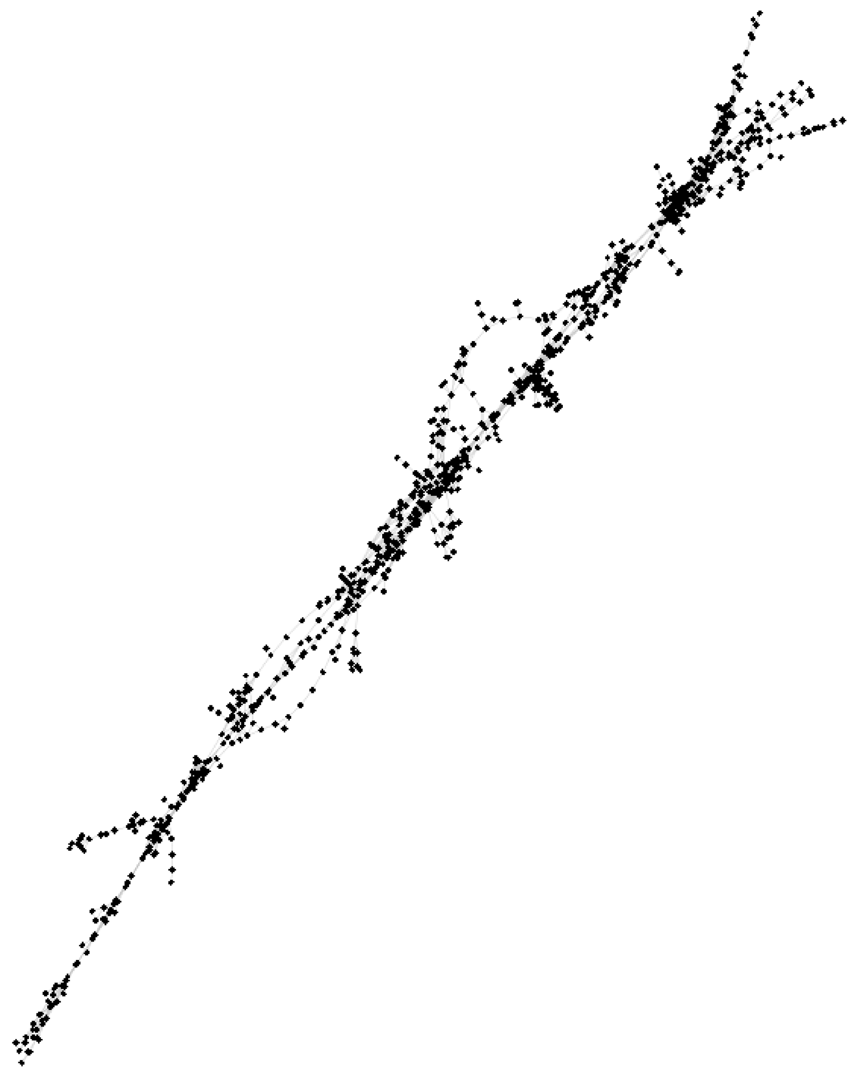

CL140

Number of reads: 836
Number of pairs: 3719
Density: 0.01066
Diameter: NA
Mean edge weigth: 160.05
Max. degree: 44

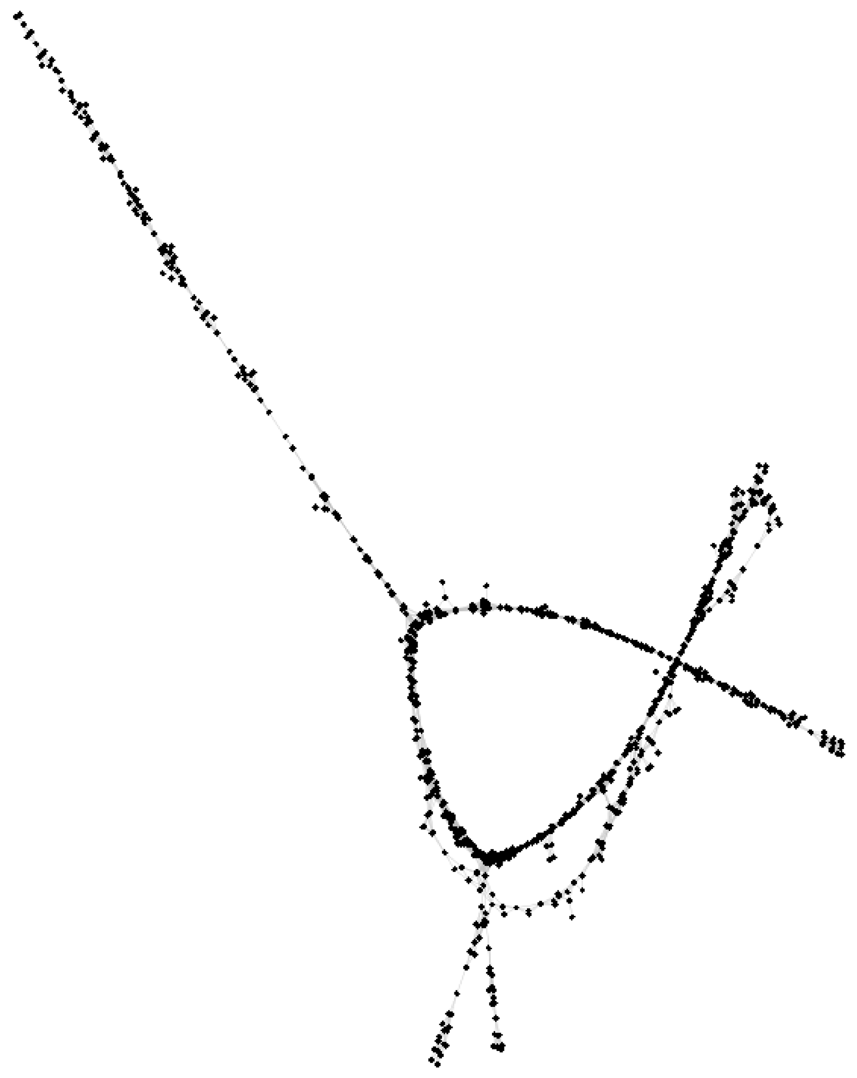

CL141

Number of reads: 835
Number of pairs: 5693
Density: 0.01635
Diameter: NA
Mean edge weigth: 177.21
Max. degree: 33

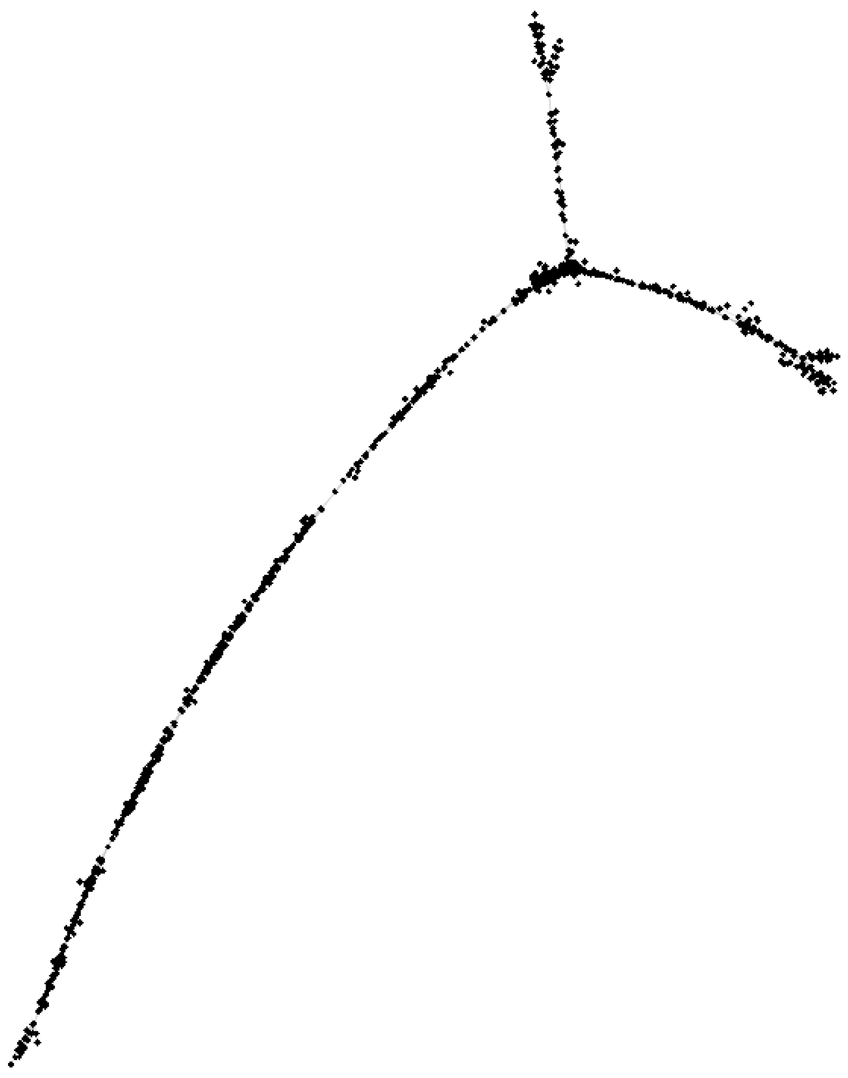

CL142

Number of reads: 794
Number of pairs: 5429
Density: 0.01724
Diameter: NA
Mean edge weigth: 169.42
Max. degree: 49

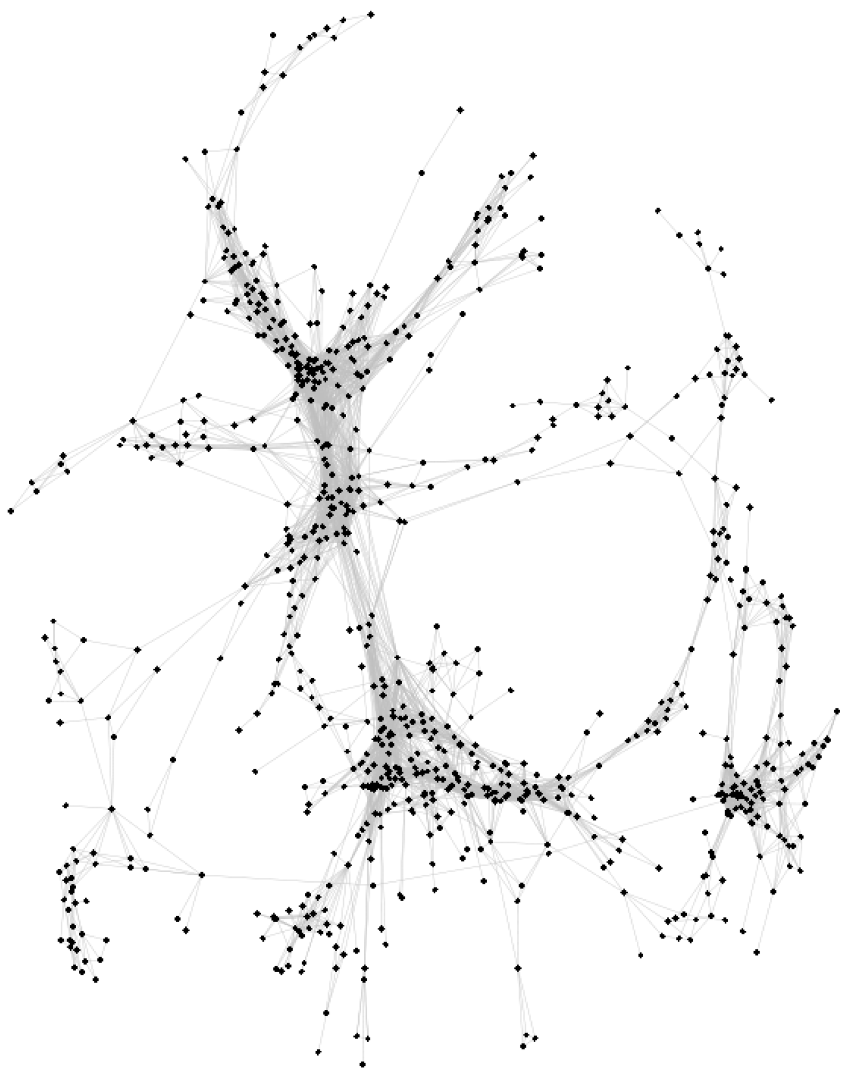

CL143

Number of reads: 782
Number of pairs: 5419
Density: 0.01775
Diameter: NA
Mean edge weigth: 163.29
Max. degree: 68

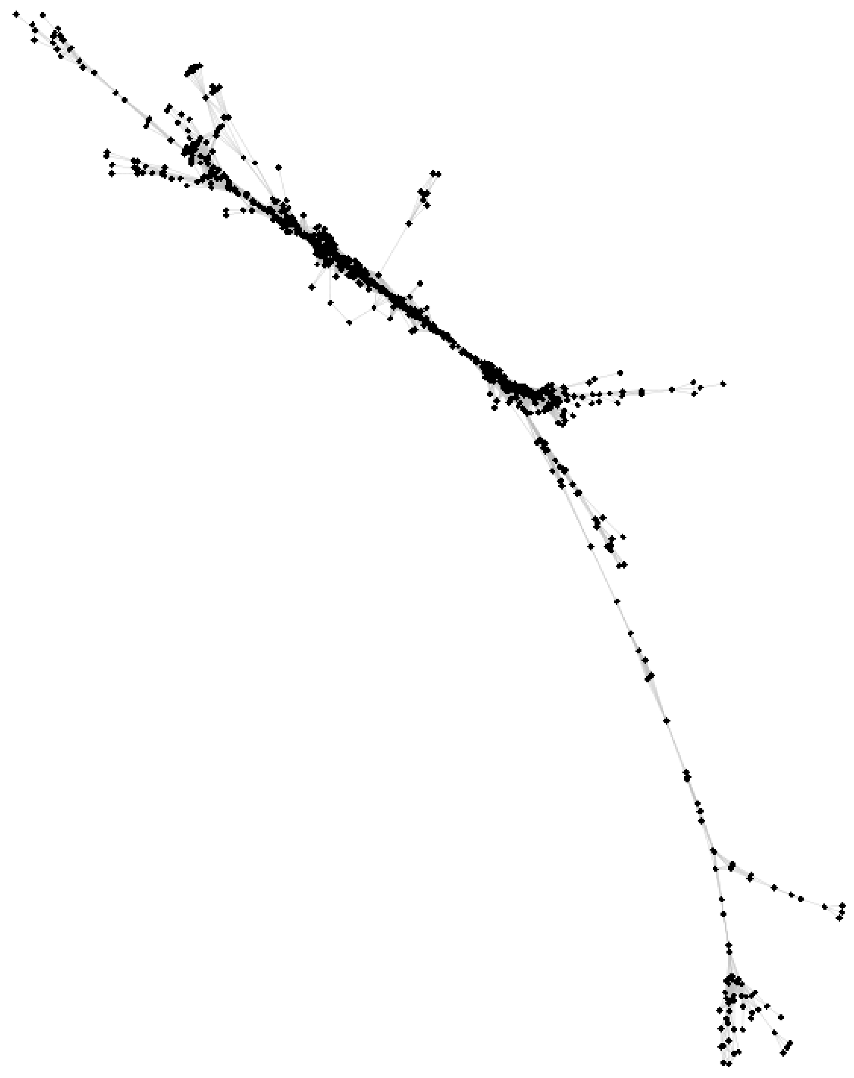

CL144

Number of reads: 751
Number of pairs: 15537
Density: 0.05517
Diameter: NA
Mean edge weigth: 174.83
Max. degree: 119

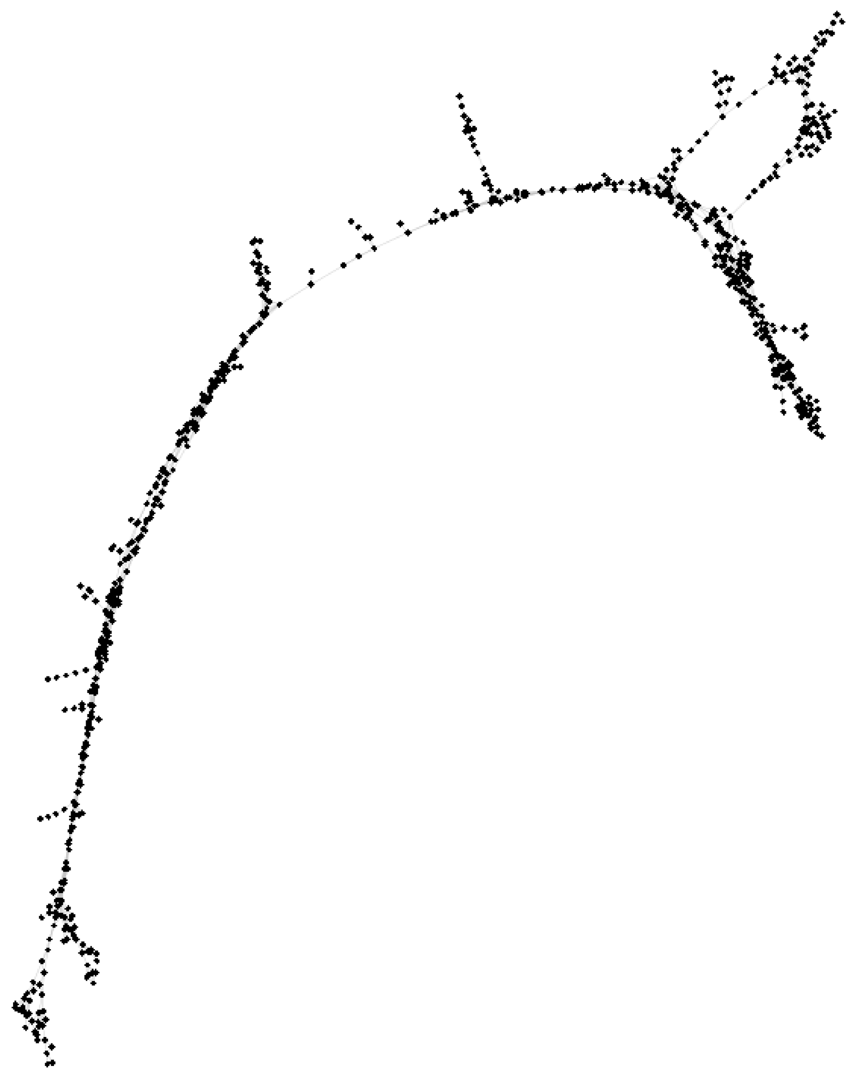

CL145

Number of reads: 734
Number of pairs: 2932
Density: 0.0109
Diameter: NA
Mean edge weigth: 162.29
Max. degree: 22

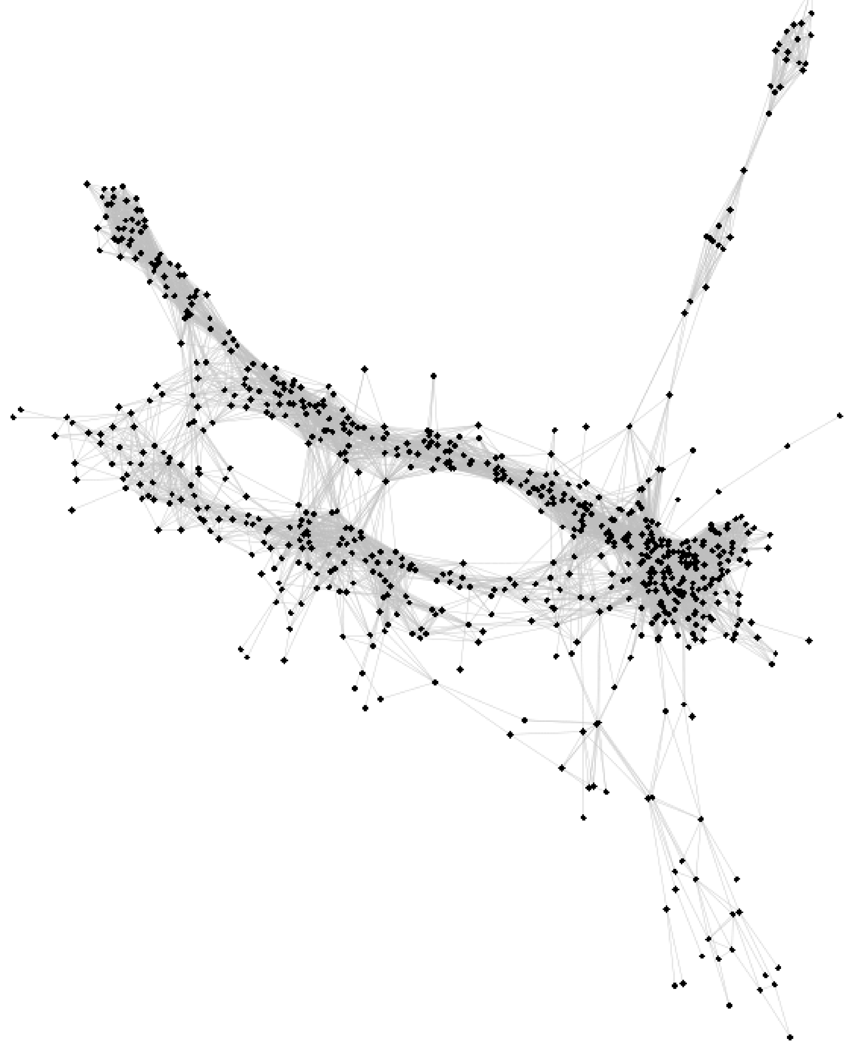

CL146

Number of reads: 691
Number of pairs: 9294
Density: 0.03899
Diameter: NA
Mean edge weigth: 153.28
Max. degree: 110

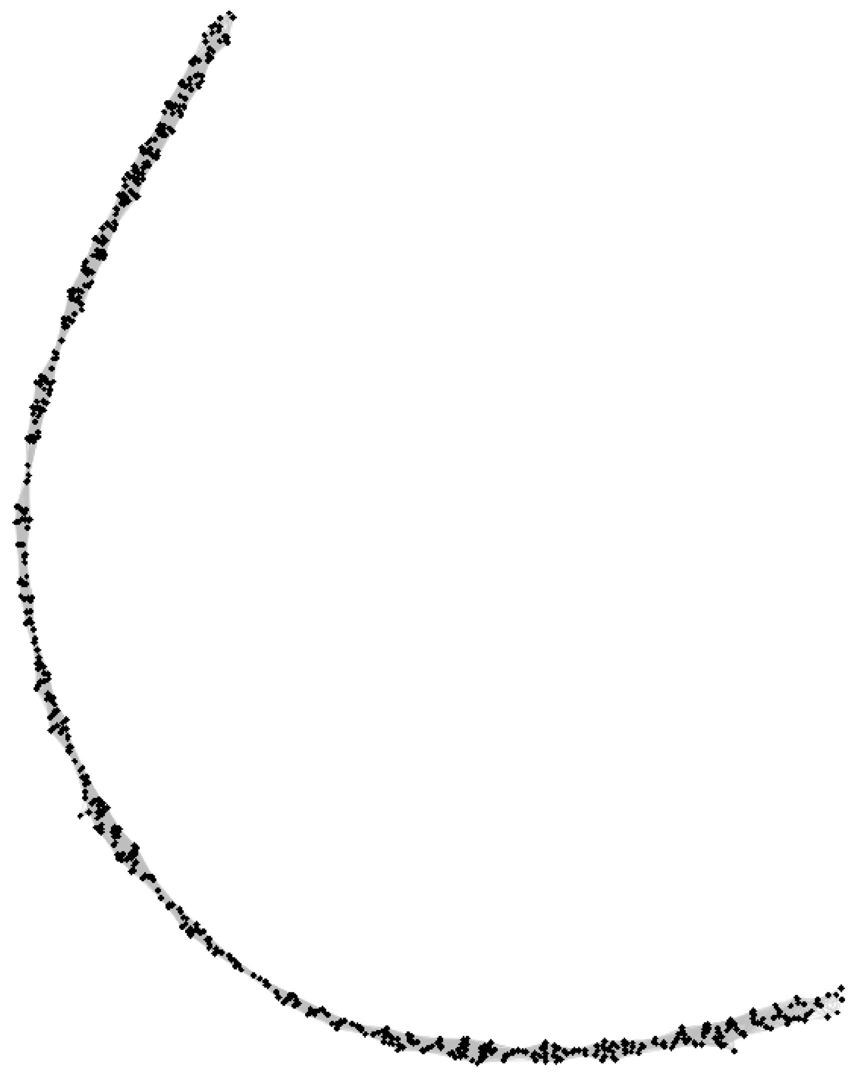

CL147

Number of reads: 628
Number of pairs: 9000
Density: 0.04571
Diameter: NA
Mean edge weigth: 214.11
Max. degree: 47

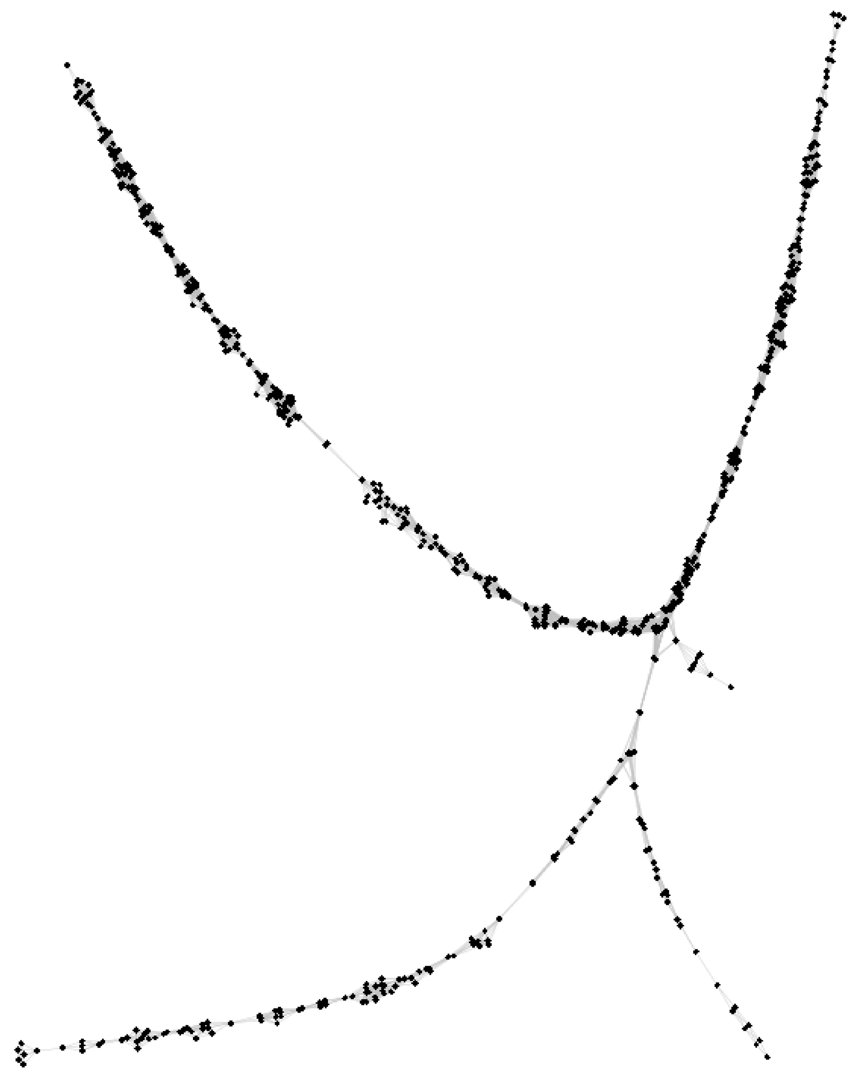

CL148

Number of reads: 625
Number of pairs: 5736
Density: 0.02942
Diameter: NA
Mean edge weigth: 216.12
Max. degree: 37

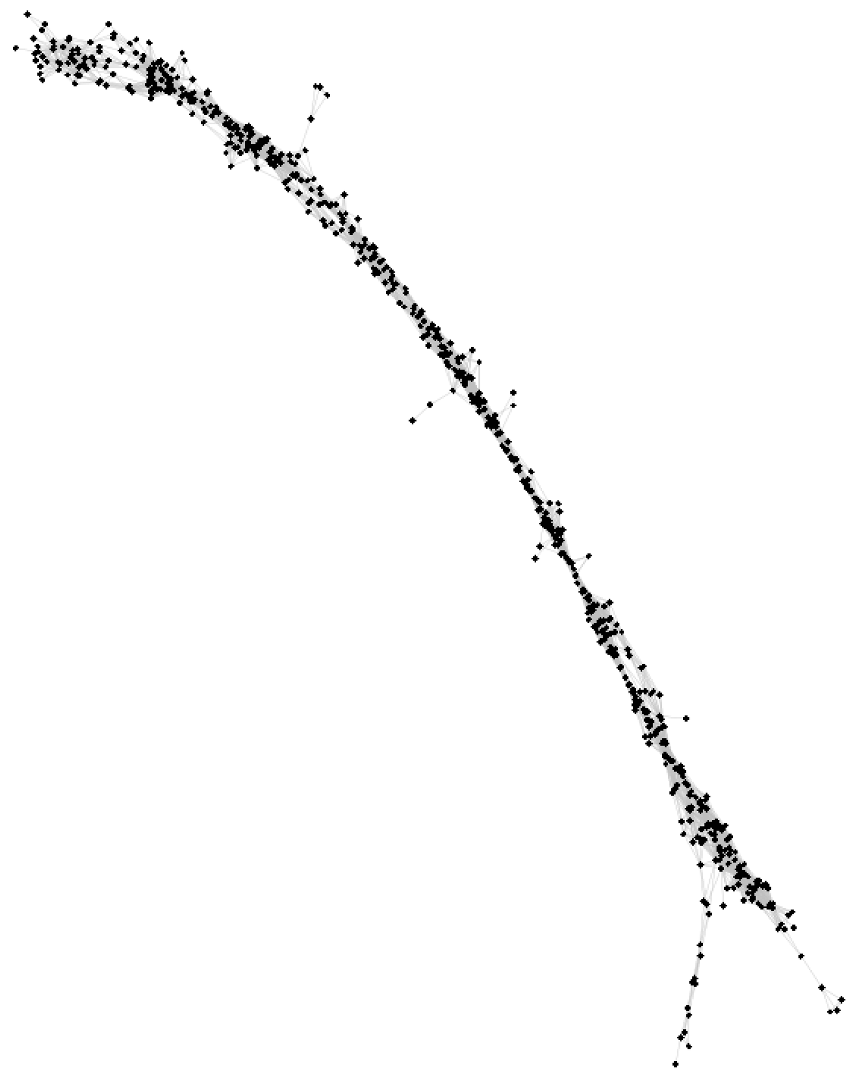

CL149

Number of reads: 618
Number of pairs: 6983
Density: 0.03663
Diameter: NA
Mean edge weigth: 165.06
Max. degree: 44

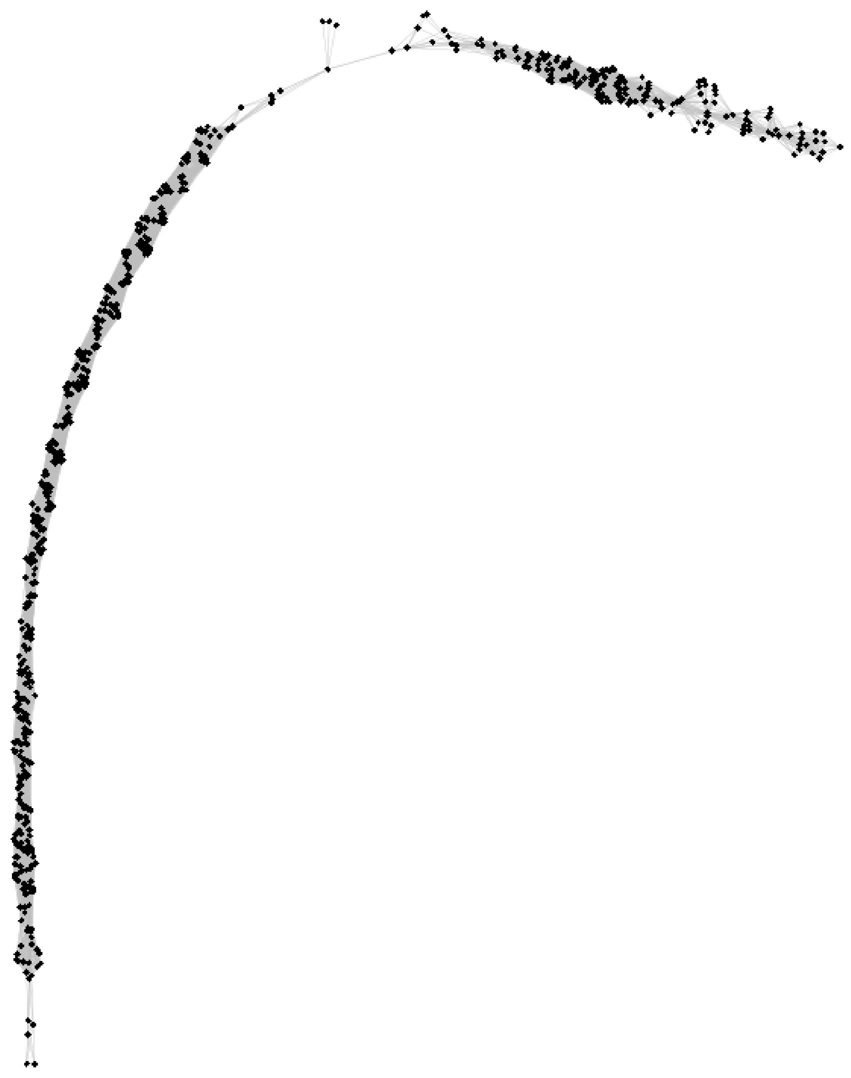

CL150

Number of reads: 608
Number of pairs: 11681
Density: 0.0633
Diameter: NA
Mean edge weigth: 215.89
Max. degree: 60

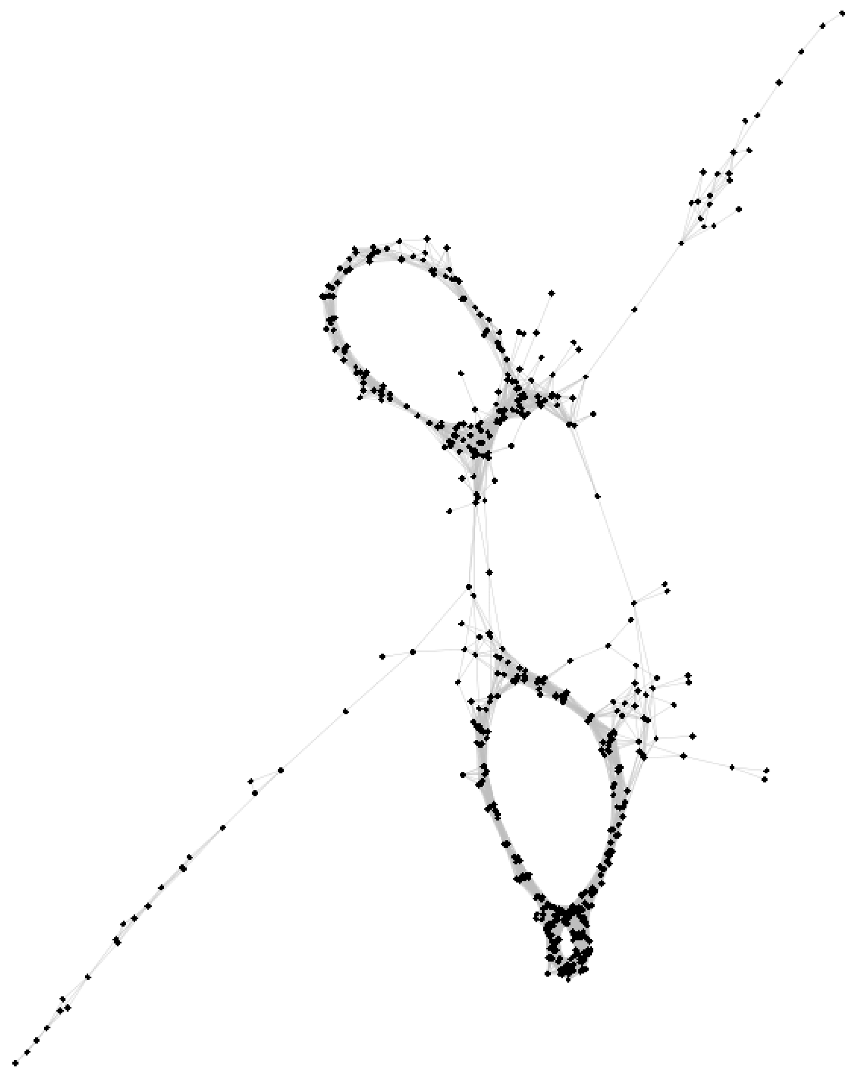

CL151

Number of reads: 548
Number of pairs: 7841
Density: 0.05232
Diameter: NA
Mean edge weigth: 186.89
Max. degree: 88

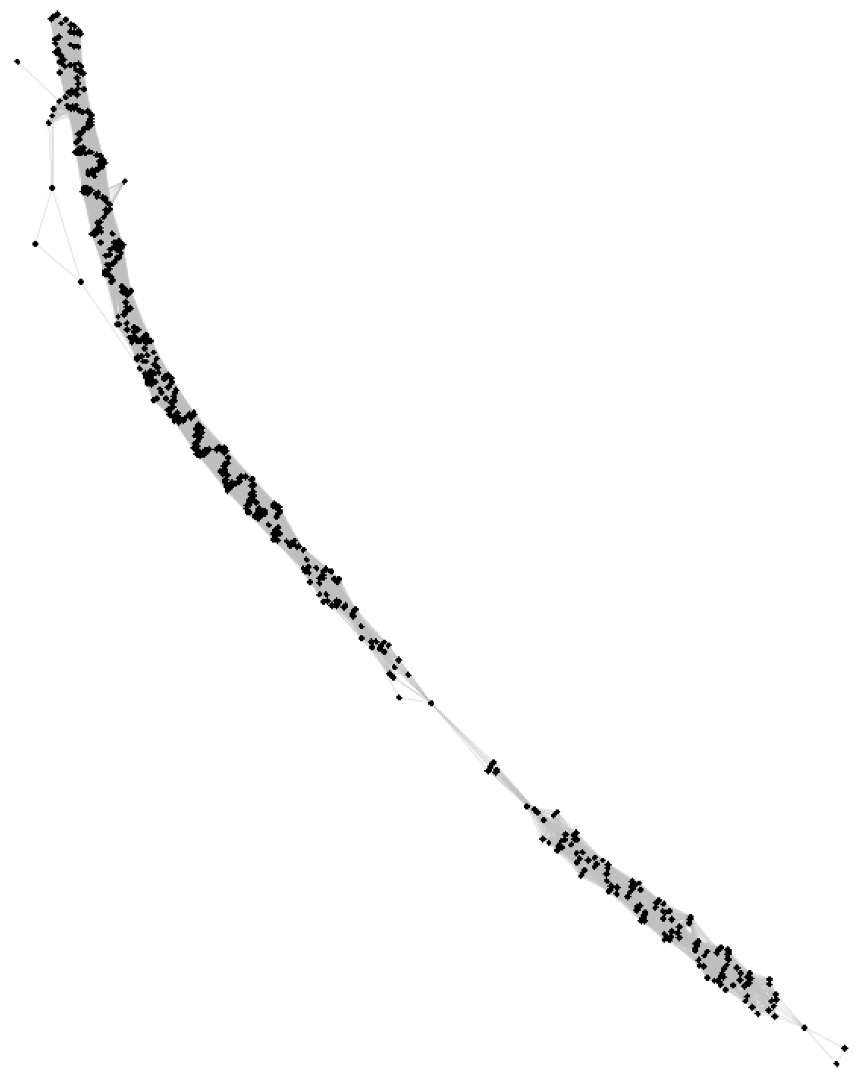

CL152

Number of reads: 546
Number of pairs: 13168
Density: 0.0885
Diameter: NA
Mean edge weigth: 214.53
Max. degree: 74

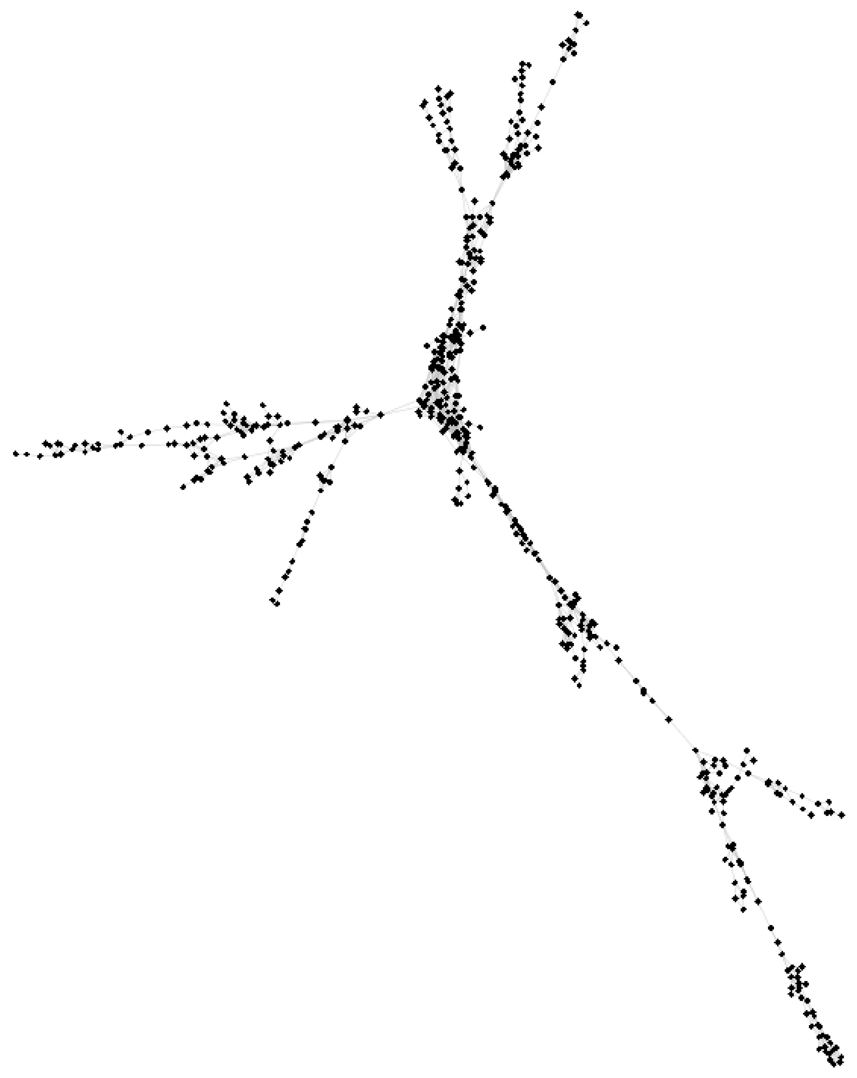

CL153

Number of reads: 518
Number of pairs: 1644
Density: 0.01228
Diameter: NA
Mean edge weigth: 156.76
Max. degree: 26

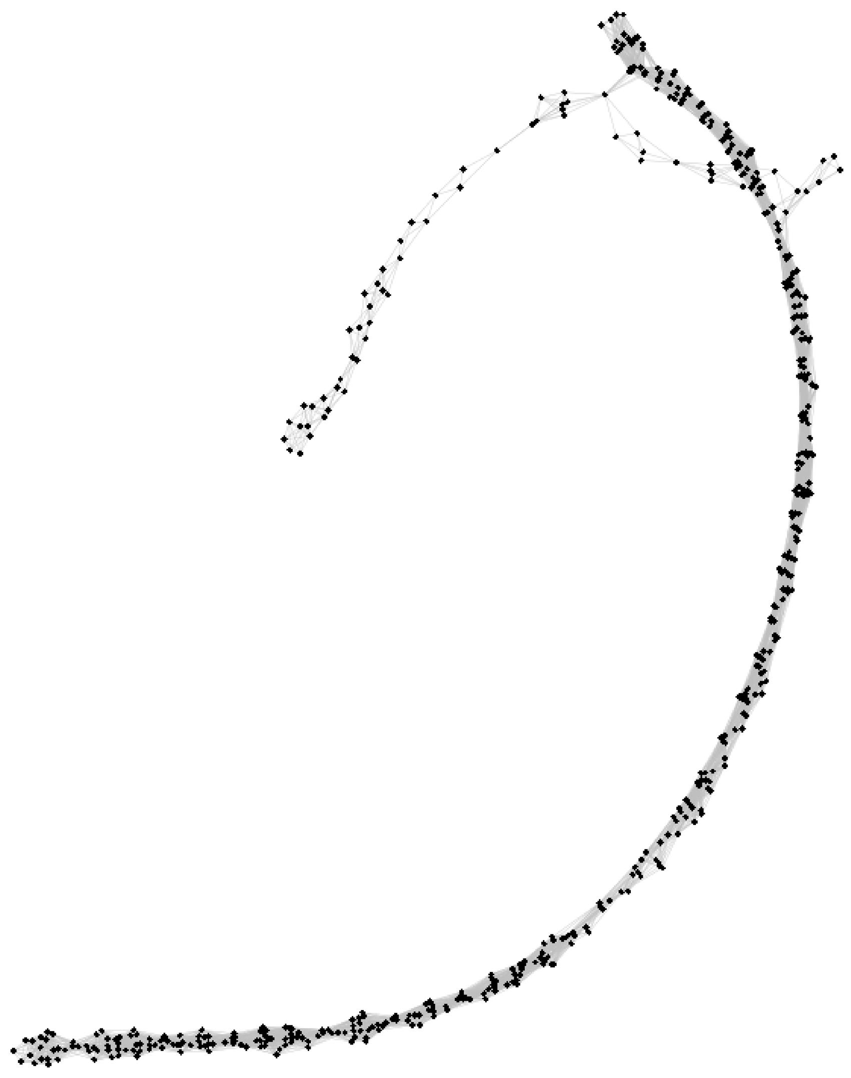

CL154

Number of reads: 514
Number of pairs: 5737
Density: 0.04351
Diameter: NA
Mean edge weigth: 214.33
Max. degree: 32

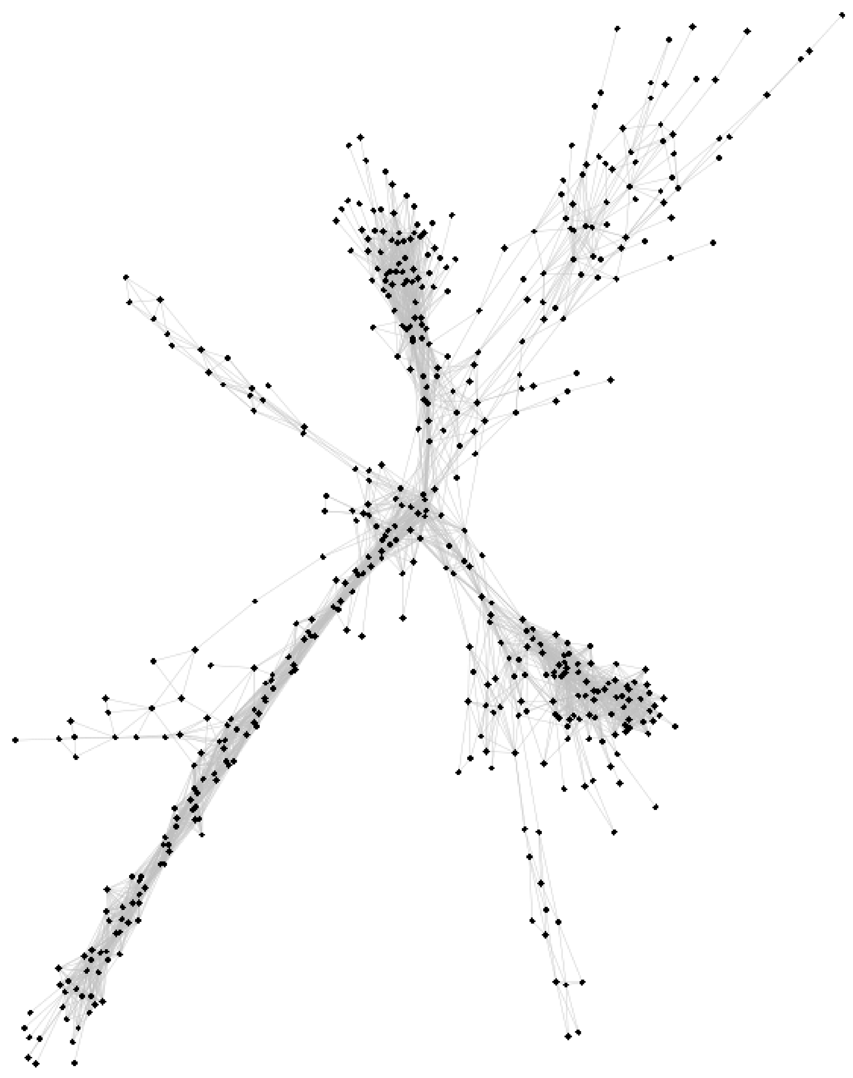

CL155

Number of reads: 483
Number of pairs: 3329
Density: 0.0286
Diameter: NA
Mean edge weigth: 161.46
Max. degree: 46

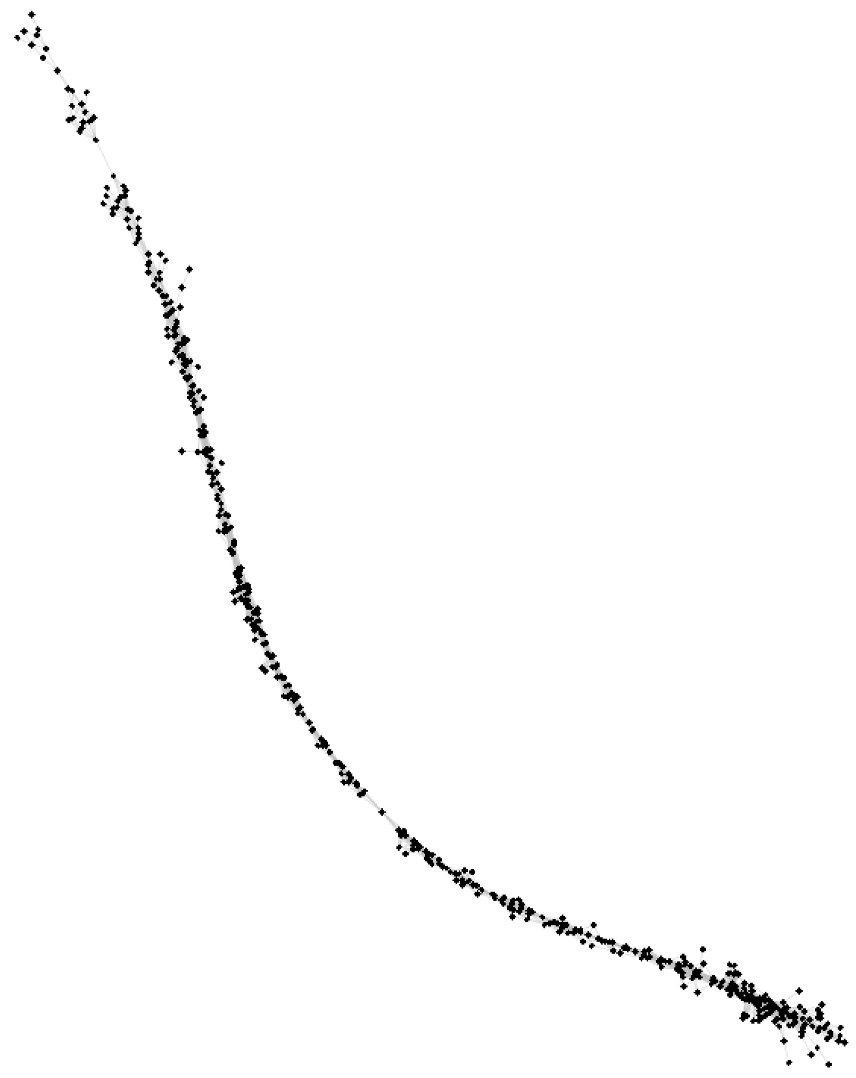

CL156

Number of reads: 478
Number of pairs: 3432
Density: 0.0301
Diameter: NA
Mean edge weigth: 166.07
Max. degree: 40

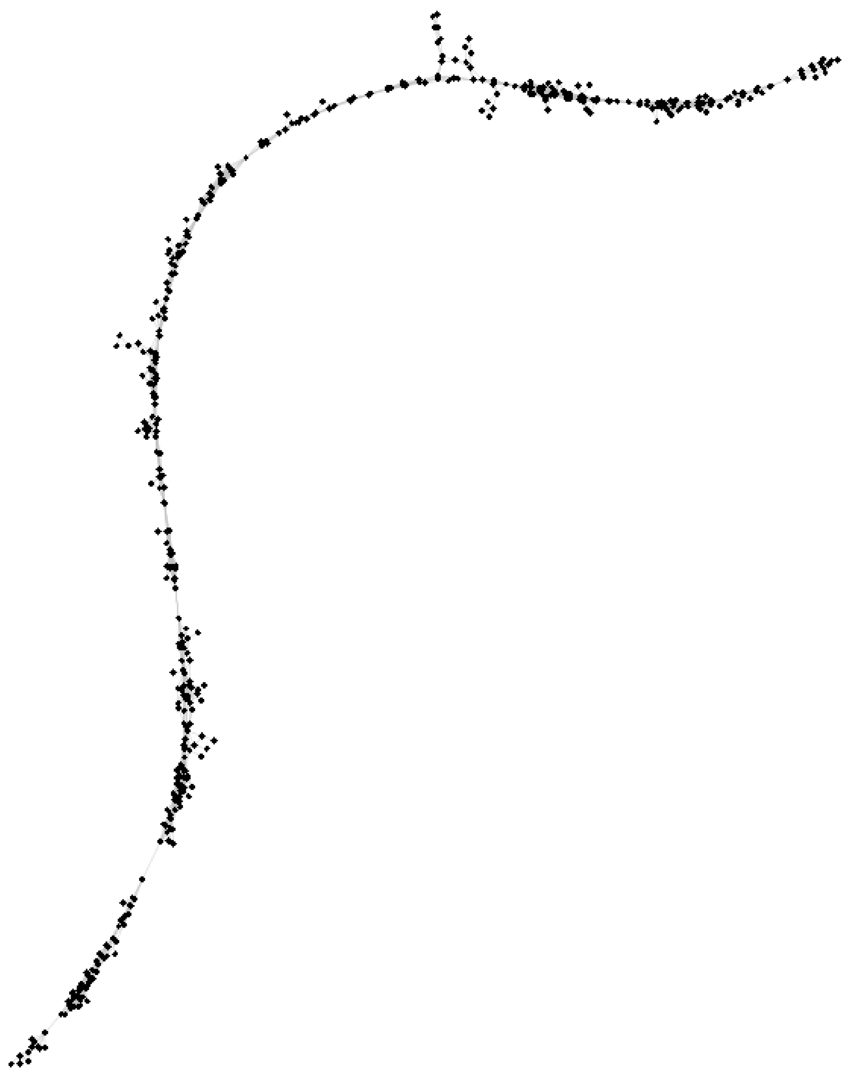

CL157

Number of reads: 470
Number of pairs: 2472
Density: 0.02243
Diameter: NA
Mean edge weigth: 165.36
Max. degree: 28

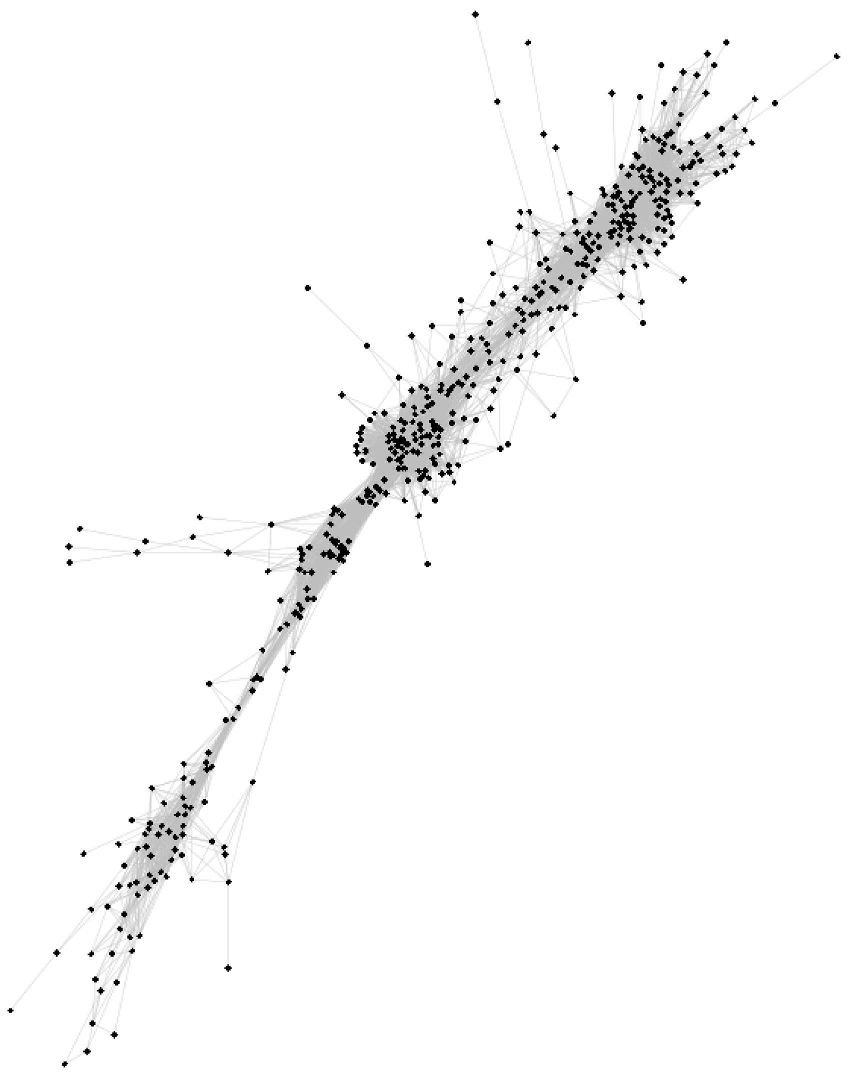

CL158

Number of reads: 463
Number of pairs: 7140
Density: 0.06676
Diameter: NA
Mean edge weigth: 147.16
Max. degree: 99

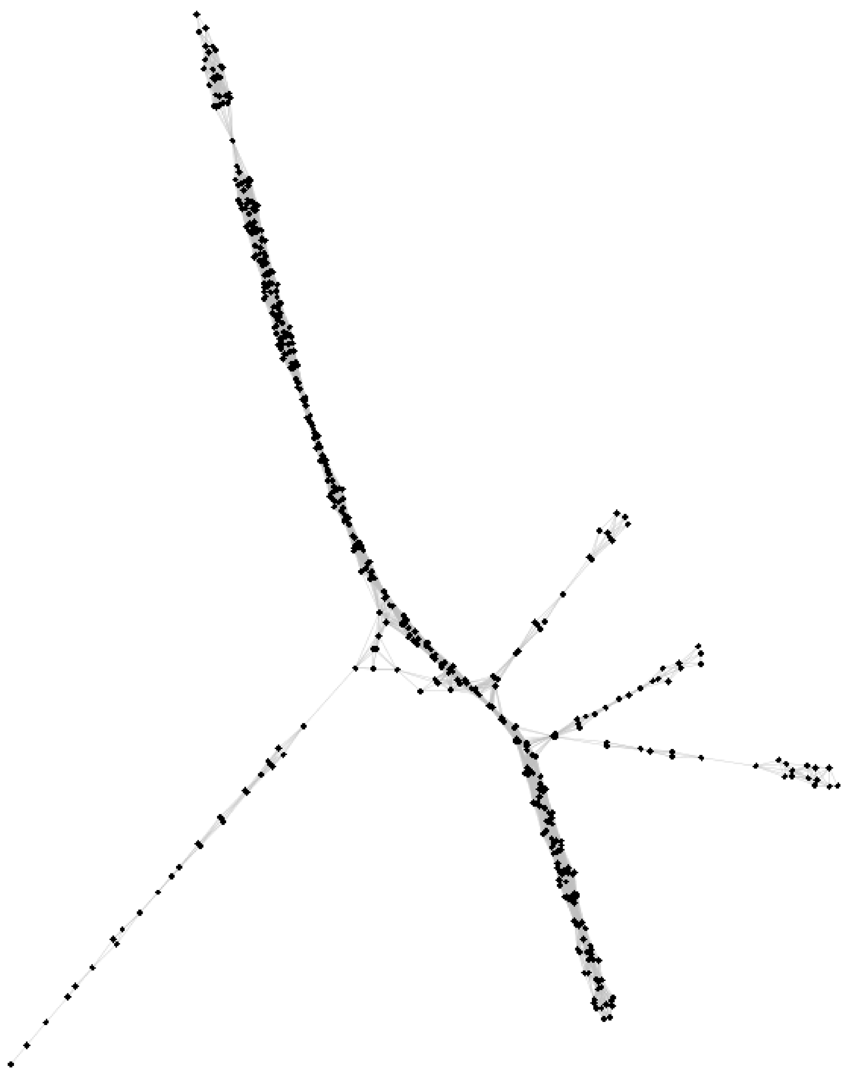

CL159

Number of reads: 455
Number of pairs: 5031
Density: 0.04871
Diameter: NA
Mean edge weigth: 211.91
Max. degree: 41

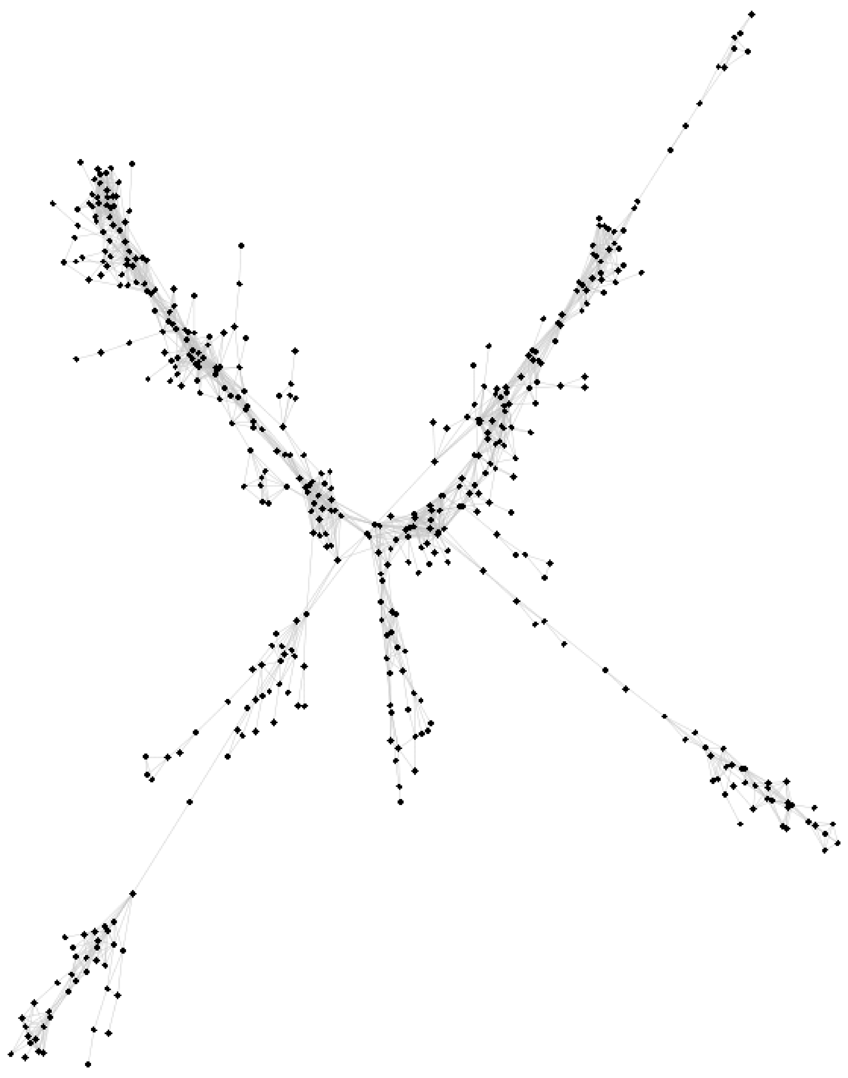

CL160

Number of reads: 441
Number of pairs: 1799
Density: 0.01854
Diameter: NA
Mean edge weigth: 154.83
Max. degree: 30

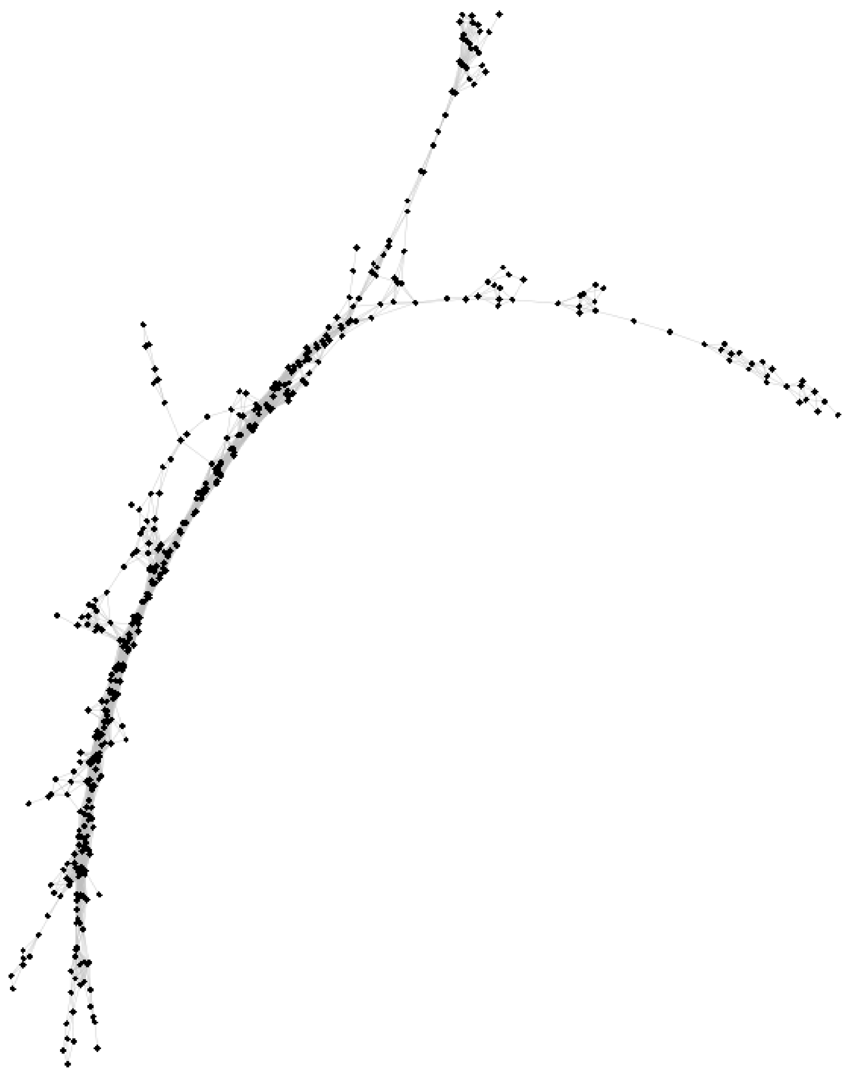

CL161

Number of reads: 431
Number of pairs: 3294
Density: 0.03555
Diameter: NA
Mean edge weigth: 183.29
Max. degree: 32

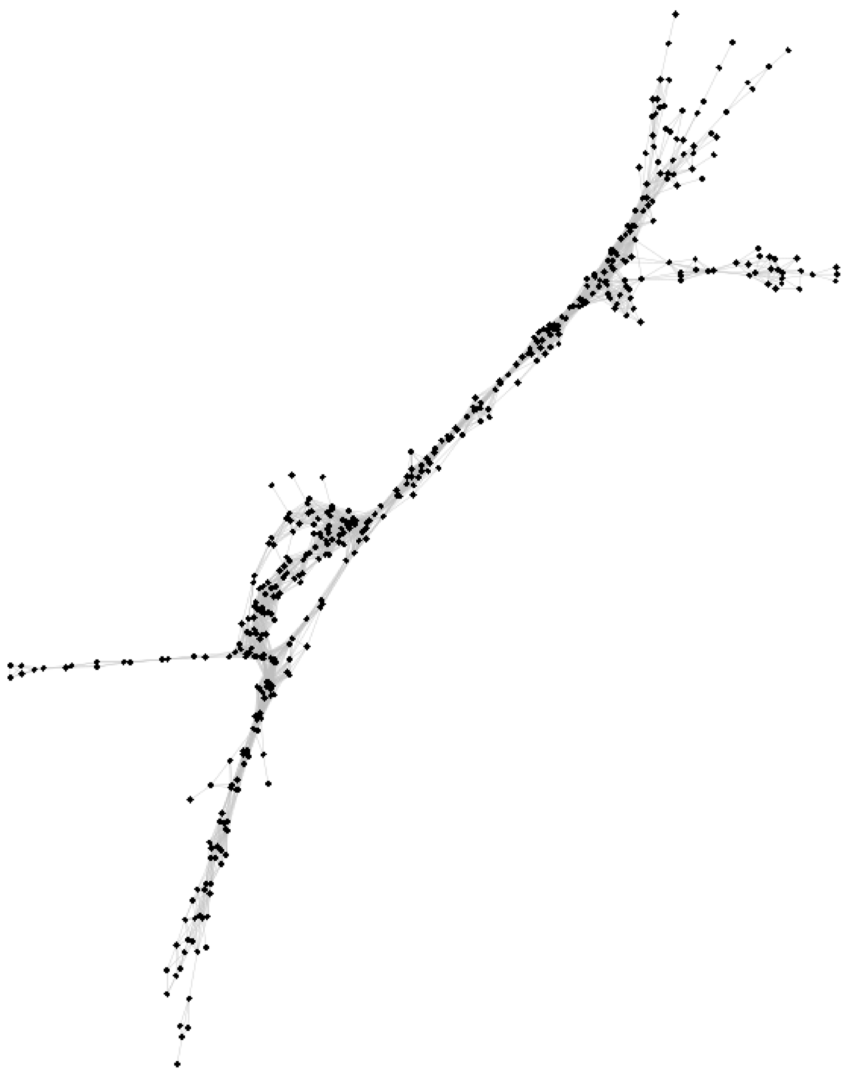

CL162

Number of reads: 422
Number of pairs: 3201
Density: 0.03603
Diameter: NA
Mean edge weigth: 166.48
Max. degree: 35

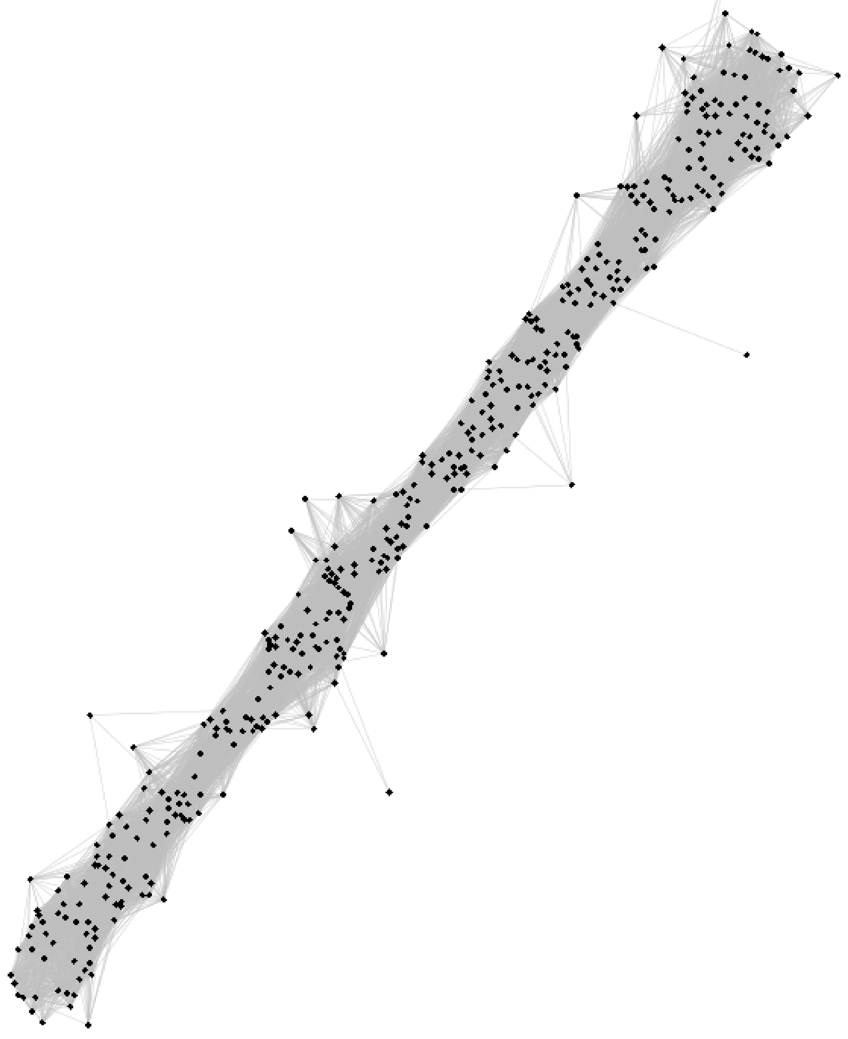

CL163

Number of reads: 392
Number of pairs: 12261
Density: 0.16
Diameter: NA
Mean edge weigth: 166.42
Max. degree: 84

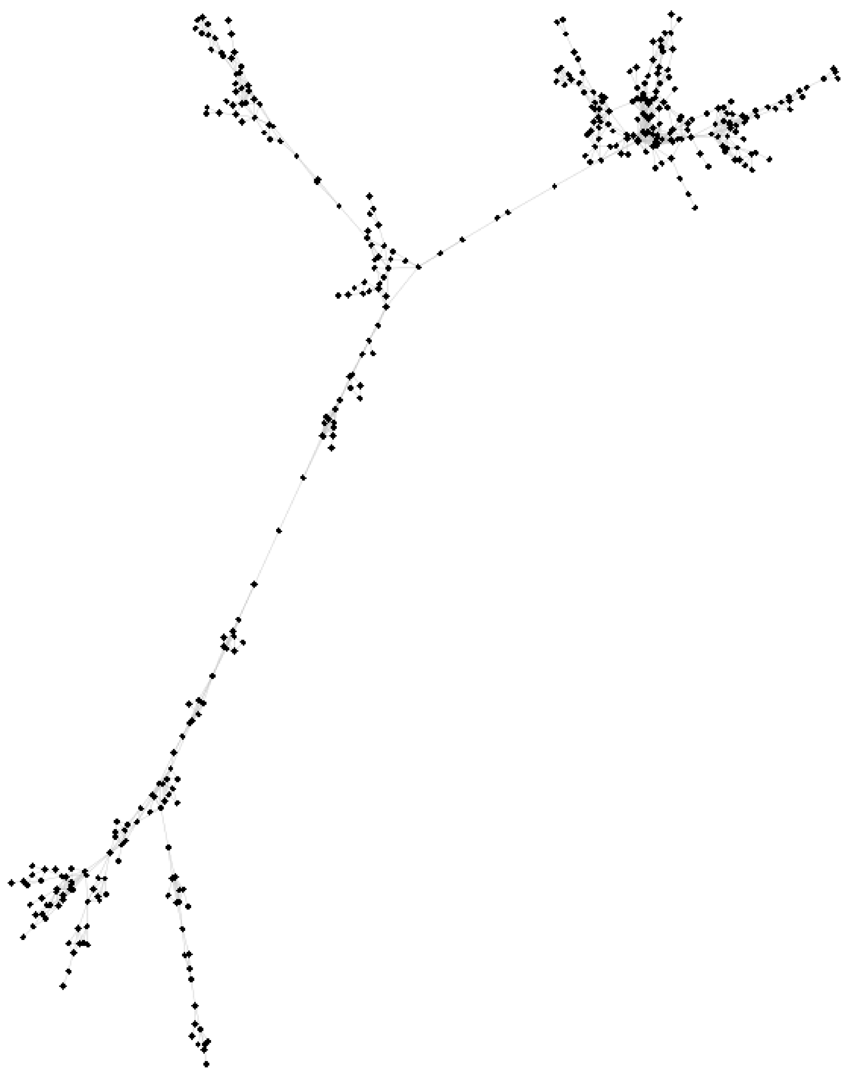

CL164

Number of reads: 373
Number of pairs: 1016
Density: 0.01464
Diameter: NA
Mean edge weigth: 161.3
Max. degree: 20

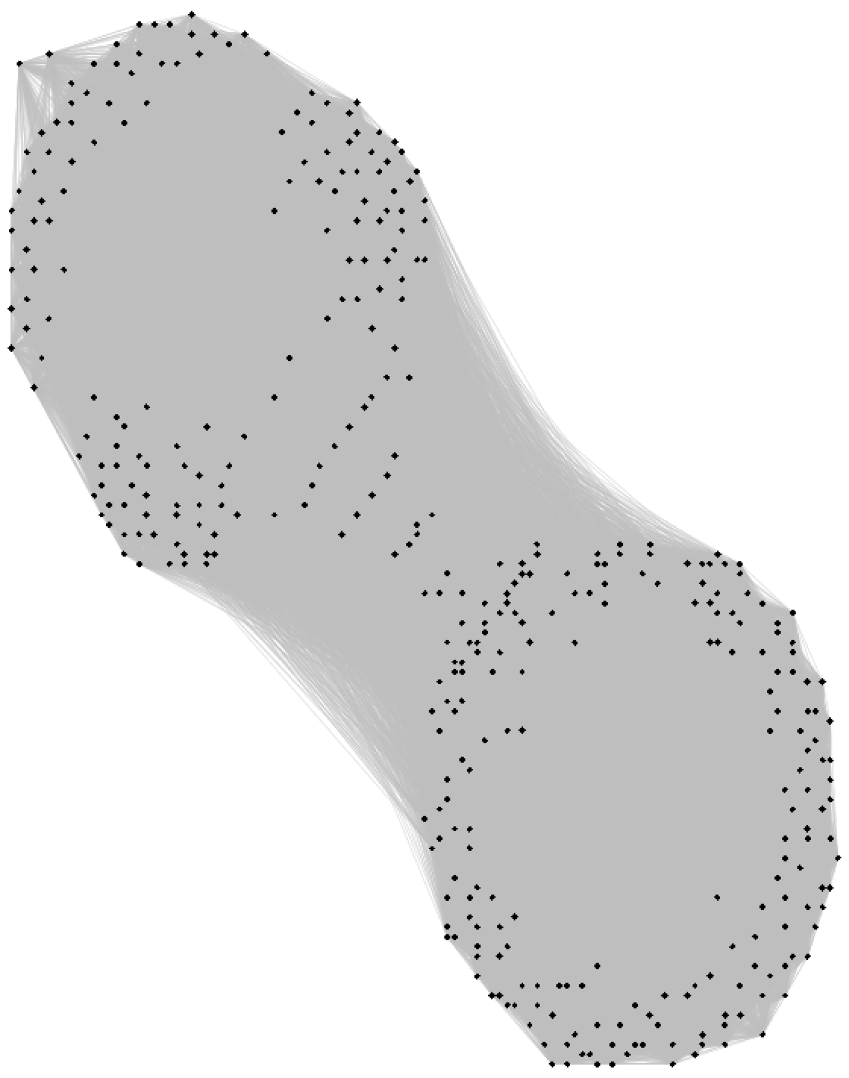

CL165

Number of reads: 372
Number of pairs: 42177
Density: 0.6112
Diameter: NA
Mean edge weigth: 224.38
Max. degree: 343

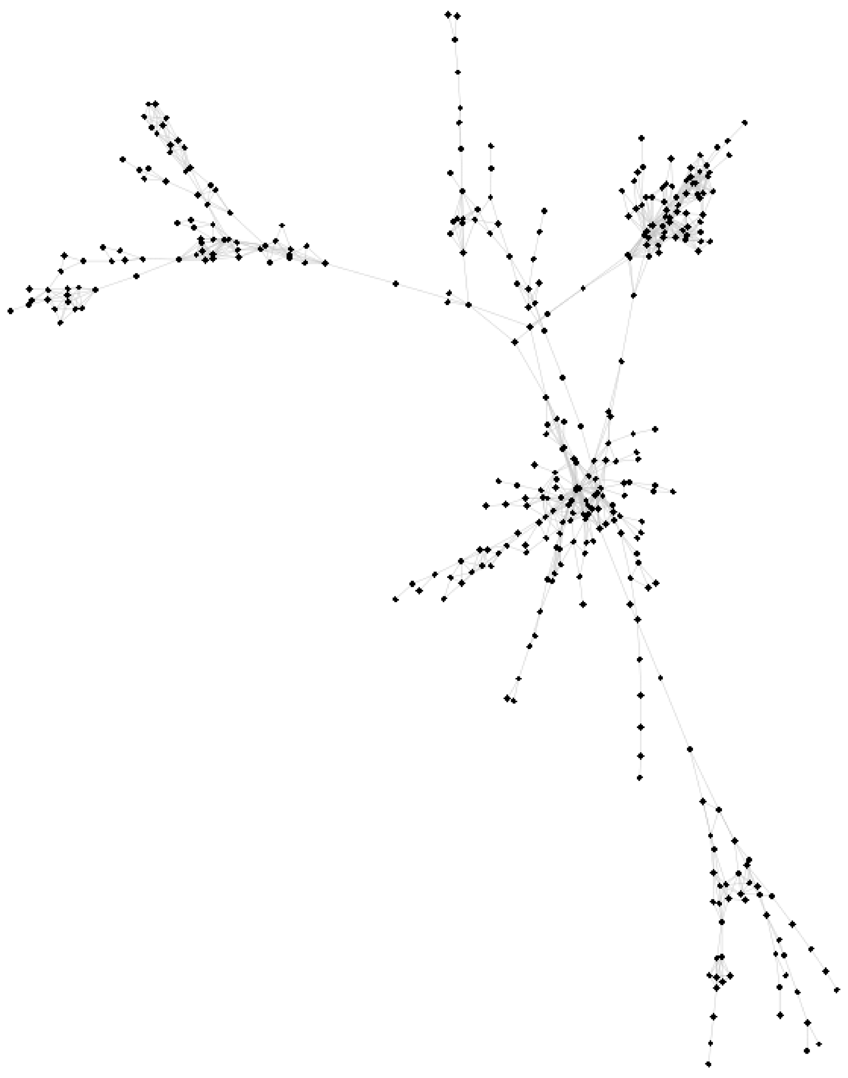

CL166

Number of reads: 358
Number of pairs: 1051
Density: 0.01645
Diameter: NA
Mean edge weigth: 157.02
Max. degree: 24

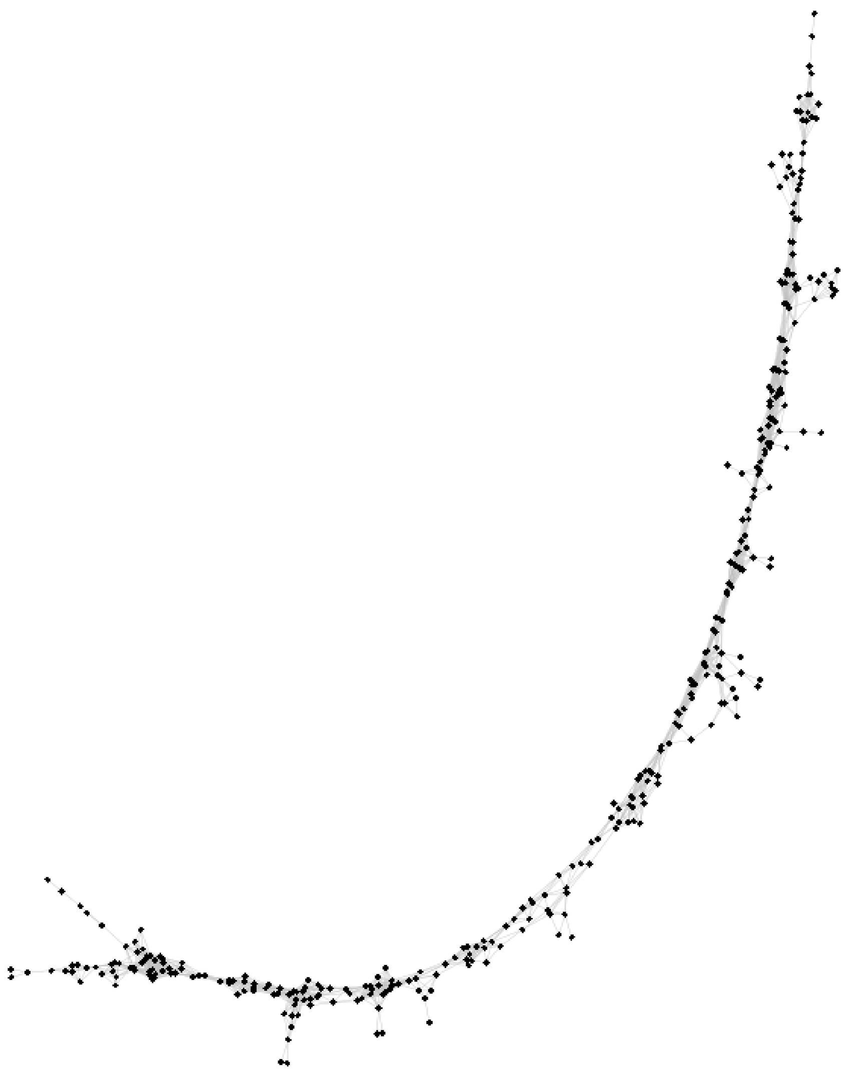

CL167

Number of reads: 342
Number of pairs: 1835
Density: 0.03147
Diameter: NA
Mean edge weigth: 184.75
Max. degree: 23

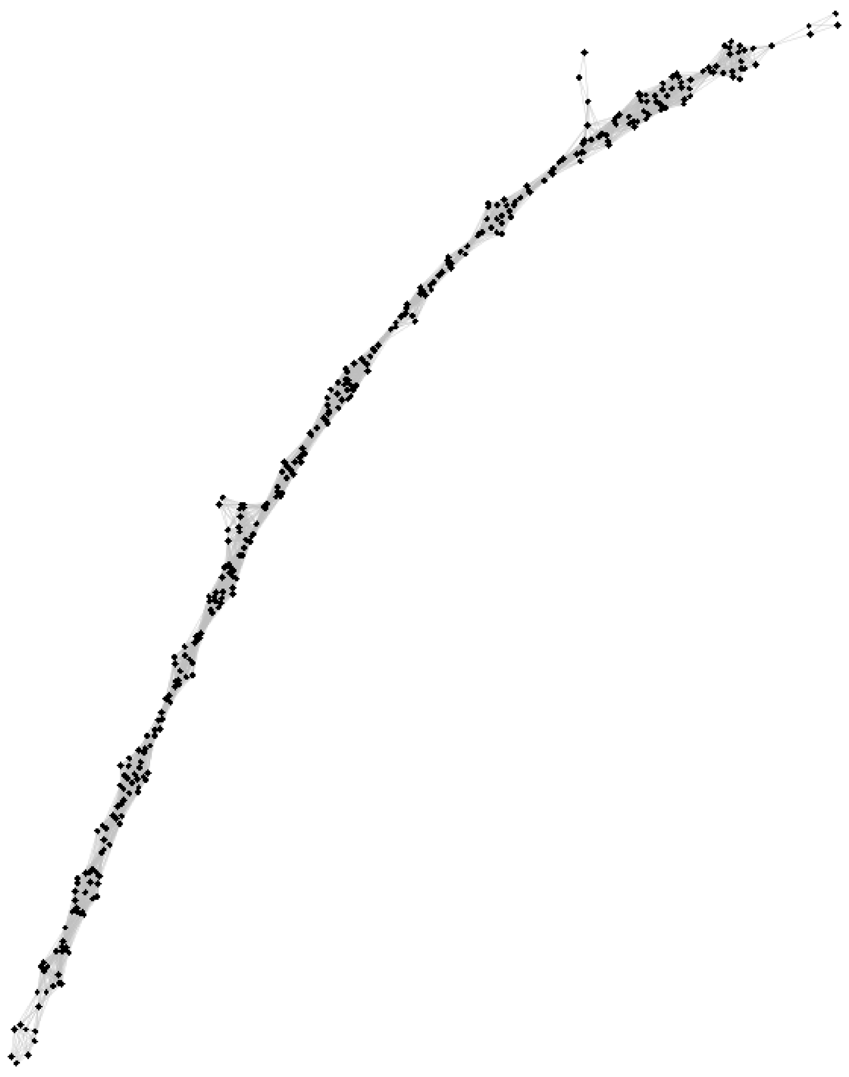

CL168

Number of reads: 340
Number of pairs: 3862
Density: 0.06701
Diameter: NA
Mean edge weigth: 214.66
Max. degree: 34

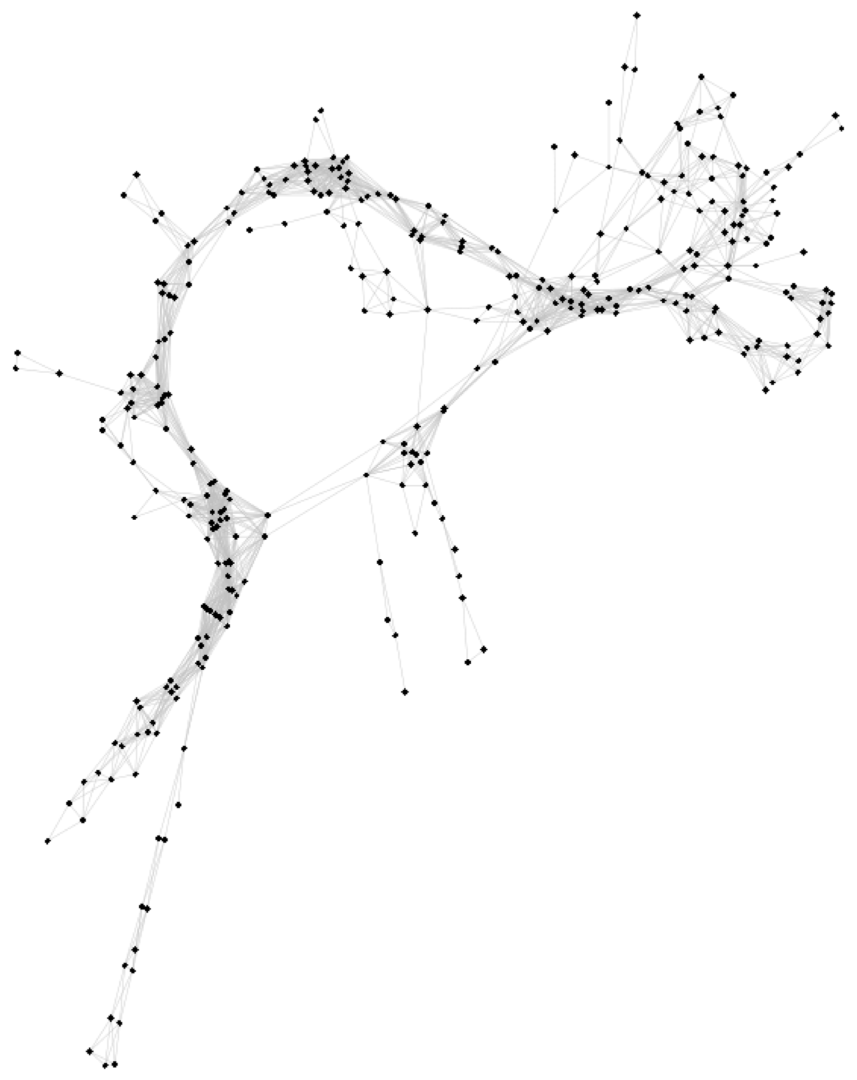

CL169

Number of reads: 332
Number of pairs: 1999
Density: 0.03638
Diameter: NA
Mean edge weigth: 188.36
Max. degree: 27

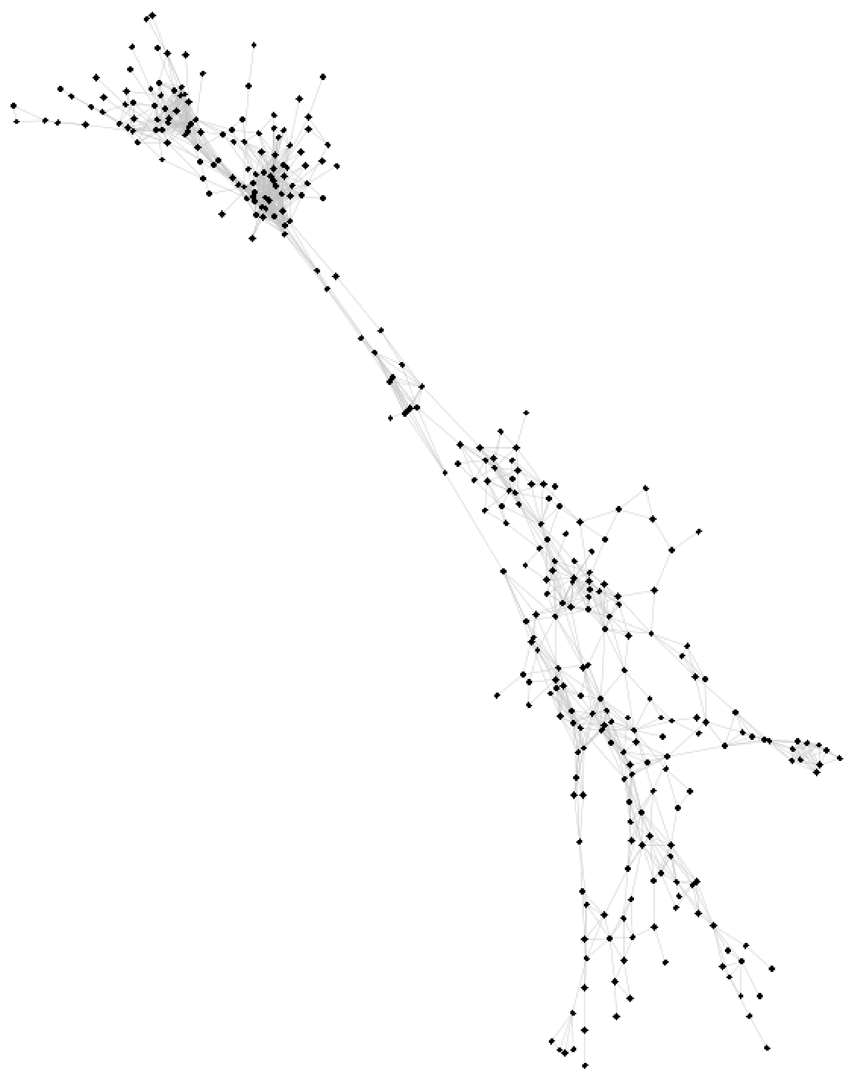

CL170

Number of reads: 323
Number of pairs: 1228
Density: 0.02361
Diameter: NA
Mean edge weigth: 149.6
Max. degree: 33

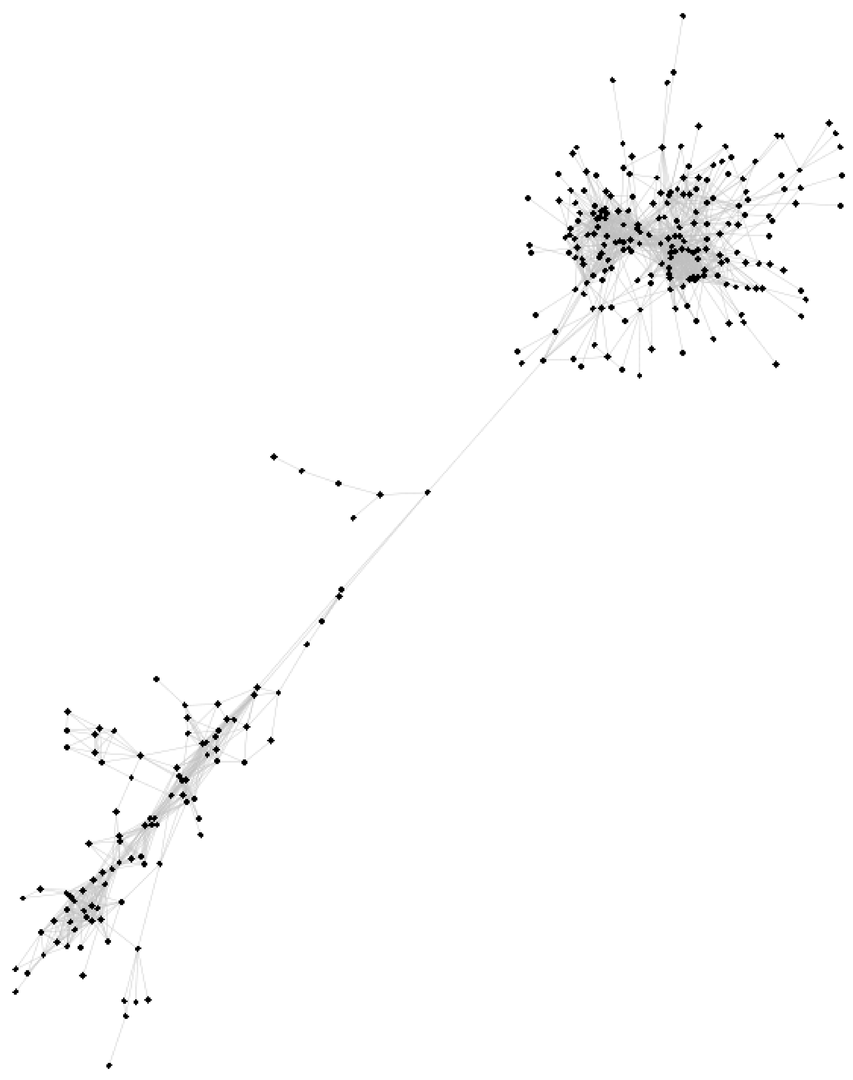

CL171

Number of reads: 302
Number of pairs: 1518
Density: 0.0334
Diameter: NA
Mean edge weigth: 148.55
Max. degree: 50

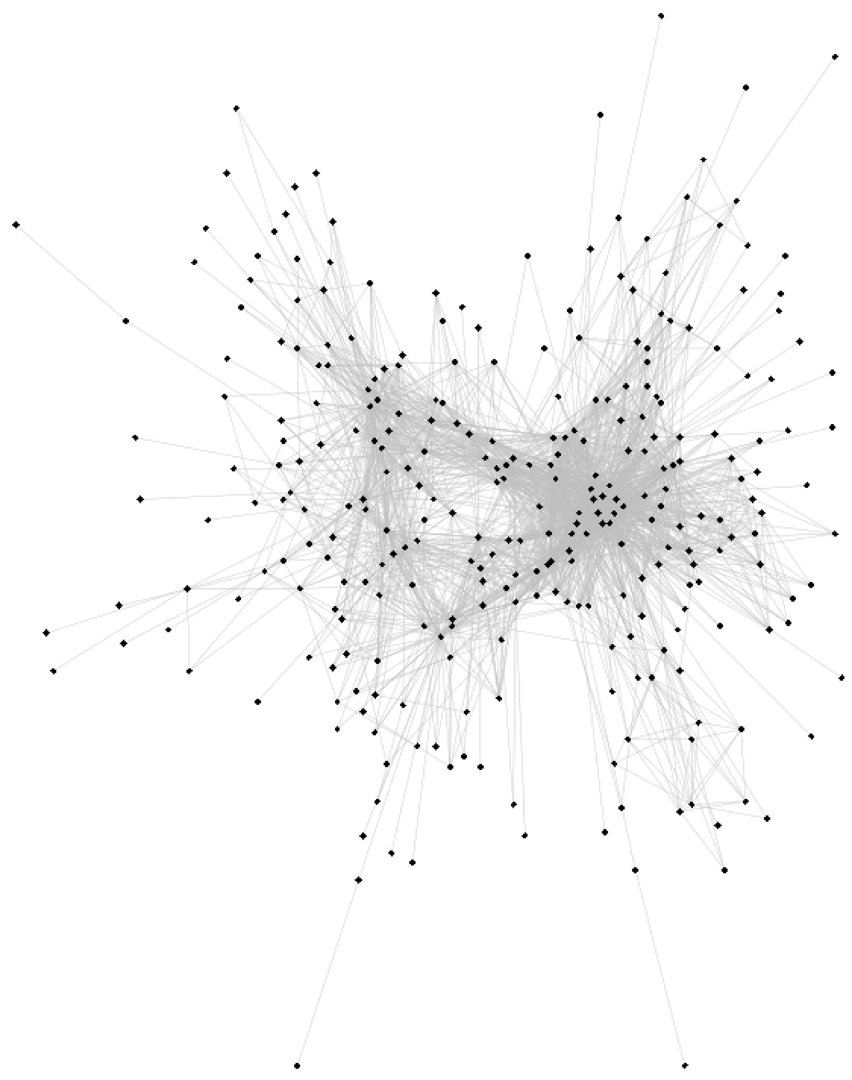

CL172

Number of reads: 299
Number of pairs: 2558
Density: 0.05742
Diameter: NA
Mean edge weigth: 147
Max. degree: 88

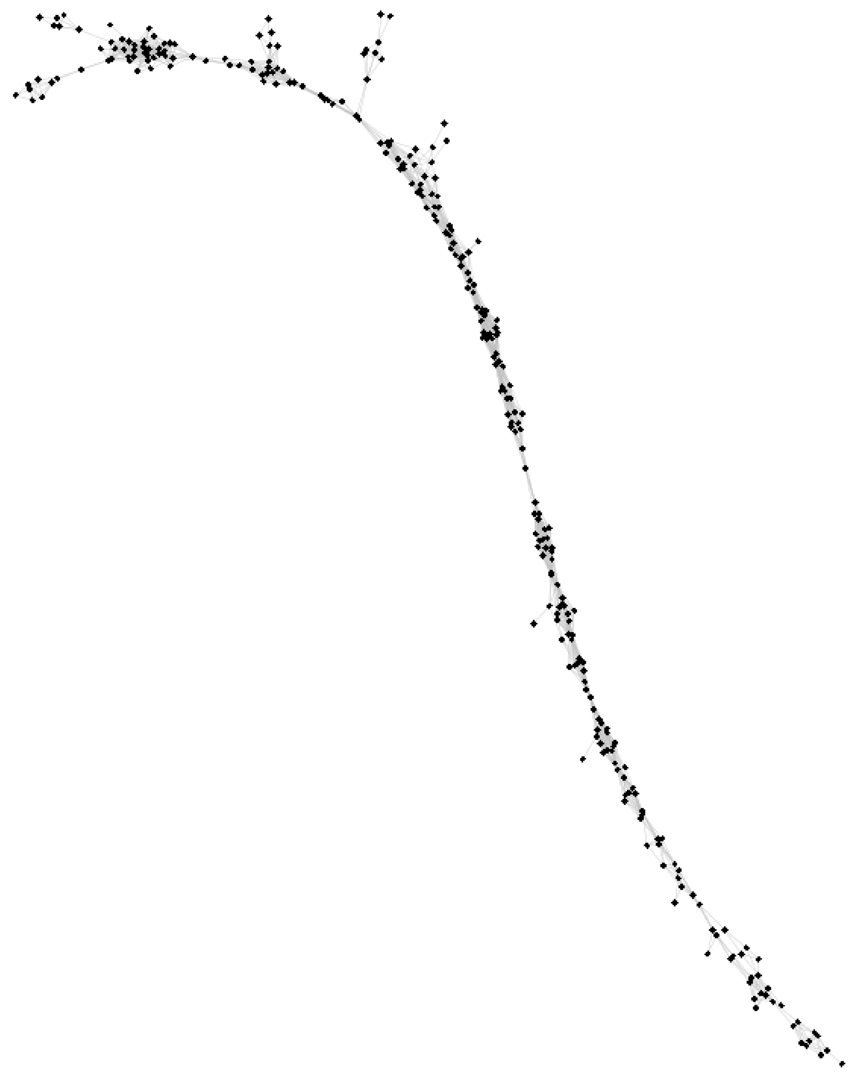

CL173

Number of reads: 282
Number of pairs: 1571
Density: 0.03965
Diameter: NA
Mean edge weigth: 176.03
Max. degree: 23

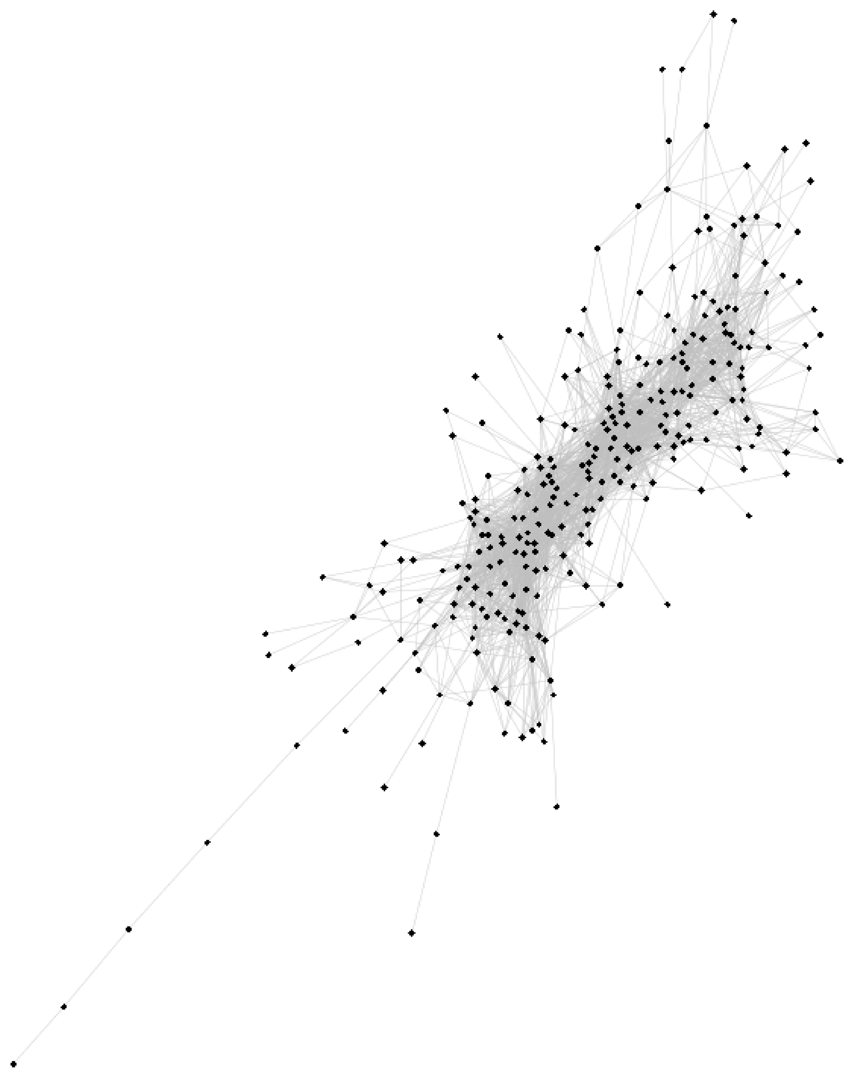

CL174

Number of reads: 277
Number of pairs: 2392
Density: 0.06258
Diameter: NA
Mean edge weigth: 135.3
Max. degree: 68

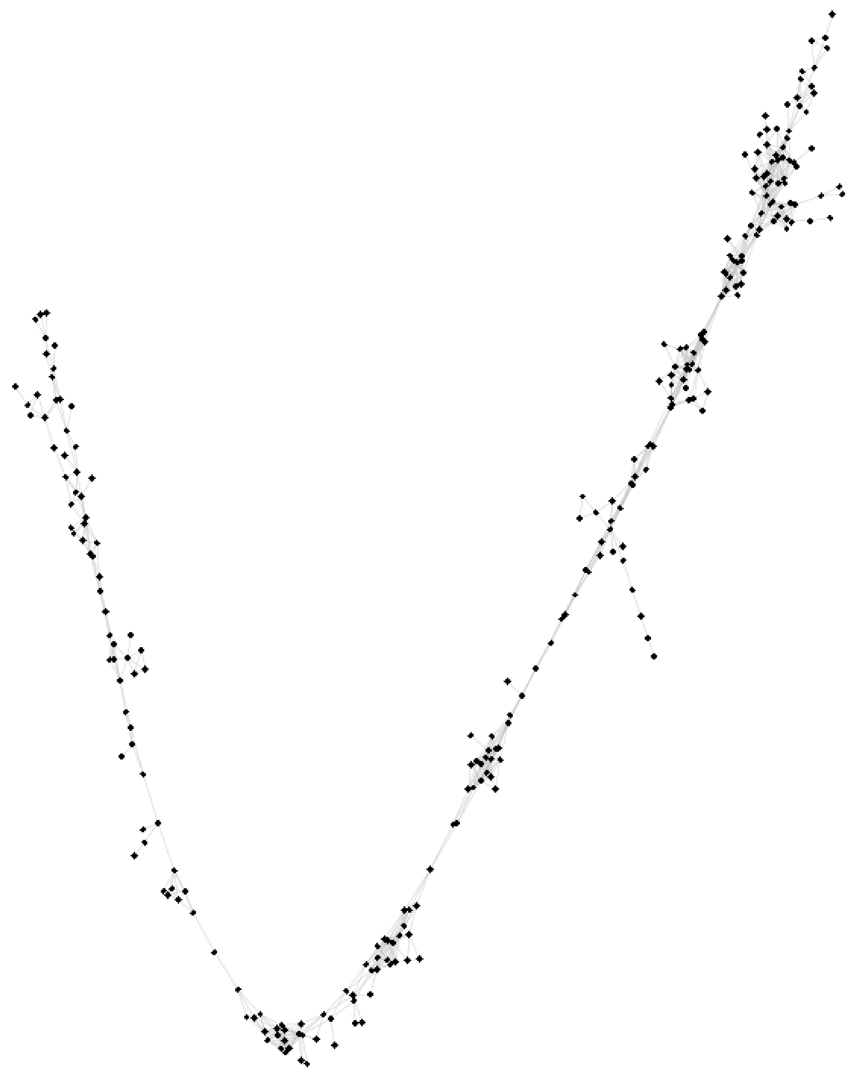

CL175

Number of reads: 270
Number of pairs: 893
Density: 0.02459
Diameter: NA
Mean edge weigth: 145.67
Max. degree: 20

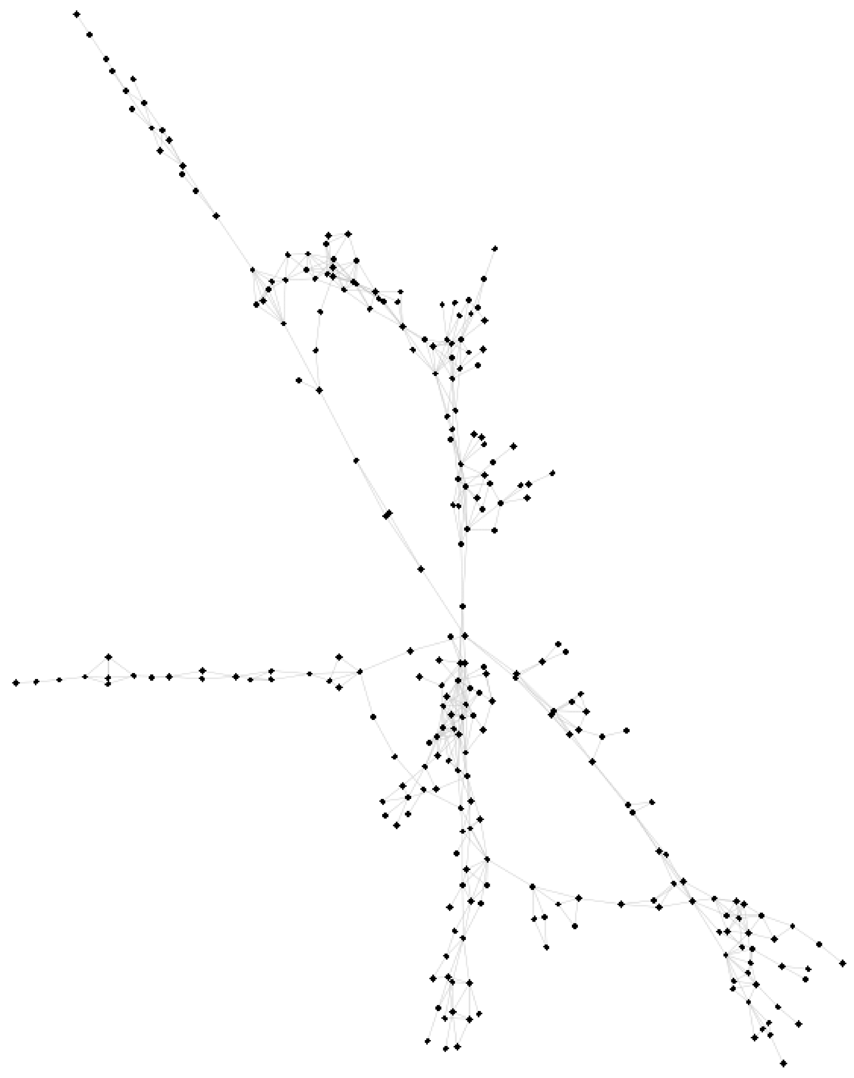

CL176

Number of reads: 258
Number of pairs: 552
Density: 0.01665
Diameter: NA
Mean edge weigth: 159.78
Max. degree: 17

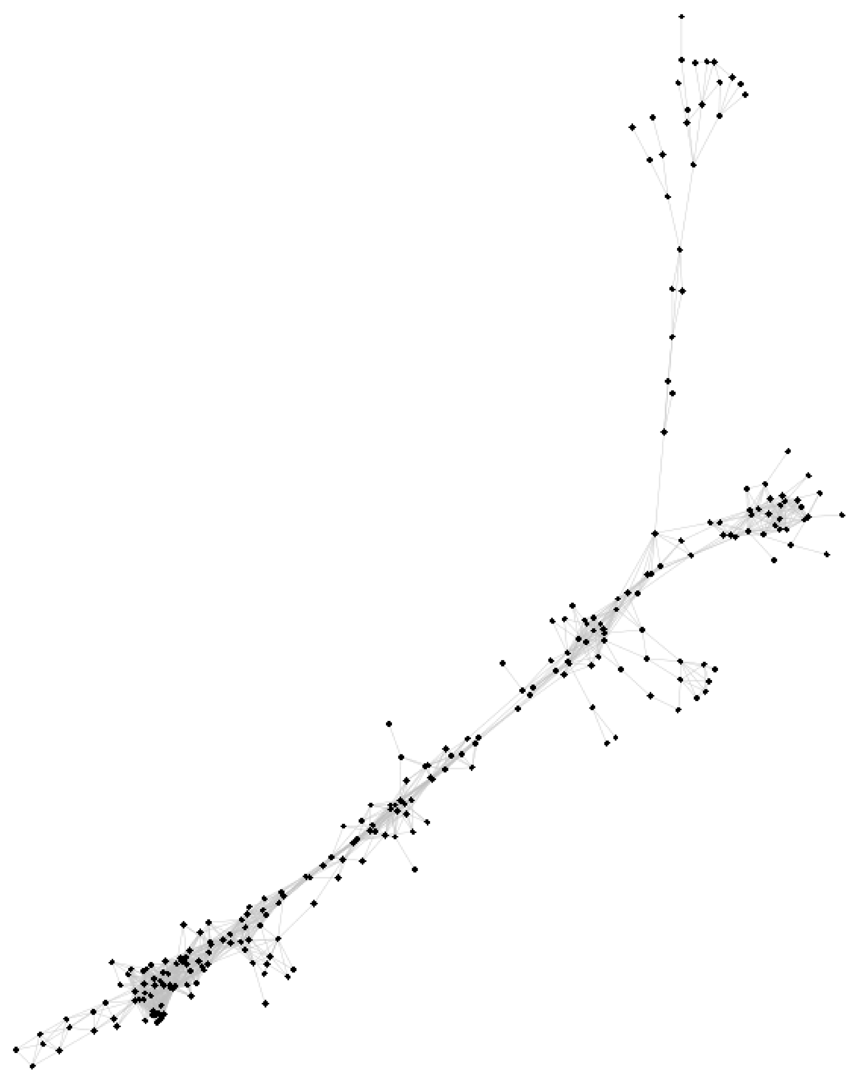

CL177

Number of reads: 245
Number of pairs: 1347
Density: 0.04507
Diameter: NA
Mean edge weigth: 150.82
Max. degree: 40

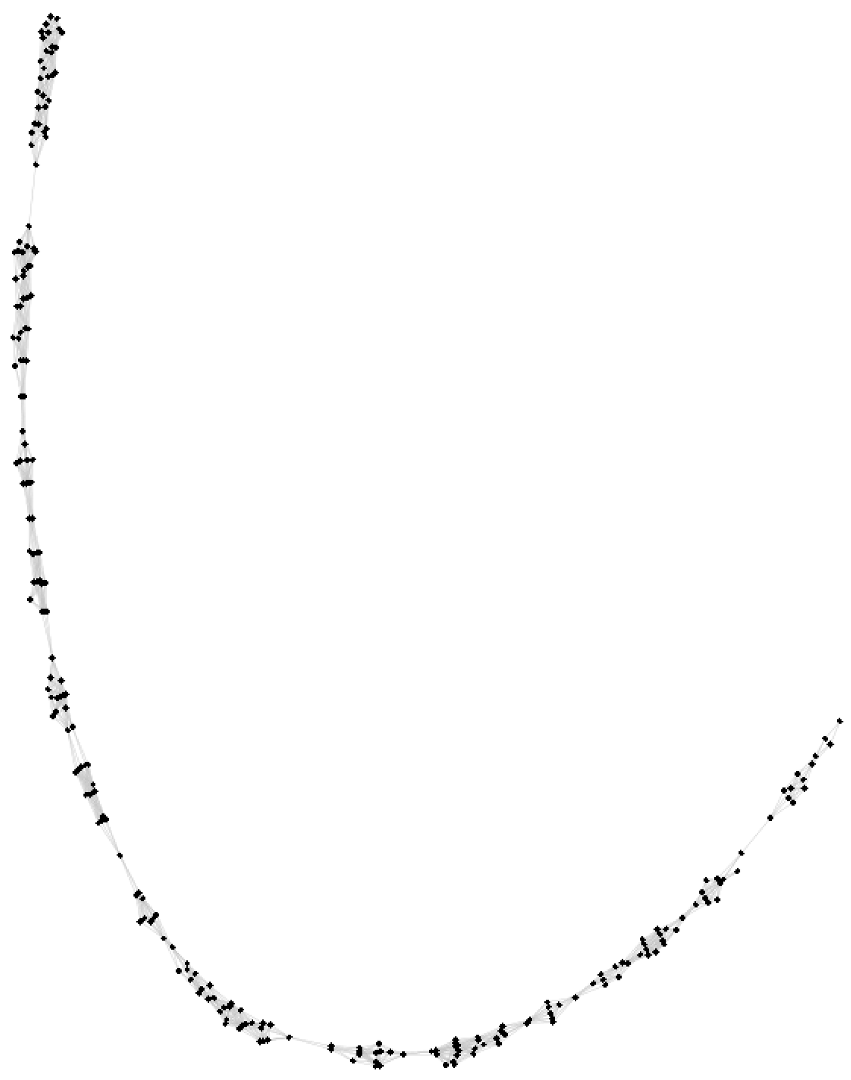

CL178

Number of reads: 242
Number of pairs: 1279
Density: 0.04386
Diameter: NA
Mean edge weigth: 216.22
Max. degree: 17

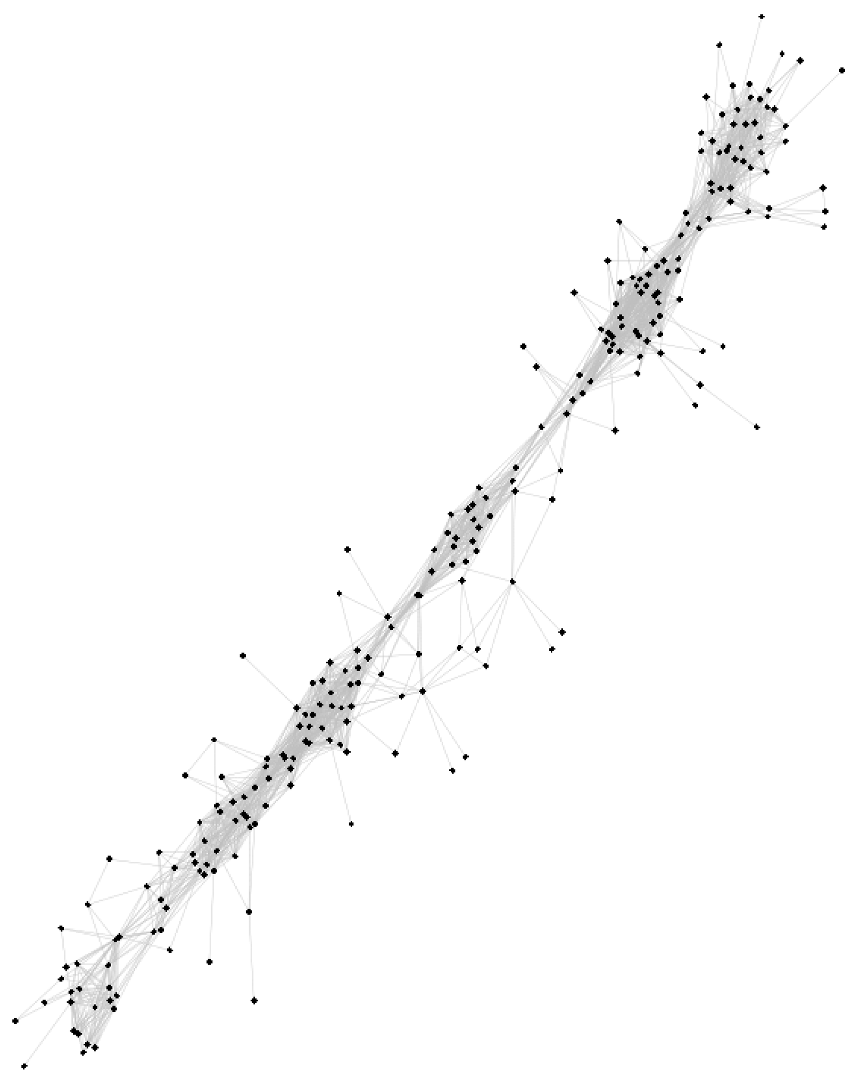

CL179

Number of reads: 240
Number of pairs: 2071
Density: 0.07221
Diameter: NA
Mean edge weigth: 162.24
Max. degree: 39

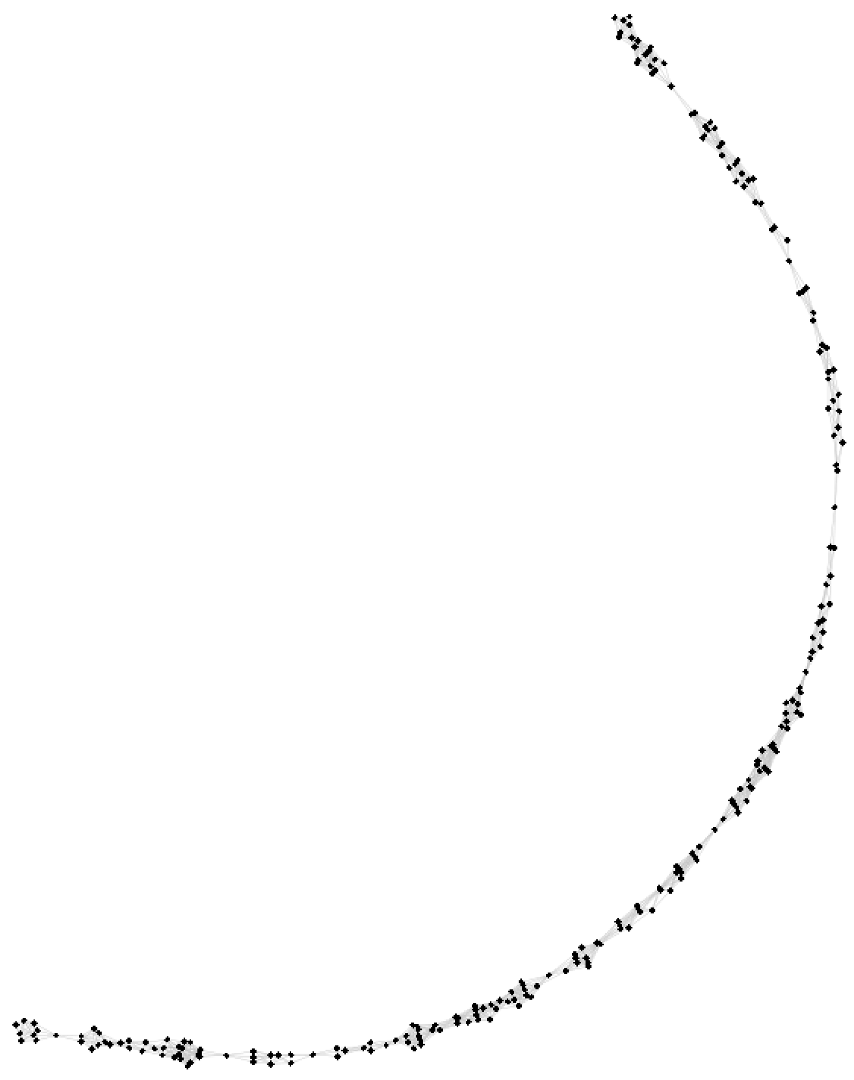

CL180

Number of reads: 235
Number of pairs: 1092
Density: 0.03972
Diameter: NA
Mean edge weigth: 214.3
Max. degree: 16

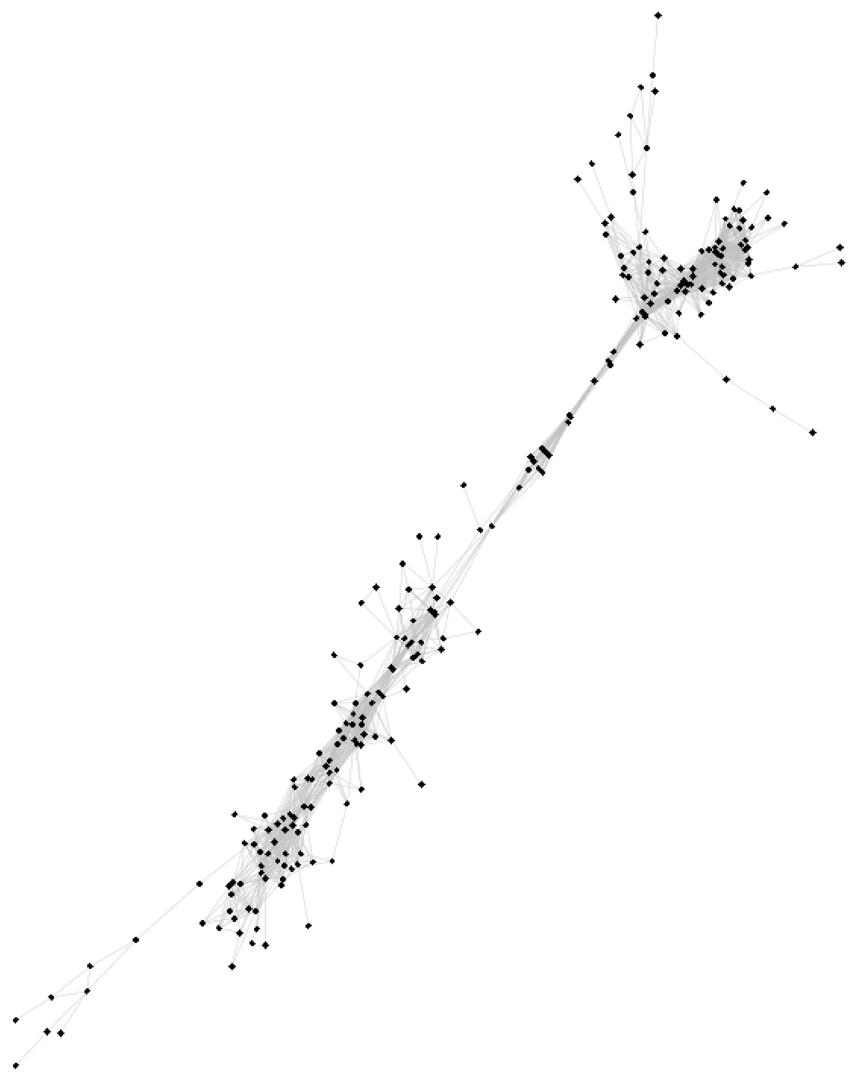

CL181

Number of reads: 231
Number of pairs: 1553
Density: 0.05846
Diameter: NA
Mean edge weigth: 153.57
Max. degree: 40

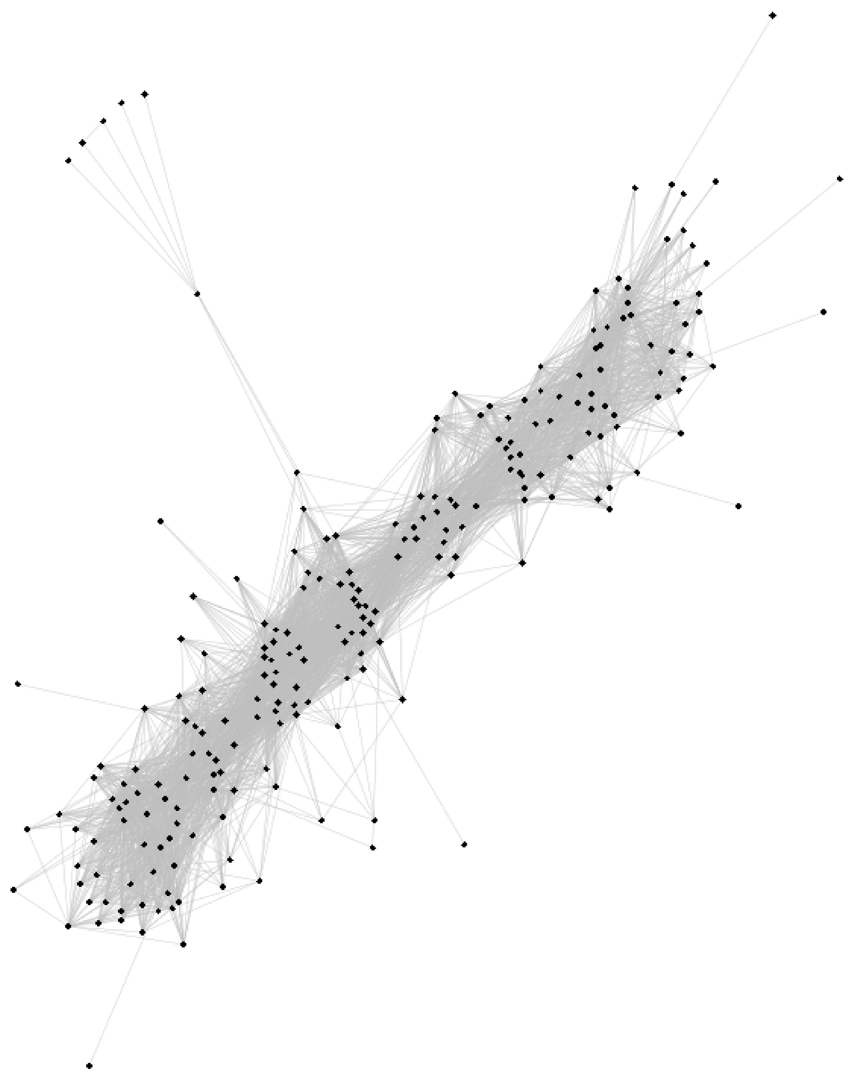

CL182

Number of reads: 224
Number of pairs: 4227
Density: 0.1692
Diameter: NA
Mean edge weigth: 159.37
Max. degree: 70

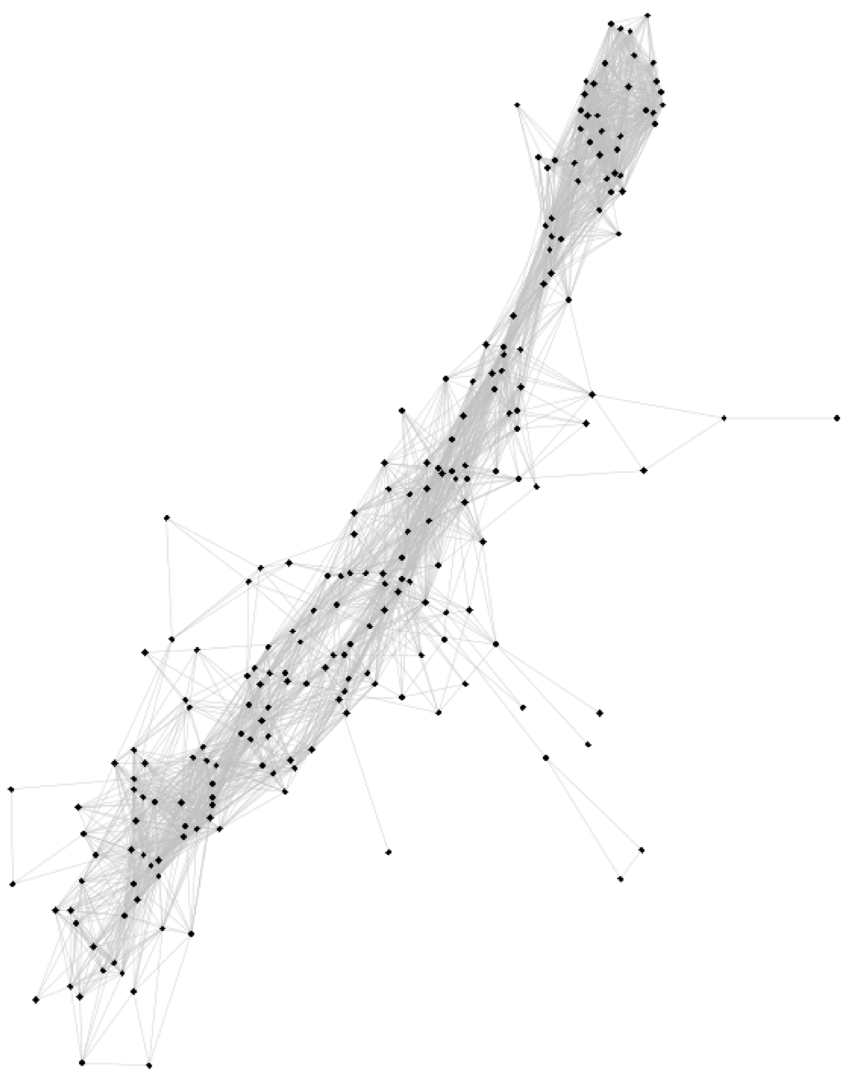

CL183

Number of reads: 211
Number of pairs: 2410
Density: 0.1088
Diameter: NA
Mean edge weigth: 157.69
Max. degree: 47

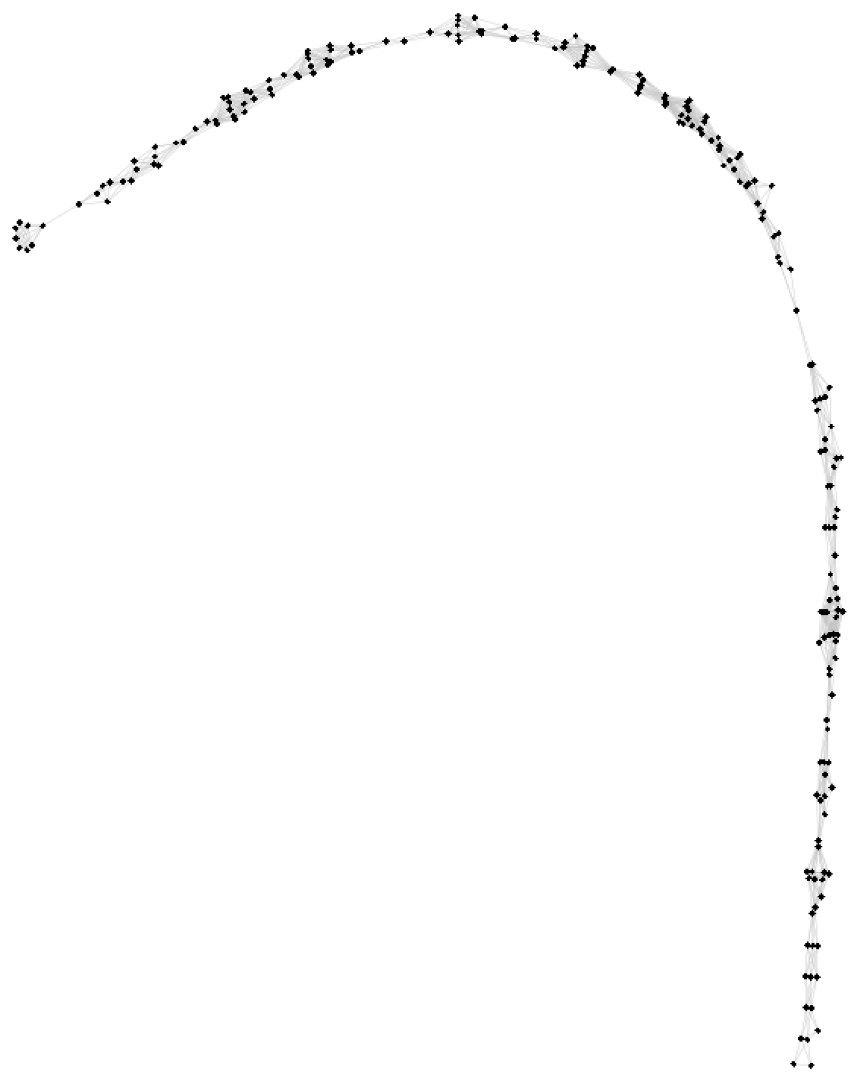

CL184

Number of reads: 211
Number of pairs: 1101
Density: 0.0497
Diameter: NA
Mean edge weigth: 214.92
Max. degree: 18
